# Cobalt-Catalyzed γ‑C–H Functionalization of Alcohols via Olefin-Tethered Radical Relay

**DOI:** 10.1021/jacsau.5c00909

**Published:** 2025-12-11

**Authors:** Phong Dam, Kosala N. Amarasinghe, Chenyang Wang, Olga S. Bokareva, Luis Miguel Azofra, Osama El-Sepelgy

**Affiliations:** † 28392 Leibniz Institute for Catalysis e.V., Albert-Einstein-Str. 29a, 18059 Rostock, Germany; ‡ Instituto de Estudios Ambientales y Recursos Naturales (i-UNAT), Universidad de Las Palmas de Gran Canaria (ULPGC), Campus de Tafira, 35017 Las Palmas de Gran Canaria, Spain; § Institute for Chemistry and Department of Life, Light & Matter, University of Rostock, Albert-Einstein-Str. 25 and 27, 18059 Rostock, Germany

**Keywords:** Cobalt, C−H functionalization, MHAT, Radical relay, Mechanistic study

## Abstract

Herein, we report a cobalt-catalyzed method for γ-selective C–H functionalization of aliphatic alcohols *via* a hydrogen atom transfer (HAT)-based radical relay. This strategy employs a readily available cobalt-salen catalyst under mild conditions, enabling diverse γ-C–H functionalization with excellent site-selectivity. Mechanistic insights were gained through spectroscopic experiments and DFT calculations, confirming the formation of carbon-centered radicals as key intermediates in the reaction pathway. This study offers a powerful tool for site-selective derivatization of alcohols and contributes to the development of sustainable and modular C–H functionalization strategies.

## Graphical abstract

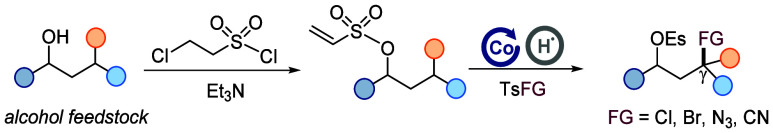

## Introduction

Alcohols are widely available in renewable feedstocks and can be efficiently synthesized through reliable methods, making them attractive and sustainable substrates in organic synthesis.^1,2^ While most strategies target the α-position, selective functionalization of remote C­(sp^3^)–H bonds is challenging due to the inertness of aliphatic C–H bonds and the difficulty in achieving regioselectivity.^3^ One approach involves palladium-catalyzed C–H activation *via* the concerted metalation-deprotonation (CMD) mechanism (Scheme 1a).^4,5^ Although effective, this method is primarily restricted to β-arylation at less hindered primary C–H bonds. Alternative radical strategies,^6–10^ such as the use of alkoxy radical intermediates, have been investigated, but they typically involve harsh conditions and are limited to δ-functionalization via selective 1,5-hydrogen atom transfer (1,5-HAT) as shown in Scheme 1b.^11–24^ However, remote functionalization of aliphatic alcohols at γ-positions, particularly at tertiary sites, remains largely underexplored. Notably, Hartwig and co-workers achieved γ-selective oxygenation of primary C–H bonds,^25,26^ and in 2018, the groups of Roizen, Zare, and others developed a sulfamate ester-guided radical-mediated γ-halogenation and alkylation of aliphatic alcohols using nitrogen-centered sulfamyl radical intermediates.^27–33^ This strategy often requires using noble metal catalysis (iridium and rhodium) or pre-*N*-functionalization of the sulphamates (Scheme 1c). Further efforts to achieve γ-functionalization have involved the use of halogen-containing silicon tethers, as demonstrated by Studer,^34^ Gevorgyan,^35–37^ Zhang,^38,39^ Ackermann,^40^ and our group.^41^

**Scheme 1:**
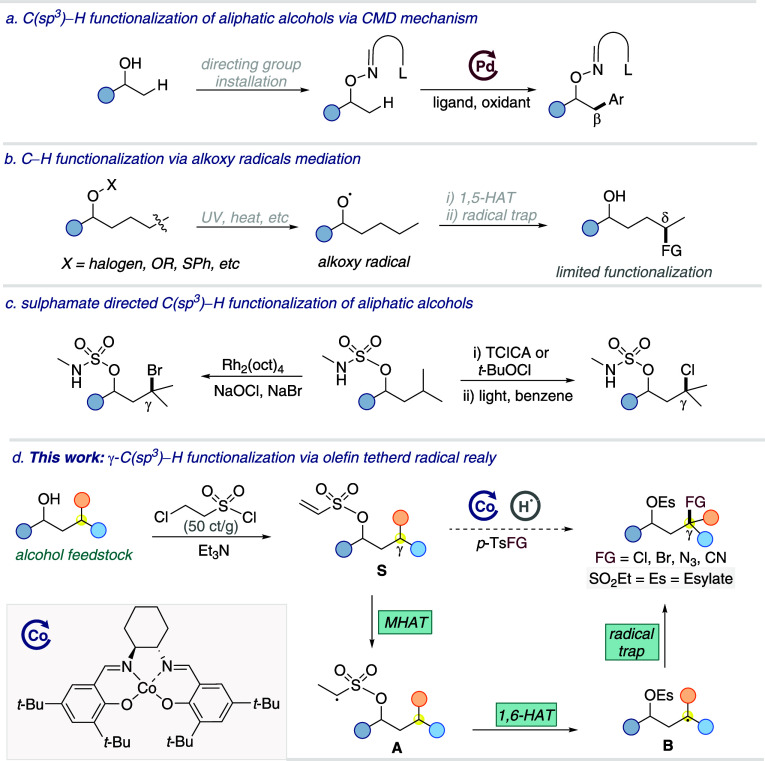
Remote Functionalization of Aliphatic Alcohols

While these approaches expand the toolbox for remote C–H functionalization, they generally lead to desaturation products or are limited to forming C–C or C–N bonds with nucleophilic partners. Given these limitations, there is a clear need for new strategies that enable γ-functionalization of tertiary C–H bonds in aliphatic alcohols using electrophilic partners.

Olefin functionalization *via* metal hydrogen atom transfer (MHAT) offers a powerful platform for generating carbon-centered radicals from unactivated alkenes under mild conditions, enabling the formation of C–C and C–heteroatom bonds with high selectivity.^42–48^ This strategy has facilitated challenging hydrofunctionalizations, as exemplified by Carreira’s hydrochlorination,^49^ hydrocyanacation,^50^ and hydroazidation^51^ of olefins with electrophilic radical traps. Our approach involves the installation of an olefinic tether onto the alcohol substrate, affording the temporary olefin intermediate **S**. Upon treatment with the *in situ* generated [Co]^III^–H species, an electrophilic radical **A** is formed. Subsequent internal 1,6-HAT yields a more stable, translocated C-centered radical **B**, which can be selectively intercepted by a variety of electrophilic radical traps, thus enabling γ-functionalization with high efficiency and selectivity (Scheme 1d).

## Results and Discussion

Our investigation commenced with the design and selection of a suitable olefin-based tether, guided by several key criteria: cost-effectiveness, ease of installation and subsequent removal, and the capacity to generate a secondary, electrophilic carbon radical upon initial functionalization, thereby facilitating efficient C-to-C radical translocation to a more stable tertiary C-radical. Crucially, to favor the desired 1,6-HAT over potential competing pathways like 1,5-HAT, the tether architecture needed to accommodate a 7-membered transition state geometry, often facilitated by incorporating heteroatoms such as sulfur or silicon.^7^ Based on these considerations, we decided to convert the parent aliphatic alcohols into their corresponding vinyl sulfonyl esters.^52^ This transformation was readily achieved using commercially available and inexpensive chloroethanesulfonyl chloride in the presence of triethylamine (Scheme 1d). The resulting ethyl sulfonyl ester groups (OEs) serve as activated alcohol surrogates, analogous to alkyl bromides, and can be efficiently deprotected to liberate the corresponding alcohol either through base-catalyzed nucleophilic substitution^53,54^ or by treatment with methylmagnesium bromide.^55^

With the tethering strategy established, we initiated our studies using 4-methylpentanol derivative **S1** as the model substrate for γ-chlorination, employing *p*-toluenesulfonyl chloride (*p*-TsCl) as chlorinating reagent. Extensive optimization studies (summarized in Table 1) identified the combination of the commercially available cobalt­(salen) complex **Co-1** and phenylsilane in a DCE:*t*-BuOH solvent mixture as the optimal conditions, affording the desired γ-chlorinated product **1** in 84% isolated yield (Table 1, entry 1). While a cobalt–porphyrin catalyst (**Co-2**) also provided good yield (76%, Table 1, entry 2), a cobaloxime catalyst (**Co-3**) under blue light irradiation proved ineffective (Table 1, entry 3).^56^ Control experiments systematically demonstrated the necessity of each reaction component – the cobalt catalyst, silane reductant, and *t*-butanol as cosolvent – for successful product formation (Table 1, entries 4 and 5). It is likely that the advantage of addition of *t*-butanol is due to facilitating the oxidation of the [Co]^II^ precatalyst *via* the quenching of the *in situ* formed sulphonyl radicals.^49^ Conducting the reaction under air or in the presence of water resulted in a pronounced decrease in yield (Table 1, entries 6 and 7). Phenylsilane was selected as the preferred silane source due to its favorable cost and air stability compared to alternatives (Table 1, entries 8 and 9). The addition of external oxidants did not improve the yield (Table 1, entry 10), whereas reducing the stoichiometry of *p*-TsCl or lowering the reaction temperature significantly diminished the reaction efficiency (Table 1, entries 11 and 12). The solvent choice was also critical, with *t*-butanol alone providing a lower yield than the optimized DCE:*t*-BuOH mixture (Table 1, entry 13). Finally, alternative chlorinating agents yielded only moderate amounts of the desired product (Table 1, entries 14–16).

**Table 1:**
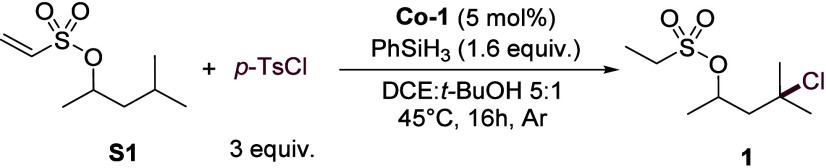
Optimization for γ-Chlorination of Aliphatic Alcohols

**entry**	**deviation from the standard conditions** [Table-fn t1fn2]	**yield (%)**
1	none	90 (84)
2	**Co-2** instead of **Co-1**	76
3	**Co-3** instead of **Co-1**, blue light, no silane	0
4	no **Co-1** or PhSiH_3_	0
5	no *t*-butanol	0
6	under air	32
7	addition of 1 equiv. of H_2_O	47
8	Et_3_SiH instead of PhSiH_3_	85
9	PhSi(O*i*-Pr)H_2_ instead of PhSiH_3_	0
10	with Selectfluor (16 mol %)	88
11	1 equiv of *p*-TsCl	32
12	RT instead of 45 °C	25
13	only *t*-butanol as solvent	60
14	NCS instead of *p*-TsCl	48
15	DCDMH instead of *p*-TsCl	30
16	*N*-chlorophthalimide instead of *p*-TsCl	40

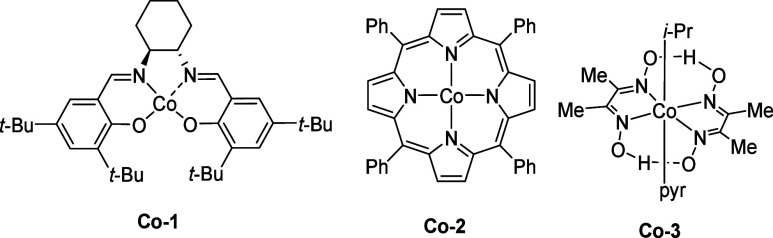

a Standard conditions: **S1** (0.2 mmol), **Co-1** (0.01 mmol, 6 mg), PhSiH_3_ (0.32 mmol, 40 μL), DCE (2.5 mL), *t*-BuOH (0.5 mL), 45 ^ο^C, 16 h, ^1^H NMR yields, isolated yields in parentheses.

Having established robust conditions for γ-chlorination, we next explored the generality of this cobalt-catalyzed radical relay strategy by extending it to other synthetically valuable γ-functionalizations, including bromination, cyanation, and azidation. As depicted in Scheme 2, the methodology proved remarkably versatile, successfully accommodating a diverse range of electrophilic radical traps including *p*-TsBr, *p*-TsCN and *p*-TsN_3_. To the best of our knowledge, this is the first example of remote cyanation and azidation of alcohols. We evaluated the substrate scope using a series of 14 vinyl sulfonyl-tethered alcohols (**S1**–**S14**). GC and TLC analyses of the reaction mixtures consistently indicated high conversion, with no detectable volatile side products arising from reduction, desaturation, dimerization, or regioisomer formation. Polymerization may occur, but the resulting high-MW byproducts are not detectable by GC or TLC.

**Scheme 2:**
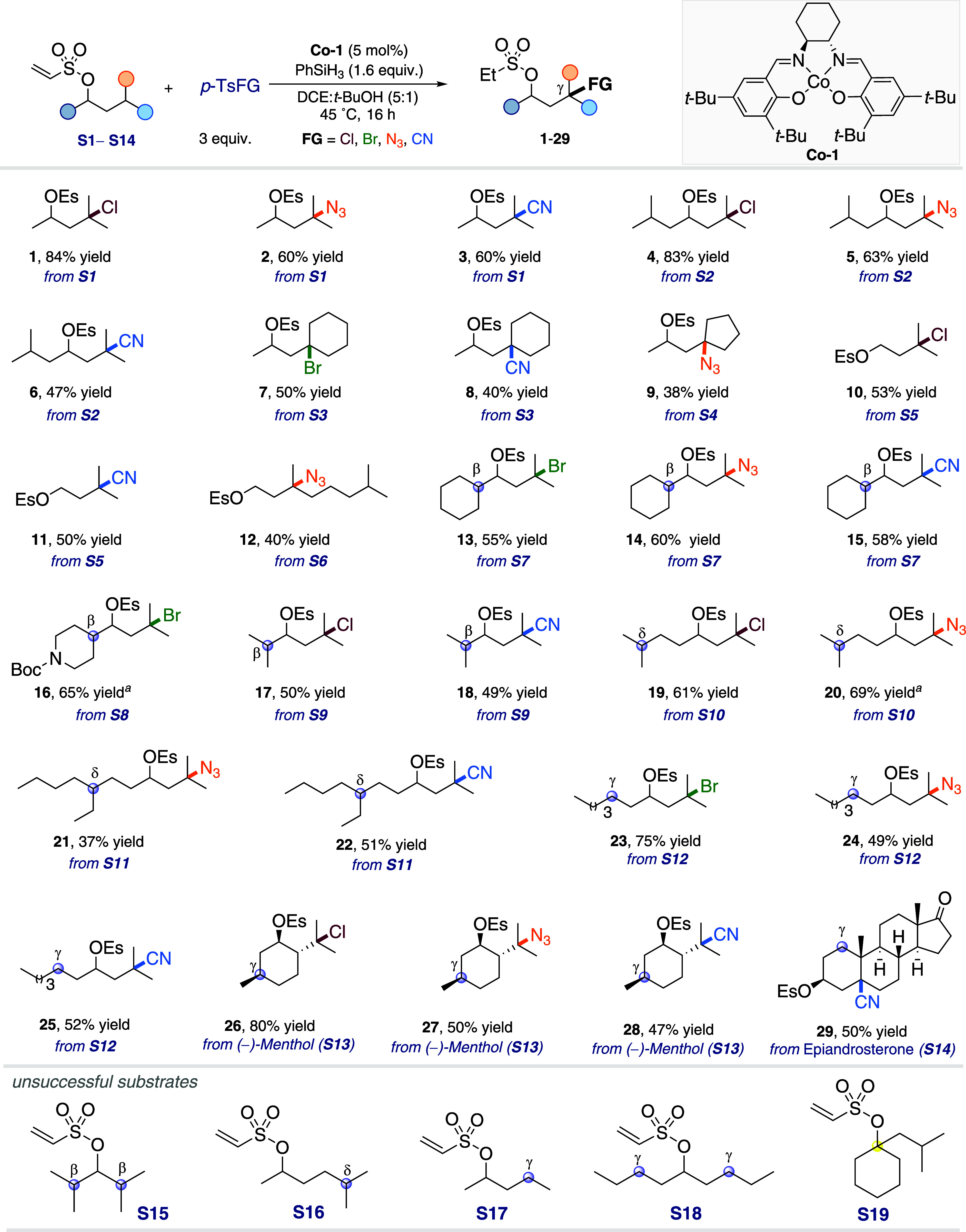
Scope of Cobalt Catalyzed γ-C­(sp^3^)–H Functionalization of Alcohols[Fn sch2-fn1]

a
^1^H NMR yields
b Standard conditions: Substrate (0.2 mmol), radical trapping reagent (*p*-Ts-FG) (3 equiv., 0.6 mmol), **Co-1** (0.01 mmol, 6 mg), PhSiH_3_ (0.32 mmol, 40 μL), DCE (2.5 mL), *t*-BuOH (0.5 mL), 45 °C, 16 h, isolated yields.

First, we focused on substrates **S1** and **S2**, which possess a single tertiary γ-C–H bond and lack potentially competing tertiary β- or δ-C–H sites. These substrates underwent smooth functionalization to provide the corresponding γ chlorinated, brominated, cyanated, and azidated secondary alcohols (**1**–**6**) in high yields and with excellent regioselectivity. The reaction tolerated significant steric hindrance at the target γ-C–H bond, as demonstrated by the successful functionalization of substrates **S3** and **S4**, albeit in moderate yields (**7**–**9**). Primary alcohol derivatives (**S5**, **S6**) were also viable substrates, furnishing products **10**–**12** in moderate yields. A key challenge in remote C–H functionalization is achieving high regioselectivity in substrates possessing multiple potentially reactive sites. We, therefore, investigated substrates that present such complexities. For alcohol derivatives **S7**–**S9**, which feature competing tertiary β and γ-C–H bonds, functionalization occurred exclusively at the γ–position via the desired 1,6-HAT pathway, yielding products **13**–**18** with excellent regioselectivity and good yields, demonstrating the strong preference for the 7-membered HAT transition state. Similarly, substrates **S10** and **S11**, containing both tertiary γ and δ C–H sites, reacted selectively at the γ position to afford products **19**–**22** (41–69% yield). Substrate **S12**, possessing both secondary and tertiary γ-C–H bonds, underwent selective functionalization at the more electron-rich tertiary site, delivering products **23**–**25** in good yields. To further showcase the utility of our method, we applied it to structurally complex natural product derivatives. Derivatives of (−)-menthol (**S13**), and an alkaloid (**S14**) were all successfully functionalized at their respective tertiary γ-C­(sp^3^)–H bonds, affording products **28**–**29** in good yields, highlighting the robustness and potential applicability of this strategy in complex molecular settings. The complete lack of reactivity observed for substrates **S15–S18** highlights the exclusive selectivity of the method for tertiary γ-C–H bonds (Scheme 2, below). In addition, a complete lack of reactivity was likewise observed for the tertiary alcohol derivative **S19**.

A scale-up of substrate **S2** (1.2 mmol) delivered γ-chlorinated product **4** in 80% yield, consistent with the small-scale reaction. Reductive cleavage of the vinyl sulfonyl tether with LiAlH_4_ furnished the free γ-chlorinated alcohol **4a** in 50% yield, demonstrating the scalability and synthetic utility of the method (Scheme 3).

**Scheme 3:**
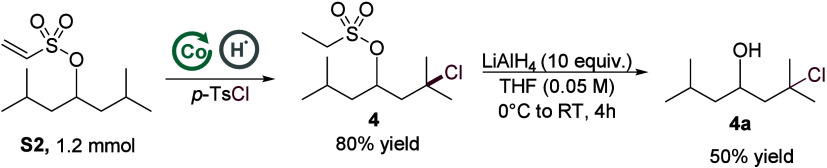
A Scale-Up Experiment and Esylate Group Deprotection

### Mechanistic Investigations

Afterward, we decided to investigate the reaction mechanism. First, we conducted deuterium labeling experiments for the chlorination of **S1**, using Ph_2_SiD_2_ instead of PhSiH_3_. The reaction showed complete deuterium incorporation at the terminal carbon of the tether (**1′**, 42% yield). In contrast, replacing *t*-butanol with *t*-BuOD resulted in no deuterium incorporation, but led to lower yields. These findings support the proposed MHAT-mediated radical relay mechanism (Scheme 4).

**Scheme 4:**
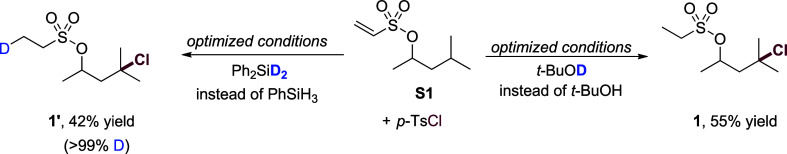
Deuterium Labeling Experiments

To further investigate the mechanism of the γ-functionalization reaction, EPR and UV–vis spectroscopy were used to monitor the cobalt species during the chlorination of substrate **S1** (Figure 1). Initially, the EPR spectrum of **Co-1** recorded at −173 °C displayed a characteristic signal indicative of a square planar [Co]^II^ species, an EPR-active species with g_1_ = 1.947, g_2_ = 1.933, and g_3_ = 3.213 (Figure 1a, black line).^57^ Upon the addition of tosyl chloride, the [Co]^II^ signal disappeared, replaced by an isotropic signal at g = 2.027, which accounted for only 5% of the initial intensity (Figure 1a, red line). This observation suggested that most of the [Co]^II^ was oxidized to an EPR-inactive [Co]^III^ species. The residual isotropic signal was attributed to a [Co]^II^–phenoxyl radical, indicating tautomerism between the two redox states, [Co^III^(phenolate)]^+^ and [Co^II^(phenoxyl•)]^+^.^58–60^ This results demonstrated the dual role of *p*-TsCl as oxidant and chlorinating reagent. The coordination environment of the [Co]^III^ species was further analyzed by UV–vis spectroscopy, revealing a broad absorption band around 860 nm (Figure 1b, red line), characteristic of an axial ligand-to-metal charge transfer in the newly formed [Co]^III^–Cl complex.^58^ Subsequent addition of **S1** to the mixture did not alter the cobalt coordination, apart from a decrease in the isotropic EPR signal, suggesting stabilization of the [Co]^III^ species (Figure 1a,b, blue line). However, the addition of silane led to a decrease in the broad absorption band at 880 nm, indicating the conversion of [Co]^III^–Cl to a new species, likely [Co]^III^–H (Figure 1b, green line). Upon heating the complete reaction mixture to 45 °C, a transient absorption band at 750 nm appeared but quickly disappeared, possibly corresponding to an intermediate [Co]^III^–alkyl species.^61^ Over prolonged reaction times, a *d*–*d* transition band emerged at 680 nm, suggesting the formation of decomposed cobalt species. Notably, the small residual EPR signal remained unchanged, indicating that [Co]^III^ species (i.e., [Co]^III^–Cl, [Co]^III^–H, and [Co]^III^–alkyl) predominated throughout the reaction (see SI).

**Figure 1:**
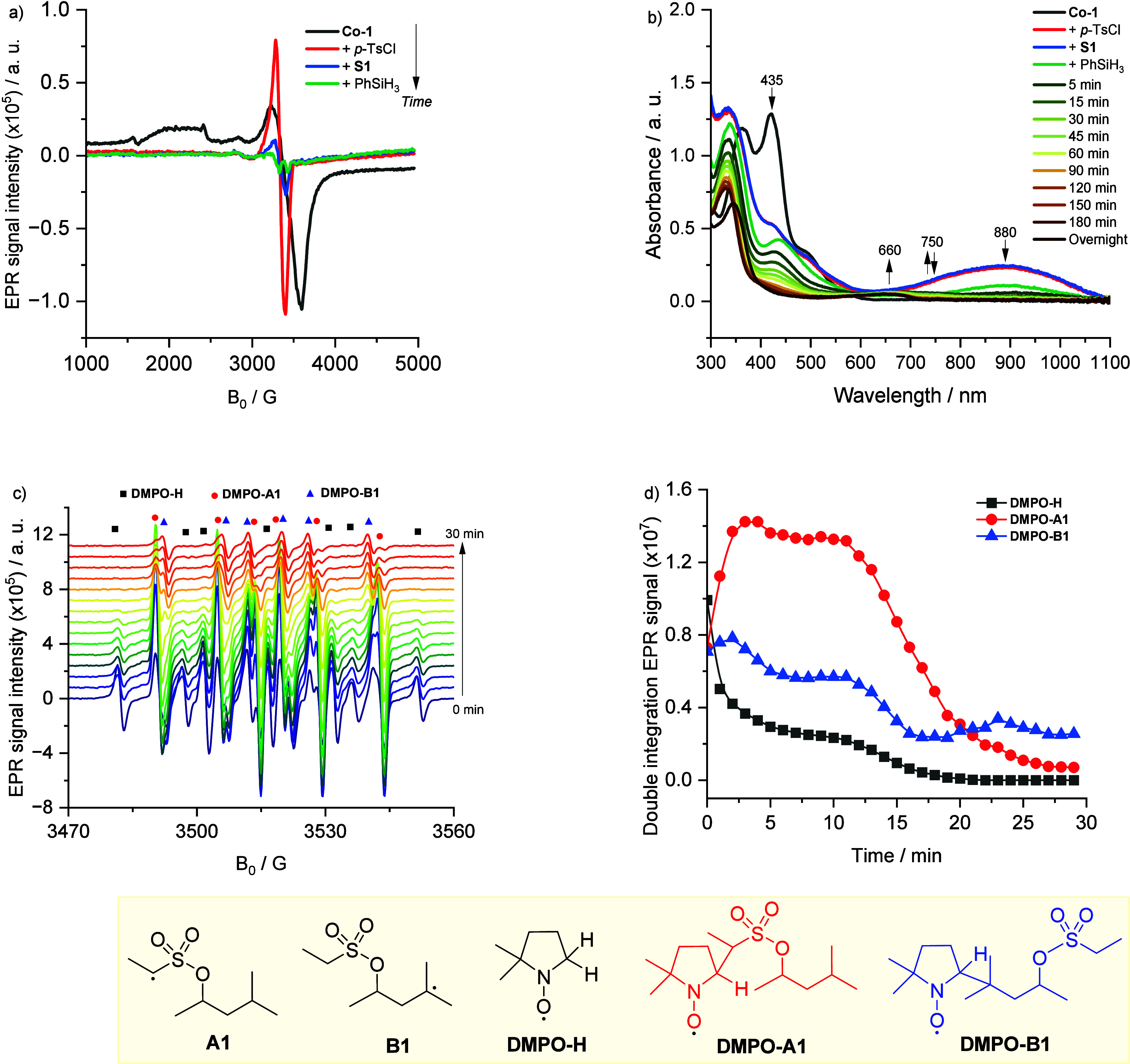
(a) EPR spectra recorded at −173 °C of **Co-1** upon different components addition. (b) UV–vis spectra of **Co-1** upon different components addition and the reaction mixture over time. (c) EPR spectra recorded at room temperature of the reaction mixture with DMPO overtime. (d) Reaction profile of the radical intermediates.

To characterize the radical species formed during the chlorination reaction, spin-trapping experiments were performed using 5,5-dimethyl-1-pyrroline *N*-oxide (DMPO) (Figure 1c). Three distinct radical adducts were detected by EPR: a hydrogen radical (a_N_ = 15 G, a_H1_ = a_H2_ = 19.8 G) and two different carbon-centered radical adducts (Adduct 1: a_N_ = 14.3 G, a_H_ = 23.2 G; Adduct 2: a_N_ = 14.3 G, a_H_ = 19.8 G). To aid in assigning these carbon-centered radicals, theoretical calculations were performed. Geometries were optimized at the BPW91/SVP level with PCM solvation (dichloromethane, ε = 8.93), and single-point hyperfine coupling constants were calculated at the MP2/TZVP//BPW91/SVP level of theory using Gaussian16 (further mini benchmark of methods can be found in SI). The computed hyperfine constants for the initial secondary alkyl radical formed on the tether (**DMPO-A1**) were a_N_ = 11.8 G and a_H_ = 25.2 G, while those for the translocated tertiary radical at the γ position (**DMPO-B1**) were a_N_ = 14.6 G and a_H_ = 16.0 G. This assignment provides direct experimental support for the proposed radical translocation pathway. The data extraction from Figure 1c led to the reaction profile shown in Figure 1d. The results revealed that the DMPO adduct of the initial radical (**DMPO-A1**) formed rapidly within the first 15 min before decreasing significantly, while the adduct of the more stable, translocated radical (**DMPO-B1**) persisted throughout the reaction, further corroborating the proposed mechanism.

To elucidate the reaction mechanism in greater detail, comprehensive density functional theory (DFT) calculations were performed. The complete reaction energy profile is depicted in Figure 2. Initially, we examined four possible pathways for MHAT from the *in situ* generated [Co]^III^–H species to the menthol derivative **S13** (Figure 2a). Our computational analysis revealed that the single-step HAT proceeds with a significantly lower free energy activation barrier (ΔG^‡^ = 19.6 kcal mol^–1^, TS_1_) as compared to alternative pathways. This process generates a [Co]^II^ metalloradical complex and a carbon-centered radical intermediate at the terminal carbon position with Markovnikov selectivity (**A13**), with the latter species having a relative free energy of –0.6 kcal mol^–1^ compared to the starting materials. In contrast, the two-step migratory insertion/homolysis mechanism exhibited a substantially higher activation barrier (ΔG^‡^ = 26.1 kcal mol^–1^, TS_2_), rendering this pathway kinetically less accessible under the reaction conditions. Further computational investigation of alternative MHAT processes demonstrated that both one- and two-steps anti-Markovnikov pathways are energetically prohibitive, with calculated free energy activation barriers of 39.9 (TS_3_) and 43.5 kcal mol^–1^ (TS_4_), respectively, thus explaining the observed regioselectivity in the initial HAT step. Following radical formation, we evaluated the energetics of three potential HAT pathways (Figure 2b). The 1,6-HAT process was determined to be kinetically preferred, proceeding with a relative free energy activation barrier of 13.3 kcal mol^–1^. The competing 1,5-HAT and 1,7-HAT pathways were calculated to be less favorable by 2.7 and 6.0 kcal mol^–1^, respectively. This preference can be attributed to a synergistic combination of enthalpic and entropic factors: enthalpically, the 1,6-HAT transition state experiences reduced ring strain compared to the 1,5-HAT analog; entropically, the 1,6-HAT pathway requires less molecular reorganization than the 1,7-HAT process. The 1,6-HAT culminates in forming a tertiary radical at the isopropyl group with a reaction free energy of –5.6 kcal mol^–1^ relative to the reactants. In addition, DFT calculations on the model radicals **A9** and **A10** further supports the kinetic preference of 1,6- vs. 1,5- and 1,7-HATs (Figure 2c). Finally, we investigated the radical trapping step involving the reaction of the translocated tertiary carbon radical with tosyl cyanide. The computational results indicate that cyanation proceeds via the addition of the carbon-centered radical to the electrophilic CN group with a free energy barrier of 20.7 kcal mol^–1^ (TS_5_). This step leads to the formation of a highly stabilized product (ΔG = –47.9 kcal mol^–1^ relative to reactants), providing a strong thermodynamic driving force for the overall transformation.

**Figure 2:**
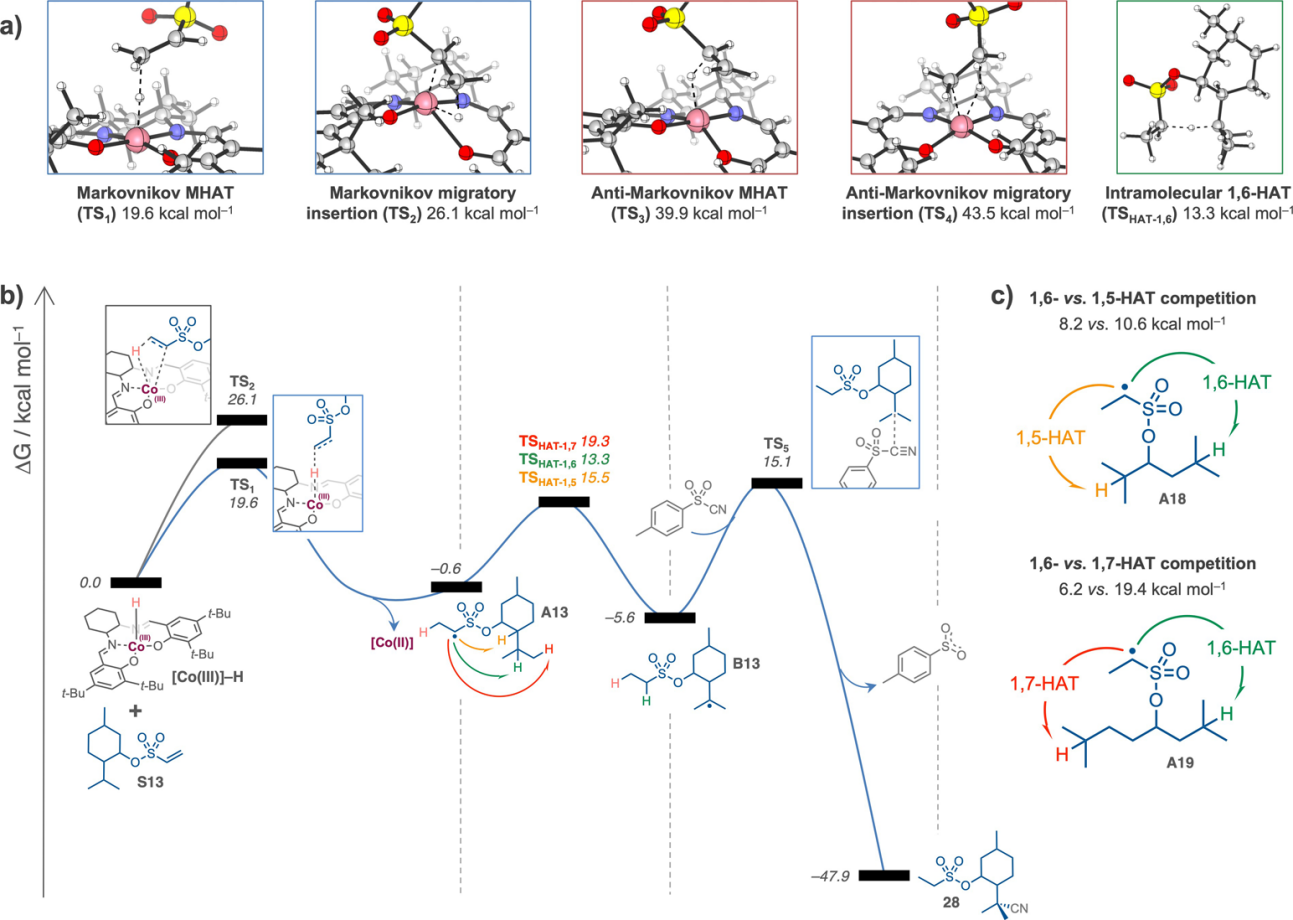
Computed reaction energy profile. Free energies, ΔG, are shown in kcal mol^–1^ at the BPW91/TZVP//BPW91/SVP level of theory in dichloromethane as a PCM solvent and 45 °C. See full computational details in the SI.

## Conclusion

In summary, we have developed a cobalt-catalyzed strategy for the remote γ-C­(sp^3^)–H functionalization of aliphatic alcohols via radical translocation. This method enables the selective introduction of diverse functional groups, including halides, cyanides, and azides, at tertiary γ-positions, which are traditionally challenging to access. Mechanistic studies, including EPR spectroscopy and DFT calculations, provide compelling evidence for a radical translocation pathway involving a 1,6-hydrogen atom transfer. The broad substrate scope, excellent regioselectivity, and applicability to complex molecular frameworks highlight the versatility and potential of this approach for late-stage functionalization and synthetic diversification.

## Experimental Content

All experimental details in this paper, such as synthetic procedure, characterization data, computational information, and NMR spectral data, are included in the Supporting Information.

## Supplementary Material



## Notes

Experimental data, characterization data for all new compounds, copies of NMR spectra and computational information (PDF)
