# Supplementary material for: Cobalt-Catalyzed γ‑C–H Functionalization of Alcohols via Olefin-Tethered Radical Relay
Source: JACS Au. 2025 Dec 11;6(1):95–102. doi: 10.1021/jacsau.5c00909 (PMC12848678; doi:10.1021/jacsau.5c00909)

Supporting Information

Cobalt-Catalyzed γ C–H Functionalization of Alcohols via Olefin Tethered Radical Relay

Phong Dam,[†] Kosala N. Amarasinghe,[†] Chenyang Wang,[†] Olga S. Bokareva,[†§] Luis Miguel Azofra,[‡] and Osama El-Sepelgy[†]*

[†]Leibniz Institute for Catalysis e.V., Albert-Einstein-Str. 29a, 18059 Rostock, Germany

[‡]Instituto de Estudios Ambientales y Recursos Naturales (i-UNAT), Universidad de Las Palmas de Gran Canaria (ULPGC), Campus de Tafira, 35017 Las Palmas de Gran Canaria, Spain

[§]Institute for Chemistry and Department of Life, Light & Matter, University of Rostock, Albert-Einstein-Str. 25 and 27, 18059 Rostock, Germany

E-Mail: Osama.Elsepelgy@Catalysis.de

Contents

I. General Information S2

II. General Procederes S3

II.1. General procedure for the synthesis of the tethered alcohols (GP1) S3

II.2. General procedure for the remote functionalization of alcohol derivatives (GP2) S4

II.3. Deprotection of ethyl sulfonyl group (GP3) S5

II.4. Additive robustness study S6

III. Characterization data S7

III.1. Tethered alcohol substrates S7

III.2. Final products S11

IV. EPR measurements S23

V. Computational information S24

V.1. Computational details S24

V.2. Reaction Profile Benchmarks Using Various Computational Methods S25

V.3. Theoretical Study of Radical Trapping in DMPO S26

V.4. Optimized Cartesian Coordinates S27

VI. References S52

VII. NMR spectra S53

I. General Information

Unless otherwise noted, all commercial reagents and solvents were purchased from commercial suppliers and used without further purification. The reactions were monitored by thin layer chromatography (TLC) with aluminum sheets silica gel 60 F254 from Merck, and flash column chromatography purifications were performed using silica gel 60 (63-200 μm) or aluminum oxide 90 neutral from MACHEREY-NAGEL. ^{1}H and ^{13}C NMR spectra were recorded with Bruker AV 300 (300 MHz), AV 400 (400 MHz) or Fourier 300 (300 MHz) NMR spectrometers. Chemical shifts (δ) are given relative to solvent: references for $CDCl_3$ were 7.26 ppm (^{1}H NMR) and 77.16 ppm (^{13}C NMR). And all signals were reported in parts per million (ppm) and spin-spin coupling constants (J) are given in Hz, while multiplicities are abbreviated by s (singlet), d (doublet), t (triplet), q (quartet), br (broad), m (multiplet). All measurements were carried out at room temperature unless otherwise stated. For the light-promoted reactions: Use of a blue-LEDs strip (λ max 467, manufacturer: Ledxon). The distance from the light source to the irradiation vessel was about 2 cm.

UV-vis spectra were recorded by a fiberoptical spectrometer with a probe consisting of a quartz fiber (AvaSpec-2048, Avantes) in a Schlenk vessel under argon after three times argon purging/evacuation to 10^{-3} mbar. The vessel was filled with 6 mL dry DCM and then each component was added stepwise to the solution. Spectra were recorded as a function of time at room temperature.

EPR measurements were recorded on a Bruker EMX CW-micro X-band spectrometer with a microwave power ≈ 6.9 mW, a modulation frequency of 100 kHz and modulation amplitude up to 5 G. The EPR spectrometer is equipped with a variable temperature control unit including a liquid N_2 cryostat and a temperature controller for recording the EPR spectra at low temperature down to 100 K. g values were calculated using the equation $h\nu = g\beta B_0$ with β, B_0 and ν being the Bohr magneton, resonance field and frequency, respectively. Calibration of the g values was performed using a DPPH standard (g = 2.0036 ± 0.0004). The simulated spectrum was acquired by using software package Easyspin.[1]

II. General Procederes

II.1. General procedure for the synthesis of the tethered alcohols (GP1)

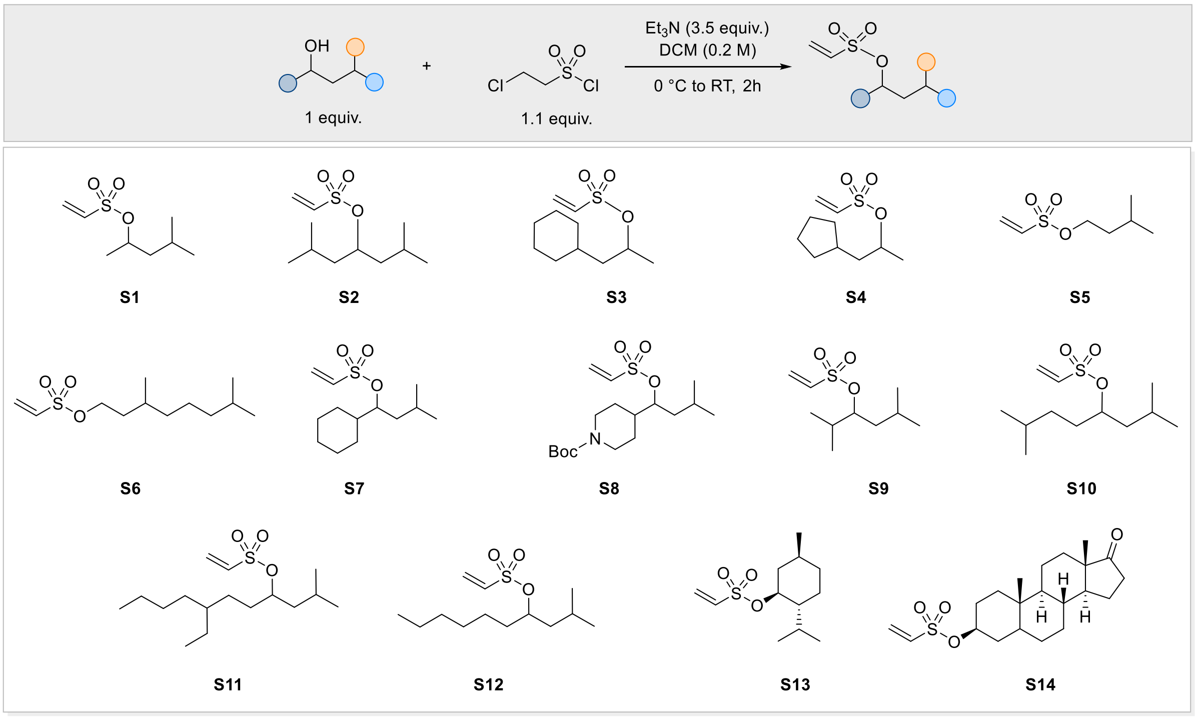

Scheme S1. *The substrate scope of the tethered alcohols.*

To a nitrogen-flushed round-bottom flask containing the desired alcohols (1.0 equiv.), dichloromethane (0.2 M with respect to the alcohol) and triethylamine (3.5 equiv.) were added. The reaction mixture was cooled to 0 °C, and 2-chloroethanesulfonyl chloride (1.1 equiv.) was added dropwise via syringe. After complete addition, the mixture was allowed to warm to room temperature and stirred for an additional 2 hours. The reaction was then diluted with dichloromethane and quenched with 1 M HCl. The organic layer was separated, washed with saturated brine, dried over $MgSO_4$, filtered, and concentrated under reduced pressure. The crude products were purified by flash column chromatography using silica gel.

II.2. General procedure for the remote functionalization of alcohol derivatives (GP2)

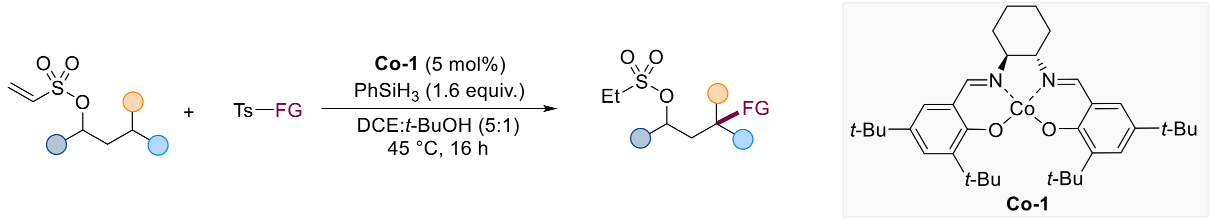

In an oven-dried 25 mL Schlenk tube equipped with a magnetic stir bar, vinyl sulfonyl-tethered alcohols (0.2 mmol), **Co-1** (5 mol%, 6 mg), and the radical trap (0.6 mmol, 3 equiv.) were added to 3 mL of a 5:1 (v/v) DCE:*t*BuOH mixture under an argon atmosphere. Phenylsilane (40 µL, 1.6 equiv.) was then added, and the reaction mixture was degassed via three freeze–pump–thaw cycles and backfilled with argon. The mixture was stirred at 45 °C for 16 hours. After the reaction was complete, the mixture was concentrated, and the crude residue was purified by column chromatography. The column was packed with *deactivated silica gel*, by flushing 1% triethylamine in hexane through the silica. Afterwards, normal hexane/ethyl acetate mixtures were used as the eluent to afford the desired products.

For yield determination by crude NMR, 1,3,5-trimethoxybenzene (0.2 mmol) was added as an internal standard. The reaction mixture was evaporated under reduced pressure to give a crude green oil, which was further filtered and washed through a short plug of neutral aluminum oxide using diethyl ether to remove paramagnetic cobalt species.

II.3. Deprotection of ethyl sulfonyl group (GP3)

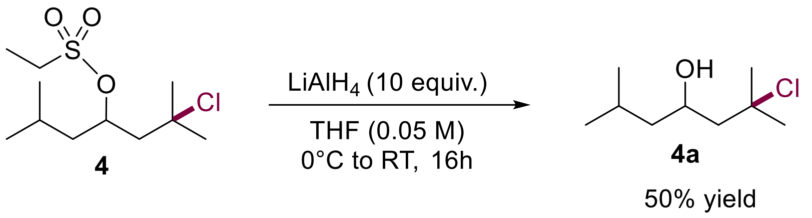

A 50 mL round-bottom flask equipped with a magnetic stirring bar was charged with compound **4** (0.2 mmol) and THF (5 mL, 0.05 M). The resulting solution was cooled to 0 °C in an ice-water bath. $LiAlH_4$ (75.9 mg, 2.0 mmol, 10 equiv) was added slowly under stirring. After complete addition, the reaction mixture was allowed to warm gradually to room temperature and stirred for 16 hours. Upon completion (as monitored by TLC), the reaction mixture was cooled again to 0 °C and quenched by the dropwise addition of methanol. The resulting mixture was filtered through a short pad of celite and was washed thoroughly with DCM. The combined filtrate was concentrated under reduced pressure, and the crude product was purified by flash column chromatography on silica gel using hexane/ethyl acetate (2:1, v/v) as the eluent to afford the desired compound **4a** in 50% isolated yield.

II.4. Additive robustness study

An additive robustness study was conducted on the reaction for the chlorination of the alcohol substrate **S1** to assess the tolerance of various reactive functional groups. Representative examples demonstrate that the remote functionalization process uniquely accommodates the presence of alcohols aryl bromides, aldehydes, aliphatic ketones and alcohols, epoxides with minimal impact on the yield. However, we have observed a yield drop in the presence of aromatic amine.

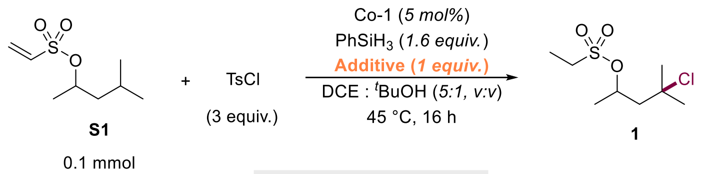

Functional group tolerance

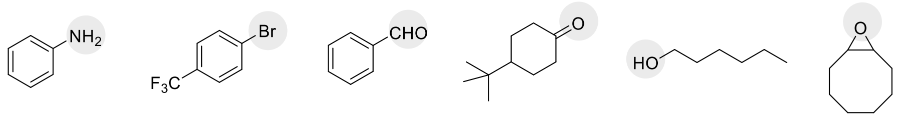

Yield	23%	64%	85%	74%	72%	81%
Additive remaining	60%	13%	23%	76%	20%	84%

III. CHARACTERIZATION DATA

III.1. Tethered alcohol substrates

4-methylpentan-2-yl ethenesulfonate (**S1**)

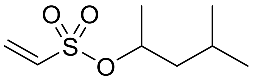

S1 was prepared according to **GP1** from 4-methylpentan-2-ol .

Purification by chromatography (pentane/diethyl ether = 5:1).

Yield: 67 % (colorless oil).

^{1}H NMR (300 MHz, CDCl$_3$) δ = 6.55 (dd, *J*=16.6, 9.8, 1H), 6.38 (d, *J*=16.7, 1H), 6.05 (d, *J*=9.7, 1H), 4.82 – 4.65 (m, 1H), 1.80 – 1.60 (m, 2H), 1.39 (d, *J*=6.2, 3H), 1.37 – 1.29 (m, 1H), 0.91 (t, *J*=6.2, 6H).

^{13}C NMR (75 MHz, CDCl$_3$) δ 134.1, 128.9, 79.9, 45.9, 24.5, 22.8, 22.3, 21.7.

2,6-dimethylheptan-4-yl ethenesulfonate (**S2**)

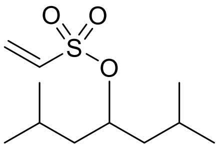

S2 was prepared according to **GP1** from 2,6-dimethylheptan-4-ol.

Purification by chromatography (pentane/diethyl ether = 5:1)

Yield: 57 % (colorless oil)

^{1}H NMR (300 MHz, CDCl$_3$) δ = 6.56 (dd, *J*=16.6, 9.8, 1H), 6.37 (d, *J*=16.6, 1H), 6.03 (d, *J*=9.8, 1H), 4.77 – 4.69 (m, 1H), 1.74 – 1.60 (m, 4H), 1.48 – 1.39 (m, 2H), 0.92 (t, *J*=6.2, 12H).

^{13}C NMR (75 MHz, CDCl$_3$) δ 134.3, 128.6, 82.6, 44.2, 24.5, 22.9, 22.4.

1-cyclohexylpropan-2-yl ethenesulfonate (**S3**)

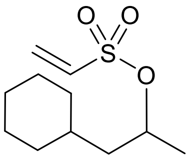

S3 was prepared according to **GP1** with 1-cyclohexylpropan-2-ol.

Purification by chromatography (pentane/diethyl ether = 10:1)

Yield: 38 % (colorless oil)

^{1}H NMR (300 MHz, CDCl$_3$) δ = 6.55 (dd, *J*=16.6, 9.8, 1H), 6.38 (d, *J*=16.7, 1H), 6.05 (d, *J*=9.8, 1H), 4.83 – 4.71 (m, 1H), 1.83 – 1.59 (m, 6H), 1.39 (d, *J*=6.2, 4H), 1.32 – 1.06 (m, 4H), 1.01 – 0.79 (m, 2H).

^{13}C NMR (75 MHz, CDCl$_3$) δ 134.1, 128.9, 79.5, 44.5, 33.9, 33.5, 33.0, 26.5, 26.3, 26.1, 21.8.

1-cyclopentylpropan-2-yl ethenesulfonate (S4)

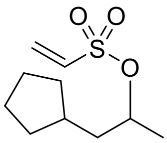

S4 was prepared according to **GP1** with 1-cyclopentylpropan-2-ol.

Purification by chromatography (pentane/diethyl ether = 7:1).

Yield: 46 % (colorless oil).

^{1}H NMR (300 MHz, CDCl$_3$) δ = 6.56 (dd, *J*=16.6, 9.8, 1H), 6.39 (d, *J*=16.7, 1H), 6.06 (d, *J*=9.8, 1H), 4.74 – 4.63 (m, 1H), 1.93 – 1.74 (m, 4H), 1.68 – 1.48 (m, 6H), 1.40 (d, *J*=6.2, 3H), 1.16 – 1.01 (m, 2H).

^{13}C NMR (75 MHz, CDCl$_3$) δ 134.1, 128.9, 81.1, 43.2, 36.3, 32.8, 32.8, 25.2, 25.1, 21.6.

isopentyl ethenesulfonate (S5)

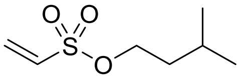

S5 was prepared according to **GP1** with isoamyl alcohol.

Purification by chromatography (pentane/diethyl ether = 10:1)

Yield: 80% (colorless oil)

^{1}H NMR (300 MHz, CDCl$_3$) δ = 6.54 (dd, *J*=16.6, 9.5, 1H), 6.41 (d, *J*=16.7, 1H), 6.12 (d, *J*=9.5, 1H), 4.16 (t, *J*=6.6, 2H), 1.82 – 1.69 (m, 1H), 1.65 – 1.58 (m, 2H), 0.93 (d, *J*=6.5, 6H).

^{13}C NMR (75 MHz, CDCl$_3$) δ 132.7, 130.1, 69.7, 37.7, 24.7, 22.4.

3,7-dimethyloctyl ethenesulfonate (S6)

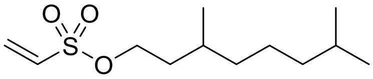

S6 was prepared according to **GP1** with 3,7-dimethyl-1-octanol.

Purification by chromatography (pentane/diethyl ether = 5:1).

Yield: 51 % (colorless oil).

^{1}H NMR (300 MHz, CDCl$_3$) δ = 6.53 (dd, *J*=16.6, 9.6, 1H), 6.39 (d, *J*=16.7, 1H), 6.11 (d, *J*=9.5, 1H), 4.18 – 4.12 (m, 2H), 1.80 – 1.70 (m, 1H), 1.58 – 1.44 (m, 3H), 1.30 – 1.09 (m, 7H), 0.89 (d, *J*=6.4, 3H), 0.85 (d, *J*=6.5, 6H).

^{13}C NMR (75 MHz, CDCl$_3$) δ 132.7, 130.1, 69.6, 39.2, 37.1, 36.0, 29.4, 28.0, 24.6, 22.8, 22.7, 19.3.

1-cyclohexyl-3-methylbutyl ethenesulfonate (S7)

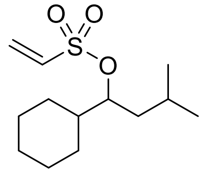

S7 was prepared according to **GP1** with 1-cyclohexyl-3-methylbutan-1-ol.

Purification by chromatography (pentane/diethyl ether = 5:1)

Yield: 57 % (colorless oil)

^{1}H NMR (300 MHz, CDCl$_3$) δ = 6.57 (dd, *J*=16.6, 9.9, 1H), 6.36 (d, *J*=16.7, 1H), 6.02 (d, *J*=9.8, 1H), 4.59 – 4.53 (m, 1H), 1.82 – 1.58 (m, 9H), 1.43 – 1.36 (m, 1H), 1.27 – 1.04 (m, 5H), 0.92 (dd, *J*=6.6, 1.3, 6H).

^{13}C NMR (75 MHz, CDCl$_3$) δ 134.2, 128.3, 87.8, 41.9, 40.2, 28.5, 27.9, 26.4, 26.2, 26.2, 24.4, 23.3, 22.0.

tert-butyl 4-(3-methyl-1-((vinylsulfonyl)oxy)butyl)piperidine-1-carboxylate (S8)

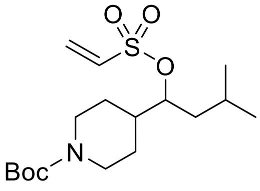

S8 was prepared according to **GP1** with tert-butyl 4-(1-hydroxy-3-methylbutyl)piperidine-1-carboxylate.

Purification by chromatography (pentane/diethyl ether = 4:1)

Yield: 60 % (colorless oil)

^{1}H NMR (300 MHz, CDCl$_3$) δ = 6.54 (dd, *J*=16.6, 9.8, 1H), 6.33 (d, *J*=16.7, 1H), 6.01 (d, *J*=9.8, 1H), 4.56 (dt, *J*=8.5, 4.3, 1H), 4.13 (br, 2H), 2.60 (br, 2H), 1.85 – 1.74 (m, 1H), 1.66 – 1.55 (m, 4H), 1.41 (s, 9H), 1.24 – 1.21 (m, 3H), 0.89 (d, *J*=2.4, 3H), 0.87 (d, *J*=2.5, 3H).

^{13}C NMR (75 MHz, CDCl$_3$) δ 154.7, 133.9, 128.7, 86.0, 79.5, 40.5, 40.2, 31.6, 28.5, 27.6, 24.2, 23.2, 22.7, 21.9, 14.2.

2,5-dimethylhexan-3-yl ethenesulfonate (**S9**)

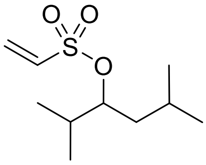

S9 was prepared according to **GP1** with 2,5-dimethylhexan-3-ol.

No need to further purify by column chromatography.

Yield: 65 %

^{1}H NMR (300 MHz, CDCl$_3$) δ = 6.60 (dd, *J*=16.6, 9.8, 1H), 6.39 (d, *J*=16.7, 1H), 6.05 (d, *J*=9.8, 1H), 4.66 – 4.58 (m, 1H), 2.14 – 1.99 (m, 1H), 1.81 – 1.68 (m, 1H), 1.68 – 1.60 (m, 1H), 1.40 – 1.33 (m, 1H), 1.00 (d, *J*=6.9, 3H), 0.97 – 0.93 (m, 9H).

^{13}C NMR (75 MHz, CDCl$_3$) δ 134.2, 128.4, 88.0, 39.6, 31.9, 24.3, 23.3, 22.0, 17.8, 17.4.

2,7-dimethyloctan-4-yl ethenesulfonate (**S10**)

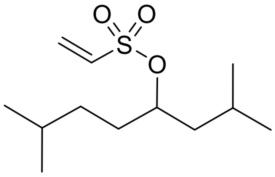

S10 was prepared according to **GP1** with 2,7-dimethyloctan-4-ol.

Purification by chromatography (pentane/diethyl ether = 10:1).

Yield: 20 %

^{1}H NMR (300 MHz, CDCl$_3$) δ = 6.56 (dd, *J*=16.7, 9.8, 1H), 6.37 (d, *J*=16.6, 1H), 6.04 (d, *J*=9.8, 1H), 4.71 – 4.61 (m, 1H), 1.74 – 1.60 (m, 4H), 1.56 – 1.37 (m, 2H), 1.29 – 1.20 (m, 2H), 0.93 (d, *J*=3.1, 3H), 0.91 (s, 3H), 0.90 (d, *J*=2.8, 3H), 0.88 (d, *J*=2.7, 3H).

^{13}C NMR (75 MHz, CDCl$_3$) δ 134.2, 128.6, 84.1, 43.6, 33.8, 32.9, 28.0, 24.5, 23.0, 22.6, 22.6, 22.3.

7-ethyl-2-methylundecan-4-yl ethenesulfonate (**S11**)

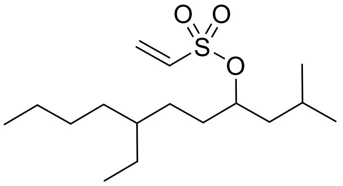

S11 was prepared according to **GP1** with 7-ethyl-2-methyl undecane-4-ol.

Purification by chromatography (pentane/diethyl ether = 10:1).

Yield: 55 % (colorless oil)

^{1}H NMR (300 MHz, CDCl$_3$) δ = 6.56 (dd, *J*=16.7, 9.8, 1H), 6.38 (d, *J*=16.7, 1H), 6.03 (d, *J*=9.8, 1H), 4.70 – 4.61 (m, 1H), 1.76 – 1.61 (m, 4H), 1.46 – 1.38 (m, 1H), 1.37 – 1.17 (m, 12H), 0.94 (d, *J*=3.2, 3H), 0.92 (d, *J*=3.0, 3H), 0.88 (d, *J*=6.8, 3H), 0.84 (td, *J*=7.1, 1.1, 3H).

^{13}C NMR (75 MHz, CDCl$_3$) δ 134.2, 128.6, 84.3, 43.6, 38.8, 38.7, 32.8, 32.2, 29.0, 28.1, 25.8, 24.5, 23.2, 23.1, 22.3, 14.3, 10.9.

2-methyldecan-4-yl ethenesulfonate (**S12**)

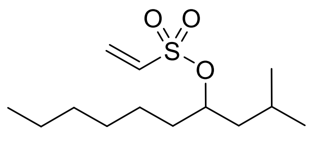

S12 was prepared according to **GP1** with 2-methyldecan-4-ol.

Purification by chromatography (pentane/diethyl ether = 10:1).

Yield: 32 % (colorless oil)

^{1}H NMR (300 MHz, CDCl$_3$) δ = 6.55 (dd, *J*=16.6, 9.8, 1H), 6.35 (d, *J*=16.7, 1H), 6.02 (d, *J*=9.8, 1H), 4.69 – 4.61 (m, 1H), 1.76 – 1.59 (m, 5H), 1.48 – 1.17 (m, 11H), 0.94 – 0.85 (m, 9H).

^{13}C NMR (75 MHz, CDCl$_3$) δ 134.1, 128.6, 83.8, 43.5, 35.0, 31.7, 29.1, 24.8, 24.4, 23.0, 22.6, 22.3, 14.1.

(1S,2R,5S)-2-isopropyl-5-methylcyclohexyl ethenesulfonate (**S13**)

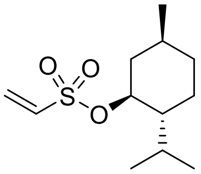

S13 was prepared according to **GP1** with (1R,2S,5R)-(−)-Menthol.

Purification by chromatography (pentane/diethyl ether = 5:1).

Yield: 66 % (colorless oil).

^{1}H NMR (300 MHz, CDCl$_3$) δ = 6.57 (dd, *J*=16.6, 9.8, 1H), 6.39 (d, *J*=16.7, 1H), 6.03 (d, *J*=9.8, 1H), 4.48 – 4.40 (m, 1H), 2.28 – 2.19 (m, 1H), 2.13 – 1.988 (m, 1H), 1.76 – 1.63 (m, 2H), 1.52 – 1.36 (m, 2H), 1.32 – 1.19 (m, 1H), 1.11 – 0.95 (m, 1H), 0.94 (d, *J*=3.2, 3H), 0.91 (d, *J*=3.8, 3H), 0.80 (d, *J*=6.9, 3H).

^{13}C NMR (75 MHz, CDCl$_3$) δ 134.3, 128.6, 84.5, 47.6, 42.3, 33.9, 31.8, 25.7, 23.2, 22.0, 21.0, 15.8.

(3S,8R,9S,10S,13S,14S)-10,13-dimethyl-17-oxohexadecahydro-1H-cyclopenta[a]phenanthren-3-yl ethenesulfonate (S14)

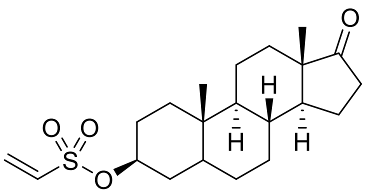

S14 was prepared according to **GP1** with Prasterone.

Purification by chromatography (pentane/diethyl ether = 5:1)

Yield: 16% (white solid).

^{1}H NMR (300 MHz, CDCl$_3$) δ = 6.56 (dd, *J*=16.6, 9.8, 1H), 6.39 (d, *J*=16.7, 1H), 6.06 (d, *J*=9.7, 1H), 4.53 – 4.41 (m, 1H), 2.49 – 2.39 (dd, *J*=19.0, 8.9, 1H), 2.05 (dd, *J*=19.1, 9.0, 1H), 1.99 – 1.87 (m, 2H), 1.85 – 1.70 (m, 5H), 1.69 – 1.56 (m, 3H), 1.54 – 1.45 (m, 2H), 1.37 – 1.16 (m, 6H), 1.08 – 0.91 (m, 2H), 0.85 (d, *J*=2.8, 6H).

^{13}C NMR (75 MHz, CDCl$_3$) δ 221.2, 134.0, 129.0, 82.8, 54.3, 51.5, 45.0, 36.9, 36.0, 35.6, 35.1, 31.6, 30.9, 28.7, 28.3, 21.9, 20.6, 14.0, 12.3.

III.2. Final products

4-chloro-4-methylpentan-2-yl ethanesulfonate (1)

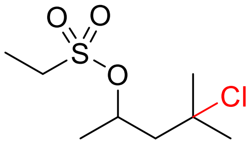

The chlorination reaction was conducted according to **GP2** with compound **S1** (38.4 mg, 0.2 mmol) and *p*-toluenesulfonyl chloride (116.6 mg, 0.6 mmol) as radical trap.

Isolated yield: 84% (38 mg, 0.16 mmol).

Eluent composition: gradient elution from pure *n*-hexane to *n*-hexane/ethyl acetate (v/v 10:1).

^{1}H NMR (300 MHz, $CDCl_3$) δ = 5.18 – 5.08 (m, 1H), 3.12 (q, *J*=7.5, 2H), 2.26 (dd, *J*=15.4, 6.8, 1H), 2.03 (dd, *J*=15.4, 3.9, 1H), 1.65 (d, *J*=8.6, 6H), 1.52 (d, *J*=6.3, 3H), 1.43 (t, *J*=7.4, 3H).

^{13}C NMR (75 MHz, $CDCl_3$) δ 76.7, 68.2, 52.2, 46.6, 33.9, 32.1, 23.3, 8.3.

HRMS (ESI) m/z: $[M + Na]^+$ Calcd for $C_8H_{17}O_3SClNa$ 251.0485; Found 251.0488.

4-azido-4-methylpentan-2-yl ethanesulfonate (2)

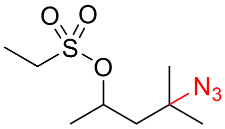

The azidation reaction was conducted according to **GP2** with compound **S1** (38.4 mg, 0.2 mmol) and *p*-toluenesulfonyl azide (118.3 mg, 0.6 mmol) as radical trap.

Isolated yield: 60% (28 mg, 0.12 mmol).

Eluent composition: gradient elution from pure *n*-hexane to *n*-hexane/ethyl acetate (v/v 5:1).

^{1}H NMR (300 MHz, $CDCl_3$) δ = 5.06 – 4.96 (m, 1H), 3.12 (q, *J*=7.4, 2H), 2.00 (dd, *J*=15.1, 7.0, 1H), 1.70 (dd, *J*=15.0, 4.4, 1H), 1.49 (d, *J*=6.3, 3H), 1.43 (t, *J*=7.4, 3H), 1.36 (d, *J*=5.4, 6H).

^{13}C NMR (75 MHz, $CDCl_3$) δ 75.9, 60.1, 47.7, 46.5, 27.1, 26.0, 23.0, 8.3.

HRMS (ESI) m/z: $[M + Na]^+$ Calcd for $C_8H_{17}N_3O_3SNa$ 258.0888; Found 258.0892.

4-cyano-4-methylpentan-2-yl ethanesulfonate (3)

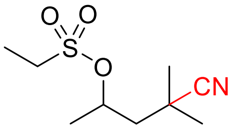

The cyanation reaction was conducted according to **GP2** with compound **S1** (38.4 mg, 0.2 mmol) and *p*-toluenesulfonyl cyanide (114 mg, 0.6 mmol) as radical trap.

Isolated yield: 60% (26 mg, 0.12 mmol).

Eluent composition: gradient elution from pure *n*-hexane to *n*-hexane/ethyl acetate (v/v 1:1).

^{1}H NMR (300 MHz, $CDCl_3$) δ = 5.11 – 4.99 (m, 1H), 3.22 – 3.11 (m, 2H), 2.10 (dd, *J*=14.9, 8.9, 1H), 1.67 (dd, *J*=14.9, 3.8, 1H), 1.53 (d, *J*=6.2, 3H), 1.44 (t, *J*=7.5, 3H), 1.43 (s, 6H).

^{13}C NMR (75 MHz, $CDCl_3$) δ 124.6, 74.3, 46.9, 46.3, 30.1, 28.0, 26.3, 22.5, 8.1.

HRMS (ESI) m/z: $[M + Na]^+$ Calcd for $C_9H_{17}NO_3SNa$ 242.0821; Found 242.0827.

2-chloro-2,6-dimethylheptan-4-yl ethanesulfonate (4)

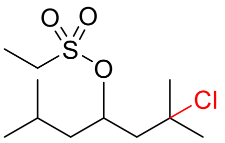

The chlorination reaction was conducted according to **GP2** with compound **S2** (46.8 mg, 0.2 mmol) and *p*-toluenesulfonyl chloride (116.6 mg, 0.6 mmol) as radical trap.

Isolated yield: 83% (44 mg, 0.17 mmol).

Eluent composition: gradient elution from pure *n*-hexane to *n*-hexane/ethyl acetate (v/v 10:1).

^{1}H NMR (300 MHz, $CDCl_3$) δ = 5.11 – 5.03 (m, 1H), 3.12 (q, *J*=7.5, 2H), 2.22 (dd, *J*=15.5, 5.8, 1H), 2.14 (dd, *J*=15.4, 4.5, 1H), 1.81 – 1.71 (m, 3H), 1.66 (d, *J*=10.9, 6H), 1.43 (t, *J*=7.4, 3H), 0.98 (dd, *J*=6.4, 3.2, 6H).

^{13}C NMR (75 MHz, $CDCl_3$) δ 79.0, 68.4, 50.4, 46.8, 45.6, 34.1, 32.1, 24.8, 22.7, 22.6, 8.3.

HRMS (ESI) m/z: $[M + Na]^+$ Calcd for $C_{11}H_3O_3SClNa$ 293.0954; Found 293.0959.

2-azido-2,6-dimethylheptan-4-yl ethanesulfonate (5)

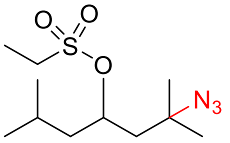

The azidation reaction was conducted according to **GP2** with compound **S2** (46.8 mg, 0.2 mmol) and *p*-toluenesulfonyl azide (118.3 mg, 0.6 mmol) as radical trap.

Isolated yield: 63% (35 mg, 0.12 mmol).

Eluent composition: gradient elution from pure *n*-hexane to *n*-hexane/ethyl acetate (v/v 5:1).

^{1}H NMR (300 MHz, $CDCl_3$) δ = 4.98 – 4.91 (m, 1H), 3.17 – 3.07 (m, 2H), 1.96 (dd, *J*=15.1, 5.9, 1H), 1.79 (dd, J=15.1, 5.1, 1H), 1.73 – 1.57 (m, 3H), 1.43 (t, *J*=7.5, 3H), 1.36 (d, *J*=6.3, 6H), 0.96 (dd, *J*=6.4, 4.8, 6H).

^{13}C NMR (75 MHz, $CDCl_3$) δ 78.3, 60.1, 46.6, 45.9, 45.3, 27.2, 26.0, 24.8, 22.7, 22.5, 8.3.

HRMS (ESI) m/z: $[M + Na]^+$ Calcd for $C_{11}H_{23}N_3O_3SNa$ 300.1358; Found 300.1359.

2-cyano-2,6-dimethylheptan-4-yl ethanesulfonate (6)

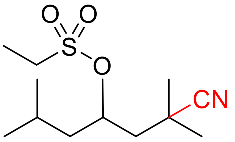

The cyanation reaction was conducted according to **GP2** with compound **S2** (46.8 mg, 0.2 mmol) and *p*-toluenesulfonyl cyanide (114 mg, 0.6 mmol) as radical trap.

Isolated yield: 47% (25 mg, 0.09 mmol).

Eluent composition: gradient elution from pure *n*-hexane to *n*-hexane/ethyl acetate (v/v 10:1).

^{1}H NMR (300 MHz, CDCl$_3$) δ = 5.03 – 4.94 (m, 1H), 3.31 – 3.10 (m, 2H), 2.05 (dd, *J*=14.9, 7.7, 1H), 1.86 – 1.63 (m, 4H), 1.44 (d, *J*=2.6, 6H), 1.44 (t, *J*=7.4, 3H), 0.99 – 0.94 (m, 6H).

^{13}C NMR (75 MHz, CDCl$_3$) δ 124.8, 76.8, 46.4, 45.1, 44.7, 30.0, 27.8, 26.7, 24.8, 22.7, 22.5, 8.1.

HRMS (ESI) m/z: $[M + Na]^+$ Calcd for $C_{12}H_{23}NO_3SNa$ 284.1291; Found 284.1295.

1-(1-bromocyclohexyl)propan-2-yl ethanesulfonate (7)

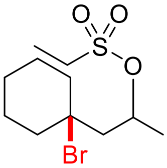

The bromination reaction was conducted according to **GP2** with compound **S3** (52.8 mg, 0.2 mmol) and *p*-toluenesulfonyl bromide (141 mg, 0.6 mmol) as radical trap.

Isolated yield: 50% (31 mg, 0.1 mmol)

Eluent composition: gradient elution from pure *n*-hexane to *n*-hexane/ethyl acetate (v/v 10:1).

^{1}H NMR (400 MHz, CDCl$_3$) δ = 5.32 – 5.25 (m, 1H), 3.16 – 3.09 (m, 2H), 2.38 (dd, *J*=15.7, 6.4, 1H), 2.24 – 2.13 (m, 2H), 2.08 – 2.00 (m, 1H), 1.85 – 1.71 (m, 2H), 1.71 – 1.51 (m, 6H), 1.55 (d, *J*=6.3, 3H), 1.43 (t, *J*=7.5, 3H).

^{13}C NMR (101 MHz, CDCl$_3$) δ 77.6, 73.2, 53.2, 46.6, 42.1, 40.3, 25.2, 23.6, 23.1, 23.1, 8.3.

HRMS (ESI) m/z: $[M + Na]^+$ Calcd for $C_{11}H_{21}[^{81}Br]O_3SNa$ 335.0395; Found 335.0398.

1-(1-cyanocyclohexyl)propan-2-yl ethanesulfonate (8)

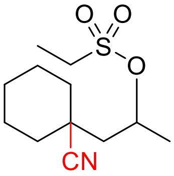

The cyanation reaction was conducted according to **GP2** with compound **S3** (46.4 mg, 0.2 mmol) and *p*-toluenesulfonyl cyanide (114 mg, 0.6 mmol) as radical trap.

Isolated yield: 40% (21 mg, 0.08 mmol).

Eluent composition: gradient elution from pure *n*-hexane to *n*-hexane/ethyl acetate (v/v 10:1).

^{1}H NMR (300 MHz, CDCl$_3$) δ = 5.14 – 5.04 (m, 1H), 3.29 – 3.11 (m, 2H), 2.14 – 1.99 (m, 3H), 1.81 – 1.57 (m, 7H), 1.54 (d, *J*=6.2, 3H), 1.44 (t, *J*=7.4, 3H), 1.32 – 1.24 (m, 2H).

^{13}C NMR (75 MHz, CDCl$_3$) δ 108.3, 74.0, 46.8, 46.3, 36.5, 36.4, 35.2, 25.3, 22.8, 22.7, 8.1.

HRMS (ESI) m/z: $[M + Na]^+$ Calcd for $C_{12}H_{21}NO_3SNa$ 282.1134; Found 282.1131.

1-(1-azidocyclopentyl)propan-2-yl ethanesulfonate (9)

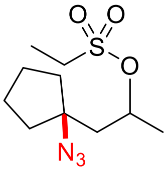

The azidation reaction was conducted according to **GP2** with compound **S4** (43.7 mg, 0.2 mmol) and p-toluenesulfonyl azide (118.3 mg, 0.6 mmol) as radical trap.

Isolated yield: 38% (20 mg, 0.07 mmol).

Eluent composition: gradient elution from pure *n*-hexane to *n*-hexane/ethyl acetate (v/v 10:1).

^{1}H NMR (300 MHz, CDCl$_3$) δ = 5.08 – 4.97 (m, 1H), 3.13 (q, *J*=7.5, 2H), 2.11 (dd, *J*=15.0, 6.9, 1H), 1.98 – 1.87 (m, 3H), 1.82 – 1.59 (m, 6H), 1.51 (d, *J*=6.2, 3H), 1.43 (t, *J*=7.4, 3H).

^{13}C NMR (75 MHz, CDCl$_3$) δ 76.8, 71.4, 46.4, 45.5, 38.2, 37.1, 23.8, 23.3, 22.8, 8.3.

HRMS (ESI) m/z: [M + Na]$^+$ Calcd for $C_{10}H_{19}N_3O_3SNa$ 284.1045; Found 284.1050.

3-chloro-3-methylbutyl ethanesulfonate (10)

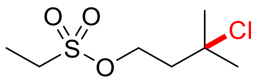

The chlorination reaction was conducted with compound **S5** (35.6 mg, 0.2 mmol) and *p*-toluenesulfonyl chloride (116.6 mg, 0.6 mmol) as radical trap according to **GP2**.

Isolated yield: 53% (23 mg, 0.11 mmol).

Eluent composition: gradient elution from pure *n*-hexane to *n*-hexane/ethyl acetate (v/v 8:1).

^{1}H NMR (300 MHz, CDCl$_3$) δ = 4.46 (t, *J*=6.8, 2H), 3.14 (q, *J*=7.4, 2H), 2.22 (t, *J*=6.8, 2H), 1.63 (s, 6H), 1.43 (t, *J*=7.4, 3H).

^{13}C NMR (75 MHz, CDCl$_3$) δ 67.9, 66.7, 45.1, 44.6, 33.0, 8.3.

LRMS (ESI) m/z: [M+ Na]$^+$ Calcd for $C_7H_{15}O_3ClSNa$ 237, Found 237.

3-cyano-3-methylbutyl ethanesulfonate (11)

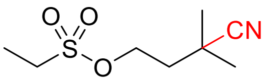

The cyanation reaction was conducted according to **GP2** with compound **S5** (35.6 mg, 0.2 mmol) and *p*-toluenesulfonyl cyanide (114 mg, 0.6 mmol) as radical trap.

Isolated yield: 50% (20 mg, 0.1 mmol).

Eluent composition: gradient elution from pure *n*-hexane to *n*-hexane/ethyl acetate (v/v 2:1).

^{1}H NMR (300 MHz, CDCl$_3$) δ = 4.41 (t, *J*=6.6, 2H), 3.18 (q, *J*=7.5, 2H), 2.02 (t, *J*=6.6, 2H), 1.44 (t, *J*=7.4, 3H), 1.43 (s, 6H).

^{13}C NMR (75 MHz, CDCl$_3$) δ 124.0, 65.3, 45.3, 39.7, 30.7, 27.0, 8.2.

HRMS (ESI) m/z: [M + Na]$^+$ Calcd for $C_8H_{15}NO_3SNa$ 228.0665; Found 228.0666.

3-azido-3,7-dimethyloctyl ethanesulfonate (12)

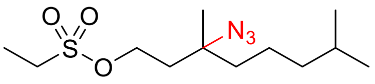

The azidation reaction was conducted with compound **S6** (49.7 mg, 0.2 mmol) and *p*-toluenesulfonyl azide (118.3 mg, 0.6 mmol) as radical trap according to **GP2**.

Isolated yield: 40% (23 mg, 0.08 mmol).

Eluent composition: gradient elution from pure *n*-hexane to *n*-hexane/ethyl acetate (v/v 8:1).

^{1}H NMR (300 MHz, $CDCl_3$) δ = 4.35 – 4.28 (m, 0.8, 2H), 3.14 (q, *J*=7.5, 2H), 1.97 – 1.91 (m, 2H), 1.56 – 1.48 (m, 3H), 1.43 (t, *J*=7.4, 3H), 1.38 – 1.29 (m, 2H), 1.31 (s, 3H), 1.22 – 1.14 (m, 2H), 0.88 (d, *J*=6.6, 6H).

^{13}C NMR (75 MHz, $CDCl_3$) δ 65.7, 62.7, 45.2, 40.3, 39.2, 38.5, 28.0, 23.6, 22.7, 21.8, 8.3.

HRMS (ESI) m/z: $[M + Na]^+$ Calcd for $C_{12}H_{25}N_3O_3SNa$ 314.1508; Found 314.1517.

3-bromo-1-cyclohexyl-3-methylbutyl ethanesulfonate (13)

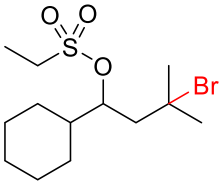

The bromination reaction was conducted according to **GP2** with compound **S7** (52.8 mg, 0.2 mmol) and *p*-toluenesulfonyl bromide (141 mg, 0.6 mmol) as radical trap.

Isolated yield: 55% (37 mg, 0.11 mmol).

Eluent composition: gradient elution from pure *n*-hexane to *n*-hexane/ethyl acetate (v/v 10:1).

^{1}H NMR (300 MHz, $CDCl_3$) δ = 4.98 – 4.90 (m, 1H), 3.16 – 3.09 (m, 2H), 2.30 (dd, *J*=15.8, 2.8, 1H), 2.08 (dd, *J*=15.8, 7.1, 1H), 1.98 – 1.91 (m, 1H), 1.83 – 1.67 (m, 5H), 1.64 (d, *J*=2.0, 6H), 1.43 (t, *J*=7.5, 3H), 1.28 – 1.07 (m, 5H).

^{13}C NMR (75 MHz, $CDCl_3$) δ 84.7, 64.8, 47.3, 47.1, 43.1, 35.7, 33.5, 28.2, 26.8, 26.5, 26.2, 26.1, 8.3.

HRMS (ESI) m/z: $[M + Na]^+$ Calcd for $C_{13}H_{25}[^{81}Br]O_3SNa$ 365.0579; Found 365.0587.

3-azido-1-cyclohexyl-3-methylbutyl ethanesulfonate (14)

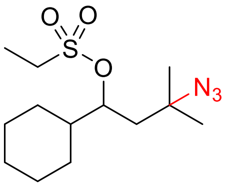

The azidation reaction was conducted according to **GP2** with compound **S7** (52.8 mg, 0.2 mmol) and *p*-toluenesulfonyl azide (118.3 mg, 0.6 mmol) as radical trap.

Isolated yield: 60% (36 mg, 0.12 mmol).

Eluent composition: gradient elution from pure *n*-hexane to *n*-hexane/ethyl acetate (v/v 5:1).

^{1}H NMR (300 MHz, $CDCl_3$) δ = 4.83 – 4.77 (m, 1H), 3.18 – 3.10 (m, 2H), 1.82 – 1.65 (m, 8H), 1.43 (t, *J*=7.4, 3H), 1.35 (d, *J*=6.1, 6H), 1.20 – 0.91 (m, 5H).

^{13}C NMR (75 MHz, CDCl$_3$) δ 83.3, 60.3, 46.8, 43.0, 41.5, 27.9, 27.4, 26.9, 26.5, 26.2, 26.1, 26.1, 8.3.

HRMS (ESI) m/z: $[M + Na]^+$ Calcd for $C_{13}H_{25}N_3O_3SNa$ 326.1514; Found 326.1518.

3-cyano-1-cyclohexyl-3-methylbutyl ethanesulfonate (15)

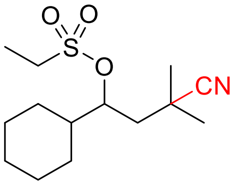

The cyanation reaction was conducted according to **GP2** with compound **S7** (52.8 mg, 0.2 mmol) and *p*-toluenesulfonyl cyanide (114 mg, 0.6 mmol) as radical trap.

Isolated yield: 58% (33 mg, 0.12 mmol).

Eluent composition: gradient elution from pure *n*-hexane to *n*-hexane/ethyl acetate (v/v 10:1).

^{1}H NMR (300 MHz, CDCl$_3$) δ = 4.86 – 4.81 (m, 1H), 3.33 – 3.11 (m, 2H), 2.05 – 1.92 (m, 2H), 1.83 – 1.66 (m, 6H), 1.44 (t, *J*=7.4, 3H), 1.42 (d, *J*=4.4, 6H), 1.33 – 1.03 (m, 5H).

^{13}C NMR (75 MHz, CDCl$_3$) δ 124.8, 81.8, 46.5, 42.4, 40.6, 30.1, 28.2, 28.0, 27.1, 26.4, 26.4, 26.1, 26.0, 8.1.

HRMS (ESI) m/z: $[M + Na]^+$ Calcd for $C_{14}H_{25}NO_3SNa$ 310.1447; Found 310.1456.

tert-butyl 4-(3-bromo-1-((ethylsulfonyl)oxy)-3-methylbutyl)piperidine-1-carboxylate (16)

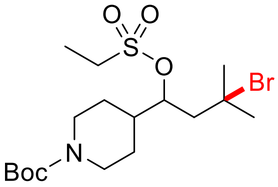

The bromination reaction was conducted according to **GP2** with compound **S8** (72.4 mg, 0.2 mmol) and *p*-toluenesulfonyl bromide (141 mg, 0.6 mmol) as radical trap.

NMR yield: 65%.

Eluent composition: gradient elution from pure *n*-hexane to *n*-hexane/ethyl acetate (v/v 3:1).

^{1}H NMR (300 MHz, CDCl$_3$) δ = 5.05 – 5.00 (m, 1H), 4.19 (br, 2H), 3.19 – 3.10 (m, 2H), 2.67 (br, 2H), 2.27 (dd, *J*=16.0, 2.9, 1H), 2.04 (dd, *J*=15.8, 6.8, 1H), 1.83 (d, *J*=2.0, 6H), 1.74 – 1.74 (m, 1H), 1.46 (s, 9H), 1.44 (t, *J*=7.4, 3H), 1.30 – 1.21 (m, 4H).

^{13}C NMR (75 MHz, CDCl$_3$) δ 154.8, 83.0, 79.7, 64.3, 47.2, 47.0, 43.8, 41.6, 35.9, 33.5, 29.8, 28.6, 27.2, 26.1, 8.3.

HRMS (ESI) m/z: $[M + Na]^+$ Calcd for $C_{17}H_{32}[^{81}Br]NO_5SNa$ 466.1056; Found 466.1066.

5-chloro-2,5-dimethylhexan-3-yl ethanesulfonate (17)

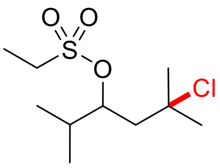

The chlorination reaction was conducted according to **GP2** with compound **S9** (46.7 mg, 0.2 mmol) and *p*-toluenesulfonyl chloride (116.6 mg, 0.6 mmol) as radical trap.

Isolated yield: 50% (25 mg, 0.1 mmol).

Eluent composition: gradient elution from pure *n*-hexane to *n*-hexane/ethyl acetate (v/v 10:1).

^{1}H NMR (400 MHz, $CDCl_3$) δ = 4.99 – 4.95 (m, 1H), 3.16 – 3.10 (m, 2H), 2.36 – 2.28 (m, 1H), 2.09 (dd, *J*=15.7, 3.2, 1H), 2.02 (dd, *J*=15.7, 6.7, 1H), 1.66 (d, *J*=6.1, 6H), 1.44 (t, *J*=7.5, 3H), 0.99 (d, *J*=6.8, 3H), 0.95 (d, *J*=7.0, 3H).

^{13}C NMR (101 MHz, $CDCl_3$) δ 84.1, 68.6, 47.0, 45.0, 33.9, 32.7, 31.9, 17.6, 16.2, 8.3.

HRMS (ESI) m/z: $[M + Na]^+$ Calcd for $C_{10}H_{21}O_3SClNa$ 279.0798; Found 279.0792.

5-cyano-2,5-dimethylhexan-3-yl ethanesulfonate (18)

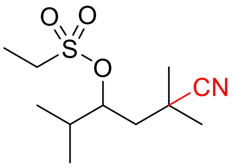

The cyanation reaction was conducted according to **GP2** with compound **S9** (44.2 mg, 0.2 mmol) and *p*-toluenesulfonyl cyanide (114 mg, 0.6 mmol) as radical trap.

Isolated yield: 49% (24 mg, 0.1 mmol)

Eluent composition: gradient elution from pure *n*-hexane to *n*-hexane/ethyl acetate (v/v 10:1).

^{1}H NMR (300 MHz, $CDCl_3$) δ = 4.90 – 4.85 (m, 1H), 3.34 – 3.12 (m, 2H), 2.36 (m, 1H), 1.96 (dd, *J*=15.1, 8.8, 1H), 1.67 (dd, *J*=15.1, 3.3, 1H), 1.44 (t, *J*=7.4, 3H), 1.43 (d, *J*=5.9, 6H), 1.01 (d, *J*=6.9, 3H), 0.94 (d, *J*=7.0, 3H).

^{13}C NMR (75 MHz, $CDCl_3$) δ 124.8, 81.9, 46.5, 39.7, 32.2, 30.0, 28.0, 26.3, 17.7, 16.3, 8.1.

HRMS (ESI) m/z: $[M + Na]^+$ Calcd for $C_{11}H_{21}NO_3SNa$ 270.1134; Found 270.1141.

2-chloro-2,7-dimethyloctan-4-yl ethanesulfonate (19)

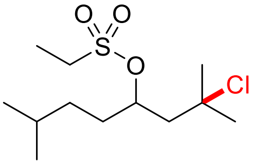

The chlorination reaction was conducted according to **GP2** with compound **S10** (46.7 mg, 0.2 mmol) and *p*-toluenesulfonyl chloride (116.6 mg, 0.6 mmol) as radical trap.

Isolated yield: 61% (35 mg, 0.12 mmol).

Eluent composition: gradient elution from pure *n*-hexane to *n*-hexane/ethyl acetate (v/v 10:1).

^{1}H NMR (300 MHz, $CDCl_3$) δ = 5.06 – 4.98 (m, 1H), 3.16 – 3.08 (m, 2H), 2.20 (dd, *J*=15.4, 6.3, 1H), 2.10 (dd, *J*=15.5, 4.0, 1H), 1.93 – 1.76 (m, 2H), 1.65 (d, *J*=7.5, 6H), 1.59 – 1.52 (m, 1H), 1.43 (t, *J*=7.4, 3H), 1.33 – 1.23 (m, 2H), 0.91 (d, *J*=2.2, 3H), 0.89 (d, *J*=2.1, 3H).

^{13}C NMR (75 MHz, CDCl$_3$) δ 80.4, 68.4, 49.7, 46.7, 34.2, 34.0, 33.3, 32.0, 28.0, 22.6, 22.6, 8.3.

HRMS (ESI) m/z: [M + Na]$^+$ Calcd for $C_{12}H_{25}O_3SClNa$ 307.1111; Found 307.1112.

2-azido-2,7-dimethyloctan-4-yl ethanesulfonate (**20**)

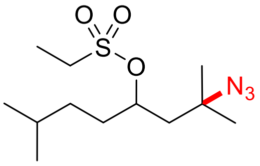

The azidation reaction was conducted according to **GP2** with compound **S10** (49.7 mg, 0.2 mmol) and *p*-toluenesulfonyl azide (118.3 mg, 0.6 mmol) as radical trap.

NMR yield: 69%.

Eluent composition: gradient elution from pure *n*-hexane to *n*-hexane/ethyl acetate (v/v 10:1).

^{1}H NMR (300 MHz, CDCl$_3$) δ = 4.93 – 4.86 (m, 1H), 3.17 – 3.09 (m, 2H), 1.95 (dd, *J*=15.1, 6.5, 1H), 1.81 – 1.72 (m, 3H), 1.44 (t, *J*=7.5, 3H), 1.36 (d, *J*=6.1, 6H), 1.32 – 1.22 (m, 3H), 0.92 (d, *J*=2.3, 3H), 0.89 (d, *J*=2.3, 3H).

^{13}C NMR (75 MHz, CDCl$_3$) δ 79.8, 60.2, 46.6, 45.3, 34.1, 33.5, 28.0, 27.1, 26.1, 22.6, 22.6, 8.3.

HRMS (ESI) m/z: [M + Na]$^+$ Calcd for $C_{12}H_{25}N_3O_3SNa$ 314.1514; Found 314.1519.

2-azido-7-ethyl-2-methylundecan-4-yl ethanesulfonate (21)

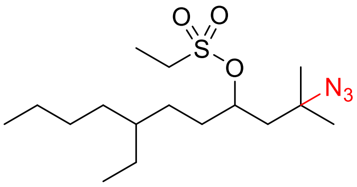

The azidation reaction was conducted according to **GP2** with compound **S11** (60.9 mg, 0.2 mmol) and *p*-toluenesulfonyl azide (118.3 mg, 0.6 mmol) as radical trap.

Isolated yield: 37% (25 mg, 0.07 mmol).

Eluent composition: gradient elution from pure *n*-hexane to *n*-hexane/ethyl acetate (v/v 5:1).

^{1}H NMR (300 MHz, CDCl$_3$) δ = 4.93 – 4.84 (m, 1H), 3.12 (q, *J*=7.5, 2H), 1.95 (dd, *J*=15.1, 6.5, 1H), 1.76 (dd, J=15.1, 4.6, 1H), 1.81 – 1.64 (m, 2H), 1.43 (t, *J*=7.5, 3H), 1.36 (d, *J*=6.4, 6H), 1.33 – 1.22 (m, 11H), 0.91 – 0.82 (m, 6H).

^{13}C NMR (75 MHz, CDCl$_3$) δ 80.0, 79.9, 60.2, 46.6, 45.4, 38.8, 38.7, 33.4, 32.8, 29.0, 29.0, 27.8, 27.1, 26.0, 25.8, 23.2, 14.3, 10.9, 10.9, 8.3.

HRMS (ESI) m/z: [M + Na]$^+$ Calcd for $C_{16}H_{33}N_3O_3SNa$ 370.2140; Found 370.2148.

2-cyano-7-ethyl-2-methylundecan-4-yl ethanesulfonate (22)

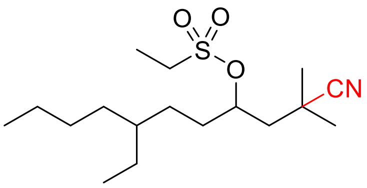

The cyanation reaction was conducted according to **GP2** with compound **S11** (60.9 mg, 0.2 mmol) and *p*-toluenesulfonyl cyanide (114 mg, 0.6 mmol) as radical trap.

Isolated yield: 51% (33 mg, 0.1 mmol).

Eluent composition: gradient elution from pure *n*-hexane to *n*-hexane/ethyl acetate (v/v 1:1).

^{1}H NMR (300 MHz, $CDCl_3$) δ = 4.98 – 4.89 (m, 1H), 3.32 – 3.12 (m, 2H), 2.08 (dd, *J*=14.9, 8.4, 1H), 1.87 – 1.82 (m, 1H), 1.74 (dd, *J*=14.9, 3.9, 1H), 1.45 (t, *J*=7.4, 3H), 1.44 (d, *J*=2.6, 6H), 1.33 – 1.19 (m, 12H), 0.91 – 0.82 (m, 6H).

^{13}C NMR (75 MHz, $CDCl_3$) δ 124.8, 78.4, 46.4, 44.5, 38.8, 33.0, 30.1, 29.0, 28.0, 27.8, 26.4, 25.7, 23.2, 14.3, 11.0, 10.8, 8.1.

HRMS (ESI) m/z: $[M + Na]^+$ Calcd for $C_{17}H_{33}NO_3SNa$ 354.2079; Found 354.2082.

2-bromo-2-methyldecan-4-yl ethanesulfonate (23)

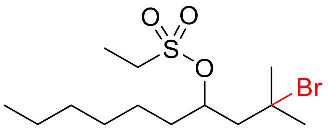

The bromination reaction was conducted according to **GP2** with compound **S12** (55.2 mg, 0.2 mmol) and *p*-toluenesulfonyl bromide (141 mg, 0.6 mmol) as radical trap.

Isolated yield: 75% (51 mg, 0.15 mmol).

Eluent composition: gradient elution from pure *n*-hexane to *n*-hexane/ethyl acetate (v/v 10:1).

^{1}H NMR (300 MHz, $CDCl_3$) δ = 5.08 – 4.99 (m, 1H), 3.17 – 3.08 (m, 2H), 2.26 – 2.22 (m, 2H), 1.84 (d, *J*=3.2, 6H), 1.43 (t, J=7.5, 1H), 1.41 – 1.19 (m, 8H), 0.91 – 0.85 (m, 3H).

^{13}C NMR (75 MHz, $CDCl_3$) δ 81.0, 64.4, 51.1, 46.8, 36.3, 35.7, 33.7, 31.8, 29.2, 24.4, 22.7, 14.2, 8.3.

HRMS (ESI) m/z: $[M + Na]^+$ Calcd for $C_{13}H_{27}O_3[^{81}Br]SNa$ 365.0864; Found 365.0874.

2-azido-2-methyldecan-4-yl ethanesulfonate (24)

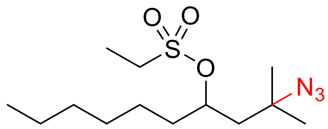

The azidation reaction was conducted according to **GP2** with compound **S12** (55.2 mg, 0.2 mmol) and *p*-toluenesulfonyl azide (118.3 mg, 0.6 mmol) as radical trap.

Isolated yield: 49% (30 mg, 0.1 mmol).

Eluent composition: gradient elution from pure *n*-hexane to *n*-hexane/ethyl acetate (v/v 10:1).

^{1}H NMR (300 MHz, $CDCl_3$) δ = 4.94 – 4.85 (m, 1H), 3.16 – 3.09 (m, 2H), 1.95 (dd, *J*=15.1, 6.5, 1H), 1.81 – 1.73 (m, 2H), 1.43 (t, *J*=7.4, 3H), 1.36 (d, *J*=6.0, 6H), 1.40 – 1.24 (m, 9H), 0.92 – 0.85 (m, 3H).

^{13}C NMR (75 MHz, CDCl$_3$) δ 79.6, 60.2, 46.6, 45.4, 36.2, 31.8, 29.1, 27.1, 26.1, 24.7, 22.7, 14.2, 8.3.

HRMS (ESI) m/z: $[M + Na]^+$ Calcd for $C_{13}H_{27}N_3O_3SNa$ 328.1671; Found 328.1675.

2-cyano-2-methyldecan-4-yl ethanesulfonate (25)

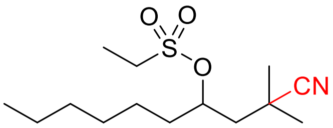

The cyanation reaction was conducted according to **GP2** with compound **S12** (55.2 mg, 0.2 mmol) and *p*-toluenesulfonyl cyanide (114 mg, 0.6 mmol) as radical trap.

Isolated yield: 52% (30 mg, 0.1 mmol).

Eluent composition: gradient elution from pure *n*-hexane to *n*-hexane/ethyl acetate (v/v 10:1).

^{1}H NMR (300 MHz, CDCl$_3$) δ = 5.00 – 4.89 (m, 1H), 3.33 – 3.10 (m, 2H), 2.07 (dd, *J*=14.9, 8.3, 1H), 1.91 – 1.80 (m, 2H), 1.75 (dd, *J*=15.0, 4.0, 1H), 1.44 (t, *J*=7.4, 3H), δ = 1.43 (d, *J*=2.3, 6H), 1.39 – 1.24 (m, 8H), 0.91 – 0.85 (m, 3H).

^{13}C NMR (75 MHz, CDCl$_3$) δ 124.0, 78.1, 46.4, 44.5, 35.7, 31.7, 30.1, 29.1, 28.0, 26.5, 24.6, 22.7, 14.2, 8.1.

HRMS (ESI) m/z: $[M + Na]^+$ Calcd for $C_{14}H_{27}NO_3SNa$ 312.1604; Found 312.1612.

(1R,2R,5R)-2-(2-chloropropan-2-yl)-5-methylcyclohexyl ethanesulfonate (26)

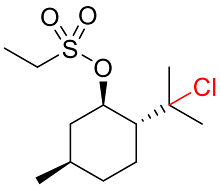

The chlorination reaction was conducted according to **GP2** with compound **S13** (49.3 mg, 0.2 mmol) and *p*-toluenesulfonyl chloride (116.6 mg, 0.6 mmol) as radical trap.

Isolated yield: 80% (45 mg, 0.16 mmol).

Eluent composition: gradient elution from pure *n*-hexane to *n*-hexane/ethyl acetate (v/v 10:1).

^{1}H NMR (300 MHz, CDCl$_3$) δ = 4.84 – 4.75 (m, 1H), 3.14 (q, *J*=7.5, 2H), 2.41 – 2.34 (m, 1H), 2.26 – 2.18 (m, 1H), 1.90 – 1.81 (m, 1H), 1.77 – 1.71 (m, 1H), 1.68 (d, *J*=3.2, 6H), 1.44 (t, *J*=7.4, 3H), 1.39 – 1.22 (m, 4H), 0.94 (d, *J*=6.5, 3H).

^{13}C NMR (75 MHz, CDCl$_3$) δ 82.4, 73.1, 52.8, 47.3, 42.9, 33.8, 33.4, 31.5, 31.3, 27.6, 21.7, 8.2.

HRMS (ESI) m/z: $[M + Na]^+$ Calcd for $C_{12}H_{23}ClO_3SNa$ 295.1056; Found 295.1058.

(1R,2R,5R)-2-(2-azidopropan-2-yl)-5-methylcyclohexyl ethanesulfonate (27)

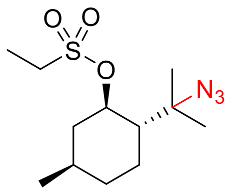

The azidation reaction was conducted according to **GP2** with compound **S13** (49.3 mg, 0.2 mmol) and *p*-toluenesulfonyl azide (118.3 mg, 0.6 mmol) as radical trap.

Isolated yield: 50% (30 mg, 0.1 mmol).

Eluent composition: gradient elution from pure *n*-hexane to *n*-hexane/ethyl acetate (v/v 8:1).

^{1}H NMR (300 MHz, CDCl$_3$) δ = 4.74 – 4.66 (m, 1H), 3.18 – 3.10 (m, 2H), 2.37 – 2.30 (m, 1H), 2.04 – 1.96 (m, 1H), 1.73 – 1.64 (m, 2H), 1.58 – 1.47 (m, 1H), 1.44 (t, *J*=7.5, 3H), 1.34 (d, *J*=5.1, 6H), 1.33 – 1.21 (m, 2H), 1.19 – 1.05 (m, 1H), 0.93 (d, *J*=6.5, 3H).

^{13}C NMR (75 MHz, CDCl$_3$) δ 81.2, 63.4, 49.7, 47.2, 42.7, 33.8, 31.5, 26.7, 25.0, 24.5, 21.7, 8.2.

HRMS (ESI) m/z: $[M + Na]^+$ Calcd for $C_{12}H_{23}N_3O_3SNa$ 312.1358; Found 312.1363.

(1R,2R,5R)-2-(2-cyanopropan-2-yl)-5-methylcyclohexyl ethanesulfonate (28)

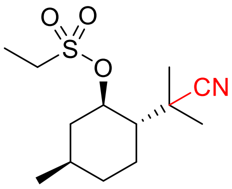

The cyanation reaction was conducted according to **GP2** with compound **S13** (49.3 mg, 0.2 mmol) and *p*-toluenesulfonyl cyanide (114 mg, 0.6 mmol) as radical trap.

Isolated yield: 47% (25 mg, 0.1 mmol).

Eluent composition: gradient elution from pure *n*-hexane to *n*-hexane/ethyl acetate (v/v 1:1).

^{1}H NMR (300 MHz, CDCl$_3$) δ = 4.74 – 4.64 (m, 1H), 3.35 – 3.12 (m, 2H), 2.46 – 2.38 (m, 1H), 1.98 – 1.78 (m, 2H), 1.78 – 1.69 (m, 1H), 1.43 (t, *J*=7.4, 3H), 1.40 (d, *J*=4.9, 6H), 1.35 – 1.23 (m, 2H), 1.20 – 1.05 (m, 2H), 0.94 (d, *J*=6.4, 3H).

^{13}C NMR (75 MHz, CDCl$_3$) δ 126.0, 79.7, 48.2, 46.7, 42.5, 33.6, 33.4, 31.3, 26.1, 25.4, 22.2, 21.6, 8.0.

HRMS (ESI) m/z: $[M + Na]^+$ Calcd for $C_{13}H_{23}NO_3SNa$ 296.1291; Found 296.1296.

(3S,8R,9S,10R,13S,14S)-5-cyano-10,13-dimethyl-17-oxohexadecahydro-1H-cyclopenta[a]phenanthren-3-yl ethanesulfonate (29)

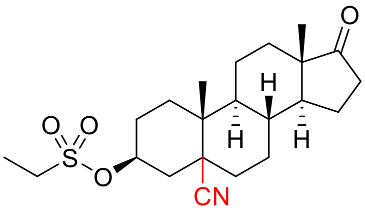

The cyanation reaction was conducted according to **GP2** with compound **S15** (76.1 mg, 0.2 mmol) and *p*-toluenesulfonyl cyanide (114 mg, 0.6 mmol) as radical trap.

Isolated yield: 50% (40 mg, 0.1 mmol).

Eluent composition: gradient elution from pure *n*-hexane to *n*-hexane/ethyl acetate (v/v 3:1).

^{1}H NMR (300 MHz, CDCl$_3$) δ = 4.65 – 4.53 (m, 1H), 3.08 (q, *J*=7.4, 2H), 2.48 – 2.37 (m, 1H), 2.12 – 1.88 (m, 3H), 1.83 – 1.74 (m, 4H), 1.69 – 1.52 (m, 4H), 1.40 (t, *J*=7.4, 3H), 1.35 – 1.16 (m, 6H), 1.09 – 0.89 (m, 2H), 0.84 (s, 6H), 0.74 – 0.63 (m, 1H).

^{13}C NMR (75 MHz, CDCl$_3$) δ 221.2, 124.8, 81.5, 54.3, 51.4, 47.9, 46.1, 44.9, 36.9, 35.9, 35.5, 35.3, 35.1, 31.6, 30.8, 28.8, 28.3, 21.9, 20.6, 13.9, 12.3, 8.4.

HRMS (ESI) m/z: [M + Na]$^+$ Calcd for $C_{22}H_{33}NO_4SNa$ 430.2191; Found 430.2196.

2-chloro-2,6-dimethylheptan-4-ol (4a)

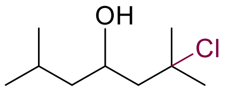

According to the general procedure **GP3** for removal of esylate group of the product **4** (54 mg, 0.2 mmol), **4a** was isolated in yield 50% (18 mg, yellowish oil).

^{1}H NMR (300 MHz, CDCl$_3$) δ = 4.13 – 4.05 (m, 1H), 1.8 – 1.7 (m, 1H), 1.5 – 1.4 (m, 2H), 1.3 (d, *J*=17.4, 6H), 1.23 – 1.14 (m, 2H), 0.9 (d, *J*=3.4, 3H), 0.9 (d, *J*=3.4, 3H).

^{13}C NMR (75 MHz, CDCl$_3$) δ 71.9, 67.9, 48.5, 47.7, 32.3, 27.9, 24.5, 23.3, 22.5.

HRMS (ESI) m/z: [M+ Na]$^+$ Calcd for $C_9H_{19}OClNa$ 201.1124; Found 201.1128.

IV. EPR measurements

All low-temperature EPR measurements were carried out using a standard quartz EPR tube. Prior to introducing the sample mixture, the tube was sealed with a rubber septum and thoroughly degassed using three cycles of argon purging and vacuum evacuation by Schlenk technique, to ensure an oxygen-free environment.

For spin-trapping experiments involving 5,5-dimethyl-1-pyrroline N-oxide (DMPO), measurements were performed using an EPR flat cell. A similar degassing procedure was applied to the flat cell before sample loading. After the sample components were introduced, DMPO was added as the final component. The cell was then immediately subjected to the spectrometer.

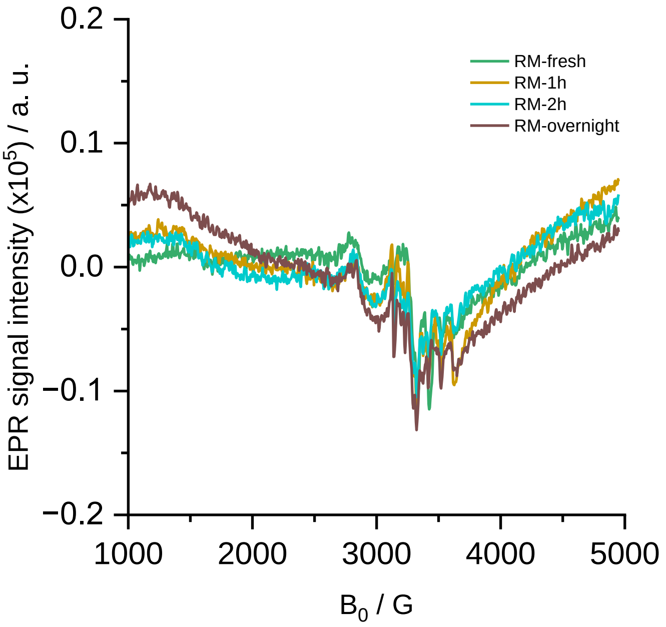

Figure S1. *EPR spectra of reaction mixture of the chlorination of substrate* ***S1*** *with time.*

V. Computational information

V.1. Computational details

The reaction mechanism underlying C–H activation in cyanation reaction was explored using density functional theory (DFT). In a first step, optimizations were performed using the BPW91 functional[2] and the split-valence double-ζ SVP basis set[3] including solvent effects through the polarizable continuum model (PCM)[4] with dichloromethane (ε = 8.93) as the solvent. Subsequently, single-point energy refinements were conducted with the BPW91 functional and the split-valence triple-ζ TZVP basis set,[3a, 5] also incorporating solvent effects for those steps. All calculations have been performed using the Gaussian16 suite package,[6] visualizing and drawing structures through the facilities provided by the Jmol program. In all cases, frequency calculations were performed in order to describe minima and first-order transition states (TS) with none and one imaginary frequency, respectively. Just for the specific case of TSs involving HAT between [Co$^{(III)}$]–H complex and substrate, optimizations were conducted in vacuum to compute entropic and thermal contributions to the energy, with subsequent single-point energy refinement calculations in dichloromethane solution.

V.2. Reaction Profile Benchmarks Using Various Computational Methods

Table S2. Benchmark of reaction profiles for different computational methods. ΔG values are reported in kcal/mol relative to the $[Co]^{III}$−H species and substrate.

Method	Dispersion included	$TS_{HAT-1,6}$	$TS_{HAT-1,5}$	$TS_{HAT-1,7}$
**BPW91/TZVP//BPW91/SVP (ref.)**	no	13.3	15.5	16.3
B3LYP/TZVP//BPW91/SVP	no	11.6	13.6	16.8
B3PW91/TZVP//BPW91/SVP	no	7.8	10.1	13.1
B97D3/TZVP//BPW91/SVP	no	11.9	13.5	18.5
BP86/TZVP//BPW91/SVP	no	13.6	15.6	19.0
M06L/TZVP//BPW91/SVP	no	12.6	14.7	19.1
PBE1PBE1/TZVP//BPW91/SVP	no	4.1	6.3	9.4
B3LYP/TZVP//BPW91/SVP	yes	10.4	12.0	16.2
B3PW91/TZVP//BPW91/SVP	yes	6.3	8.1	12.4
B97D3/TZVP//BPW91/SVP	yes	13.1	14.7	19.3
BP86/TZVP//BPW91/SVP	yes	11.9	13.5	18.1
M06L/TZVP//BPW91/SVP	yes	12.7	14.7	19.2
PBE1PBE1/TZVP//BPW91/SVP	yes	3.4	5.3	9.1
B3LYP/TZVP//B3LYP/SVP	yes	9.5	11.2	15.5
B3PW91/TZVP//B3PW91/SVP	yes	5.8	7.8	12.2
B97D3/TZVP//B97D3//SVP	yes	14.1	15.6	20.6
BP86/TZVP//BP86/SVP	yes	12.4	14.1	19.0
M06L/TZVP//M06L/SVP	yes	11.0	13.0	17.8
PBE1PBE1/TZVP//PBE1PBE/SVP	yes	4.4	6.5	10.6

V.3. Theoretical Study of Radical Trapping in DMPO

In this study, three radical species were considered: the hydrogen radical (H•), a secondary alkyl radical, and the tert-butyl radical. A set of computational approaches was applied to perform a mini benchmark study, allowing us to evaluate the accuracy of different methods for predicting hyperfine couplings.[7] First, geometry optimizations were carried out using the BPW91 functional and the split-valence double-ζ SVP basis set,[3] incorporating solvent effects through the polarizable continuum model (PCM) with dichloromethane (ε = 8.93) as the solvent.[4] Next, single-point calculations for hyperfine coupling constants were performed using several benchmark functionals and basis sets, also including solvent effects at this stage. All calculations were conducted using the Gaussian16 software.[6] Molecular structures were visualized and analyzed using Chemcraft and Jmol.[8]

***Table S1**. Calculated Hyperfine Splitting Constants (in Gauss, G) at different Level of Theory and Experimentally Obtained Hyperfine Splitting Constants for tert-butyl radical, secondary radical and H-radical.*

Method	Isotropic hyperfine Splitting Constants (G)									
	tert-butyl radical			secondary radical			H radical			
	nitronyl-N	β-H	C*	nitronyl-N	β-H	C*	nitronyl-N	β^1-H	β^2-H	C*
Experimental value	14.3	19.8	-	14.3	23.2	-	15	19.8	19.8	-
MP2*	14.6	16.0	9.1	11.8	25.2	9.5	10.2	14.0	24.5	9.1
PBE0*	12.8	14.9	8.6	11.9	23.8	8.8	11.8	14.2	24.0	8.7
M06*	11.3	15.5	9.4	10.7	25.6	9.3	11.0	15.2	25.8	9.1
B3LYP*	10.5	15.6	8.7	9.3	25.0	9.0	9.1	14.8	25.1	8.9
wB97XD*	10.9	14.5	8.5	9.7	23.3	8.8	9.5	13.9	23.5	8.8
CAM-B3LYP*	10.4	15.0	8.3	9.2	23.9	8.6	9.0	14.3	24.1	8.5
APFD*	10.2	15.3	8.8	9.1	24.4	8.9	8.9	14.5	24.6	8.8
B3P86*	9.1	15.2	8.8	7.9	24.4	8.9	7.7	14.5	24.6	8.8
TPSSH*	8.8	16.8	9.0	7.7	26.7	9.2	7.5	15.6	26.7	9.1
LC-BLYP*	8.4	14.6	7.9	7.2	23.0	8.1	7.0	14.0	23.4	8.1
LC-wHPBE*	8.1	14.5	8.1	6.9	22.9	8.2	6.7	13.9	23.2	8.1
BP86*	6.4	16.0	9.5	5.0	26.1	9.6	4.8	15.3	26.2	9.4

*All geometry optimizations were done at BPW91/def2-SVP level of theory, whereas TZVP basis was used for SP calculations.

V.4. Optimized Cartesian Coordinates

[Co(III)]-H complex

Co	0.008708	0.705337	-0.038115
O	-1.307071	-0.666535	0.056950
O	1.310977	-0.681482	0.108114
N	1.265295	2.051757	0.261176
N	-1.256345	2.064391	-0.212612
C	-1.475663	4.632680	-0.142042
C	-0.617654	3.386903	-0.397483
C	0.622507	3.362535	0.509038
C	1.480120	4.622007	0.332974
C	0.627342	5.880141	0.578842
C	-0.626001	5.906288	-0.307264
C	-2.556433	1.921555	-0.301062
C	2.567739	1.912539	0.318370
C	-3.286290	0.691070	-0.187982
C	-2.614598	-0.571226	0.024004
C	-4.703483	0.767228	-0.265806
C	-3.451210	-1.740204	0.216936
C	-5.514345	-0.356794	-0.119748
C	-4.842507	-1.583748	0.131017
C	3.295681	0.687260	0.152624
C	2.615476	-0.582870	0.031402
C	4.714501	0.767236	0.139647
C	3.442611	-1.760493	-0.148725
C	5.517885	-0.361552	-0.012437
C	4.835921	-1.599967	-0.159484
C	-7.053508	-0.241799	-0.218433
C	-7.570898	0.727407	0.875898
C	-7.439779	0.309038	-1.615583
C	-7.754792	-1.603460	-0.026942
C	7.059750	-0.240115	-0.023905
C	7.496826	0.678267	-1.194512
C	7.752711	-1.608083	-0.200048
C	7.537918	0.375382	1.316638
C	-2.833184	-3.126104	0.514584
C	-3.904126	-4.218263	0.731780
C	-1.953556	-3.566533	-0.682754
C	-1.985151	-3.044884	1.810872
C	2.808232	-3.156804	-0.343052

C	1.895043	-3.130064	-1.596554
C	1.991652	-3.538008	0.918379
C	3.864695	-4.262316	-0.562846
H	-0.253830	3.419356	-1.449599
H	-1.898593	4.586378	0.884068
H	-2.330999	4.669074	-0.844733
H	1.902900	4.640793	-0.693996
H	2.335035	4.614949	1.037121
H	1.242849	6.784648	0.403396
H	0.324862	5.912179	1.647447
H	-0.323510	6.007008	-1.371487
H	-1.244191	6.796037	-0.074921
H	0.252741	3.338924	1.559969
H	-3.165800	2.827050	-0.464235
H	3.176045	2.811516	0.517693
H	-5.147648	1.759068	-0.441000
H	-5.456625	-2.479191	0.268688
H	5.166342	1.764768	0.251140
H	5.443229	-2.500443	-0.295177
H	-8.673632	0.829490	0.818450
H	-7.136898	1.740353	0.768589
H	-7.315309	0.356924	1.888360
H	-8.540670	0.406020	-1.707127
H	-7.089085	-0.366811	-2.420482
H	-7.000647	1.308490	-1.800503
H	-7.548021	-2.041644	0.969036
H	-7.449674	-2.339647	-0.796131
H	-8.851905	-1.475218	-0.109344
H	8.600444	0.784426	-1.217600
H	7.066300	1.694340	-1.104292
H	7.174109	0.261293	-2.168991
H	7.504066	-2.308708	0.620961
H	8.852278	-1.474466	-0.198469
H	7.480837	-2.091823	-1.158575
H	7.246735	-0.263474	2.173787
H	7.107399	1.381458	1.485181
H	8.641964	0.478868	1.327991
H	-4.536074	-4.374909	-0.164527
H	-4.569645	-3.992360	1.588269
H	-3.402316	-5.181382	0.950256

H	-1.158944	-2.829028	-0.882621
H	-2.568204	-3.676646	-1.598631
H	-1.478621	-4.546690	-0.474776
H	-2.622049	-2.777090	2.677844
H	-1.187284	-2.288819	1.717037
H	-1.515620	-4.025681	2.027117
H	1.115126	-2.355576	-1.502448
H	1.400868	-4.112465	-1.736583
H	2.490852	-2.918721	-2.507142
H	2.652622	-3.601363	1.806153
H	1.511805	-4.528561	0.783632
H	1.204342	-2.793310	1.122591
H	4.543641	-4.376585	0.305157
H	4.483601	-4.081229	-1.463733
H	3.351124	-5.233239	-0.706849
H	0.204077	0.783197	-1.455359

Substrate

S	2.019400	-0.762591	0.087093
O	0.928904	0.443094	-0.246602
O	1.769196	-1.905806	-0.820742
O	2.065247	-0.983137	1.547796
C	-2.256734	1.863732	0.663296
C	-1.583713	0.662759	1.349693
C	-1.283459	-0.493768	0.366995
C	-0.395578	0.055455	-0.775098
C	-0.975207	1.300347	-1.455113
C	-1.416823	2.439829	-0.502666
C	-0.250024	3.317666	-0.013508
C	-2.549987	-1.246040	-0.153274
C	-2.210618	-2.344971	-1.179712
C	-3.349984	-1.859614	1.011338
C	3.492285	0.087131	-0.449098
C	4.458193	0.394385	0.425929
H	-0.677723	-1.256304	0.903168
H	-0.623269	0.980374	1.802768
H	-2.206074	0.304777	2.191989
H	-3.251956	1.558477	0.276763
H	-2.455848	2.661658	1.408195
H	-0.206045	-0.726668	-1.533282

H	-0.248899	1.673515	-2.205202
H	-1.856822	0.951261	-2.032129
H	-2.082783	3.102219	-1.096205
H	0.324831	3.725900	-0.868742
H	-0.630343	4.177392	0.573763
H	0.461139	2.757772	0.621637
H	-3.214181	-0.512197	-0.659357
H	-3.118482	-2.923477	-1.440653
H	-1.804389	-1.942678	-2.127278
H	-1.465635	-3.058619	-0.771968
H	-4.234416	-2.406661	0.629874
H	-2.730428	-2.582815	1.581099
H	-3.719306	-1.098845	1.724260
H	3.520925	0.272787	-1.532427
H	5.384950	0.880171	0.087341
H	4.347477	0.168751	1.496596

TS_t

Co	-1.393057	0.677362	0.937030
S	2.632055	-1.424842	-0.351965
O	-1.284513	2.355545	0.065840
O	-2.993883	0.396266	-0.053419
O	3.580780	-1.971163	-1.602761
O	3.444884	-0.504681	0.482353
O	1.959276	-2.581151	0.284806
N	-1.725288	-0.757888	2.085126
N	0.194727	0.932106	1.885753
C	1.575758	-0.073349	3.814863
C	0.536010	-0.252207	2.702295
C	-0.804244	-0.769331	3.245070
C	-0.628814	-2.093208	4.001619
C	0.416331	-1.931144	5.121050
C	1.751606	-1.394598	4.585514
C	1.042443	1.921534	1.735326
C	-2.684194	-1.646942	1.969271
C	0.846618	3.088637	0.925032
C	-0.357778	3.278046	0.147998
C	1.868808	4.075183	0.944010
C	-0.514184	4.546657	-0.535304
C	1.750877	5.276169	0.248575

C	0.536018	5.471669	-0.462666
C	-3.671612	-1.698220	0.932071
C	-3.781843	-0.648760	-0.058099
C	-4.565260	-2.803295	0.933651
C	-4.829693	-0.783558	-1.051851
C	-5.574831	-2.940019	-0.015310
C	-5.665191	-1.906202	-0.987080
C	2.888084	6.323567	0.280012
C	3.141473	6.769241	1.743340
C	4.182410	5.694279	-0.297149
C	2.550930	7.578346	-0.553136
C	-6.527982	-4.157384	0.012707
C	-5.703749	-5.462298	-0.135723
C	-7.566975	-4.113319	-1.127886
C	-7.292569	-4.182472	1.361413
C	-1.806891	4.876927	-1.315483
C	-1.782233	6.297007	-1.922701
C	-1.980430	3.870993	-2.481189
C	-3.021396	4.802425	-0.353149
C	-5.019445	0.279063	-2.158157
C	-3.722994	0.379149	-3.003072
C	-5.347992	1.648801	-1.510598
C	-6.175042	-0.071726	-3.121336
C	0.129349	-0.808526	-1.242155
C	1.461714	-0.501419	-1.296418
C	5.030898	-2.084875	-1.367098
C	5.335621	-3.350544	-0.529424
C	5.697727	-2.092373	-2.747227
C	4.980513	-4.594141	-1.378585
C	6.782117	-3.363034	0.059989
C	5.474896	-3.373433	-3.589564
C	5.729612	-4.630033	-2.722012
C	7.137207	-2.059153	0.801732
C	6.984772	-4.563414	1.003910
C	4.108891	-3.410223	-4.299137
H	0.919533	-1.027804	2.003344
H	1.254184	0.737394	4.503891
H	2.549143	0.225265	3.380593
H	-0.305228	-2.881632	3.289500
H	-1.590108	-2.425573	4.441684

H	0.567416	-2.903412	5.631541
H	0.020479	-1.235357	5.892380
H	2.205722	-2.147082	3.906748
H	2.470268	-1.250111	5.416965
H	-1.184753	-0.005131	3.962754
H	1.995796	1.874226	2.288307
H	-2.757788	-2.432861	2.741300
H	2.768386	3.860163	1.540933
H	0.406738	6.418420	-0.996165
H	-4.431050	-3.562141	1.720065
H	-6.448710	-1.991170	-1.746287
H	3.958150	7.518194	1.790337
H	3.433274	5.918590	2.389097
H	2.232956	7.226135	2.182571
H	5.016819	6.424754	-0.278702
H	4.033654	5.372169	-1.346588
H	4.503332	4.806858	0.281842
H	1.651015	8.100364	-0.173437
H	2.381866	7.335086	-1.620286
H	3.392317	8.297506	-0.508047
H	-6.366992	-6.350931	-0.113019
H	-4.964148	-5.578400	0.679948
H	-5.148419	-5.474799	-1.094126
H	-8.214488	-3.216986	-1.066054
H	-8.226987	-5.001117	-1.070397
H	-7.088403	-4.122869	-2.126601
H	-7.895093	-3.262440	1.493834
H	-6.603476	-4.257368	2.224773
H	-7.980058	-5.051563	1.407582
H	-0.965593	6.427552	-2.660073
H	-1.680227	7.084448	-1.149949
H	-2.735384	6.482059	-2.455769
H	-2.040327	2.837411	-2.102725
H	-1.129903	3.940739	-3.188652
H	-2.909226	4.090056	-3.045588
H	-2.920995	5.547131	0.461592
H	-3.110971	3.799939	0.098906
H	-3.960167	5.022880	-0.899962
H	-2.865977	0.663014	-2.368832
H	-3.835720	1.144061	-3.797207

H	-3.499221	-0.589272	-3.493675
H	-6.289923	1.590141	-0.929579
H	-5.480375	2.423109	-2.293023
H	-4.539501	1.972852	-0.834237
H	-7.152679	-0.129567	-2.603379
H	-6.007094	-1.029688	-3.652208
H	-6.260237	0.719297	-3.891998
H	-0.562400	-0.135174	-0.002517
H	-0.562940	-0.287246	-1.917669
H	-0.178231	-1.797330	-0.869426
H	1.884594	0.395214	-1.769965
H	5.326047	-1.172939	-0.815327
H	4.650606	-3.321939	0.345203
H	5.374690	-1.192465	-3.308857
H	6.785649	-1.965614	-2.564570
H	3.887152	-4.578943	-1.558038
H	5.174630	-5.522741	-0.808080
H	7.504836	-3.476821	-0.777560
H	6.246469	-3.360756	-4.389494
H	6.820383	-4.726474	-2.535474
H	5.442421	-5.538821	-3.290233
H	8.117106	-2.156114	1.309307
H	7.211258	-1.184250	0.128124
H	6.380442	-1.818078	1.575720
H	8.011819	-4.566306	1.419019
H	6.279661	-4.518360	1.859519
H	6.834684	-5.535394	0.497805
H	3.963013	-2.507996	-4.925907
H	4.040618	-4.293268	-4.965556
H	3.264859	-3.451043	-3.586661

TS_i

Co	0.802588	0.973952	0.903798
S	-1.000548	-2.679374	-0.252747
O	2.309082	1.089006	-0.275064
O	0.243765	2.655836	0.177422
O	-2.114166	-2.890136	-1.495934
O	-0.160822	-3.895763	-0.184381
O	-1.767538	-2.235477	0.942847
N	-0.520059	1.044260	2.242202

N	1.581710	-0.403576	1.948392
C	1.197457	-1.784944	4.092141
C	0.631079	-0.955028	2.934424
C	-0.183305	0.242542	3.441025
C	-1.341338	-0.217413	4.338317
C	-0.802237	-1.070446	5.502109
C	0.049834	-2.248663	5.008637
C	2.796781	-0.880485	1.813482
C	-1.604424	1.780622	2.207040
C	3.744400	-0.533294	0.797262
C	3.445662	0.439078	-0.233530
C	5.000422	-1.199553	0.837114
C	4.473341	0.682893	-1.228086
C	5.995357	-0.962451	-0.105583
C	5.682353	-0.015915	-1.119106
C	-1.931690	2.759330	1.209582
C	-0.937596	3.216085	0.262919
C	-3.220218	3.355247	1.267062
C	-1.284561	4.353668	-0.564963
C	-3.586461	4.402855	0.425893
C	-2.580367	4.876863	-0.459194
C	7.349763	-1.705094	-0.032819
C	8.039756	-1.386614	1.318675
C	7.106993	-3.232723	-0.139815
C	8.306214	-1.288904	-1.170570
C	-5.008572	5.006963	0.486416
C	-6.046127	3.907129	0.142313
C	-5.192316	6.171074	-0.510395
C	-5.283825	5.546057	1.913787
C	4.246865	1.688136	-2.380353
C	5.466648	1.790723	-3.322909
C	3.038919	1.227559	-3.237032
C	3.992202	3.099021	-1.791326
C	-0.254355	4.982132	-1.530705
C	0.191941	3.932011	-2.579204
C	0.966021	5.484619	-0.715071
C	-0.830443	6.191702	-2.299776
C	-0.462783	0.052623	-0.551772
C	-0.087896	-1.332718	-0.862225
C	-2.535672	-4.262332	-1.800730

C	-3.544458	-4.763272	-0.737041
C	-3.114384	-4.241397	-3.221239
C	-4.837307	-3.922785	-0.860628
C	-3.772221	-6.307563	-0.779427
C	-4.475476	-3.517857	-3.376646
C	-5.453192	-3.981698	-2.269368
C	-2.453840	-7.106455	-0.769973
C	-4.660936	-6.768891	0.391323
C	-4.348141	-1.985234	-3.449690
H	-0.074969	-1.598434	2.363146
H	1.930315	-1.180348	4.669542
H	1.738074	-2.671611	3.705807
H	-2.058455	-0.805390	3.728452
H	-1.891959	0.652049	4.748942
H	-1.648822	-1.438348	6.115651
H	-0.190920	-0.428123	6.172579
H	-0.593639	-2.959091	4.448227
H	0.462609	-2.812538	5.868962
H	0.504808	0.873517	4.052078
H	3.130214	-1.645104	2.536338
H	-2.327957	1.679427	3.034945
H	5.161652	-1.925982	1.648371
H	6.444872	0.183892	-1.878070
H	-3.930084	2.959367	2.009455
H	-2.833375	5.720352	-1.108729
H	9.012743	-1.913074	1.396596
H	7.421773	-1.701317	2.181756
H	8.229649	-0.300193	1.422130
H	8.065688	-3.787547	-0.085021
H	6.616624	-3.491421	-1.098742
H	6.459537	-3.606147	0.676944
H	8.545043	-0.207925	-1.138434
H	7.888450	-1.519030	-2.170079
H	9.262399	-1.840500	-1.078753
H	-7.077384	4.313052	0.183396
H	-5.993918	3.056229	0.848718
H	-5.877175	3.505746	-0.876190
H	-4.498349	7.009123	-0.303834
H	-6.222695	6.570736	-0.436036
H	-5.039337	5.848591	-1.558749

H	-4.557729	6.336581	2.187513
H	-5.214058	4.748238	2.678248
H	-6.302374	5.979696	1.979217
H	5.705218	0.826391	-3.813610
H	6.375308	2.147247	-2.798943
H	5.245482	2.520550	-4.126173
H	2.125514	1.146663	-2.624205
H	3.240518	0.240236	-3.698605
H	2.844736	1.950709	-4.054314
H	4.871032	3.442441	-1.209960
H	3.112212	3.101186	-1.126964
H	3.818707	3.830990	-2.605582
H	0.672266	3.067211	-2.092554
H	0.919772	4.378522	-3.285949
H	-0.675227	3.573600	-3.169255
H	0.659305	6.272258	0.001730
H	1.729152	5.919917	-1.391060
H	1.434767	4.662044	-0.148035
H	-1.152762	7.007735	-1.623339
H	-1.689724	5.913769	-2.941692
H	-0.047633	6.607237	-2.964026
H	0.551757	-0.535160	0.166245
H	-0.226073	0.796289	-1.324764
H	-1.432149	0.173981	-0.045855
H	0.640164	-1.579323	-1.645768
H	-1.618965	-4.881501	-1.783885
H	-3.087507	-4.548911	0.252767
H	-2.360680	-3.808567	-3.909940
H	-3.234292	-5.302953	-3.524400
H	-4.581528	-2.875303	-0.605466
H	-5.582387	-4.239730	-0.105342
H	-4.308785	-6.561528	-1.719998
H	-4.897881	-3.847035	-4.350817
H	-5.779897	-5.020864	-2.487304
H	-6.375385	-3.365225	-2.305156
H	-2.657463	-8.191048	-0.671118
H	-1.862242	-6.974518	-1.695785
H	-1.808029	-6.805829	0.079845
H	-4.819187	-7.864872	0.356037
H	-4.185874	-6.532121	1.365825

H	-5.660142	-6.294287	0.382385
H	-3.660131	-1.683010	-4.264270
H	-5.333618	-1.521381	-3.656093
H	-3.952044	-1.552755	-2.512920

[Co(III)]-H-substrate complex

Co	0.689569	-0.914500	0.768554
S	-1.225551	1.650719	0.496213
O	-0.457291	-1.751076	-0.509761
O	2.078710	-1.100619	-0.554917
O	-1.080784	3.051718	-0.435061
O	-2.302431	0.825613	-0.101033
O	-1.363289	2.098534	1.907220
N	1.947284	-0.689885	2.164369
N	-0.600082	-0.998609	2.129000
C	-0.899034	-0.671727	4.671282
C	-0.108245	-0.398841	3.386747
C	1.348211	-0.857830	3.513717
C	2.050978	-0.160063	4.687634
C	1.265873	-0.400972	5.990326
C	-0.206673	0.014855	5.863525
C	-1.791875	-1.536319	2.046801
C	3.248351	-0.605273	2.035263
C	-2.385866	-2.130738	0.886336
C	-1.678903	-2.207376	-0.370753
C	-3.702913	-2.642073	1.025047
C	-2.361536	-2.853060	-1.474784
C	-4.376321	-3.245723	-0.035347
C	-3.662512	-3.331755	-1.260619
C	3.987496	-0.550312	0.806517
C	3.349614	-0.793756	-0.469442
C	5.380869	-0.288219	0.897554
C	4.188298	-0.722506	-1.651377
C	6.194475	-0.221495	-0.231788
C	5.551841	-0.441322	-1.480183
C	-5.814823	-3.783727	0.143210
C	-5.826740	-4.866097	1.253604
C	-6.752326	-2.618158	0.552298
C	-6.367998	-4.415575	-1.151936
C	7.706135	0.077009	-0.101973

C	7.902000	1.458839	0.574047
C	8.416405	0.105358	-1.472037
C	8.373766	-1.019590	0.767818
C	-1.672112	-3.046411	-2.845055
C	-2.592889	-3.730454	-3.880419
C	-1.258232	-1.673334	-3.432406
C	-0.430243	-3.955263	-2.654372
C	3.610681	-0.961626	-3.065812
C	2.462834	0.042252	-3.345378
C	3.089092	-2.417683	-3.160381
C	4.668121	-0.771690	-4.176421
C	1.486914	1.947806	0.639734
C	0.429750	0.954052	0.188566
C	-2.256706	3.480962	-1.202455
C	-3.287228	4.163525	-0.267546
C	-1.752952	4.403179	-2.320064
C	-2.672153	5.484522	0.252160
C	-4.698025	4.325078	-0.918227
C	-1.253796	5.799054	-1.869597
C	-2.264833	6.436511	-0.885801
C	-5.243677	3.006489	-1.500732
C	-5.712486	4.899680	0.088808
C	0.180798	5.789647	-1.311452
H	-0.112595	0.698687	3.214807
H	-0.967754	-1.766076	4.851017
H	-1.933803	-0.288564	4.574784
H	2.122826	0.927552	4.475614
H	3.084894	-0.536344	4.811768
H	1.751616	0.148230	6.821197
H	1.323659	-1.478696	6.254479
H	-0.269719	1.116248	5.735099
H	-0.754896	-0.221243	6.797081
H	1.333058	-1.953704	3.716981
H	-2.412955	-1.544885	2.958430
H	3.854515	-0.570415	2.957362
H	-4.184104	-2.541494	2.009605
H	-4.164812	-3.811119	-2.106429
H	5.802015	-0.129346	1.901688
H	6.168800	-0.388858	-2.382458
H	-6.852167	-5.260729	1.403242

H	-5.479756	-4.464503	2.225292
H	-5.169588	-5.717351	0.986704
H	-7.790555	-2.982343	0.690724
H	-6.767908	-1.829076	-0.225121
H	-6.434471	-2.147368	1.502569
H	-5.759896	-5.278677	-1.486735
H	-6.415468	-3.685409	-1.983361
H	-7.397596	-4.785189	-0.978315
H	8.980855	1.690684	0.682445
H	7.451197	1.491668	1.584771
H	7.438966	2.265556	-0.027939
H	8.341191	-0.865368	-1.999978
H	9.493479	0.321391	-1.330892
H	8.006949	0.891063	-2.136616
H	8.255654	-2.020035	0.306640
H	7.936914	-1.063408	1.784234
H	9.458984	-0.821575	0.879693
H	-3.506422	-3.138651	-4.086946
H	-2.902865	-4.746547	-3.566485
H	-2.046492	-3.836872	-4.838112
H	-0.574722	-1.137907	-2.753305
H	-2.149680	-1.037184	-3.602254
H	-0.749106	-1.810283	-4.407755
H	-0.732719	-4.955897	-2.285817
H	0.275283	-3.515602	-1.928765
H	0.100571	-4.095309	-3.617386
H	1.653869	-0.063718	-2.603535
H	2.036433	-0.132516	-4.353426
H	2.836486	1.085152	-3.312263
H	3.915677	-3.139952	-3.006845
H	2.654039	-2.608825	-4.162106
H	2.311709	-2.610985	-2.402624
H	5.507610	-1.489020	-4.089167
H	5.088459	0.253324	-4.187045
H	4.193685	-0.943215	-5.162463
H	2.495248	1.560020	0.409775
H	1.373857	2.909953	0.101126
H	1.431260	2.154567	1.723873
H	0.421733	0.816004	-0.908875
H	-2.690710	2.567947	-1.650673

H	-3.426827	3.481610	0.598651
H	-0.968487	3.874231	-2.898319
H	-2.606976	4.536811	-3.016668
H	-1.782207	5.225455	0.860005
H	-3.369258	5.993614	0.945316
H	-4.619358	5.050303	-1.757299
H	-1.232656	6.432974	-2.782167
H	-3.169960	6.749772	-1.447744
H	-1.836239	7.369235	-0.463832
H	-6.284697	3.142044	-1.854801
H	-4.656544	2.639410	-2.363798
H	-5.251820	2.202957	-0.736442
H	-6.712484	4.998499	-0.377665
H	-5.819386	4.231448	0.968436
H	-5.425477	5.900964	0.461273
H	0.885631	5.329004	-2.032119
H	0.529850	6.823766	-1.117282
H	0.258459	5.221127	-0.366214

[Co(II)] metalloradical

Co	0.000003	0.688646	0.000043
O	1.306962	-0.649982	-0.142636
O	-1.306968	-0.649964	0.142751
N	-1.237406	2.033567	-0.304777
N	1.237427	2.033560	0.304830
C	1.460306	4.606200	0.336991
C	0.590507	3.352410	0.492856
C	-0.590477	3.352415	-0.492796
C	-1.460272	4.606207	-0.336925
C	-0.594162	5.870936	-0.485907
C	0.594199	5.870930	0.485986
C	2.545139	1.900143	0.385960
C	-2.545115	1.900153	-0.385944
C	3.281671	0.684371	0.204882
C	2.615204	-0.563473	-0.092316
C	4.699609	0.749723	0.294697
C	3.443841	-1.724775	-0.342231
C	5.505222	-0.367887	0.086580
C	4.834223	-1.580019	-0.237158
C	-3.281657	0.684384	-0.204883

C	-2.615206	-0.563457	0.092366
C	-4.699592	0.749737	-0.294767
C	-3.443860	-1.724753	0.342265
C	-5.505216	-0.367869	-0.086676
C	-4.834235	-1.579997	0.237113
C	7.044639	-0.262864	0.197444
C	7.568122	0.757054	-0.846844
C	7.426522	0.218329	1.621260
C	7.743787	-1.615296	-0.056943
C	-7.044627	-0.262845	-0.197616
C	-7.568157	0.757081	0.846640
C	-7.743789	-1.615275	0.056747
C	-7.426444	0.218338	-1.621453
C	2.813520	-3.089189	-0.705250
C	3.877361	-4.177807	-0.969172
C	1.928472	-3.574087	0.471798
C	1.965356	-2.945923	-1.996767
C	-2.813561	-3.089165	0.705333
C	-1.965510	-2.945903	1.996925
C	-1.928409	-3.574053	-0.471640
C	-3.877421	-4.177788	0.969159
H	0.157170	3.352614	1.518945
H	1.951198	4.596733	-0.659425
H	2.266205	4.619840	1.096931
H	-1.951165	4.596732	0.659491
H	-2.266170	4.619856	-1.096866
H	-1.221727	6.770372	-0.327666
H	-0.217441	5.934068	-1.529238
H	0.217478	5.934053	1.529318
H	1.221765	6.770366	0.327755
H	-0.157141	3.352621	-1.518886
H	3.146244	2.799683	0.601596
H	-3.146212	2.799693	-0.601602
H	5.147503	1.727001	0.530745
H	5.447986	-2.467811	-0.418309
H	-5.147473	1.727013	-0.530850
H	-5.448009	-2.467785	0.418241
H	8.670940	0.852557	-0.781008
H	7.136491	1.764714	-0.691512
H	7.314723	0.437089	-1.876904

H	8.527333	0.306744	1.721353
H	7.070584	-0.494708	2.391005
H	6.990388	1.209192	1.853136
H	7.542109	-2.003470	-1.074428
H	7.432812	-2.387629	0.673469
H	8.840625	-1.493634	0.037845
H	-8.670972	0.852588	0.780750
H	-7.136515	1.764738	0.691322
H	-7.314808	0.437121	1.876715
H	-7.432763	-2.387619	-0.673632
H	-8.840621	-1.493617	-0.038117
H	-7.542179	-2.003433	1.074251
H	-7.070470	-0.494704	-2.391177
H	-6.990302	1.209200	-1.853317
H	-8.527250	0.306750	-1.721597
H	4.505083	-4.379693	-0.078977
H	4.547487	-3.916488	-1.811884
H	3.370372	-5.126482	-1.233763
H	1.127452	-2.848035	0.690147
H	2.538101	-3.713098	1.387143
H	1.460592	-4.548836	0.226431
H	2.603345	-2.644160	-2.851724
H	1.169647	-2.191655	-1.872091
H	1.492321	-3.914957	-2.254912
H	-1.169794	-2.191631	1.872324
H	-1.492492	-3.914937	2.255104
H	-2.603575	-2.644150	2.851830
H	-2.537958	-3.713067	-1.387038
H	-1.460543	-4.548800	-0.226234
H	-1.127376	-2.847995	-0.689918
H	-4.505076	-4.379658	0.078912
H	-4.547610	-3.916482	1.811824
H	-3.370452	-5.126467	1.233772

Radical substrate

S	1.982533	-0.898120	0.148129
O	0.907399	0.347917	-0.147384
O	1.560149	-2.085334	-0.632038
O	2.143008	-0.985329	1.615670
C	4.389832	0.595345	0.211426

C	3.436844	-0.230952	-0.562874
C	-0.405620	0.029953	-0.735662
C	-1.367549	-0.452269	0.378320
C	-0.892624	1.291155	-1.457312
C	-1.641409	0.735263	1.331803
C	-2.655346	-1.140234	-0.177421
C	-1.305723	2.469280	-0.539790
C	-2.219176	1.960325	0.601667
C	-2.348221	-2.255944	-1.195473
C	-3.516736	-1.710818	0.965309
C	-0.111171	3.290025	-0.019759
H	5.426740	0.426087	-0.141693
H	4.183307	1.682386	0.072753
H	4.327190	0.377409	1.293409
H	3.485372	-0.357968	-1.654621
H	-0.231520	-0.776923	-1.471407
H	-0.821990	-1.237575	0.945415
H	-0.120498	1.612232	-2.185558
H	-1.770702	0.980391	-2.061173
H	-0.683610	1.007314	1.818891
H	-2.316214	0.426500	2.153004
H	-3.266948	-0.373323	-0.700586
H	-1.911503	3.157236	-1.167806
H	-3.214722	1.704490	0.181629
H	-2.401328	2.780458	1.326578
H	-3.279692	-2.781183	-1.484641
H	-1.891774	-1.876328	-2.129424
H	-1.658005	-3.011324	-0.767635
H	-4.421261	-2.206158	0.560891
H	-2.952523	-2.469325	1.546529
H	-3.859277	-0.932410	1.672546
H	0.516083	3.652579	-0.858648
H	-0.464535	4.178610	0.540837
H	0.543833	2.701935	0.649253

TS1-5

S	2.184457	0.173775	0.161386
O	1.062543	0.688794	-0.952517
O	2.037190	0.964088	1.408359
O	3.461315	0.253499	-0.578548

C	2.025255	-2.534496	-0.532593
C	1.672472	-1.493477	0.494027
C	-0.339091	0.209231	-1.085023
C	-0.851615	-0.594085	0.122085
C	-1.188120	1.444234	-1.426852
C	-1.265520	0.287609	1.303335
C	-1.717955	-1.826945	-0.232890
C	-1.581925	2.339479	-0.230264
C	-2.181716	1.465534	0.892949
C	-1.847319	-2.804840	0.950906
C	-3.116927	-1.505849	-0.805190
C	-0.448058	3.259360	0.257220
H	1.553804	-3.500691	-0.270347
H	1.696732	-2.251555	-1.550770
H	3.122917	-2.698524	-0.572534
H	1.871007	-1.716672	1.556047
H	-0.307651	-0.458139	-1.971007
H	0.250277	-1.166511	0.457654
H	-0.653025	2.026481	-2.204176
H	-2.111362	1.065463	-1.910303
H	-0.351356	0.700520	1.775145
H	-1.763163	-0.322926	2.081098
H	-1.163019	-2.358806	-1.036937
H	-2.396035	3.001553	-0.596014
H	-3.162549	1.072142	0.558070
H	-2.388729	2.088284	1.787616
H	-2.338305	-3.741415	0.622521
H	-0.861533	-3.073672	1.377684
H	-2.464618	-2.383244	1.768698
H	-3.624350	-2.446225	-1.098631
H	-3.760366	-1.004520	-0.056426
H	-3.071312	-0.863963	-1.704621
H	-0.073799	3.892813	-0.571556
H	-0.814986	3.936390	1.054621
H	0.414842	2.697226	0.657402

TS1-6

S	-1.658454	-1.142141	0.424740
O	-0.346503	-1.269165	-0.588453
O	-1.196329	-0.978522	1.825019

O	-2.448567	-2.341535	0.073291
C	-3.066012	0.383934	-1.458063
C	-2.453257	0.364215	-0.082376
C	0.553697	-0.154209	-0.946475
C	0.810100	0.862553	0.197614
C	1.839870	-0.810375	-1.472182
C	2.266003	1.380109	0.104007
C	-0.252983	1.979053	0.244632
C	2.916088	-1.083947	-0.400729
C	3.274328	0.241459	0.349810
C	-0.445488	2.603784	1.619320
C	-0.222324	2.991585	-0.892566
C	2.529800	-2.222252	0.561004
H	-3.438479	1.400480	-1.688584
H	-2.344546	0.092964	-2.244685
H	-3.929519	-0.311332	-1.522745
H	-3.093188	0.689745	0.757200
H	0.058851	0.364067	-1.794270
H	0.734109	0.302622	1.150706
H	1.563637	-1.739274	-2.009263
H	2.260954	-0.130889	-2.240230
H	2.424126	2.185568	0.847980
H	2.436212	1.844287	-0.888987
H	-1.337745	1.295697	0.056490
H	3.819791	-1.420791	-0.949729
H	4.285617	0.587787	0.056416
H	3.326404	0.040659	1.439693
H	-1.295290	3.314481	1.625961
H	-0.629705	1.832000	2.390986
H	0.456926	3.176140	1.926650
H	-1.139514	3.612590	-0.898655
H	0.636167	3.688242	-0.776640
H	-0.128377	2.518732	-1.889488
H	2.328588	-3.161662	0.008308
H	3.351220	-2.424193	1.277338
H	1.621289	-1.985349	1.146872

TS1-7

S	1.702688	-1.107984	-0.492839
O	0.226373	-1.117466	0.272462

O	1.530465	-0.785101	-1.929518
O	2.271098	-2.422496	-0.125950
C	2.907702	0.097959	1.746049
C	2.601349	0.237665	0.274083
C	-0.493082	0.121801	0.619190
C	-0.810248	1.040855	-0.599494
C	-1.743680	-0.329202	1.377919
C	-2.314134	1.033850	-0.948660
C	-0.211467	2.473081	-0.422829
C	-2.847020	-0.998427	0.514874
C	-2.863910	-0.398657	-0.915930
C	1.299121	2.470671	-0.602770
C	-0.649801	3.199775	0.861453
C	-2.748236	-2.531699	0.489904
H	3.439631	1.001370	2.101574
H	1.998199	-0.031590	2.361902
H	3.566286	-0.775101	1.931969
H	3.483663	0.387647	-0.376090
H	0.155318	0.663719	1.335207
H	-0.293920	0.592520	-1.470311
H	-1.441957	-0.988548	2.215824
H	-2.149672	0.589576	1.846715
H	-2.449204	1.480030	-1.954552
H	-2.891135	1.674646	-0.248682
H	-3.813169	-0.737887	0.997991
H	-3.892081	-0.436207	-1.328293
H	-2.239645	-1.028188	-1.586053
H	1.852417	3.293954	-0.115176
H	1.881800	1.362226	-0.045631
H	1.638635	2.299693	-1.640282
H	-0.302756	4.251240	0.846746
H	-1.751232	3.219022	0.970435
H	-0.227582	2.733478	1.774123
H	-2.829300	-2.958439	1.509678
H	-3.561328	-2.971629	-0.122217
H	-1.781810	-2.861078	0.061678
H	-0.617212	3.061751	-1.279820

Radical substrate after internal HAT

S	1.961594	-0.948364	0.157444

O	0.936683	0.299726	-0.257322
O	1.621819	-2.146533	-0.645960
O	2.008397	-1.053467	1.633005
C	3.964499	1.015471	0.281890
C	3.523599	-0.246724	-0.446611
C	-0.405343	-0.056803	-0.746019
C	-1.313244	-0.510311	0.427158
C	-0.957537	1.167753	-1.482791
C	-1.570464	0.699690	1.355115
C	-2.561682	-1.237426	-0.048197
C	-1.358231	2.366367	-0.588339
C	-2.216633	1.873691	0.600810
C	-2.440626	-2.234524	-1.168480
C	-3.728130	-1.374146	0.887249
C	-0.165230	3.229970	-0.140288
H	4.955623	1.325159	-0.100719
H	3.256547	1.847507	0.116869
H	4.055184	0.834640	1.368531
H	4.231456	-1.087431	-0.305197
H	-0.263226	-0.886351	-1.463467
H	-0.715147	-1.258966	1.007263
H	-0.234268	1.480725	-2.262846
H	-1.860655	0.809227	-2.019536
H	-0.598540	1.011910	1.786214
H	-2.197073	0.398559	2.216614
H	3.369549	-0.103017	-1.532234
H	-2.003239	3.019476	-1.214579
H	-3.208581	1.553701	0.217235
H	-2.410489	2.715983	1.296771
H	-3.400100	-2.754727	-1.351230
H	-2.128392	-1.780217	-2.133227
H	-1.678287	-3.020492	-0.947549
H	-4.605425	-1.814836	0.374852
H	-3.493909	-2.047302	1.750182
H	-4.048593	-0.412042	1.334285
H	0.426908	3.573023	-1.012133
H	-0.518649	4.131934	0.398454
H	0.522747	2.681498	0.529570

CN-substrate

S	1.730327	0.013216	0.468886
O	2.168872	-1.261163	1.067649
O	2.172404	1.318707	0.993392
O	-2.322624	-0.490360	2.003783
O	-2.958823	-2.566595	0.607902
N	2.844775	-0.067715	-2.296044
C	2.358924	-0.036713	-1.228225
C	-0.712074	-1.223805	0.173752
C	-0.035043	0.008164	0.229056
C	-2.099383	-1.213621	-0.005406
C	-0.711392	1.235277	0.118450
C	-2.817243	0.000114	-0.126214
C	-4.314689	-0.011008	-0.297675
C	-2.100323	1.216781	-0.059695
H	-4.692918	0.957772	-0.670979
H	-4.813154	-0.213038	0.673142
H	-4.635333	-0.808952	-0.994181
H	-0.160249	2.182974	0.175224
H	-2.640387	2.170179	-0.148066
H	-0.160315	-2.167580	0.273647
H	-2.639180	-2.170293	-0.051237

TS_c

S	4.918296	-0.216249	-0.900301
S	-2.769543	-1.102380	0.732095
O	3.700507	0.682576	-0.205961
O	4.902929	-0.000248	-2.364431
O	4.853926	-1.609349	-0.394711
N	-1.466718	-0.229619	-1.676512
C	6.465336	0.477355	1.335207
C	6.324686	0.663596	-0.169265
C	2.334956	0.246325	-0.574960
C	1.755666	-0.559960	0.616185
C	1.523377	1.484135	-0.962663
C	1.408994	0.402236	1.779635
C	0.740628	-1.645259	0.267736
C	1.116803	2.412117	0.206147
C	0.507341	1.568535	1.349007
C	0.898479	-2.351870	-1.048906

C	0.251171	-2.479714	1.416260
C	2.247663	3.339984	0.685517
C	-1.542383	-0.661765	-0.570600
C	-4.262372	-0.274870	0.179690
C	-5.103630	-0.929529	-0.733955
C	-4.560815	1.006315	0.675270
C	-5.733543	1.634903	0.240960
C	-6.607432	1.008938	-0.679303
C	-6.271535	-0.278716	-1.154201
C	-7.870150	1.702387	-1.125883
H	7.385362	0.988772	1.676478
H	5.607655	0.910937	1.880252
H	6.548562	-0.593459	1.596027
H	7.181011	0.245375	-0.734055
H	2.436059	-0.398940	-1.467576
H	2.616601	-1.183004	0.963886
H	2.085610	2.041654	-1.738389
H	0.603291	1.105471	-1.451244
H	2.364408	0.798149	2.178235
H	0.945846	-0.154242	2.615536
H	6.189553	1.716487	-0.479912
H	0.311000	3.069638	-0.184480
H	-0.471805	1.162967	1.026171
H	0.294941	2.216266	2.224468
H	0.172462	-3.178213	-1.155543
H	0.779422	-1.679727	-1.919840
H	1.921585	-2.790063	-1.116868
H	-0.535326	-3.187632	1.102621
H	1.102183	-3.083284	1.814747
H	-0.138589	-1.880312	2.257547
H	2.652807	3.937686	-0.155252
H	1.872629	4.049525	1.450207
H	3.094441	2.781886	1.126932
H	-4.853201	-1.933264	-1.101300
H	-6.938942	-0.785280	-1.866133
H	-3.890593	1.493542	1.395252
H	-5.978333	2.634939	0.627454
H	-8.578245	1.811634	-0.279218
H	-8.384531	1.144060	-1.928579
H	-7.655671	2.724760	-1.494463

Product of cyanation

S	2.194001	-0.998948	-0.028665
O	1.228427	0.345921	-0.253910
O	1.784845	-2.052338	-0.987059
O	2.240195	-1.315842	1.417056
N	-4.103690	0.082353	-1.286381
C	4.293420	0.826712	0.349257
C	3.782627	-0.292109	-0.548248
C	-0.161209	0.118173	-0.684147
C	-1.005419	-0.338271	0.536954
C	-0.639128	1.420395	-1.336437
C	-1.214374	0.859592	1.492216
C	-2.289751	-1.187506	0.168311
C	-0.919734	2.599583	-0.373962
C	-1.785290	2.115773	0.813149
C	-1.894106	-2.451516	-0.649449
C	-3.004996	-1.653423	1.467776
C	0.348709	3.343265	0.081422
C	-3.280368	-0.443403	-0.637562
H	5.297800	1.135189	0.002009
H	3.631256	1.710376	0.314357
H	4.380231	0.486066	1.397130
H	4.447017	-1.178956	-0.541129
H	-0.122522	-0.678212	-1.450973
H	-0.386535	-1.082444	1.078222
H	0.094427	1.711197	-2.115100
H	-1.574906	1.170762	-1.875605
H	-0.220856	1.095083	1.924078
H	-1.843293	0.565931	2.353358
H	3.626067	0.014766	-1.599073
H	-1.526454	3.330865	-0.949203
H	-2.814225	1.913883	0.454272
H	-1.879527	2.928174	1.562735
H	-2.772444	-3.109239	-0.785881
H	-1.501846	-2.204191	-1.651681
H	-1.115235	-3.015663	-0.103054
H	-3.840883	-2.334591	1.222846
H	-2.285329	-2.202938	2.104442
H	-3.414851	-0.809074	2.049426

H	0.943520	3.683666	-0.789447
H	0.081355	4.240599	0.674481
H	1.009021	2.709924	0.702730

By-product of cyanation (CN-substrate)

S	2.176731	-0.001135	-0.246807
O	2.683757	-1.314430	0.294737
O	2.687228	1.311535	0.292732
C	-0.321655	-1.226316	-0.079799
C	0.360445	0.001018	-0.090833
C	-1.720962	-1.211722	-0.017442
C	-0.318682	1.228450	-0.078782
C	-2.444636	0.003662	0.015849
C	-3.950455	-0.001122	0.103631
C	-1.719435	1.216269	-0.015790
H	-4.381804	0.974271	-0.186270
H	-4.278932	-0.214637	1.142147
H	-4.391854	-0.786563	-0.539135
H	0.238985	2.173905	-0.101374
H	-2.260658	2.173258	0.009136
H	0.234799	-2.172392	-0.103187
H	-2.263828	-2.167967	0.006847

VI. References

[1] S. Stoll, A. Schweiger, *Journal of Magnetic Resonance* **2006**, *178*, 42-55.

[2] a) A. D. Becke, *Physical Review A* **1988**, *38*, 3098-3100; b) J. P. Perdew, Y. Wang, *Physical Review B* **1992**, *45*, 13244-13249.

[3] a) R. Ahlrichs, M. Bär, M. Häser, H. Horn, C. Kölmel, *Chemical Physics Letters* **1989**, *162*, 165-169; b) J. A. Pople, *Reviews of Modern Physics* **1999**, *71*, 1267-1274.

[4] a) J. Tomasi, B. Mennucci, R. Cammi, *Chemical Reviews* **2005**, *105*, 2999-3094; b) J. Tomasi, M. Persico, *Chemical Reviews* **1994**, *94*, 2027-2094.

[5] J. P. Perdew, K. Burke, M. Ernzerhof, *Physical Review Letters* **1996**, *77*, 3865-3868.

[6] M. J. Frisch, G. W. Trucks, H. B. Schlegel, G. E. Scuseria, M. A. Robb, J. R. Cheeseman, G. Scalmani, V. Barone, G. A. Petersson, H. Nakatsuji, X. Li, M. Caricato, A. V. Marenich, J. Bloino, B. G. Janesko, R. Gomperts, B. Mennucci, H. P. Hratchian, J. V. Ortiz, A. F. Izmaylov, J. L. Sonnenberg, Williams, F. Ding, F. Lipparini, F. Egidi, J. Goings, B. Peng, A. Petrone, T. Henderson, D. Ranasinghe, V. G. Zakrzewski, J. Gao, N. Rega, G. Zheng, W. Liang, M. Hada, M. Ehara, K. Toyota, R. Fukuda, J. Hasegawa, M. Ishida, T. Nakajima, Y. Honda, O. Kitao, H. Nakai, T. Vreven, K. Throssell, J. A. Montgomery Jr., J. E. Peralta, F. Ogliaro, M. J. Bearpark, J. J. Heyd, E. N. Brothers, K. N. Kudin, V. N. Staroverov, T. A. Keith, R. Kobayashi, J. Normand, K. Raghavachari, A. P. Rendell, J. C. Burant, S. S. Iyengar, J. Tomasi, M. Cossi, J. M. Millam, M. Klene, C. Adamo, R. Cammi, J. W. Ochterski, R. L. Martin, K. Morokuma, O. Farkas, J. B. Foresman, D. J. Fox, Wallingford, CT, **2016**.

[7] Z. W. Windom, A. Perera, R. J. Bartlett, *The Journal of Chemical Physics* **2022**, *156*.

[8] a) Chemcraft - graphical software for visualization of quantum chemistry computations. Version 1.8, build 682. https://www.chemcraftprog.com; b) Jmol development team. (2016). Jmol. Retrieved from http://jmol.sourceforge.net/

VII. NMR SPECTRA

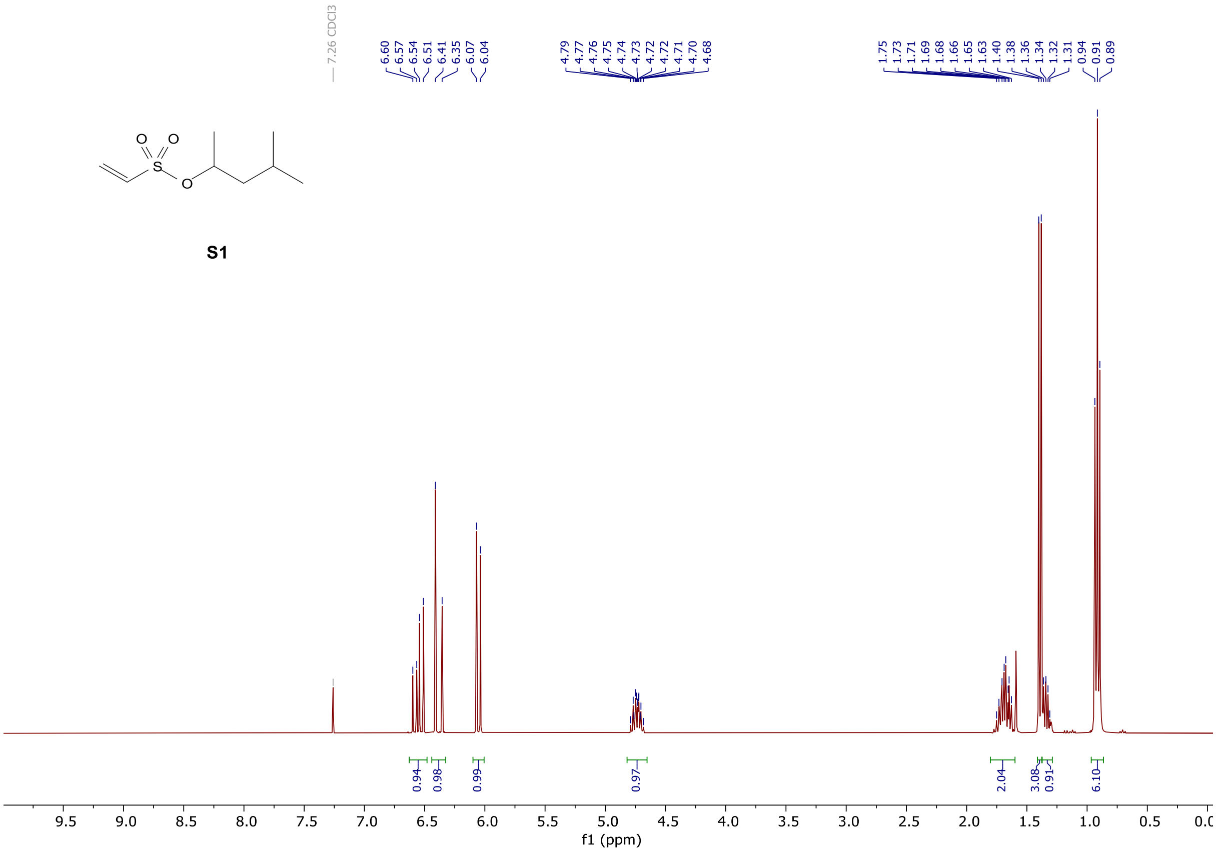

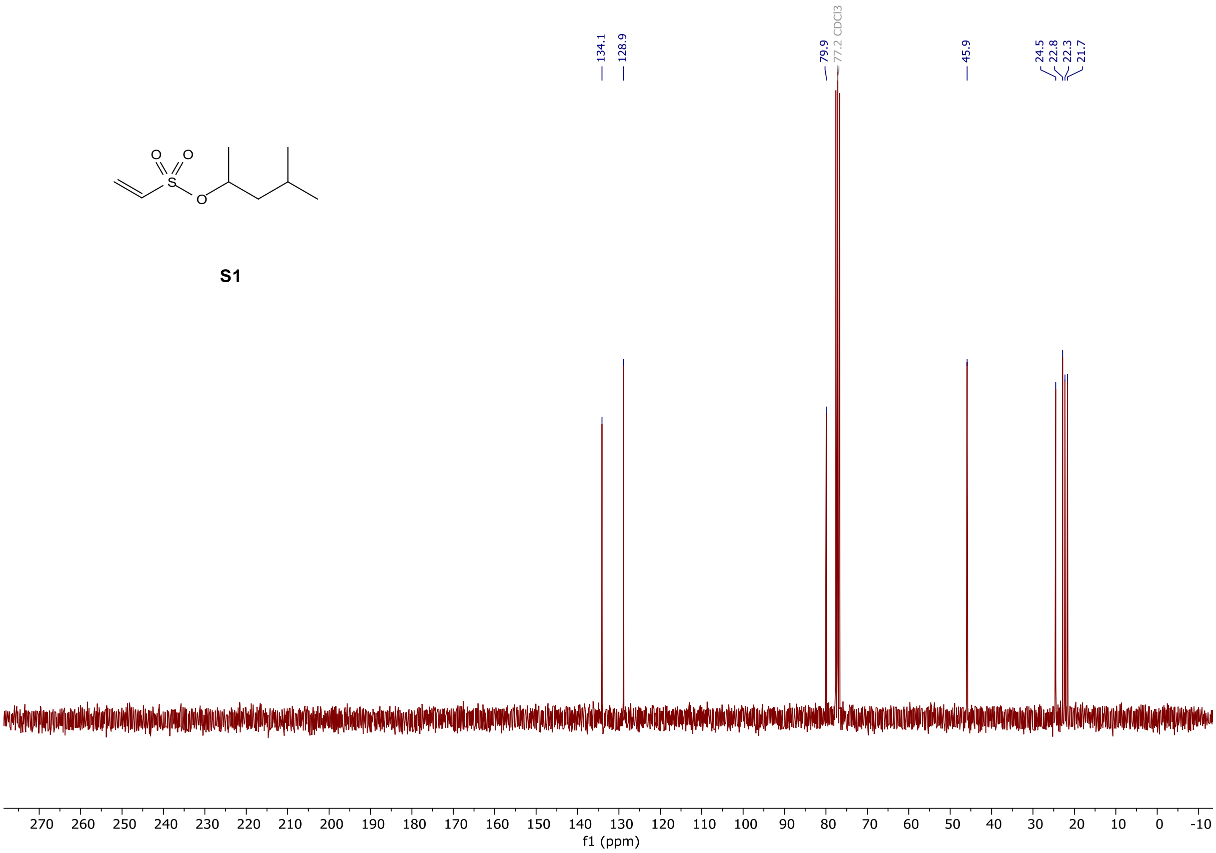

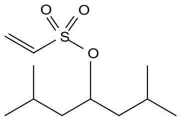

S2

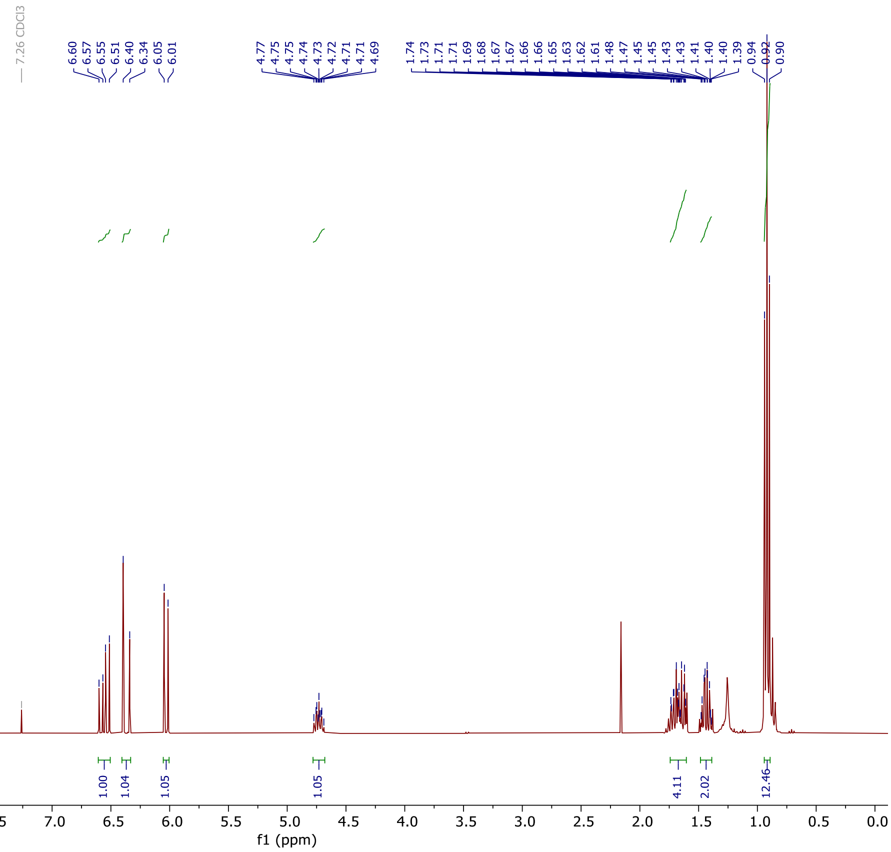

7.26 CDCl3
6.60
6.57
6.55
6.51
6.40
6.34
6.05
6.01
4.77
4.75
4.75
4.74
4.73
4.72
4.71
4.71
4.69
1.74
1.73
1.71
1.71
1.69
1.68
1.67
1.67
1.66
1.65
1.63
1.62
1.61
1.48
1.47
1.45
1.45
1.43
1.43
1.41
1.40
1.40
1.39
0.94
0.92
0.90
1.00
1.04
1.05
1.05
4.11
2.02
12.46
10.0 9.5 9.0 8.5 8.0 7.5 7.0 6.5 6.0 5.5 5.0 4.5 4.0 3.5 3.0 2.5 2.0 1.5 1.0 0.5 0.0
f1 (ppm)

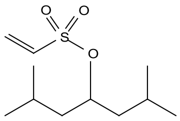

S2

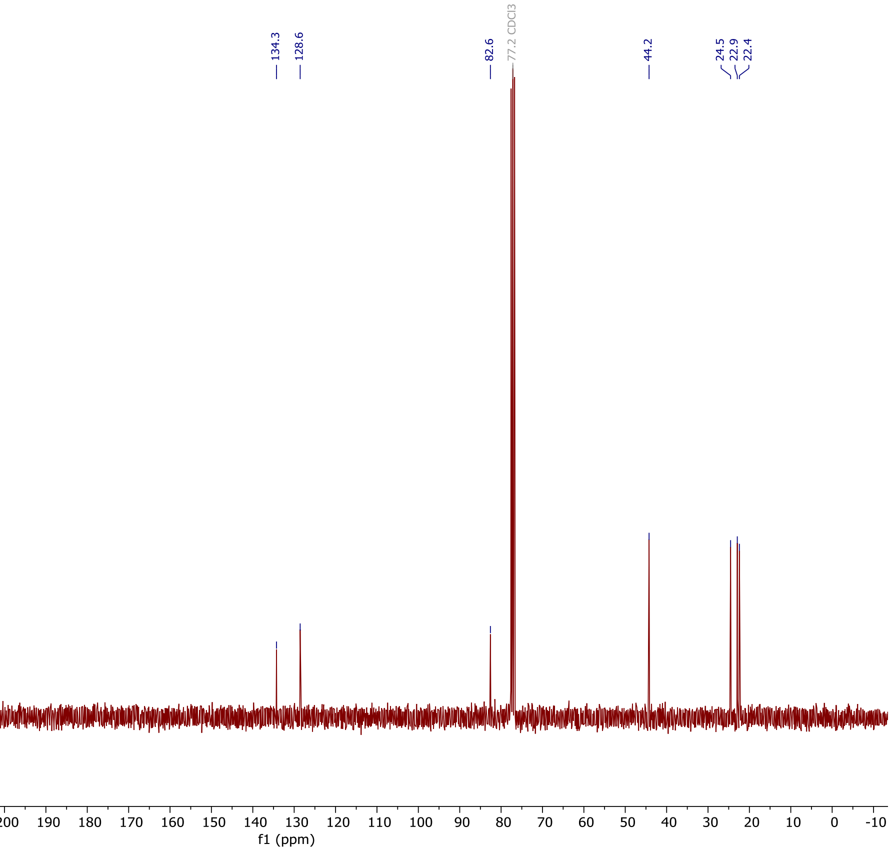

134.3
128.6
82.6
77.2 CDCl3
44.2
24.5
22.9
22.4
270 260 250 240 230 220 210 200 190 180 170 160 150 140 130 120 110 100 90 80 70 60 50 40 30 20 10 0 -10
f1 (ppm)

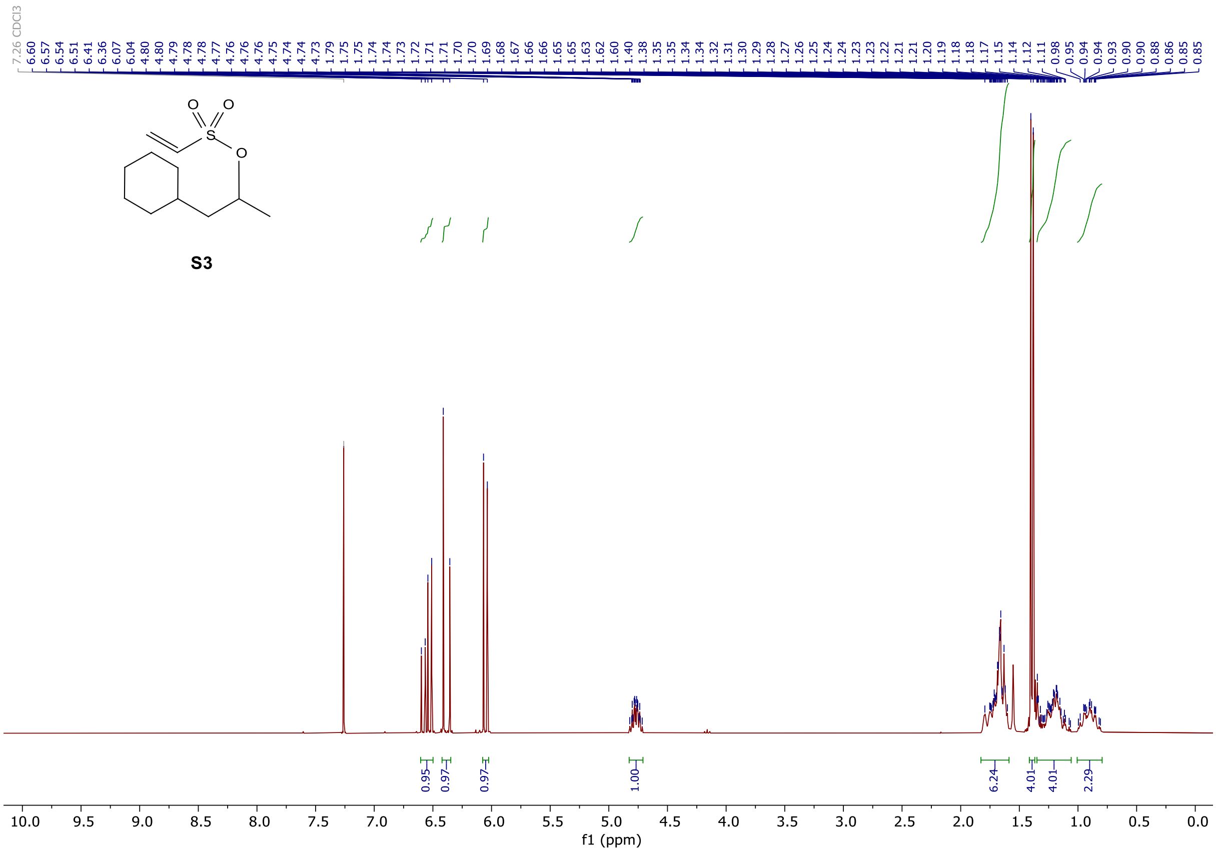
7.26 CDCl3
6.60 6.57 6.54 6.51 6.41 6.36 6.07 6.04 4.80 4.80 4.79 4.78 4.78 4.77 4.76 4.76 4.75 4.74 4.74 4.73 1.79 1.75 1.75 1.74 1.74 1.73 1.72 1.71 1.71 1.70 1.70 1.69 1.68 1.67 1.66 1.66 1.65 1.65 1.63 1.62 1.60 1.40 1.38 1.35 1.35 1.34 1.34 1.32 1.31 1.30 1.29 1.28 1.27 1.26 1.25 1.24 1.24 1.23 1.23 1.22 1.22 1.21 1.21 1.20 1.19 1.18 1.18 1.17 1.15 1.14 1.12 1.11 0.98 0.95 0.94 0.94 0.93 0.90 0.90 0.88 0.86 0.85 0.85
O
O
S
O
S3
0.95
0.97
0.97
1.00
6.24
4.01
4.01
2.29
10.0 9.5 9.0 8.5 8.0 7.5 7.0 6.5 6.0 5.5 5.0 4.5 4.0 3.5 3.0 2.5 2.0 1.5 1.0 0.5 0.0
f1 (ppm)

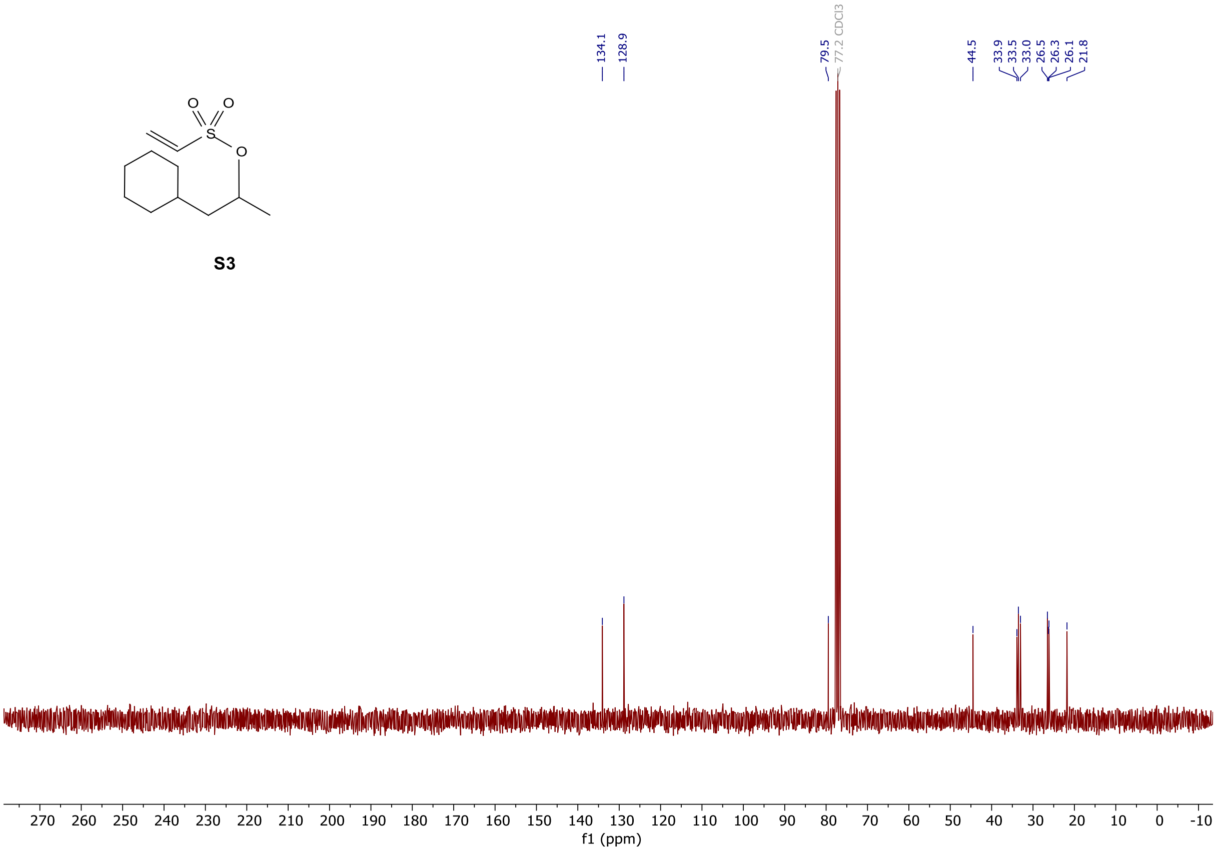
134.1
128.9
79.5
77.2 CDCl3
44.5
33.9
33.5
33.0
26.5
26.3
26.1
21.8
O
O
S
O
S3
270 260 250 240 230 220 210 200 190 180 170 160 150 140 130 120 110 100 90 80 70 60 50 40 30 20 10 0 -10
f1 (ppm)

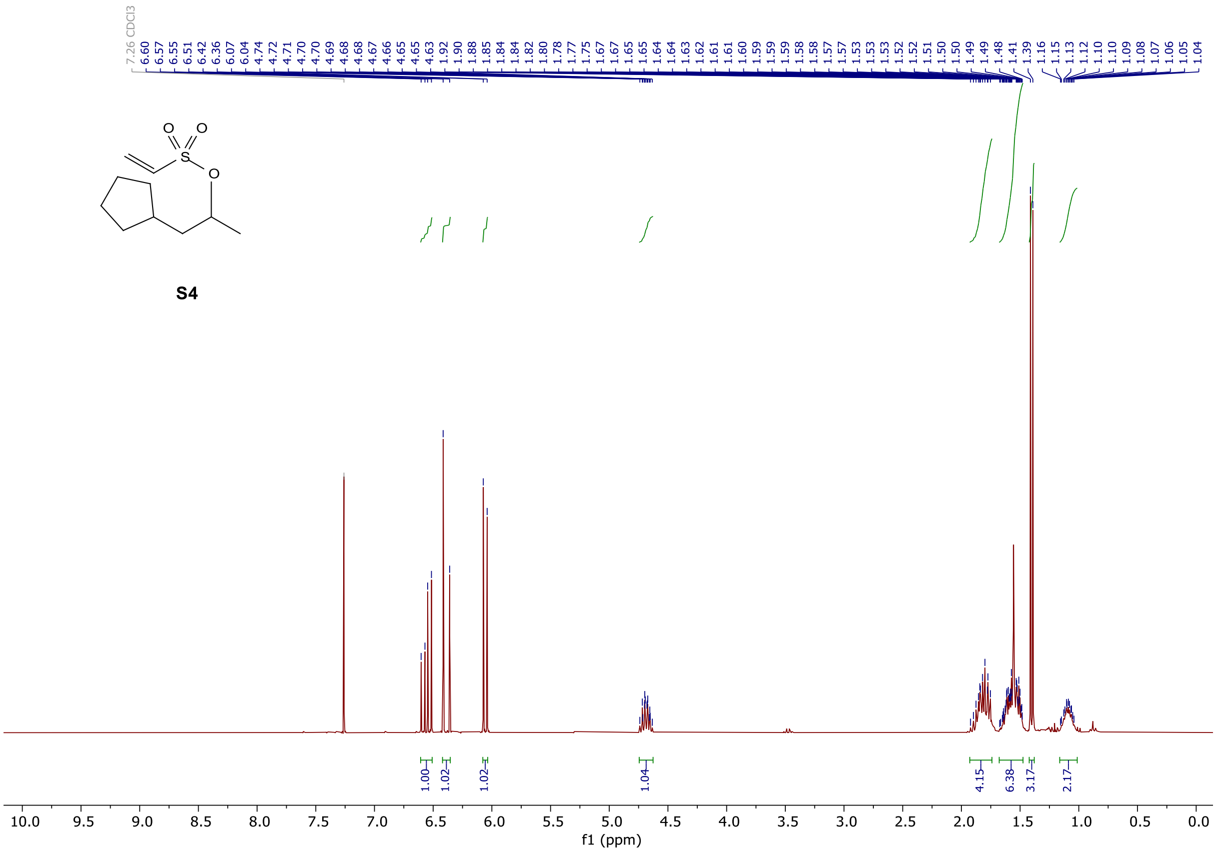
7.26 CDCl3
6.60 6.57 6.55 6.51 6.42 6.36 6.07 6.04 4.74 4.72 4.71 4.70 4.70 4.69 4.68 4.68 4.67 4.66 4.65 4.65 4.63 1.92 1.90 1.88 1.85 1.84 1.84 1.84 1.82 1.80 1.78 1.77 1.75 1.67 1.67 1.65 1.65 1.64 1.63 1.62 1.61 1.61 1.60 1.59 1.59 1.59 1.58 1.58 1.57 1.57 1.53 1.53 1.53 1.52 1.52 1.51 1.50 1.50 1.49 1.49 1.48 1.41 1.39 1.16 1.15 1.13 1.12 1.10 1.09 1.08 1.07 1.06 1.05 1.04
S4
1.00 1.02 1.02 1.04 4.15 6.38 3.17 2.17
10.0 9.5 9.0 8.5 8.0 7.5 7.0 6.5 6.0 5.5 5.0 4.5 4.0 3.5 3.0 2.5 2.0 1.5 1.0 0.5 0.0
f1 (ppm)

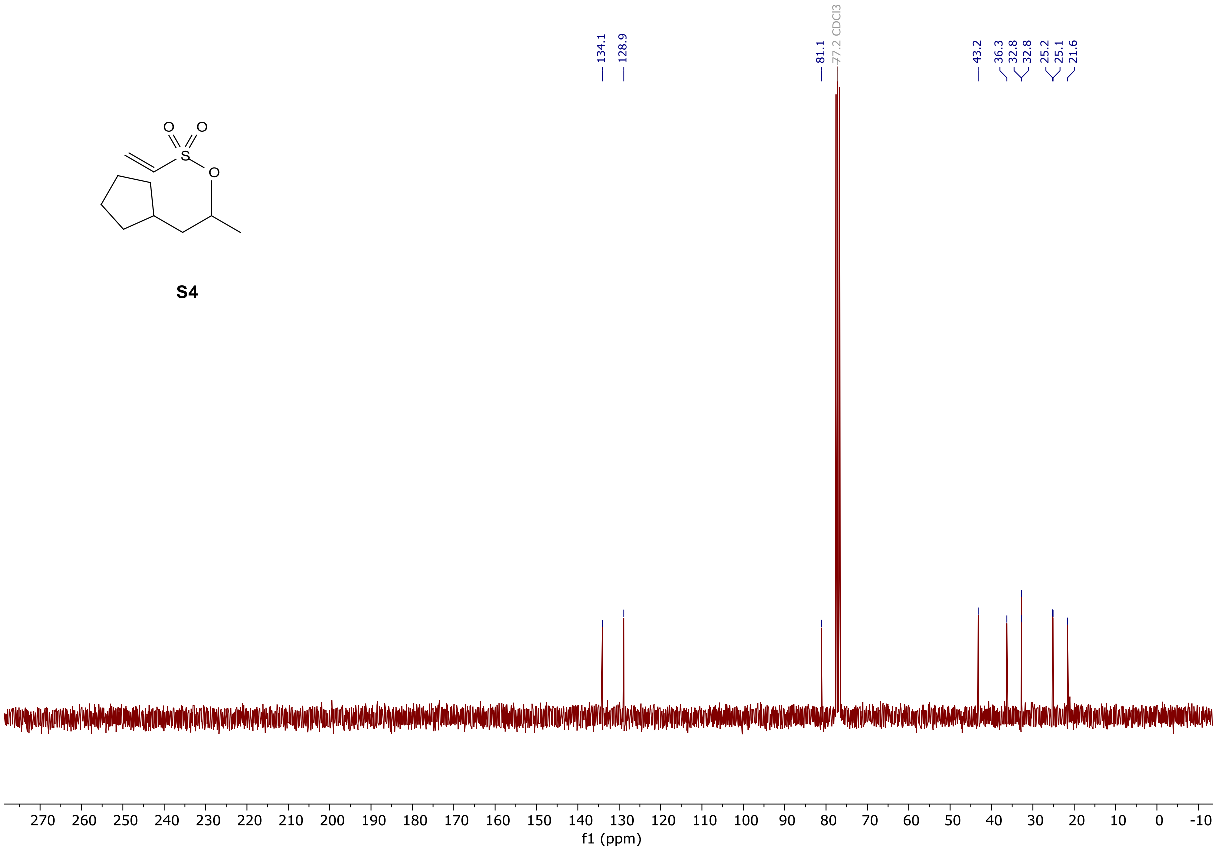
134.1 128.9
81.1 77.2 CDCl3
43.2 36.3 32.8 32.8 25.2 25.1 21.6
S4
270 260 250 240 230 220 210 200 190 180 170 160 150 140 130 120 110 100 90 80 70 60 50 40 30 20 10 0 -10
f1 (ppm)

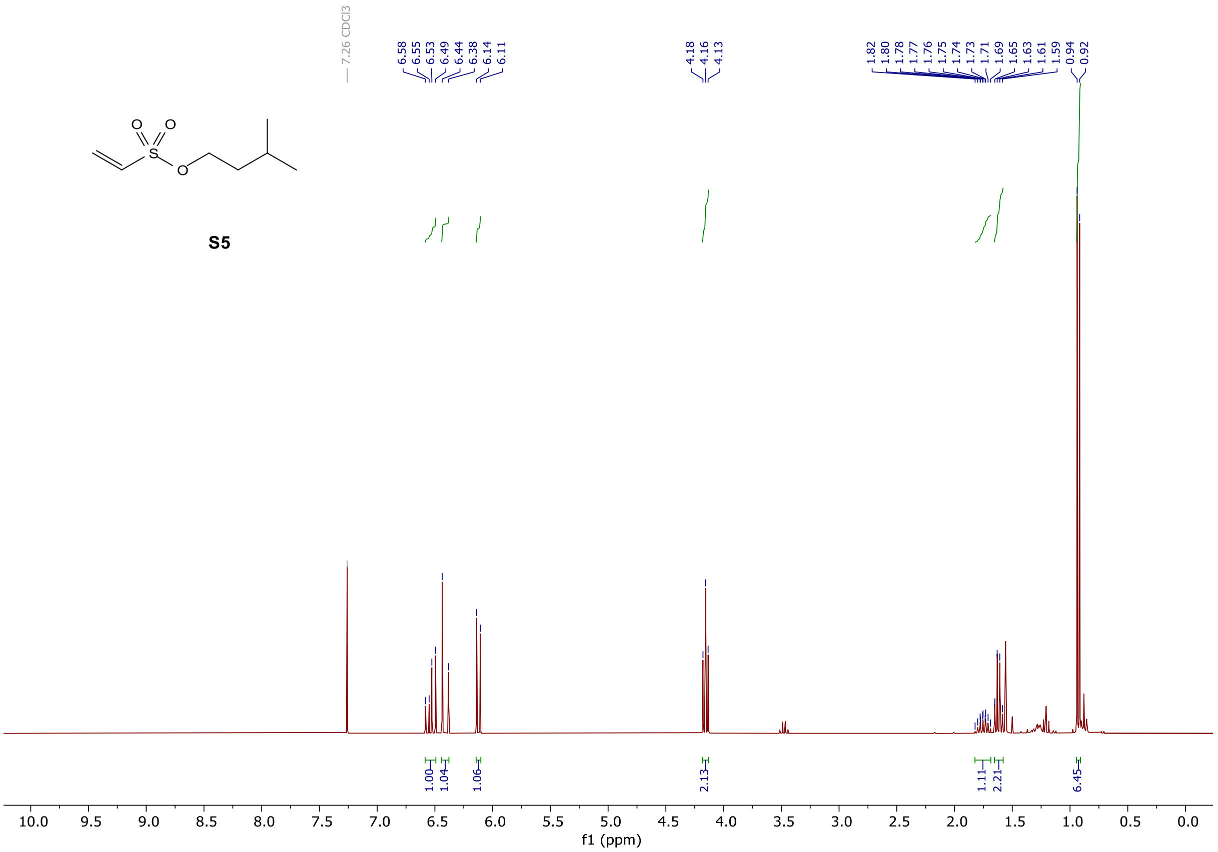
7.26 CDCl3
6.58
6.55
6.53
6.49
6.44
6.38
6.14
6.11
4.18
4.16
4.13
1.82
1.80
1.78
1.77
1.76
1.75
1.74
1.73
1.71
1.69
1.65
1.63
1.61
1.59
0.94
0.92
S5
1.00
1.04
1.06
2.13
1.11
2.21
6.45
f1 (ppm)

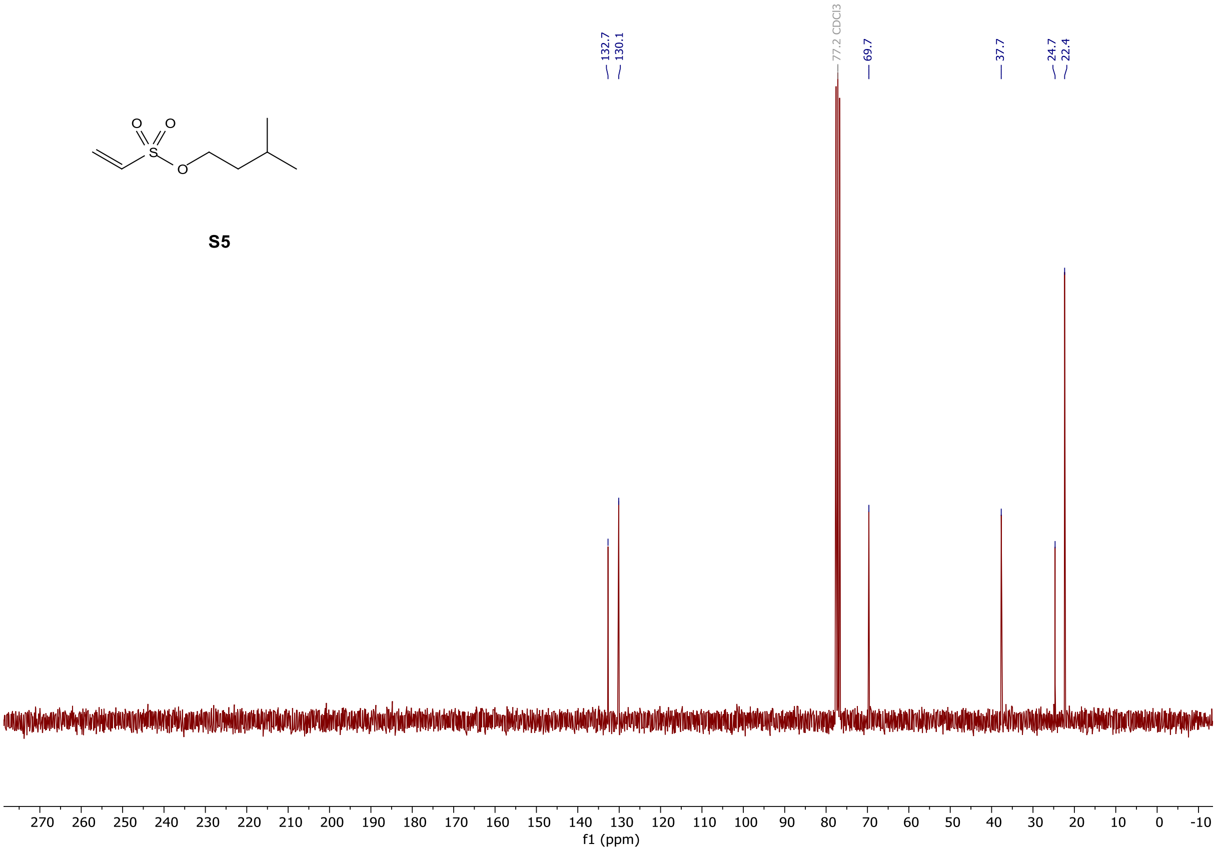
132.7
130.1
77.2 CDCl3
69.7
37.7
24.7
22.4
S5
f1 (ppm)

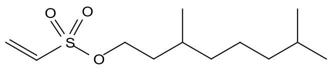

S6

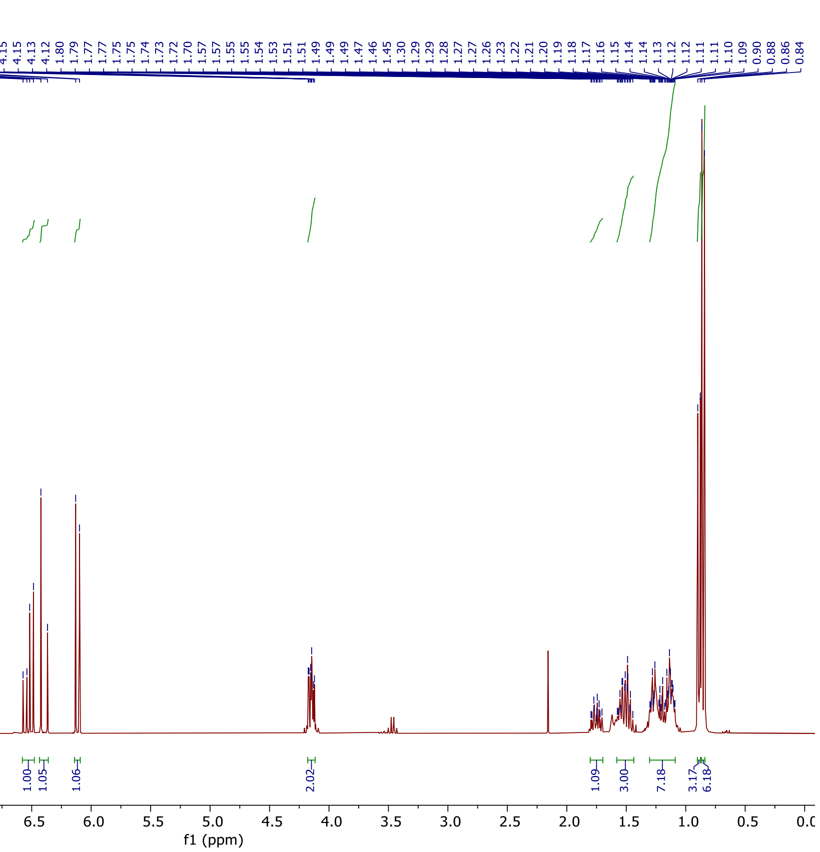

7.26 CDCl3
6.57
6.54
6.52
6.49
6.42
6.37
6.13
6.10
4.17
4.17
4.15
4.15
4.15
4.13
4.12
1.80
1.79
1.77
1.77
1.75
1.75
1.74
1.73
1.72
1.70
1.57
1.57
1.55
1.55
1.54
1.53
1.51
1.51
1.49
1.49
1.47
1.46
1.45
1.30
1.29
1.29
1.28
1.27
1.27
1.26
1.23
1.22
1.21
1.20
1.19
1.18
1.17
1.16
1.15
1.14
1.14
1.13
1.12
1.12
1.11
1.11
1.10
1.09
0.90
0.88
0.86
0.84
1.00
1.05
1.06
2.02
1.09
3.00
7.18
3.17
6.18
10.0 9.5 9.0 8.5 8.0 7.5 7.0 6.5 6.0 5.5 5.0 4.5 4.0 3.5 3.0 2.5 2.0 1.5 1.0 0.5 0.0
f1 (ppm)

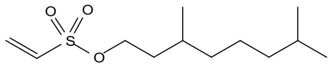

S6

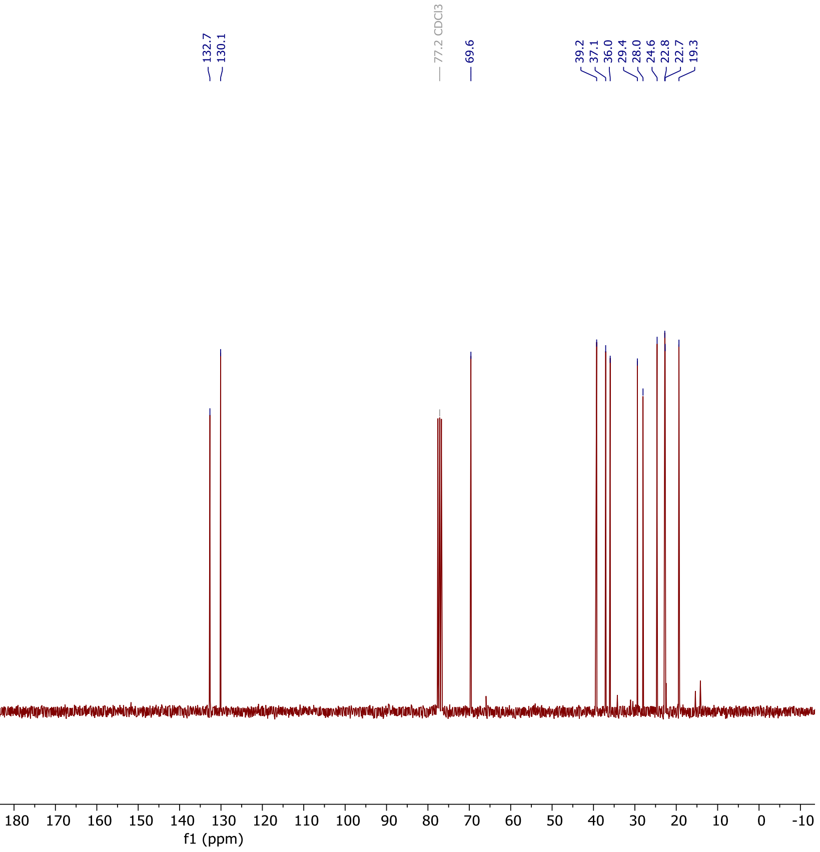

132.7
130.1
77.2 CDCl3
69.6
39.2
37.1
36.0
29.4
28.0
24.6
22.8
22.7
19.3
270 260 250 240 230 220 210 200 190 180 170 160 150 140 130 120 110 100 90 80 70 60 50 40 30 20 10 0 -10
f1 (ppm)

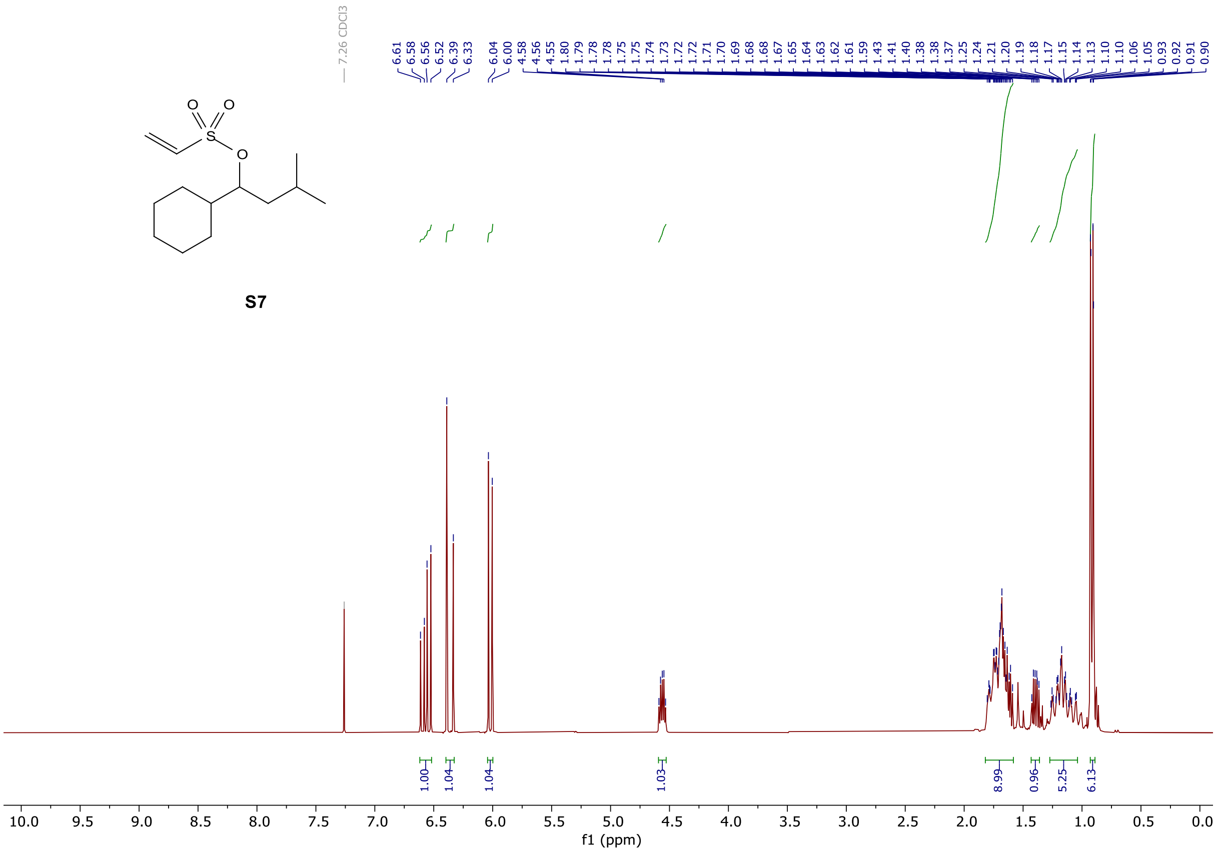

S7

7.26 CDCl3
6.61 6.58 6.56 6.52 6.39 6.33 6.04 6.00 4.58 4.56 4.55 1.80 1.79 1.78 1.75 1.75 1.74 1.73 1.72 1.72 1.71 1.70 1.69 1.68 1.68 1.67 1.65 1.64 1.63 1.62 1.61 1.59 1.43 1.41 1.40 1.38 1.38 1.37 1.25 1.24 1.21 1.20 1.19 1.18 1.17 1.15 1.14 1.13 1.10 1.10 1.06 1.05 0.93 0.92 0.91 0.90
1.00 1.04 1.04 1.03 8.99 0.96 5.25 6.13
10.0 9.5 9.0 8.5 8.0 7.5 7.0 6.5 6.0 5.5 5.0 4.5 4.0 3.5 3.0 2.5 2.0 1.5 1.0 0.5 0.0
f1 (ppm)

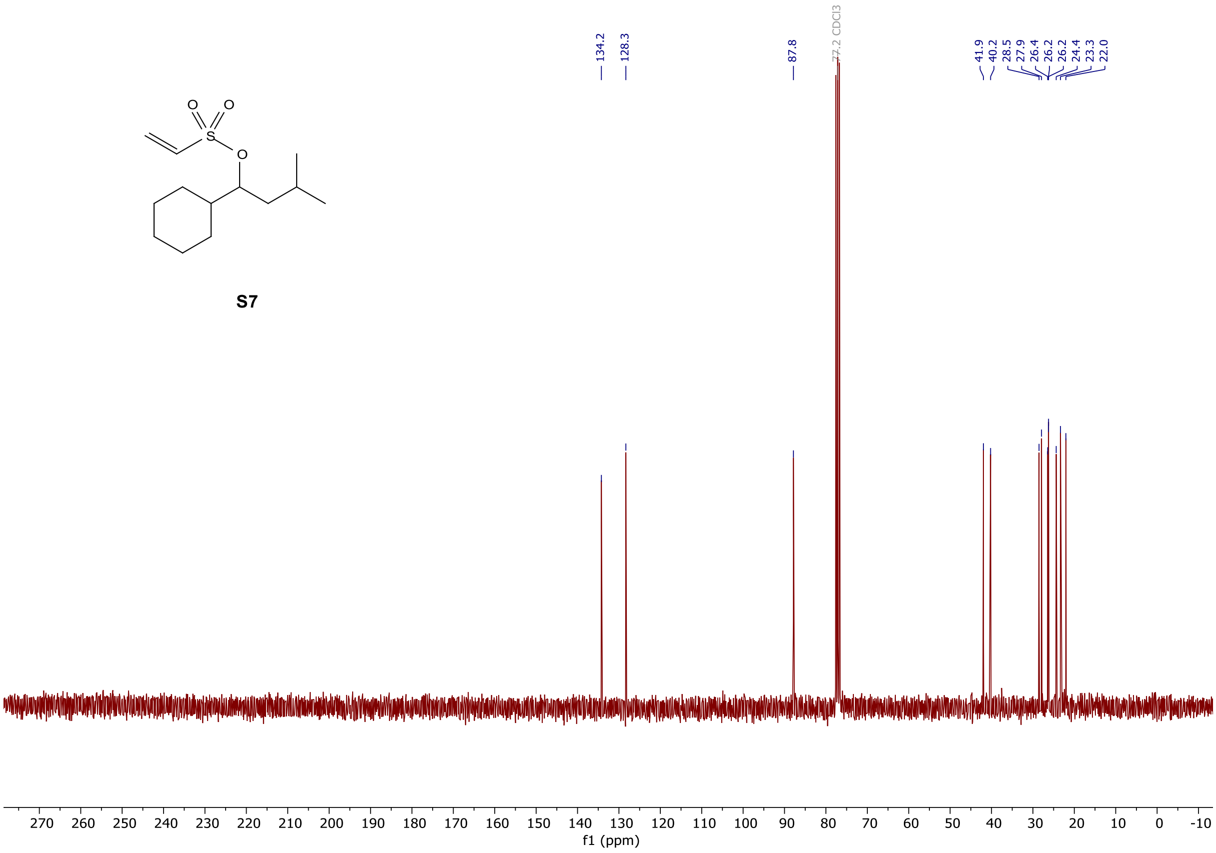

S7

134.2
128.3
87.8
77.2 CDCl3
41.9 40.2 28.5 27.9 26.4 26.2 26.2 24.4 23.3 22.0
270 260 250 240 230 220 210 200 190 180 170 160 150 140 130 120 110 100 90 80 70 60 50 40 30 20 10 0 -10
f1 (ppm)

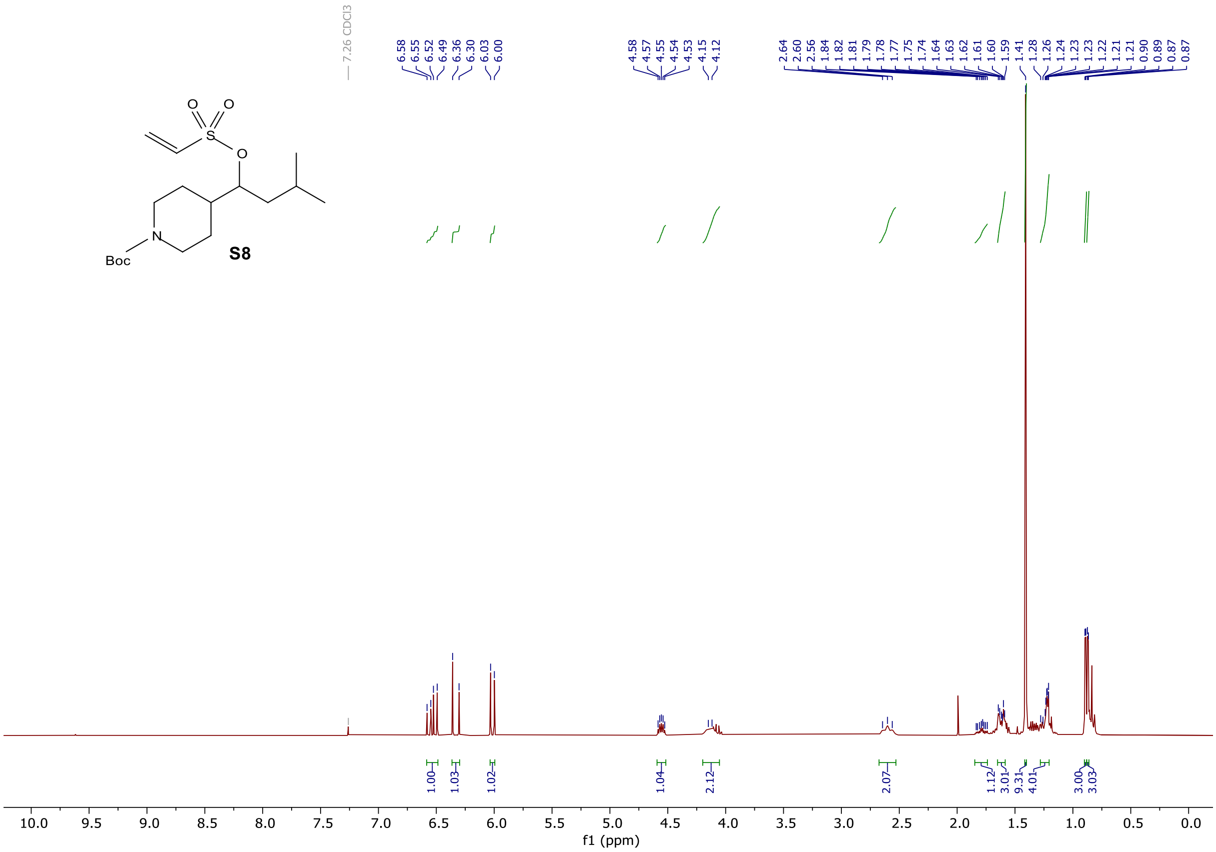
7.26 CDCl3
6.58
6.55
6.52
6.49
6.36
6.30
6.03
6.00
4.58
4.57
4.55
4.54
4.53
4.15
4.12
2.64
2.60
2.56
1.84
1.82
1.81
1.79
1.78
1.77
1.75
1.74
1.64
1.63
1.62
1.61
1.60
1.59
1.41
1.28
1.26
1.24
1.23
1.23
1.22
1.21
1.21
0.90
0.89
0.87
0.87
O
O
S
O
N
Boc
S8
1.00
1.03
1.02
1.04
2.12
2.07
1.12
3.01
9.31
4.01
3.00
3.03
10.0 9.5 9.0 8.5 8.0 7.5 7.0 6.5 6.0 5.5 5.0 4.5 4.0 3.5 3.0 2.5 2.0 1.5 1.0 0.5 0.0
f1 (ppm)

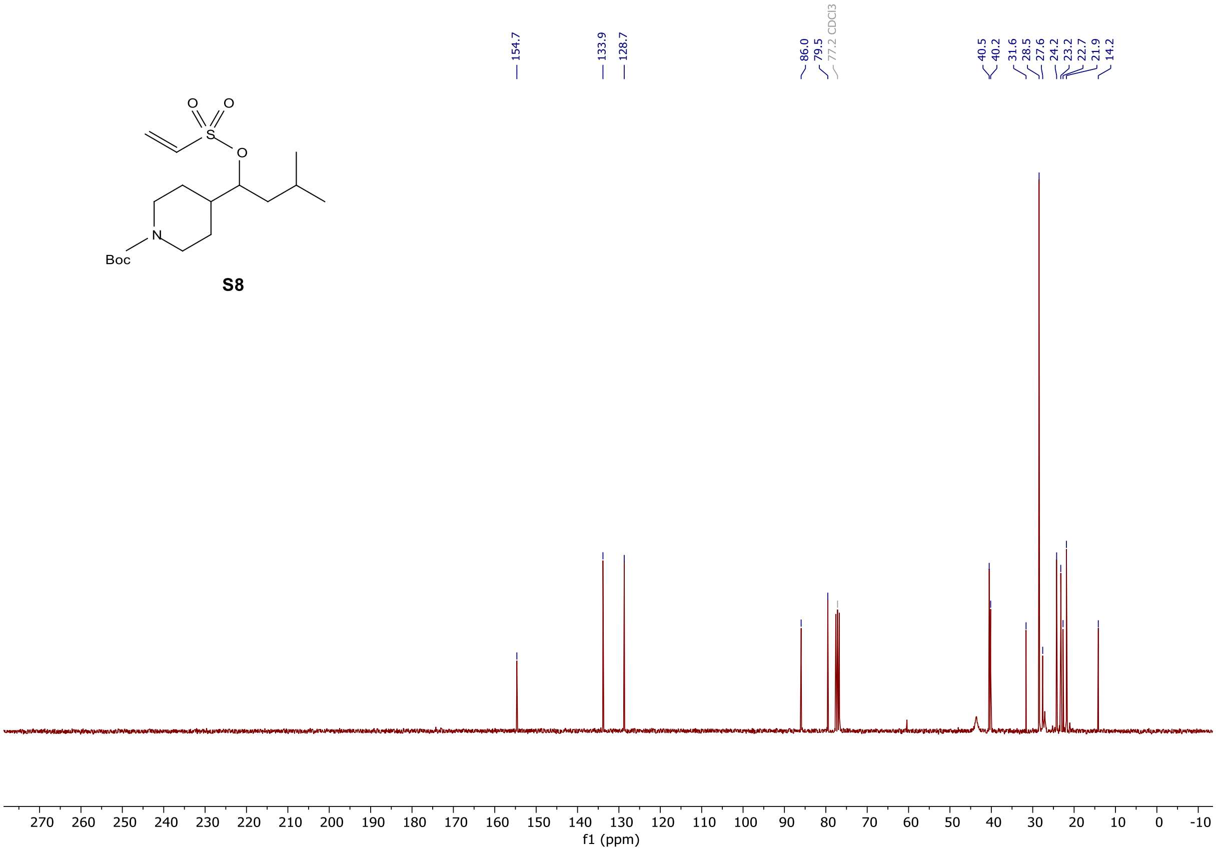
154.7
133.9
128.7
86.0
79.5
77.2 CDCl3
40.5
40.2
31.6
28.5
27.6
24.2
23.2
22.7
21.9
14.2
O
O
S
O
N
Boc
S8
270 260 250 240 230 220 210 200 190 180 170 160 150 140 130 120 110 100 90 80 70 60 50 40 30 20 10 0 -10
f1 (ppm)

S9

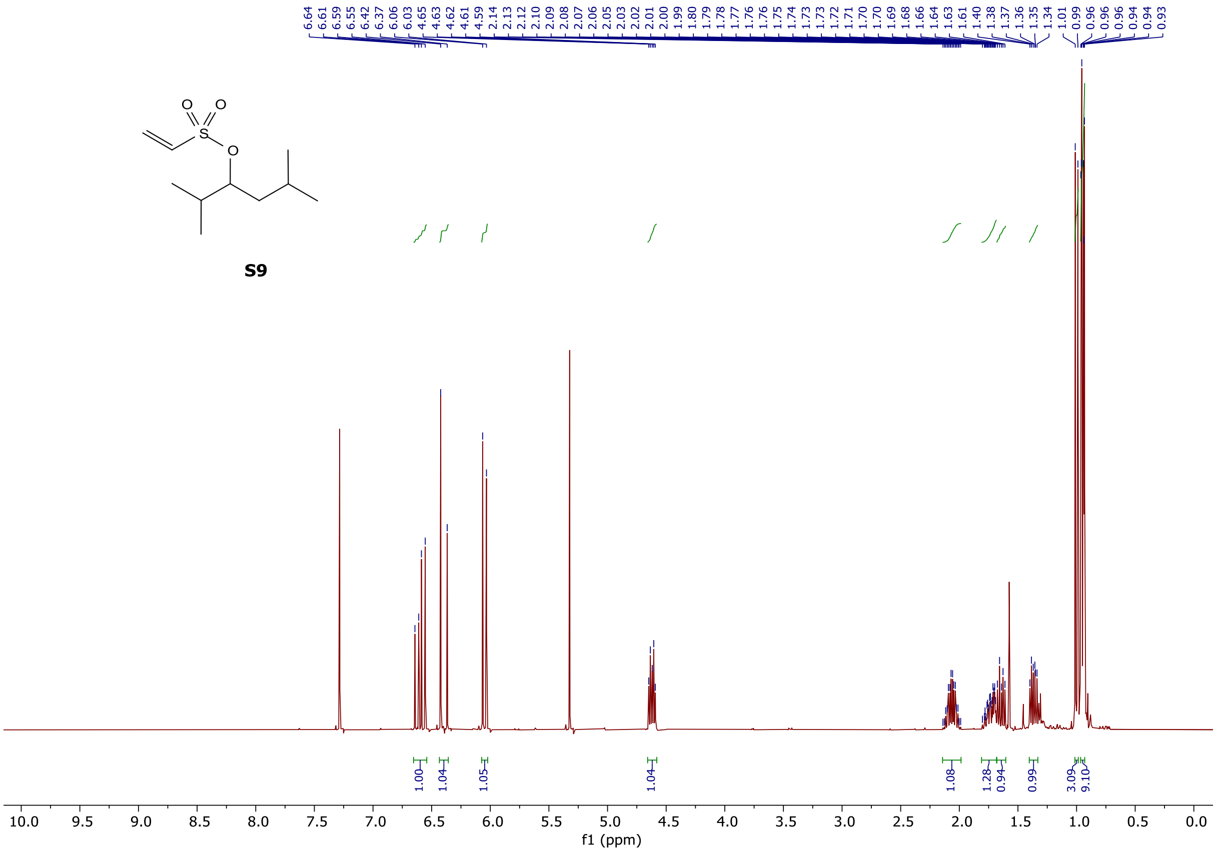
6.64 6.61 6.59 6.55 6.42 6.37 6.06 6.03 4.65 4.63 4.62 4.61 4.59 2.14 2.13 2.12 2.10 2.09 2.08 2.07 2.06 2.05 2.03 2.02 2.01 2.00 1.99 1.80 1.79 1.78 1.77 1.76 1.76 1.75 1.74 1.73 1.73 1.72 1.71 1.70 1.70 1.69 1.68 1.66 1.64 1.63 1.61 1.40 1.38 1.37 1.36 1.35 1.34 1.01 0.99 0.96 0.96 0.96 0.94 0.94 0.93
1.00 1.04 1.05 1.04 1.08 1.28 0.94 0.99 3.09 9.10
f1 (ppm)

S9

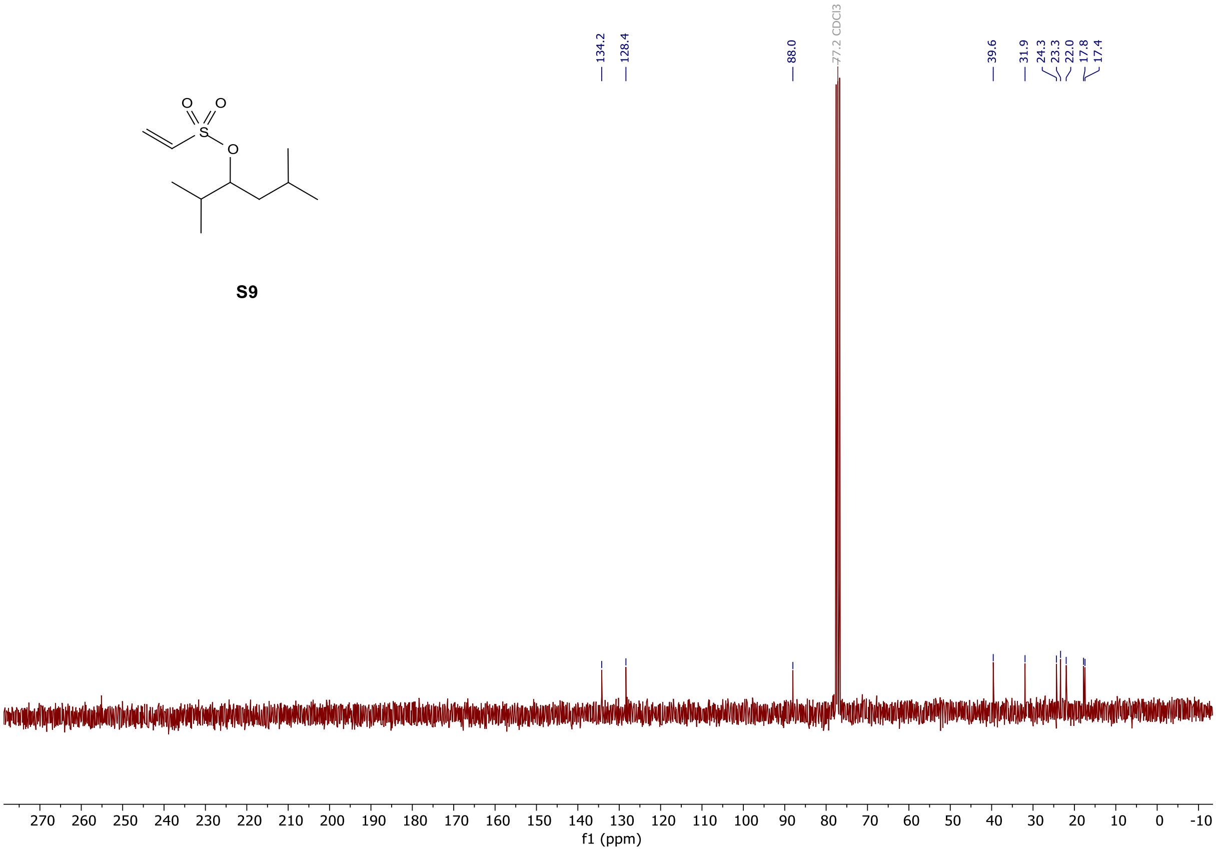
134.2
128.4
88.0
77.2 CDCl3
39.6
31.9
24.3
23.3
22.0
17.8
17.4
f1 (ppm)

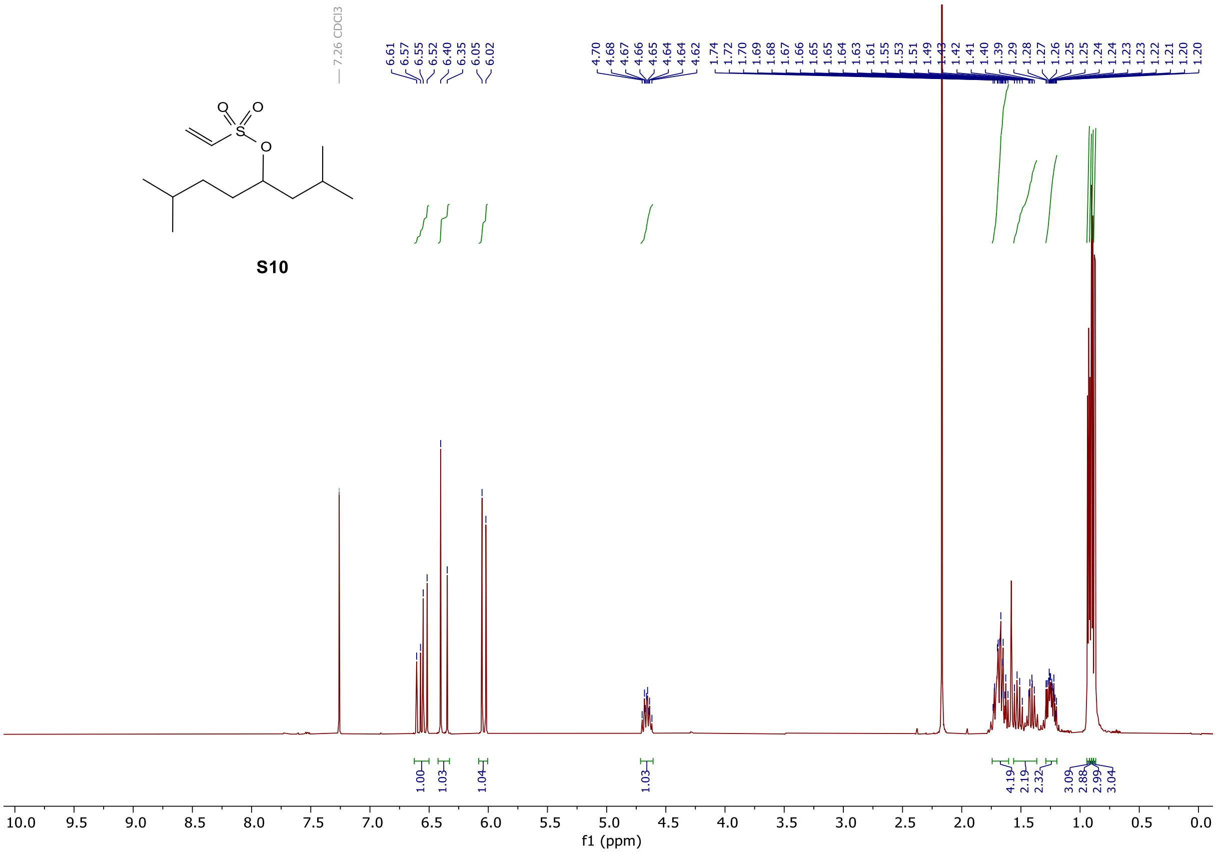
S10
7.26 CDCl3
f1 (ppm)

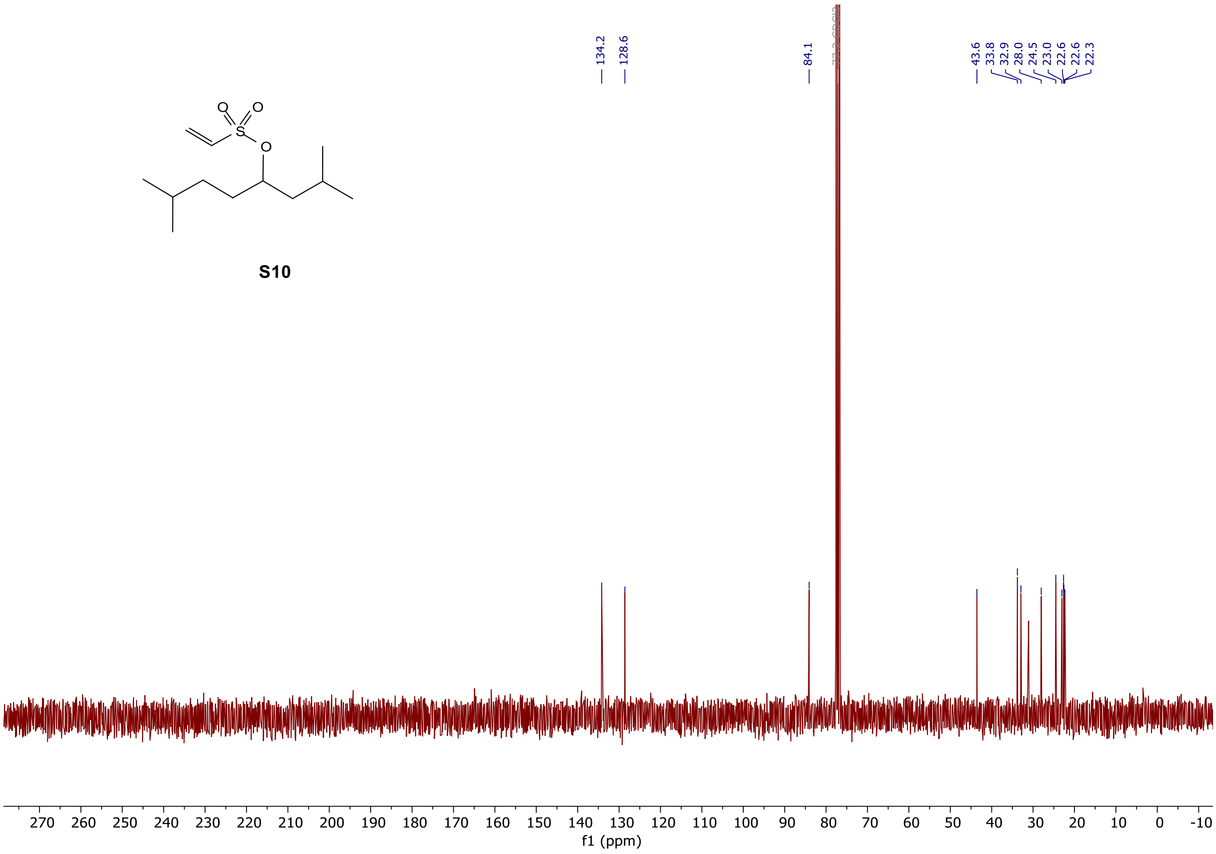
S10
134.2
128.6
84.1
43.6
33.8
32.9
28.0
24.5
23.0
22.6
22.6
22.3
f1 (ppm)

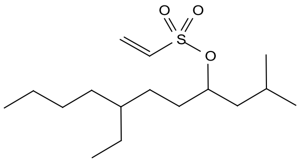

S11

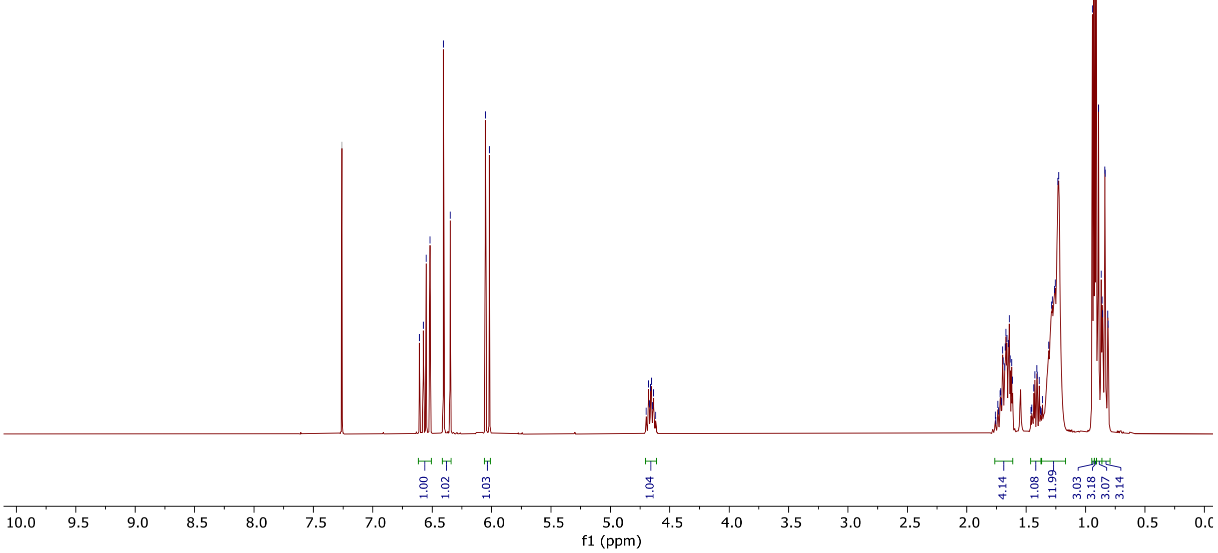
7.26 CDCl3
6.61 6.57 6.55 6.52 6.40 6.35 6.05 6.02 4.70 4.68 4.67 4.66 4.65 4.64 4.64 4.62 1.76 1.76 1.74 1.73 1.72 1.71 1.71 1.70 1.69 1.68 1.67 1.66 1.65 1.64 1.63 1.62 1.46 1.45 1.44 1.43 1.41 1.41 1.39 1.38 1.37 1.36 1.31 1.29 1.28 1.26 1.25 1.23 1.23 0.94 0.93 0.92 0.91 0.89 0.87 0.86 0.84 0.83
1.00 1.02 1.03 1.04 4.14 1.08 11.99 3.03 3.18 3.07 3.14
10.0 9.5 9.0 8.5 8.0 7.5 7.0 6.5 6.0 5.5 5.0 4.5 4.0 3.5 3.0 2.5 2.0 1.5 1.0 0.5 0.0
f1 (ppm)

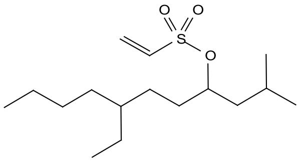

S11

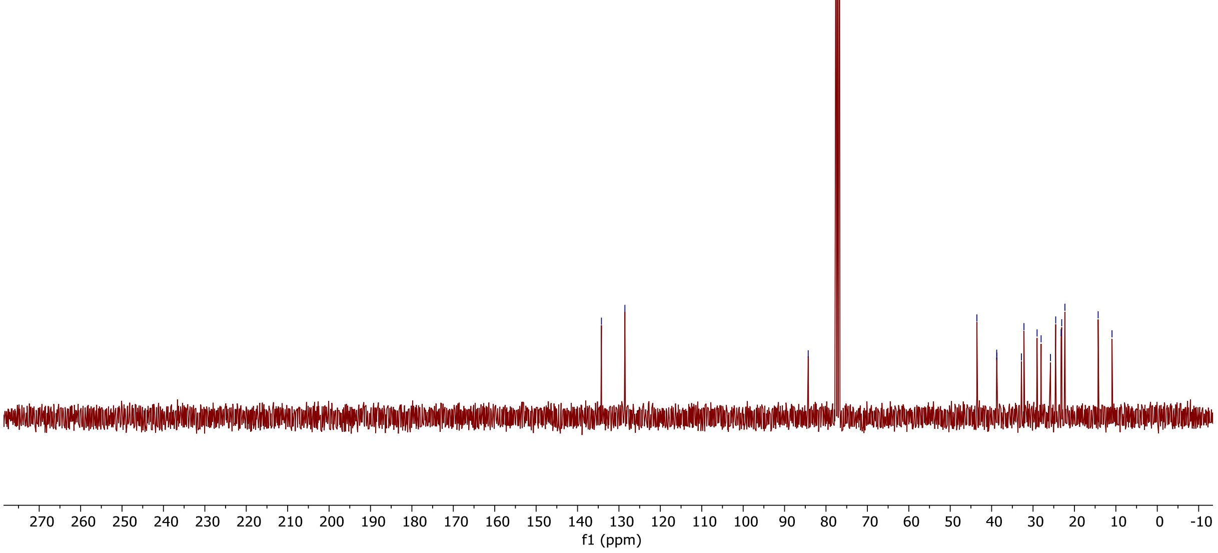
134.2 128.6 84.3 77.2 CDCl3
43.6 38.8 38.7 32.8 32.2 29.0 28.1 25.8 24.5 23.2 23.1 22.3 14.3 10.9
270 260 250 240 230 220 210 200 190 180 170 160 150 140 130 120 110 100 90 80 70 60 50 40 30 20 10 0 -10
f1 (ppm)

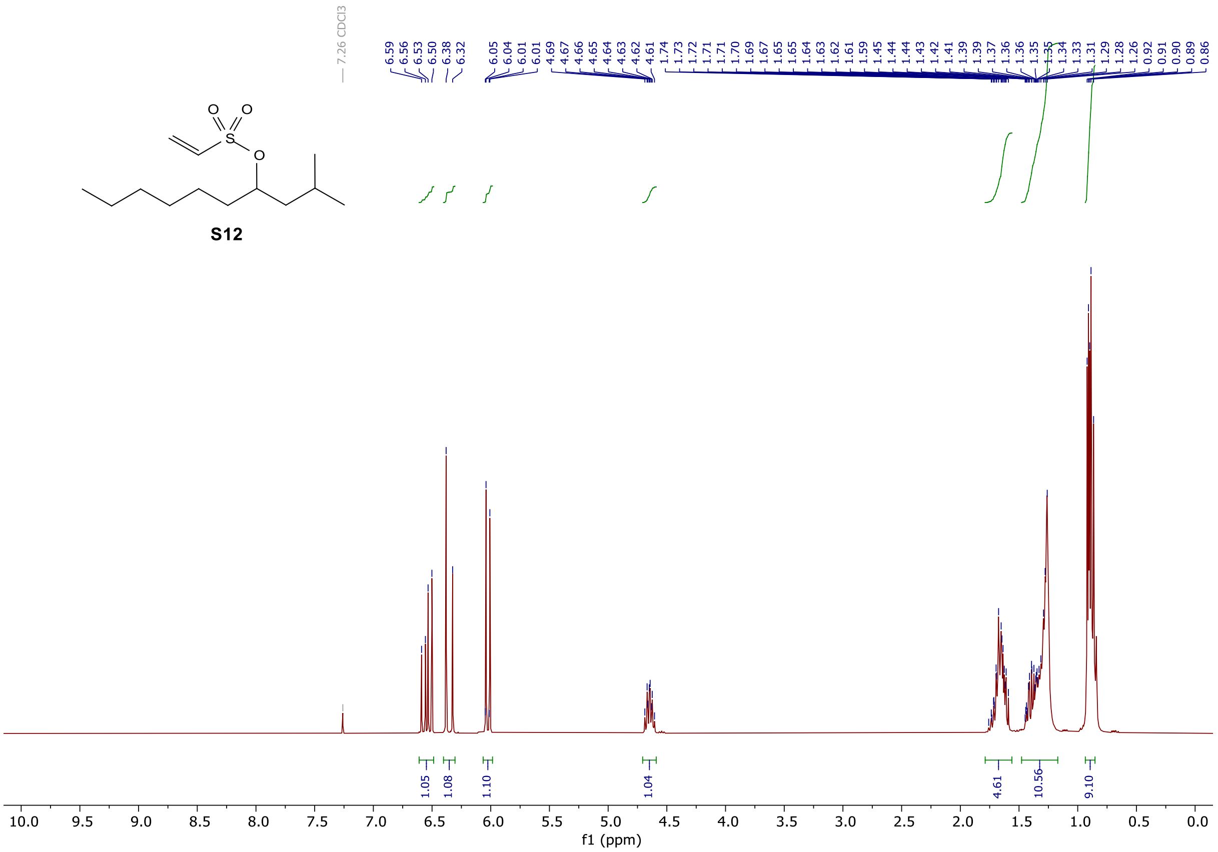
7.26 CDCl3
6.59 6.56 6.53 6.50 6.38 6.32
6.05 6.04 6.01 6.01
4.69 4.67 4.66 4.65 4.64 4.63 4.62 4.61
1.74 1.73 1.72 1.71 1.71 1.70 1.69 1.67 1.65 1.65 1.64 1.63 1.62 1.61 1.59 1.45 1.44 1.44 1.43 1.42 1.41 1.39 1.37 1.36 1.36 1.35 1.35 1.34 1.33 1.31 1.29 1.28 1.26 0.92 0.91 0.90 0.89 0.86
1.05 1.08 1.10 1.04 4.61 10.56 9.10
10.0 9.5 9.0 8.5 8.0 7.5 7.0 6.5 6.0 5.5 5.0 4.5 4.0 3.5 3.0 2.5 2.0 1.5 1.0 0.5 0.0
f1 (ppm)
S12

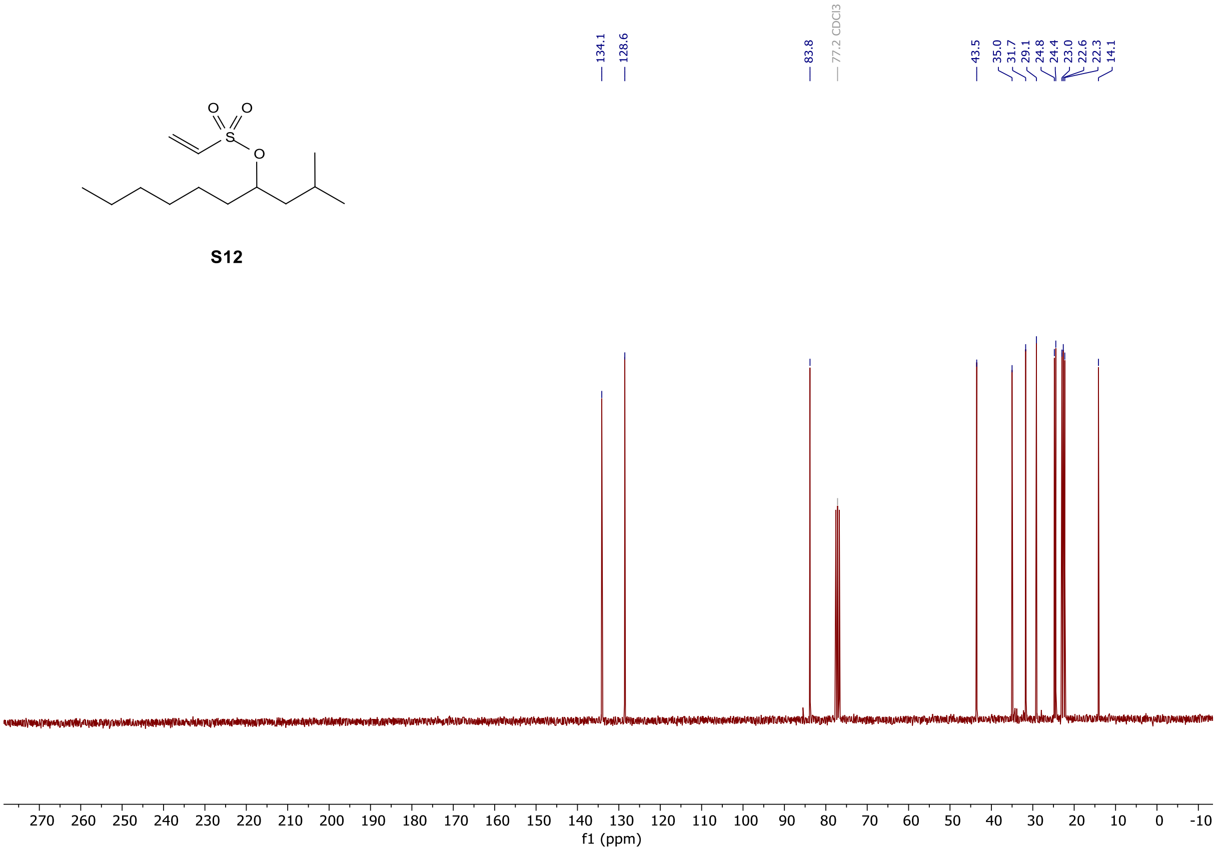
134.1 128.6
83.8 77.2 CDCl3
43.5 35.0 31.7 29.1 24.8 24.4 23.0 22.6 22.3 14.1
270 260 250 240 230 220 210 200 190 180 170 160 150 140 130 120 110 100 90 80 70 60 50 40 30 20 10 0 -10
f1 (ppm)
S12

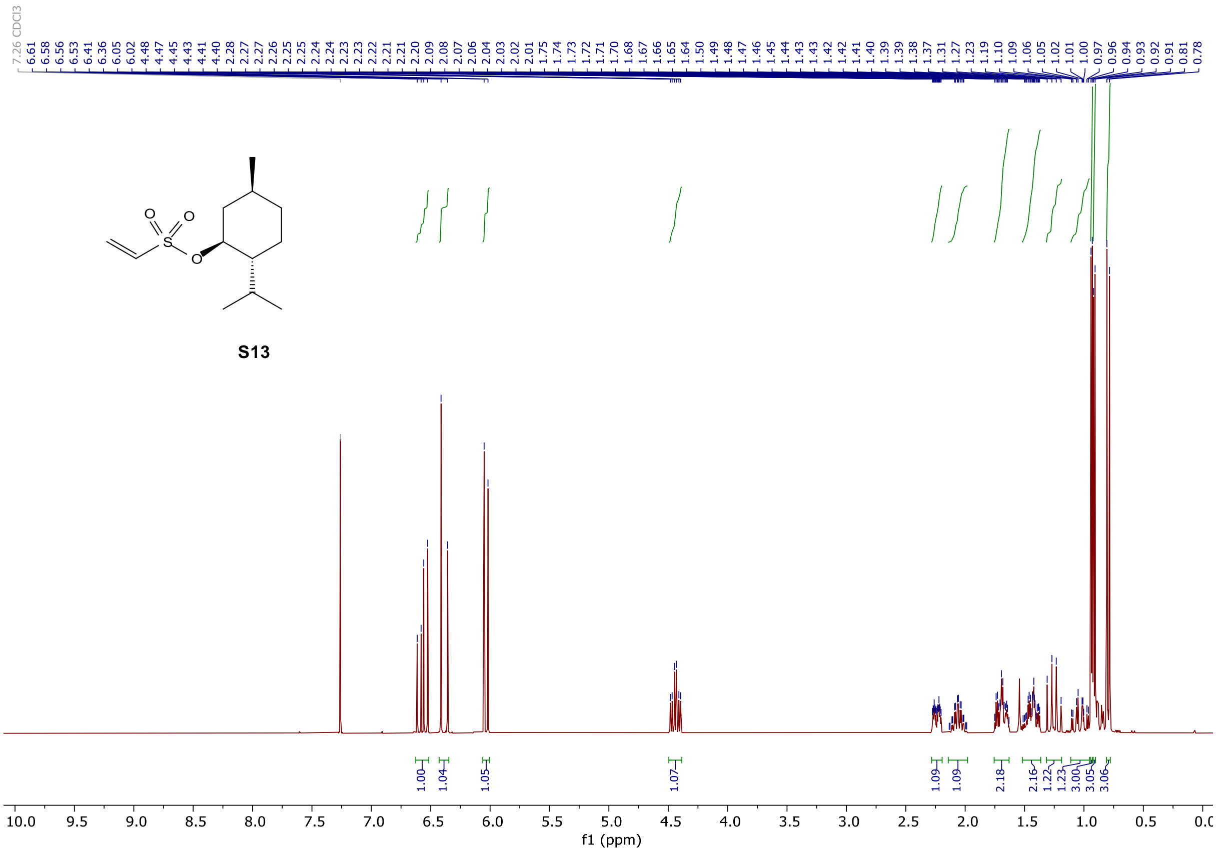
S13
f1 (ppm)
1.00
1.04
1.05
1.07
1.09
1.09
2.18
2.16
1.22
1.23
3.00
3.05
3.06

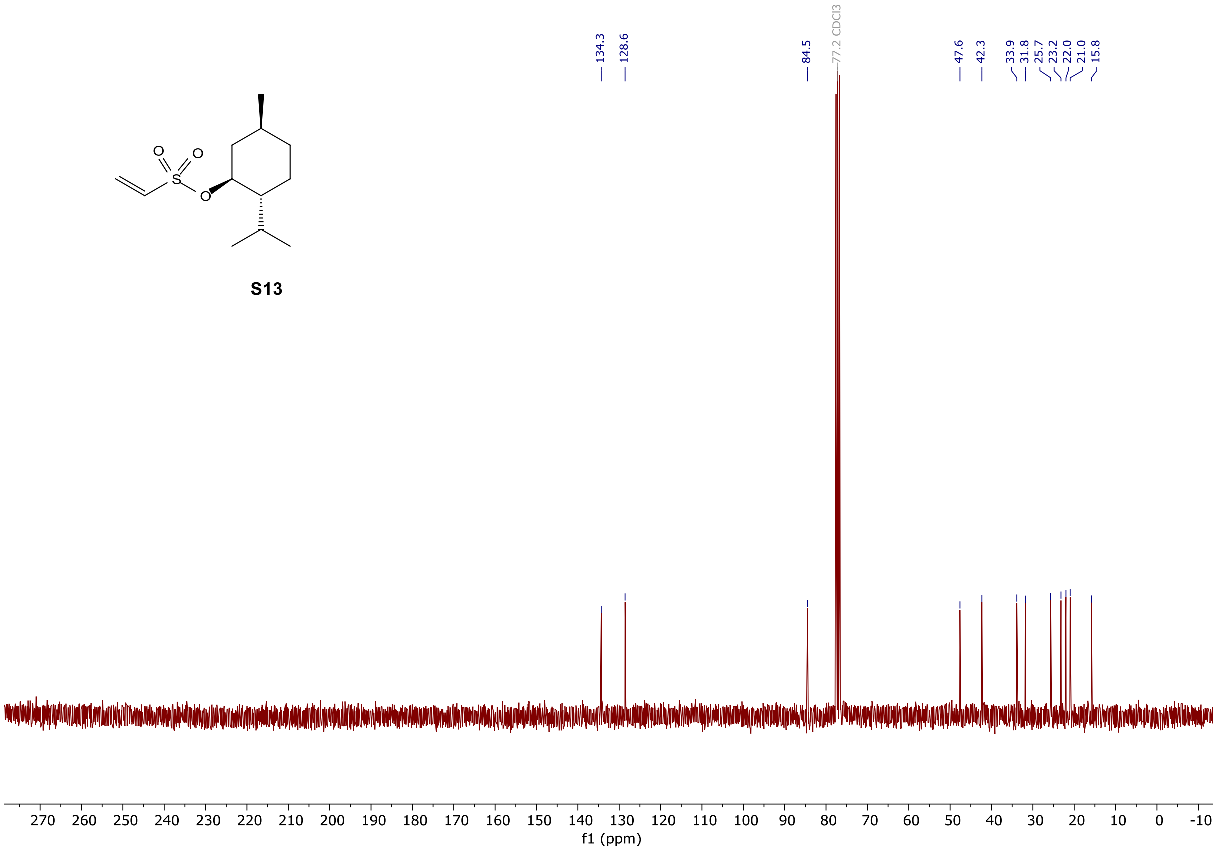
S13
134.3
128.6
84.5
77.2 CDCl3
47.6
42.3
33.9
31.8
25.7
23.2
22.0
21.0
15.8
f1 (ppm)

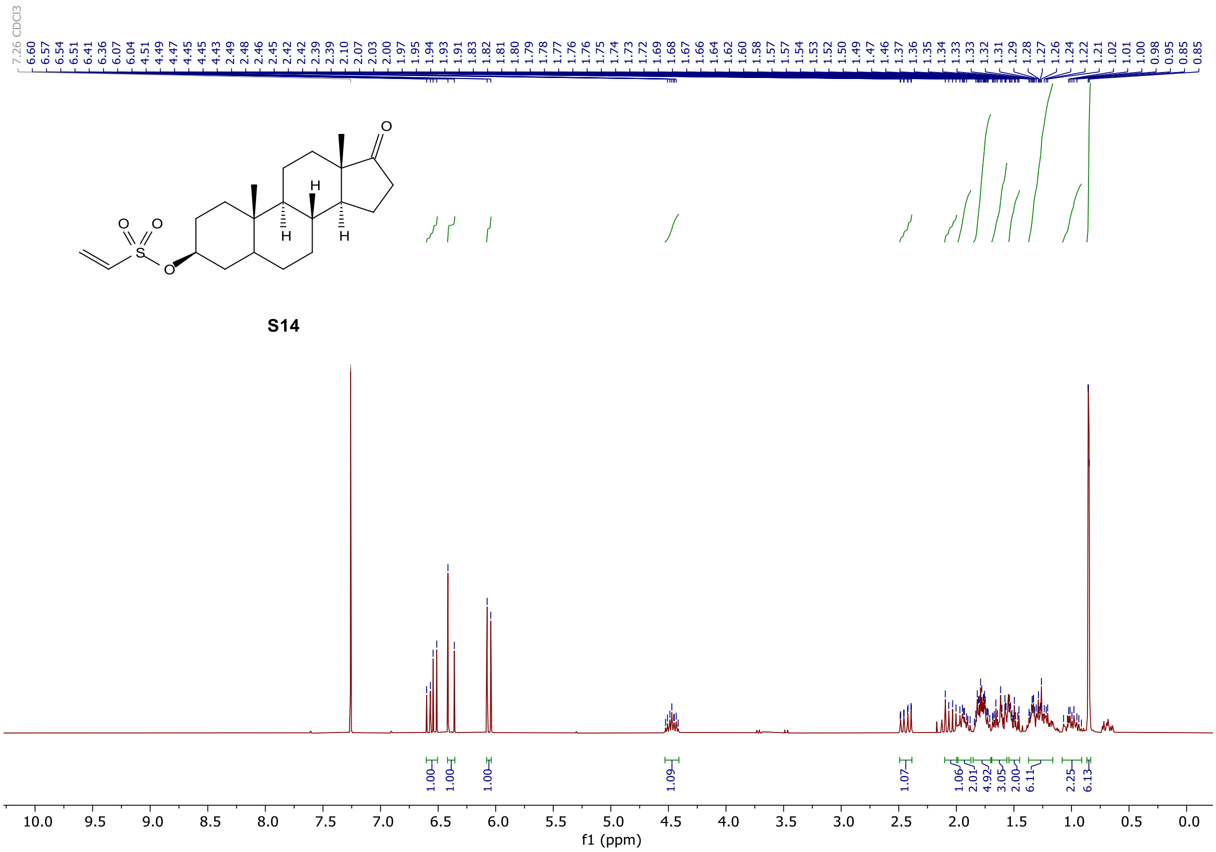
7.26 CDCl3
6.60
6.57
6.54
6.51
6.41
6.36
6.07
6.04
4.51
4.49
4.47
4.45
4.45
4.43
2.49
2.48
2.46
2.45
2.42
2.42
2.39
2.39
2.10
2.07
2.03
2.00
1.97
1.95
1.94
1.93
1.91
1.83
1.82
1.81
1.80
1.79
1.78
1.77
1.76
1.76
1.75
1.74
1.73
1.72
1.69
1.68
1.67
1.66
1.64
1.62
1.60
1.58
1.57
1.57
1.54
1.53
1.52
1.50
1.49
1.47
1.46
1.37
1.36
1.35
1.34
1.33
1.33
1.32
1.31
1.29
1.28
1.27
1.26
1.24
1.22
1.21
1.02
1.01
1.00
0.98
0.95
0.85
0.85
1.00
1.00
1.00
1.09
1.07
1.06
2.01
4.92
3.05
2.00
6.11
2.25
6.13
10.0 9.5 9.0 8.5 8.0 7.5 7.0 6.5 6.0 5.5 5.0 4.5 4.0 3.5 3.0 2.5 2.0 1.5 1.0 0.5 0.0
f1 (ppm)

S14

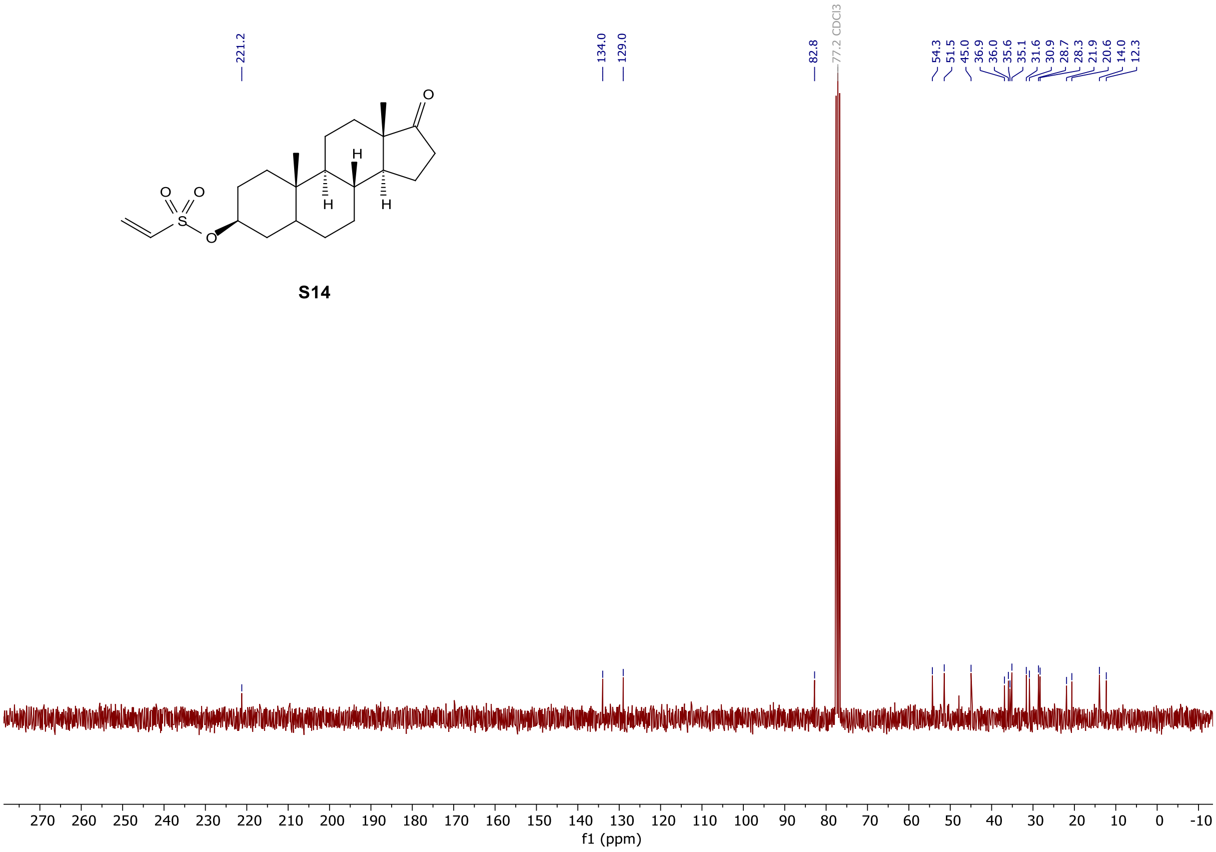
221.2
134.0
129.0
82.8
77.2 CDCl3
54.3
51.5
45.0
36.9
36.0
35.6
35.1
31.6
30.9
28.7
28.3
21.9
20.6
14.0
12.3
270 260 250 240 230 220 210 200 190 180 170 160 150 140 130 120 110 100 90 80 70 60 50 40 30 20 10 0 -10
f1 (ppm)

S14

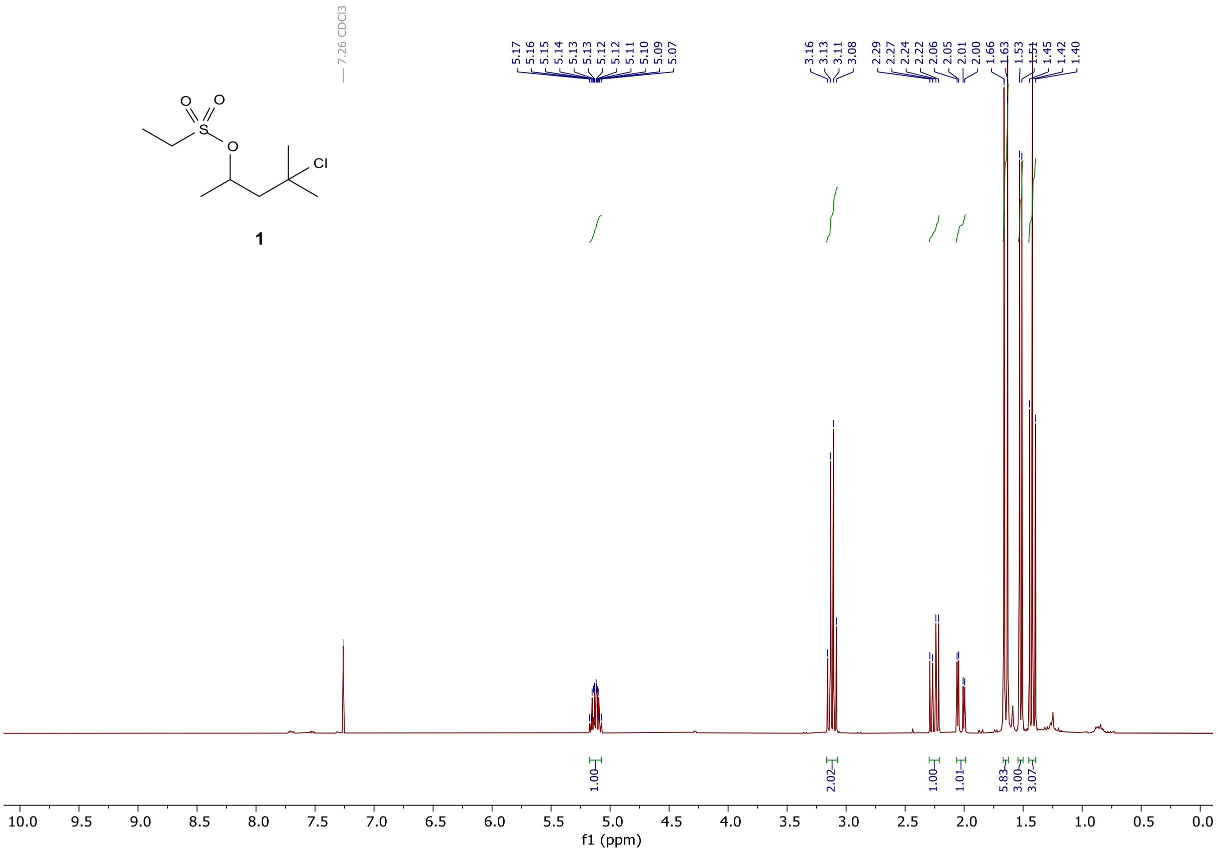
7.26 CDCl3
5.17 5.16 5.15 5.14 5.13 5.13 5.12 5.12 5.11 5.10 5.09 5.07
3.16 3.13 3.11 3.08 2.29 2.27 2.24 2.22 2.06 2.05 2.01 2.00 1.66 1.63 1.53 1.51 1.45 1.42 1.40
O
O
S
O
Cl
1
1.00
2.02
1.00
1.01
5.83
3.00
3.07
10.0 9.5 9.0 8.5 8.0 7.5 7.0 6.5 6.0 5.5 5.0 4.5 4.0 3.5 3.0 2.5 2.0 1.5 1.0 0.5 0.0
f1 (ppm)

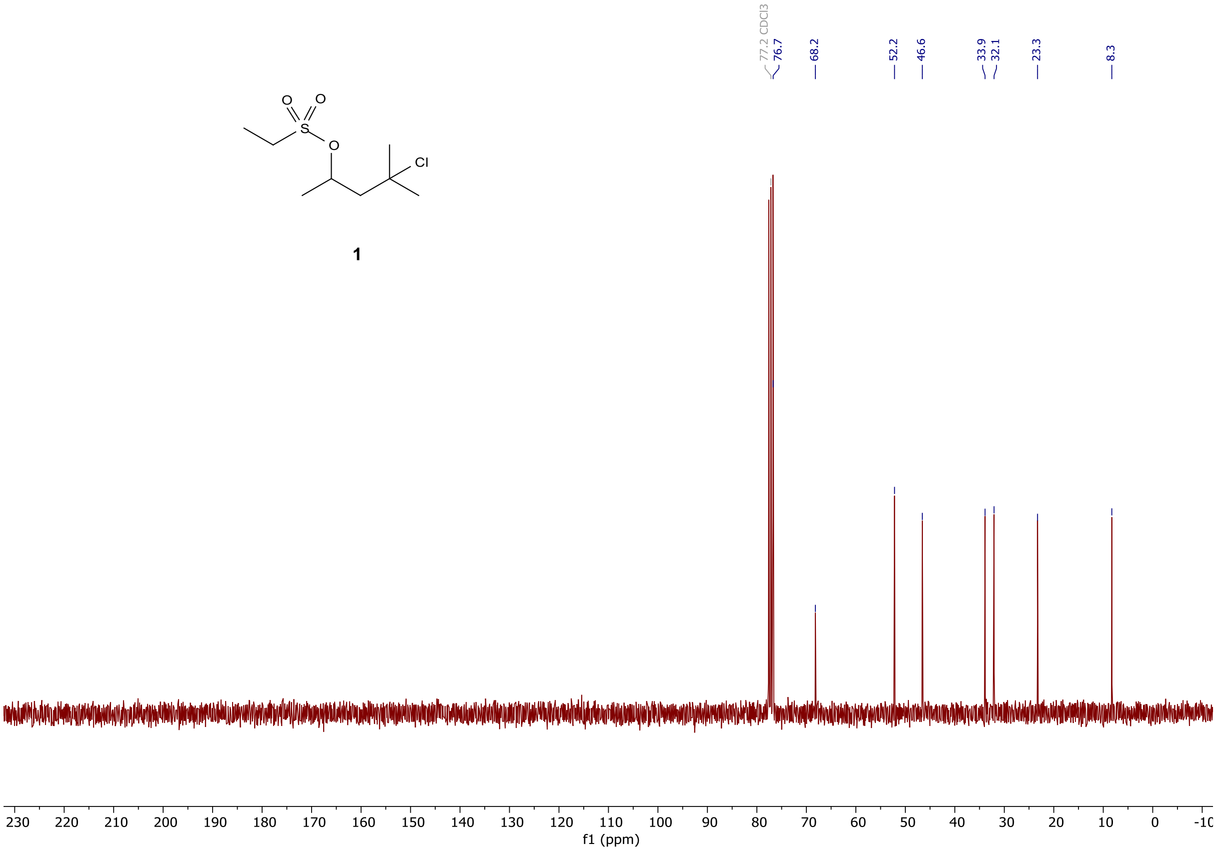
77.2 CDCl3
76.7
68.2
52.2
46.6
33.9
32.1
23.3
8.3
O
O
S
O
Cl
1
230 220 210 200 190 180 170 160 150 140 130 120 110 100 90 80 70 60 50 40 30 20 10 0 -10
f1 (ppm)

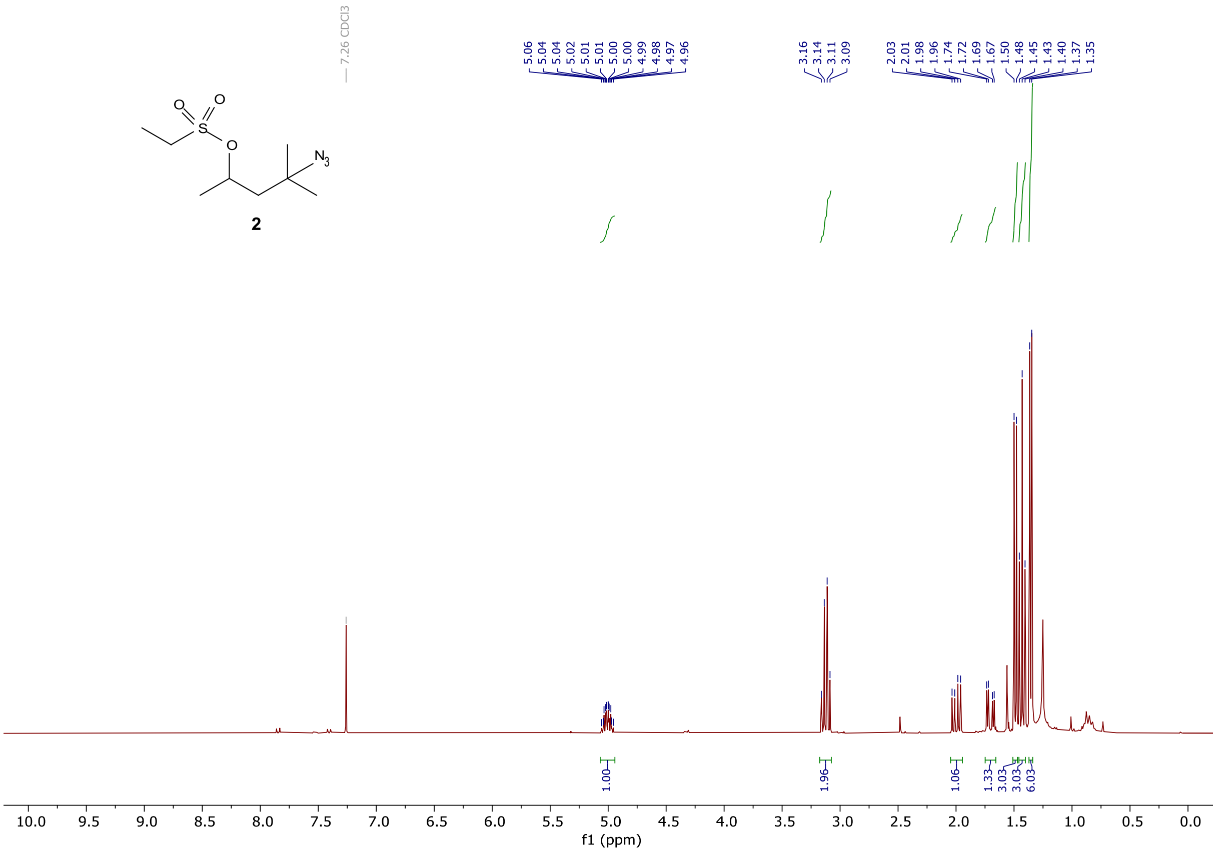

7.26 CDCl3
5.06
5.04
5.04
5.02
5.01
5.01
5.00
5.00
4.99
4.98
4.97
4.96
3.16
3.14
3.11
3.09
2.03
2.01
1.98
1.96
1.74
1.72
1.69
1.67
1.50
1.48
1.45
1.43
1.40
1.37
1.35
O
O
S
O
N3
2
1.00
1.96
1.06
1.33
3.03
3.03
6.03
10.0 9.5 9.0 8.5 8.0 7.5 7.0 6.5 6.0 5.5 5.0 4.5 4.0 3.5 3.0 2.5 2.0 1.5 1.0 0.5 0.0
f1 (ppm)

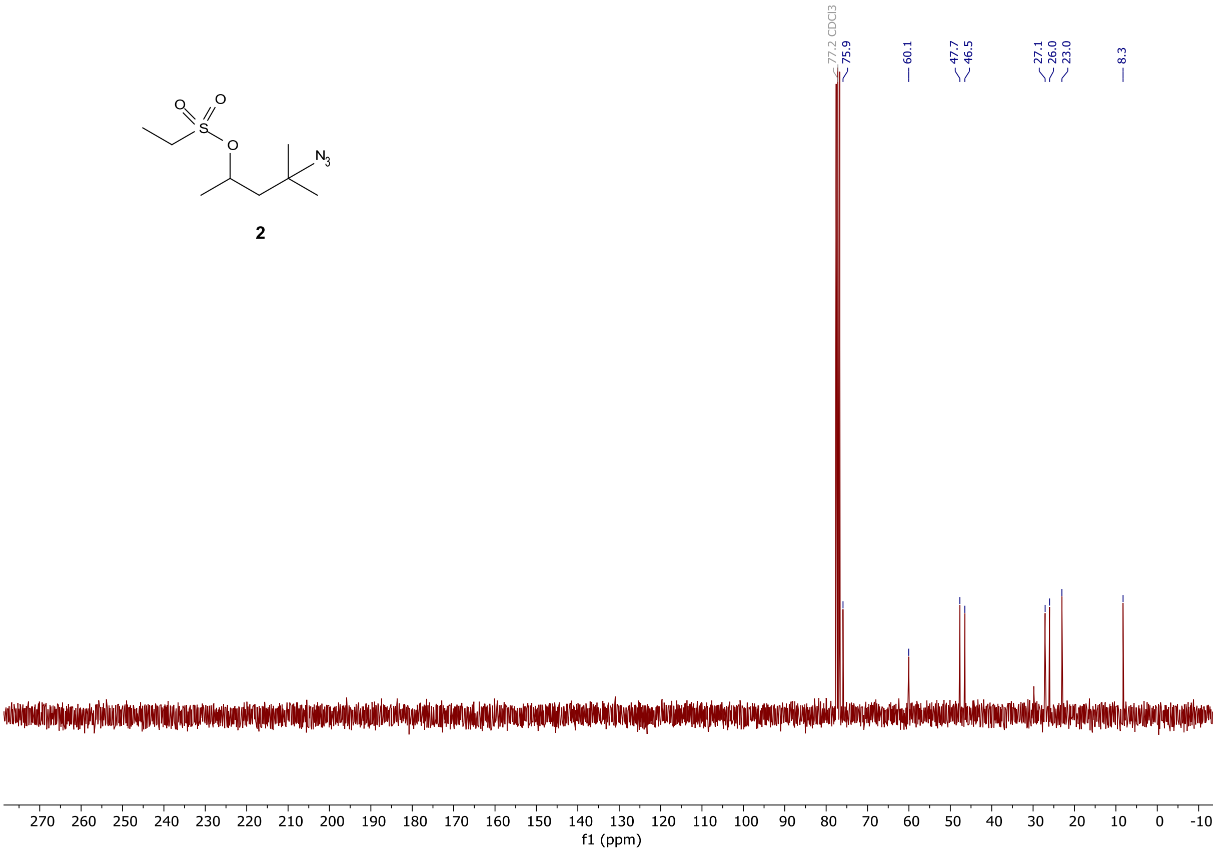

77.2 CDCl3
75.9
60.1
47.7
46.5
27.1
26.0
23.0
8.3
O
O
S
O
N3
2
270 260 250 240 230 220 210 200 190 180 170 160 150 140 130 120 110 100 90 80 70 60 50 40 30 20 10 0 -10
f1 (ppm)

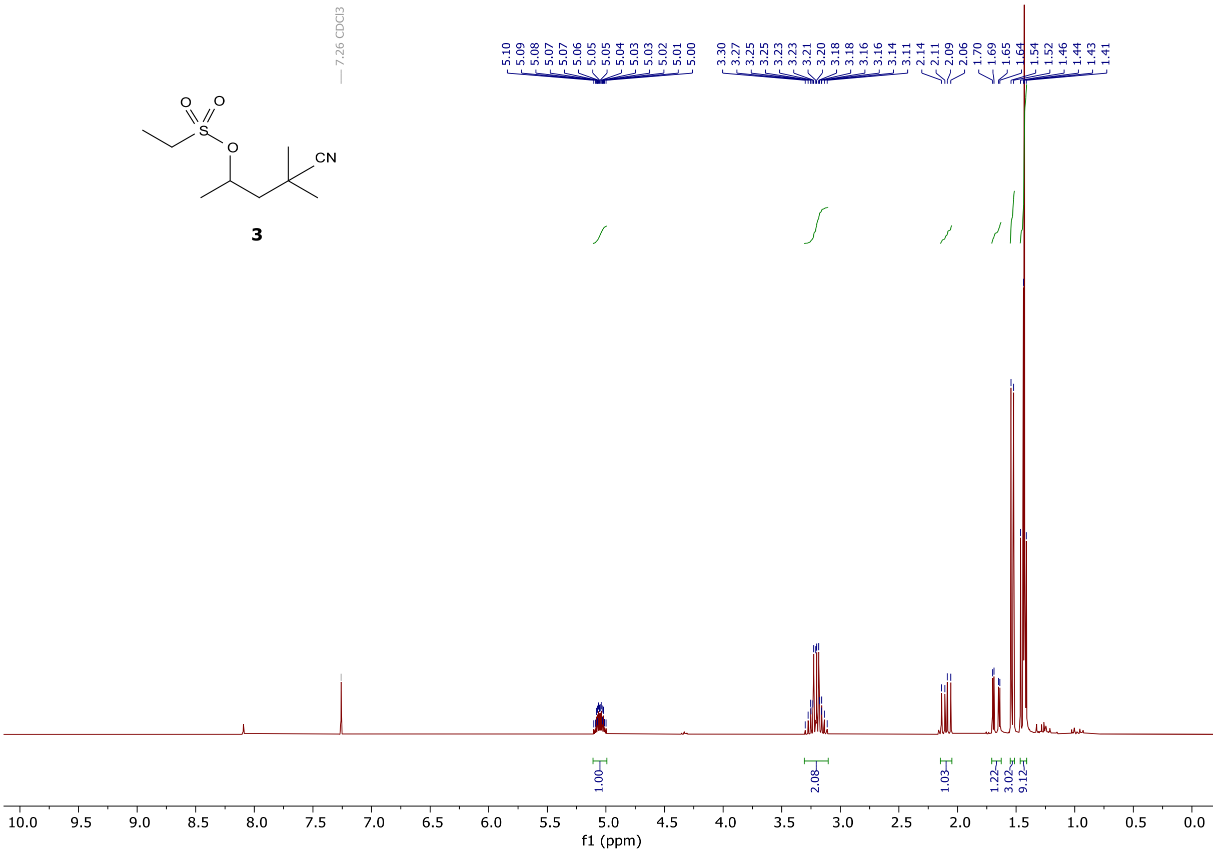
7.26 CDCl3
5.10 5.09 5.08 5.07 5.06 5.05 5.05 5.04 5.03 5.03 5.02 5.01 5.00
3.30 3.27 3.25 3.25 3.23 3.23 3.21 3.20 3.18 3.18 3.16 3.16 3.14 3.11
2.14 2.11 2.09 2.06
1.70 1.69 1.65 1.64 1.54 1.52 1.46 1.44 1.43 1.41
CN
3
1.00
2.08
1.03
1.22
3.02
9.12
f1 (ppm)

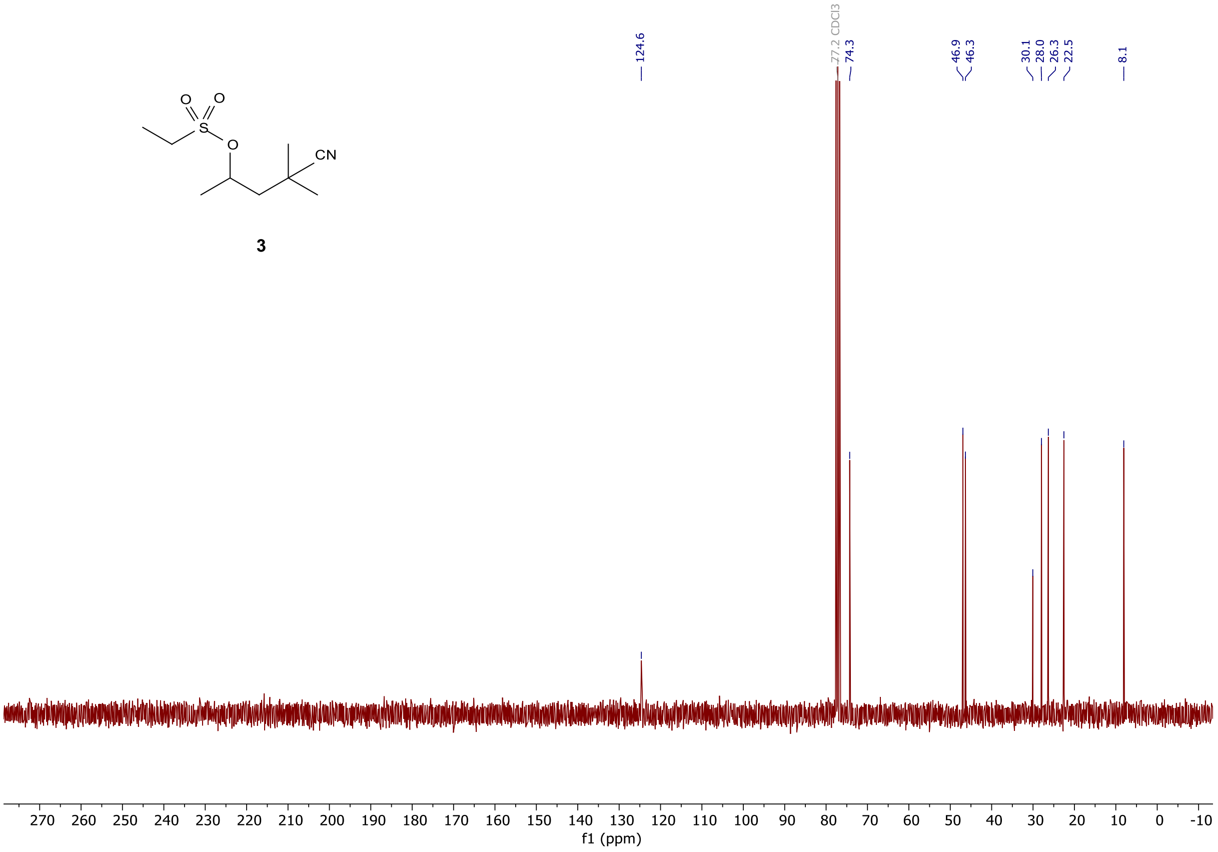
124.6
77.2 CDCl3
74.3
46.9 46.3
30.1 28.0 26.3 22.5
8.1
CN
3
f1 (ppm)

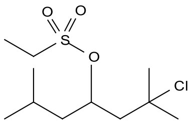
O
O
S
O
Cl

4

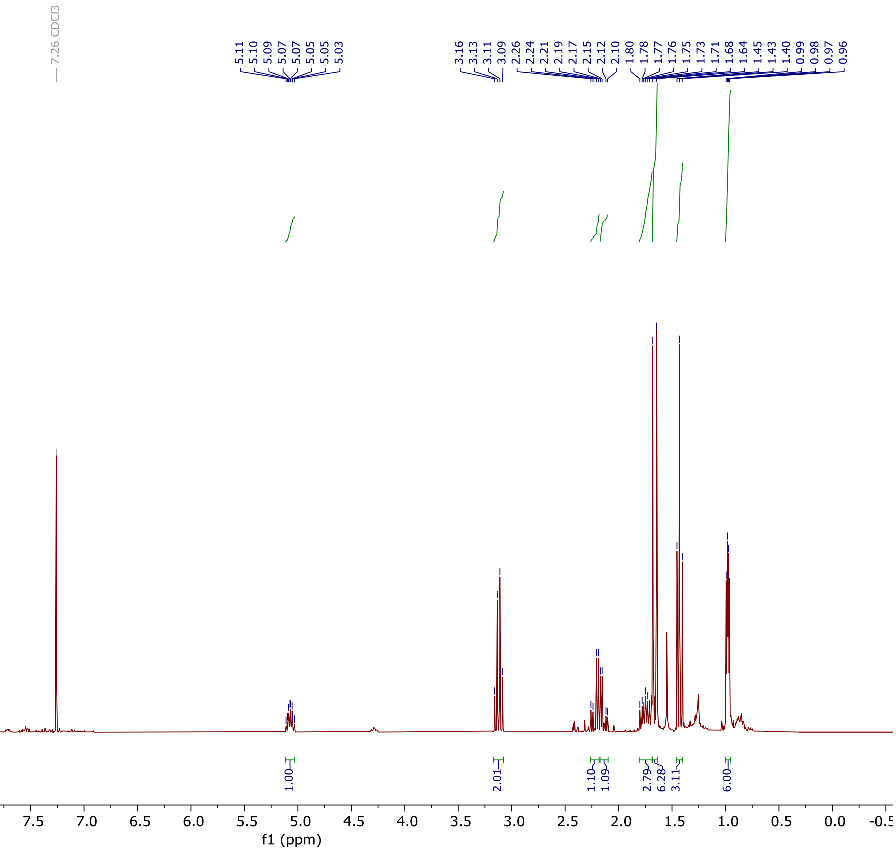
7.26 CDCl3
5.11
5.10
5.09
5.07
5.07
5.05
5.05
5.03
3.16
3.13
3.11
3.09
2.26
2.24
2.21
2.19
2.17
2.15
2.12
2.10
1.80
1.78
1.77
1.76
1.75
1.73
1.71
1.68
1.64
1.45
1.43
1.40
0.99
0.98
0.97
0.96
1.00
2.01
1.10
1.09
2.79
6.28
3.11
6.00
10.5 10.0 9.5 9.0 8.5 8.0 7.5 7.0 6.5 6.0 5.5 5.0 4.5 4.0 3.5 3.0 2.5 2.0 1.5 1.0 0.5 0.0 -0.5
f1 (ppm)

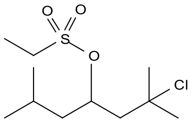
O
O
S
O
Cl

4

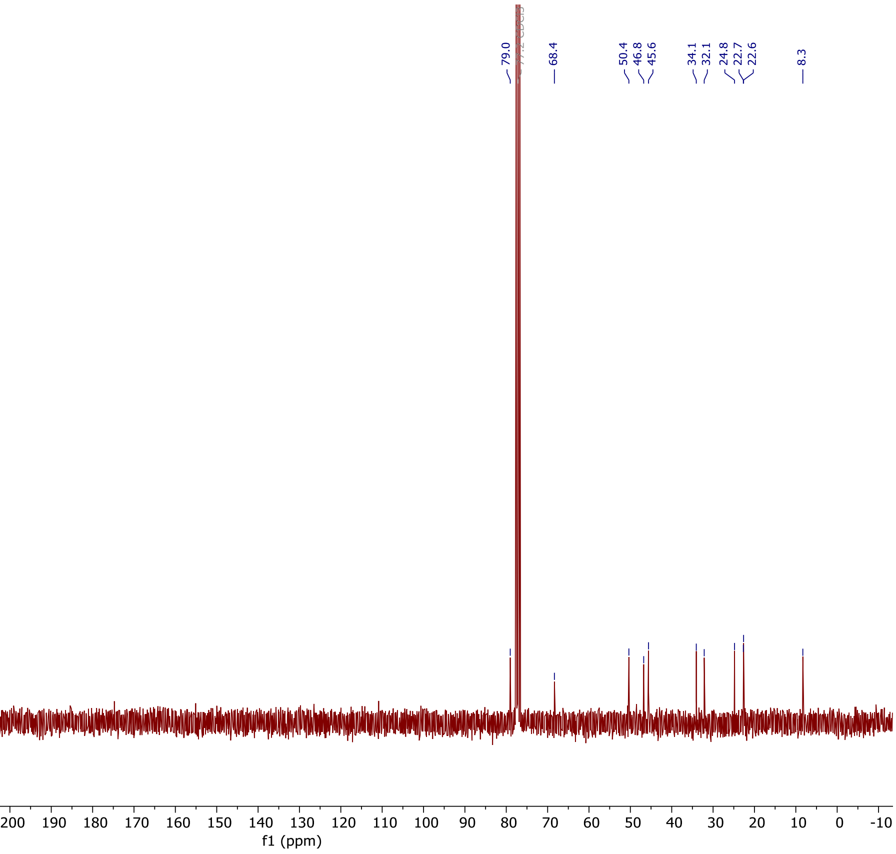
79.0
68.4
50.4
46.8
45.6
34.1
32.1
24.8
22.7
22.6
8.3
270 260 250 240 230 220 210 200 190 180 170 160 150 140 130 120 110 100 90 80 70 60 50 40 30 20 10 0 -10
f1 (ppm)

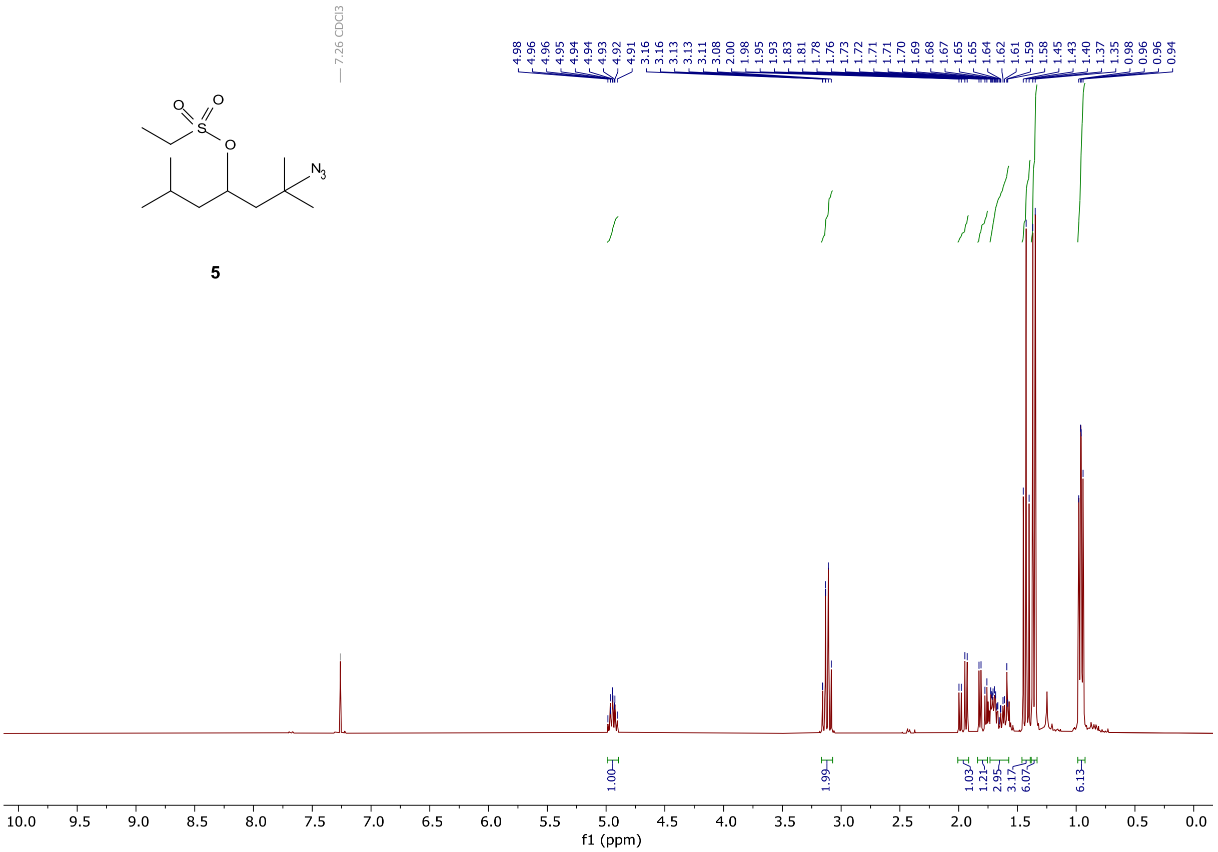
5
7.26 CDCl3
4.98 4.96 4.96 4.95 4.94 4.94 4.93 4.92 4.91 3.16 3.13 3.13 3.11 3.08 2.00 1.98 1.95 1.93 1.83 1.81 1.78 1.76 1.73 1.72 1.71 1.71 1.70 1.69 1.68 1.67 1.65 1.65 1.64 1.62 1.61 1.59 1.58 1.45 1.43 1.40 1.37 1.35 0.98 0.96 0.96 0.94
1.00 1.99 1.03 1.21 2.95 3.17 6.07 6.13
10.0 9.5 9.0 8.5 8.0 7.5 7.0 6.5 6.0 5.5 5.0 4.5 4.0 3.5 3.0 2.5 2.0 1.5 1.0 0.5 0.0
f1 (ppm)

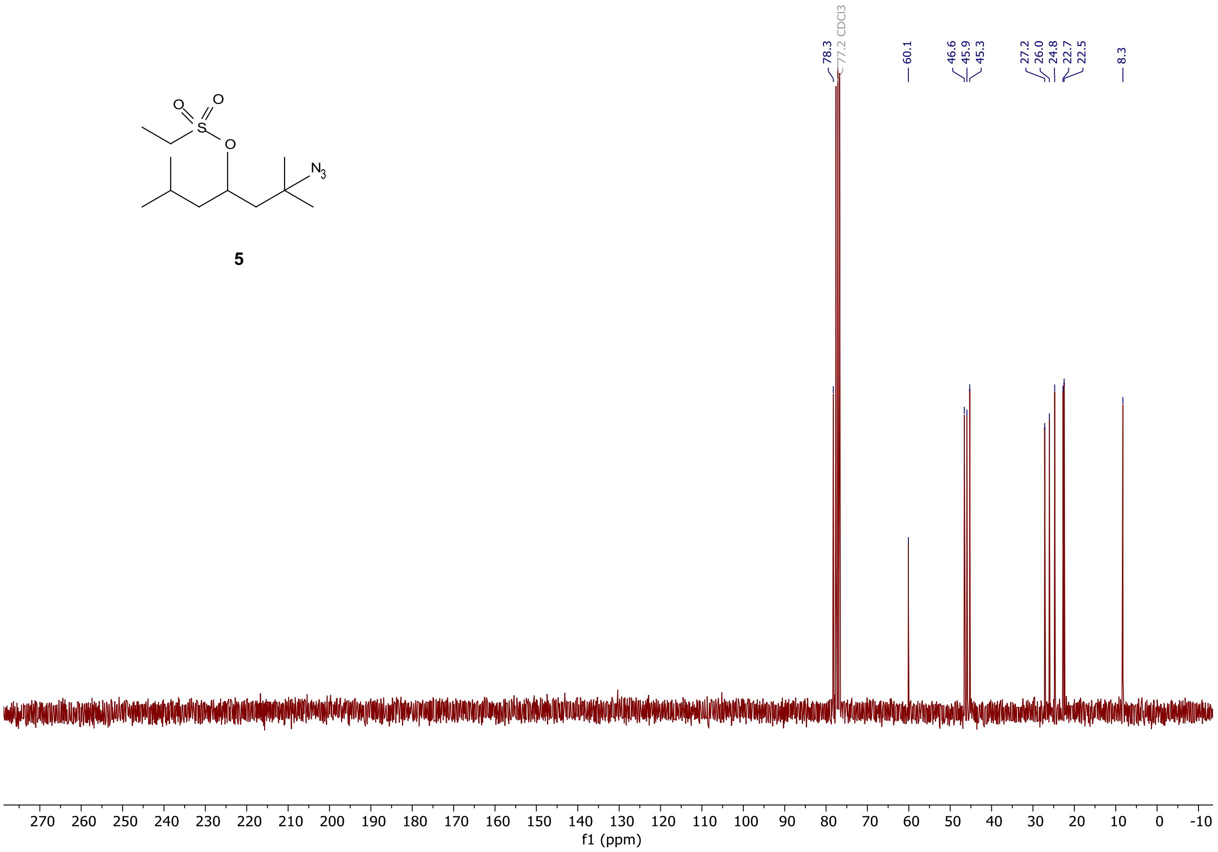
5
78.3 77.2 CDCl3 60.1 46.6 45.9 45.3 27.2 26.0 24.8 22.7 22.5 8.3
270 260 250 240 230 220 210 200 190 180 170 160 150 140 130 120 110 100 90 80 70 60 50 40 30 20 10 0 -10
f1 (ppm)

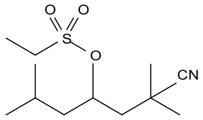

6

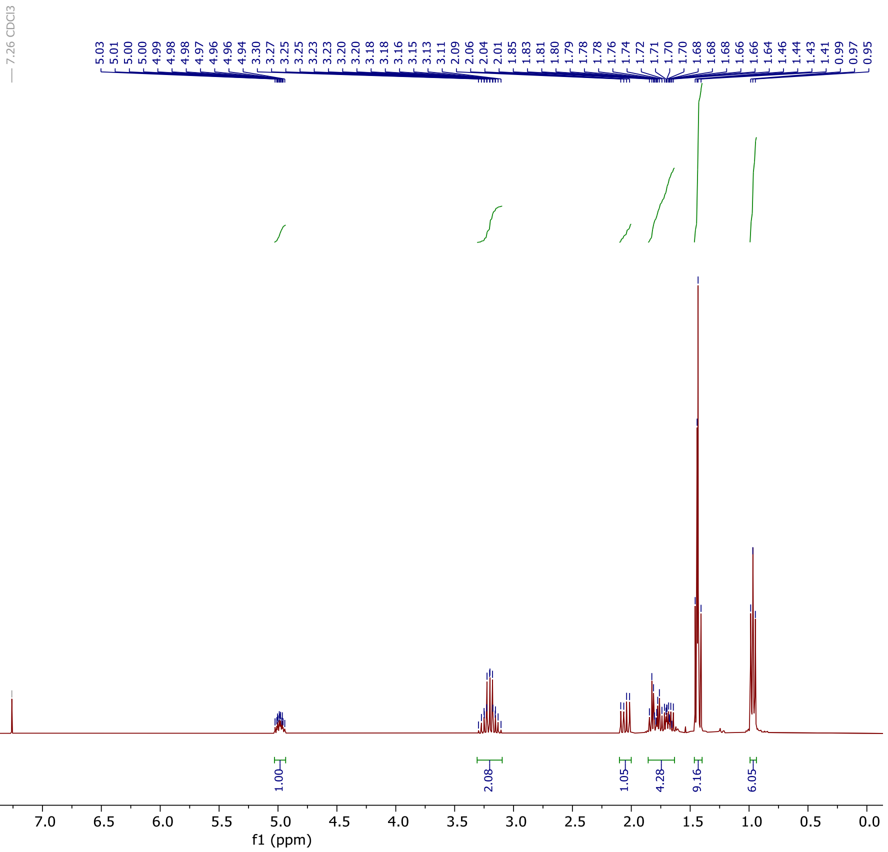

7.26 CDCl3
5.03
5.01
5.00
5.00
4.99
4.98
4.98
4.97
4.96
4.96
4.94
3.30
3.27
3.25
3.25
3.23
3.23
3.20
3.20
3.18
3.18
3.16
3.15
3.13
3.11
2.09
2.06
2.04
2.01
1.85
1.83
1.81
1.80
1.79
1.78
1.78
1.76
1.74
1.72
1.71
1.70
1.70
1.68
1.68
1.68
1.66
1.66
1.64
1.46
1.44
1.43
1.41
0.99
0.97
0.95
1.00
2.08
1.05
4.28
9.16
6.05
10.0 9.5 9.0 8.5 8.0 7.5 7.0 6.5 6.0 5.5 5.0 4.5 4.0 3.5 3.0 2.5 2.0 1.5 1.0 0.5 0.0
f1 (ppm)

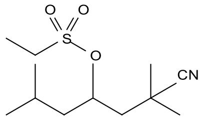

6

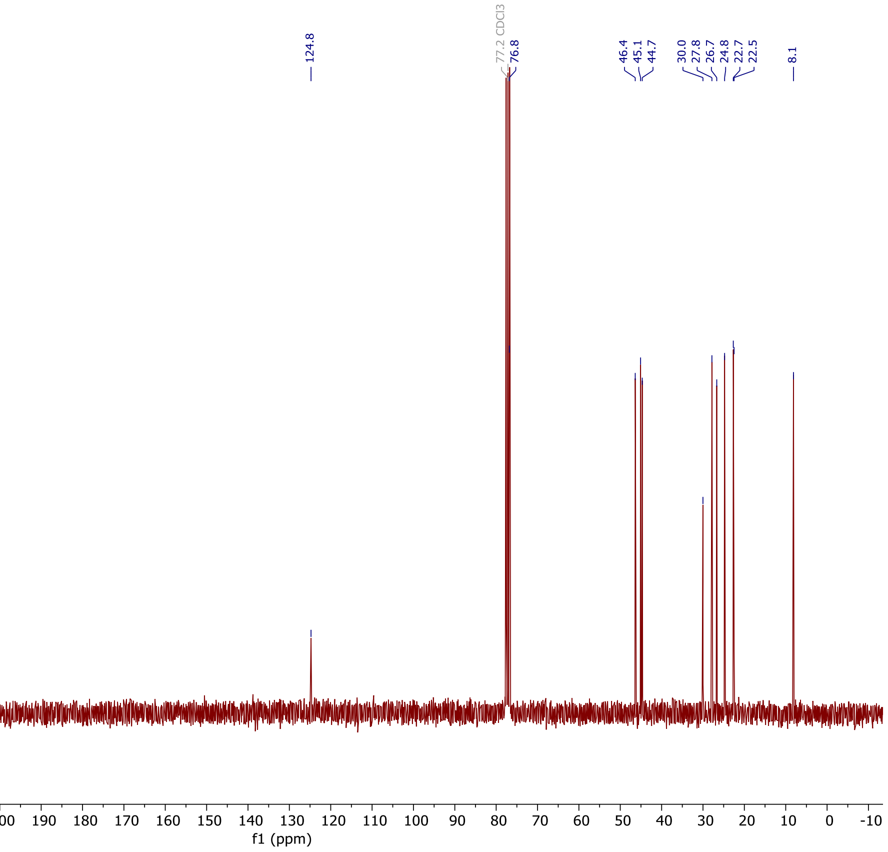

124.8
77.2 CDCl3
76.8
46.4
45.1
44.7
30.0
27.8
26.7
24.8
22.7
22.5
8.1
270 260 250 240 230 220 210 200 190 180 170 160 150 140 130 120 110 100 90 80 70 60 50 40 30 20 10 0 -10
f1 (ppm)

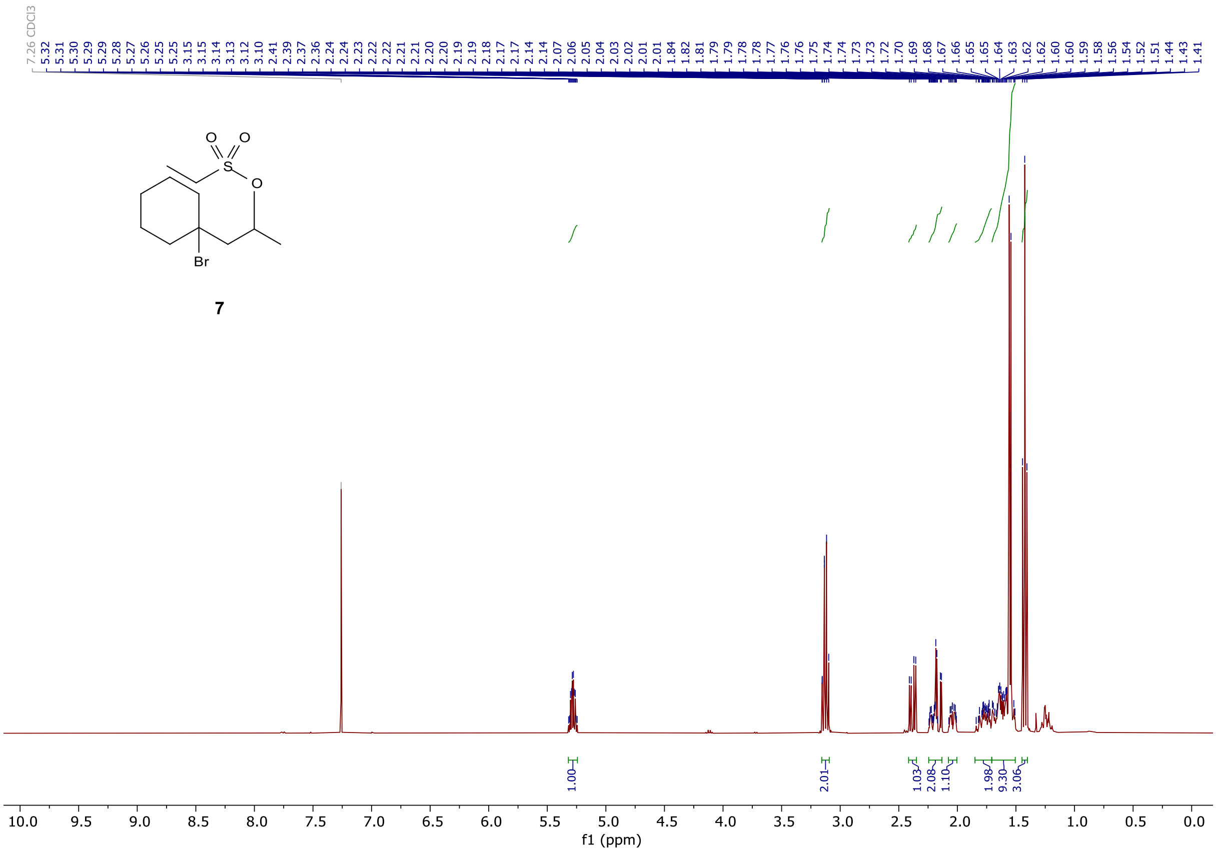
7.26 CDCl3
5.32
5.31
5.30
5.29
5.29
5.28
5.27
5.26
5.25
5.25
3.15
3.15
3.14
3.13
3.12
3.10
2.41
2.39
2.37
2.36
2.24
2.24
2.23
2.22
2.22
2.21
2.21
2.20
2.20
2.19
2.19
2.18
2.17
2.17
2.14
2.14
2.07
2.06
2.05
2.04
2.03
2.02
2.01
2.01
1.84
1.82
1.81
1.79
1.79
1.78
1.77
1.76
1.76
1.75
1.74
1.74
1.73
1.73
1.72
1.70
1.69
1.68
1.67
1.66
1.65
1.65
1.64
1.63
1.62
1.62
1.60
1.59
1.58
1.56
1.54
1.52
1.51
1.44
1.43
1.41
O
O
S
O
Br
7
1.00
2.01
1.03
2.08
1.10
1.98
9.30
3.06
10.0
9.5
9.0
8.5
8.0
7.5
7.0
6.5
6.0
5.5
5.0
4.5
4.0
3.5
3.0
2.5
2.0
1.5
1.0
0.5
0.0
f1 (ppm)

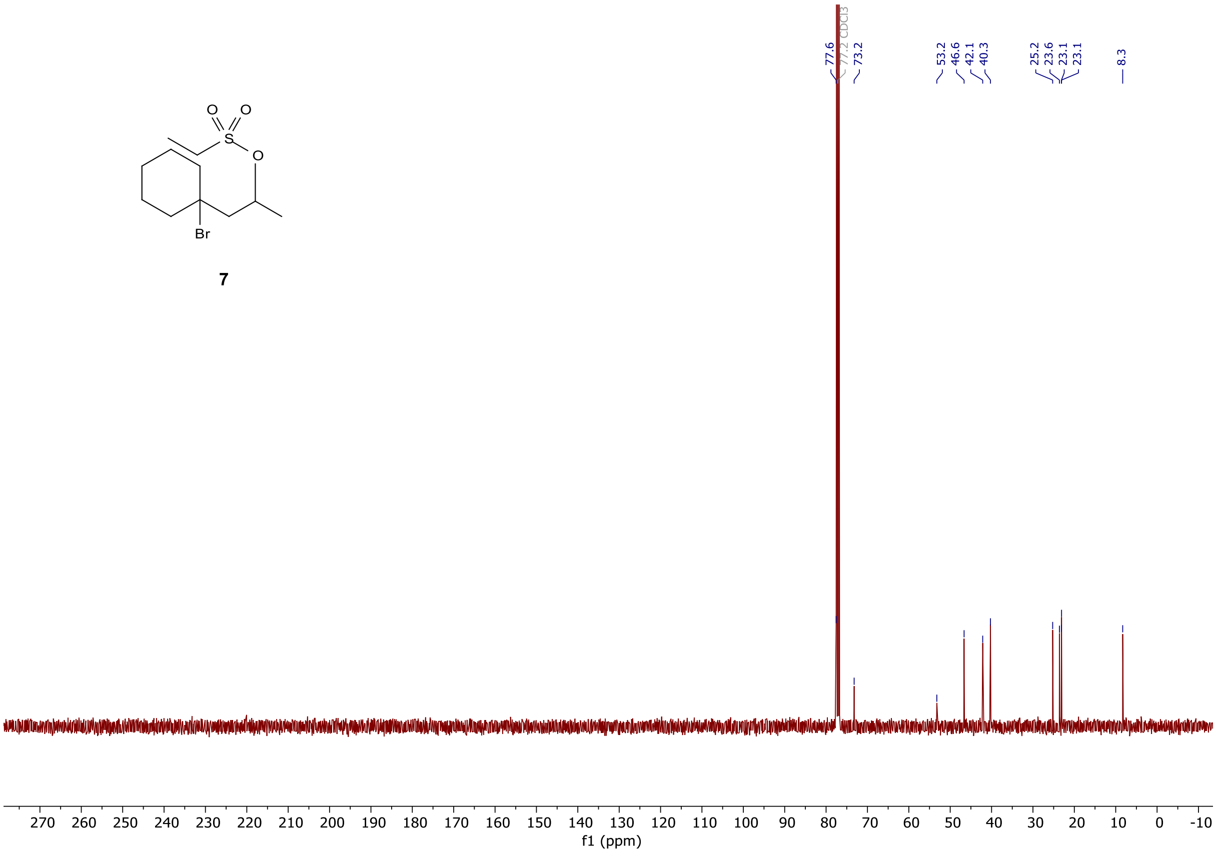
77.6
77.2 CDCl3
73.2
53.2
46.6
42.1
40.3
25.2
23.6
23.1
23.1
8.3
O
O
S
O
Br
7
270
260
250
240
230
220
210
200
190
180
170
160
150
140
130
120
110
100
90
80
70
60
50
40
30
20
10
0
-10
f1 (ppm)

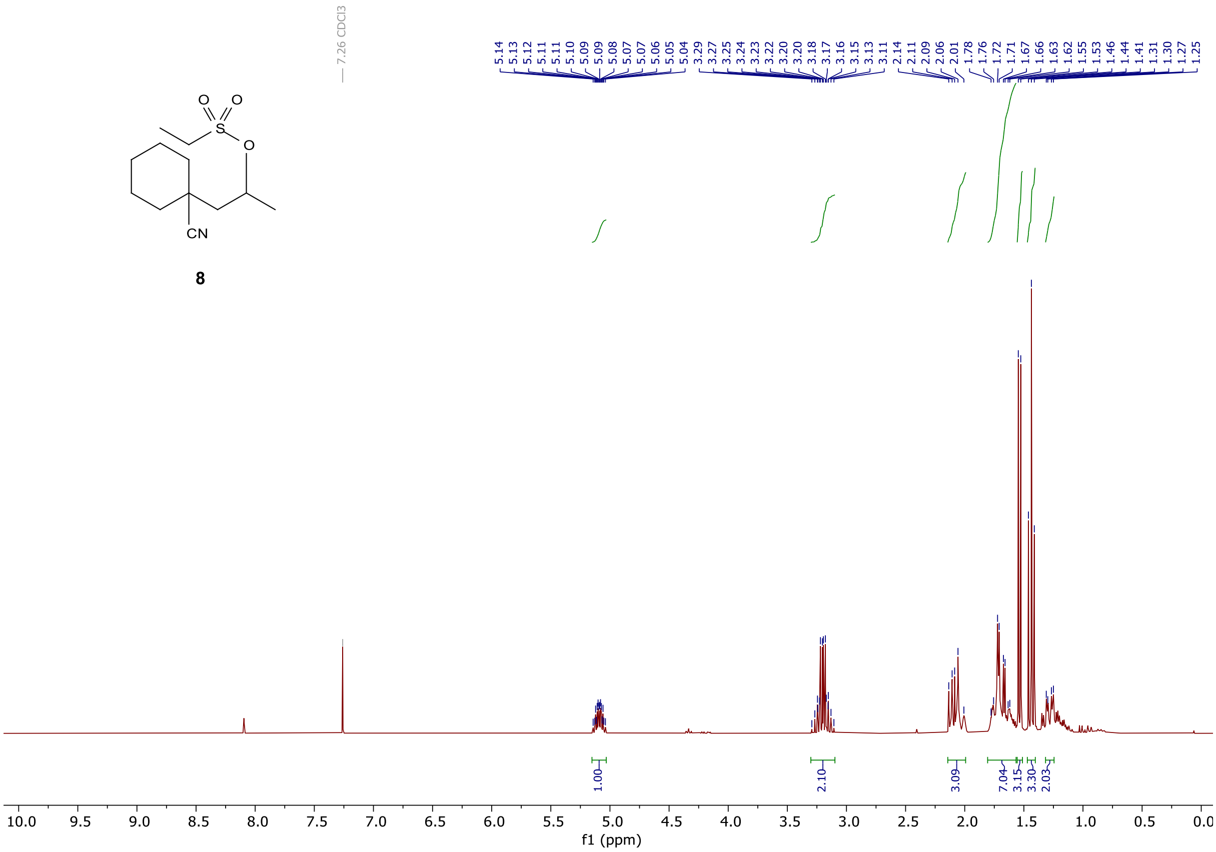

O
O
S
O
CN
8
7.26 CDCl3
5.14
5.13
5.12
5.11
5.11
5.10
5.09
5.08
5.07
5.07
5.06
5.05
5.04
3.29
3.27
3.25
3.24
3.23
3.22
3.20
3.20
3.18
3.17
3.16
3.15
3.13
3.13
3.11
2.14
2.11
2.09
2.06
2.01
1.78
1.76
1.72
1.71
1.67
1.66
1.63
1.62
1.55
1.53
1.46
1.44
1.41
1.31
1.30
1.27
1.25
1.00
2.10
3.09
7.04
3.15
3.30
2.03
10.0 9.5 9.0 8.5 8.0 7.5 7.0 6.5 6.0 5.5 5.0 4.5 4.0 3.5 3.0 2.5 2.0 1.5 1.0 0.5 0.0
f1 (ppm)

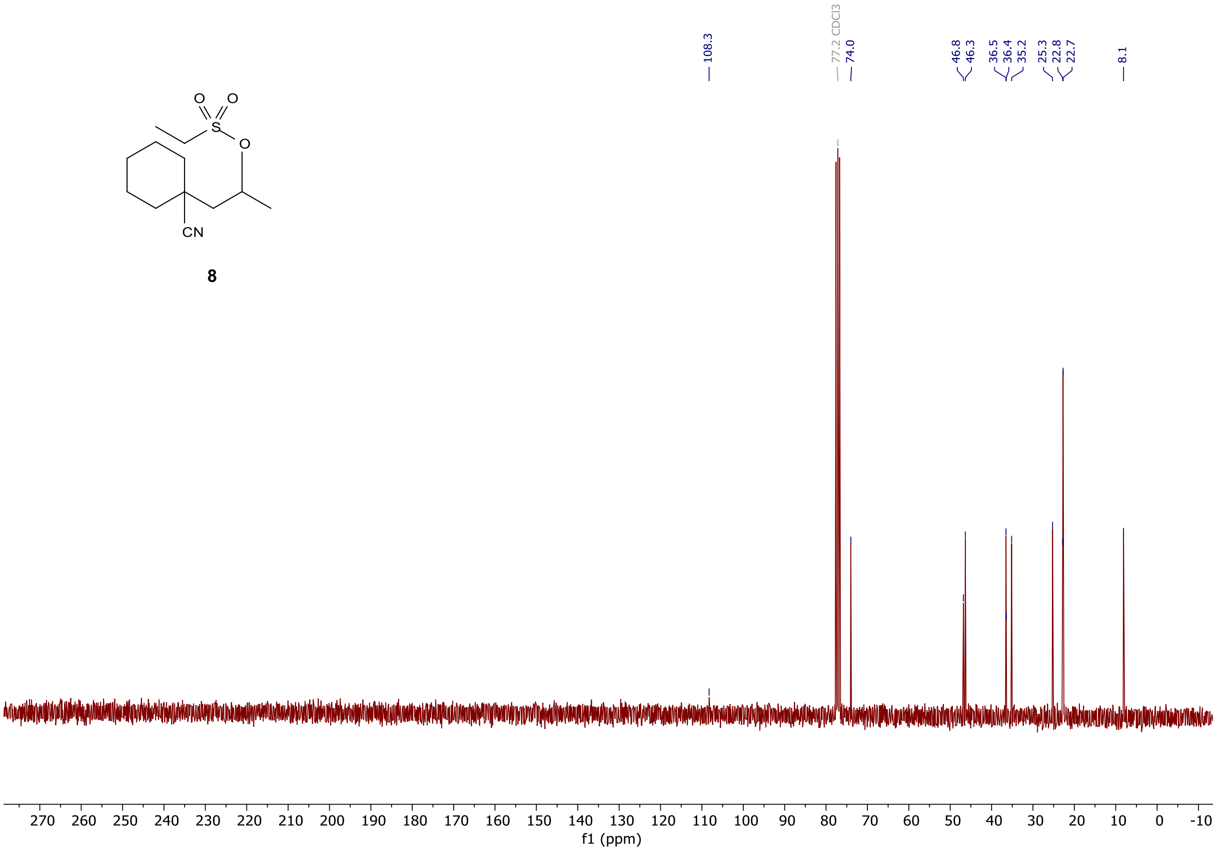

O
O
S
O
CN
8
108.3
77.2 CDCl3
74.0
46.8
46.3
36.5
36.4
35.2
25.3
22.8
22.7
8.1
270 260 250 240 230 220 210 200 190 180 170 160 150 140 130 120 110 100 90 80 70 60 50 40 30 20 10 0 -10
f1 (ppm)

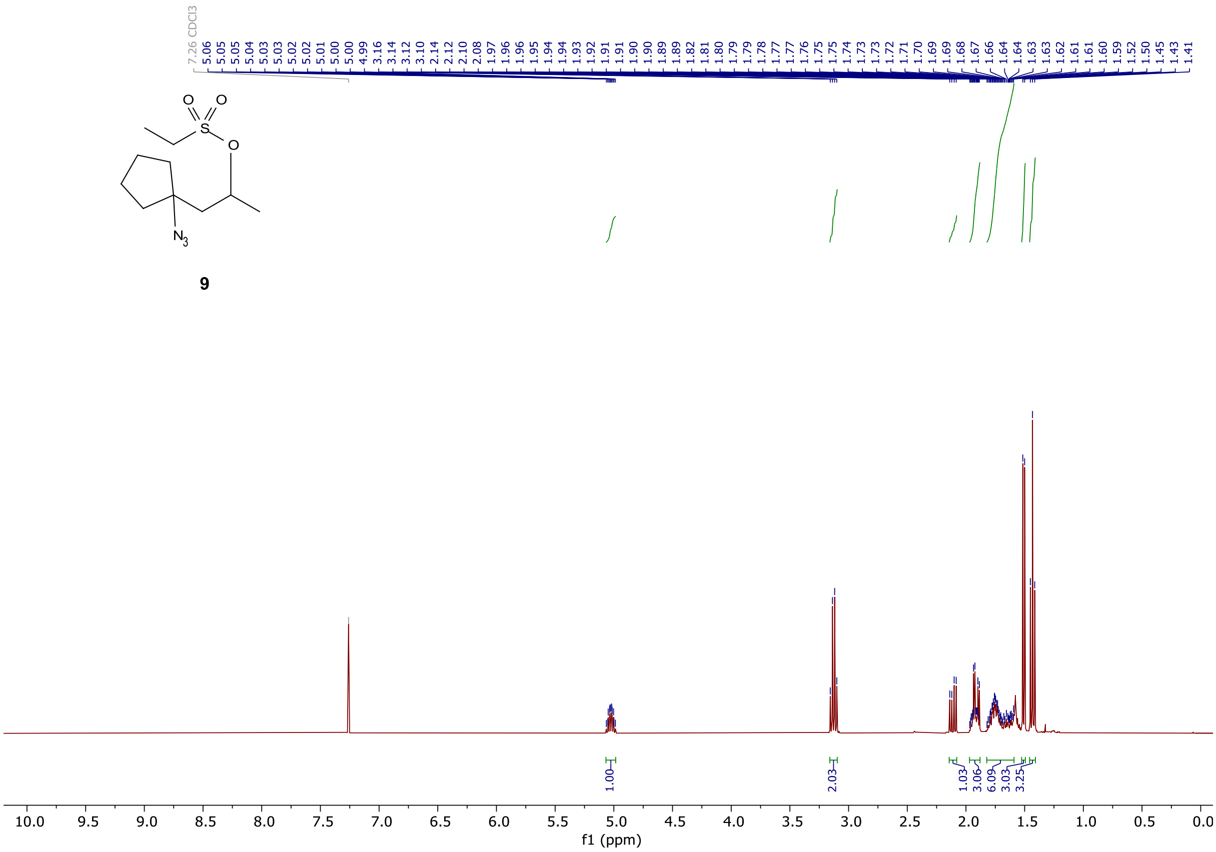
7.26 CDCl3
5.06 5.05 5.05 5.04 5.03 5.03 5.02 5.02 5.01 5.00 5.00 4.99 3.16 3.14 3.12 3.10 2.14 2.12 2.10 2.08 1.97 1.96 1.96 1.95 1.94 1.94 1.93 1.92 1.91 1.91 1.90 1.90 1.89 1.89 1.82 1.81 1.80 1.79 1.79 1.78 1.77 1.77 1.76 1.75 1.75 1.74 1.73 1.73 1.72 1.71 1.70 1.69 1.69 1.68 1.67 1.66 1.64 1.64 1.63 1.63 1.62 1.61 1.61 1.60 1.59 1.52 1.50 1.45 1.43 1.41
9
1.00
2.03
1.03
3.06
6.09
3.03
3.25
f1 (ppm)

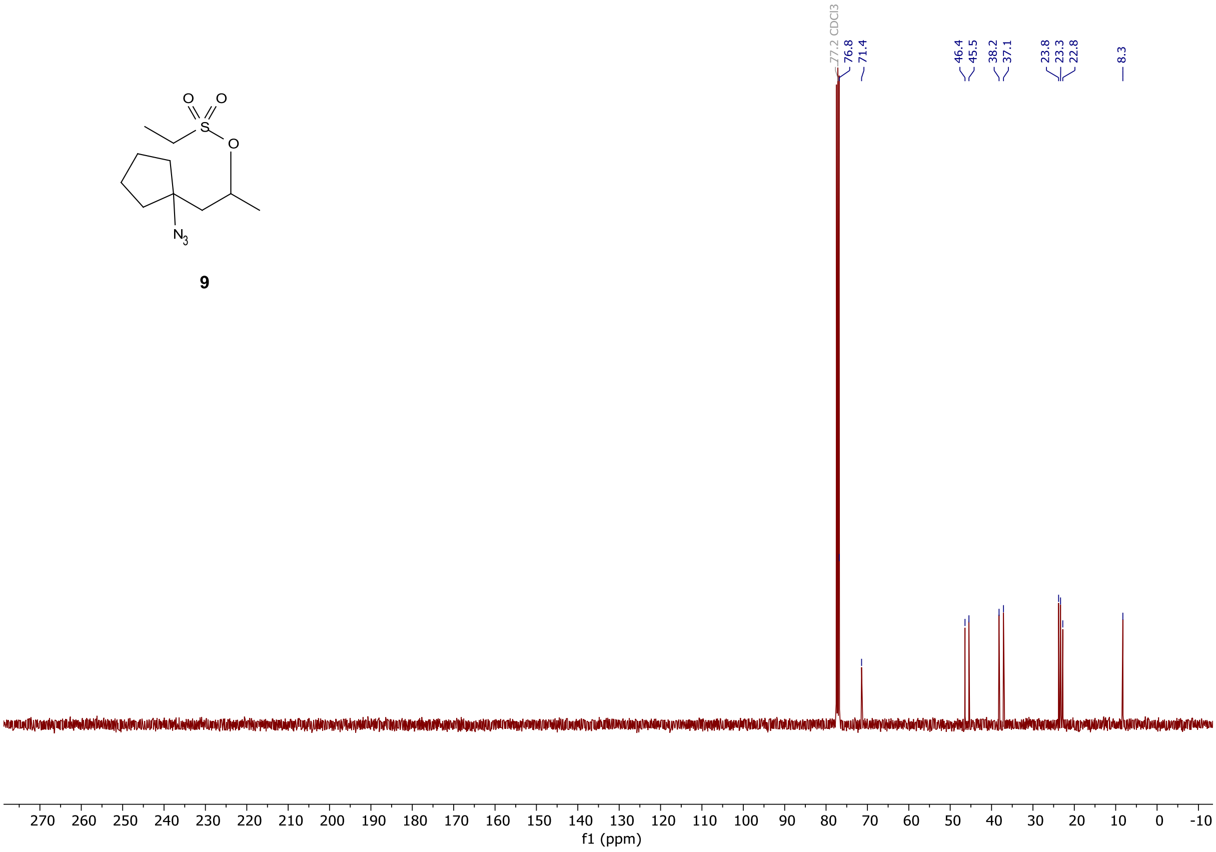
77.2 CDCl3
76.8
71.4
46.4
45.5
38.2
37.1
23.8
23.3
22.8
8.3
9
f1 (ppm)

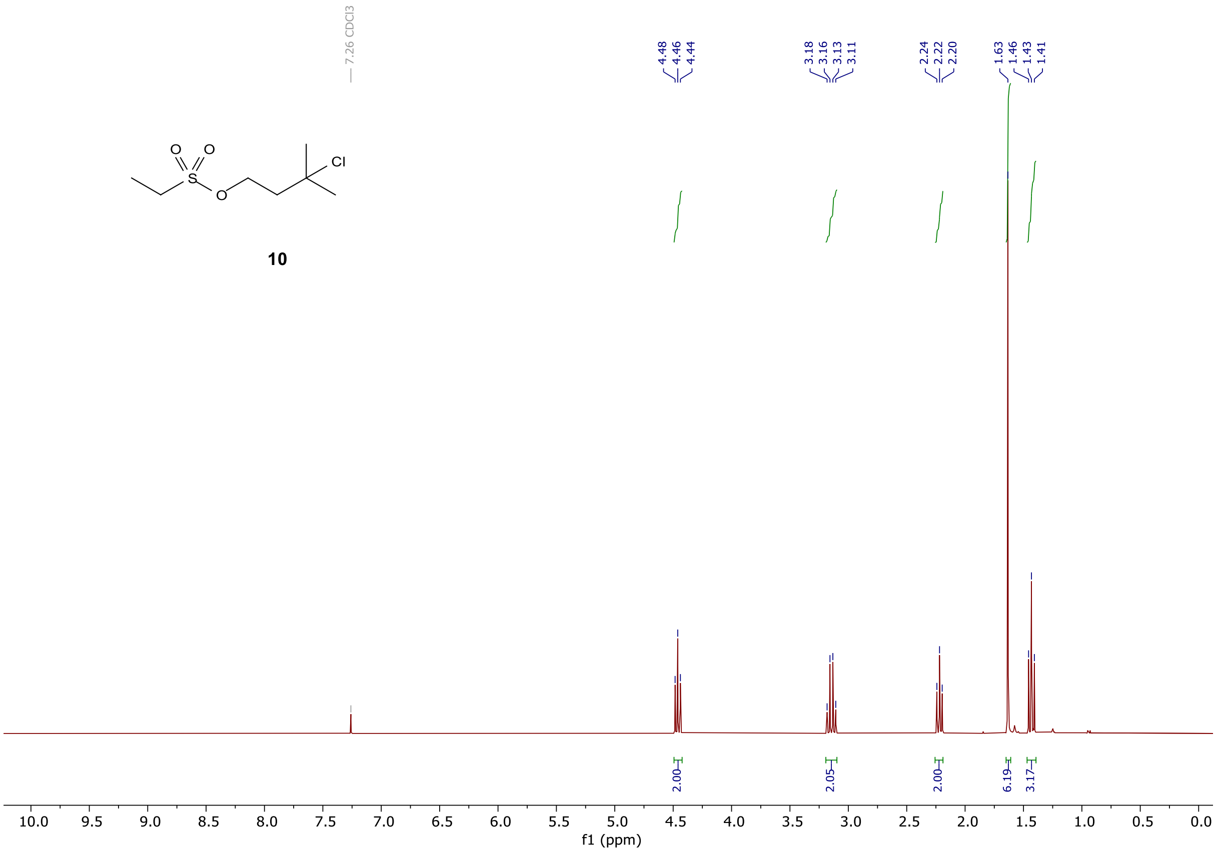
7.26 CDCl3
4.48
4.46
4.44
3.18
3.16
3.13
3.11
2.24
2.22
2.20
1.63
1.46
1.43
1.41
O
O
S
O
Cl
10
2.00
2.05
2.00
6.19
3.17
10.0 9.5 9.0 8.5 8.0 7.5 7.0 6.5 6.0 5.5 5.0 4.5 4.0 3.5 3.0 2.5 2.0 1.5 1.0 0.5 0.0
f1 (ppm)

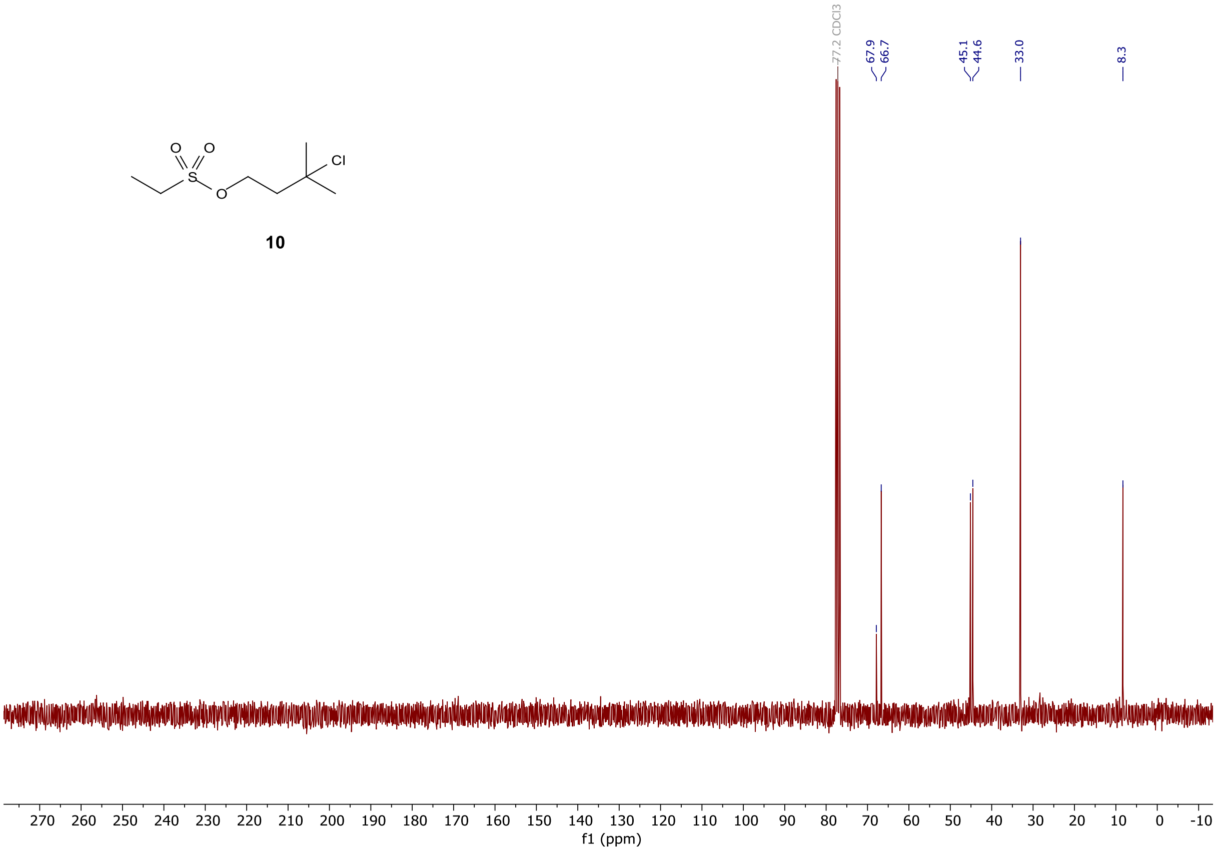
77.2 CDCl3
67.9
66.7
45.1
44.6
33.0
8.3
O
O
S
O
Cl
10
270 260 250 240 230 220 210 200 190 180 170 160 150 140 130 120 110 100 90 80 70 60 50 40 30 20 10 0 -10
f1 (ppm)

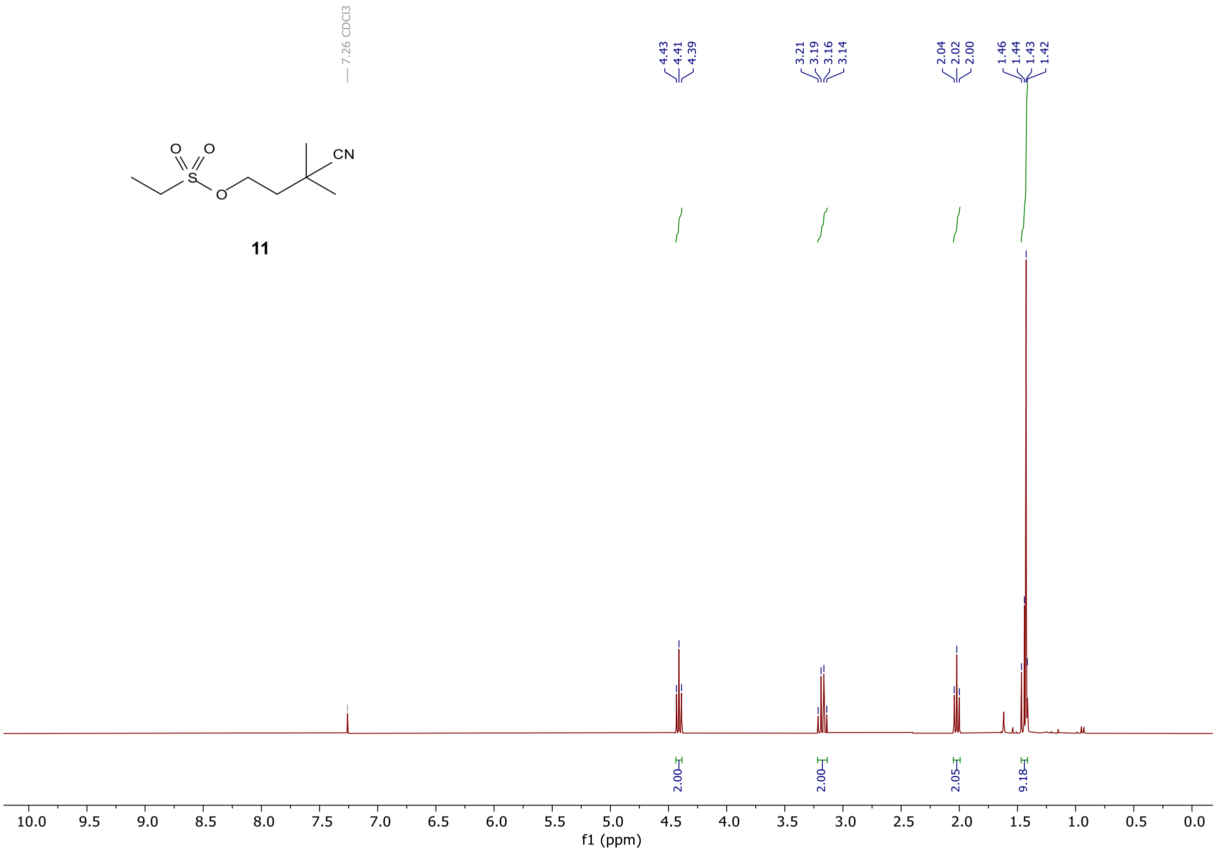
7.26 CDCl3
4.43
4.41
4.39
3.21
3.19
3.16
3.14
2.04
2.02
2.00
1.46
1.44
1.43
1.42
O
O
S
O
CN
11
2.00
2.00
2.05
9.18
10.0 9.5 9.0 8.5 8.0 7.5 7.0 6.5 6.0 5.5 5.0 4.5 4.0 3.5 3.0 2.5 2.0 1.5 1.0 0.5 0.0
f1 (ppm)

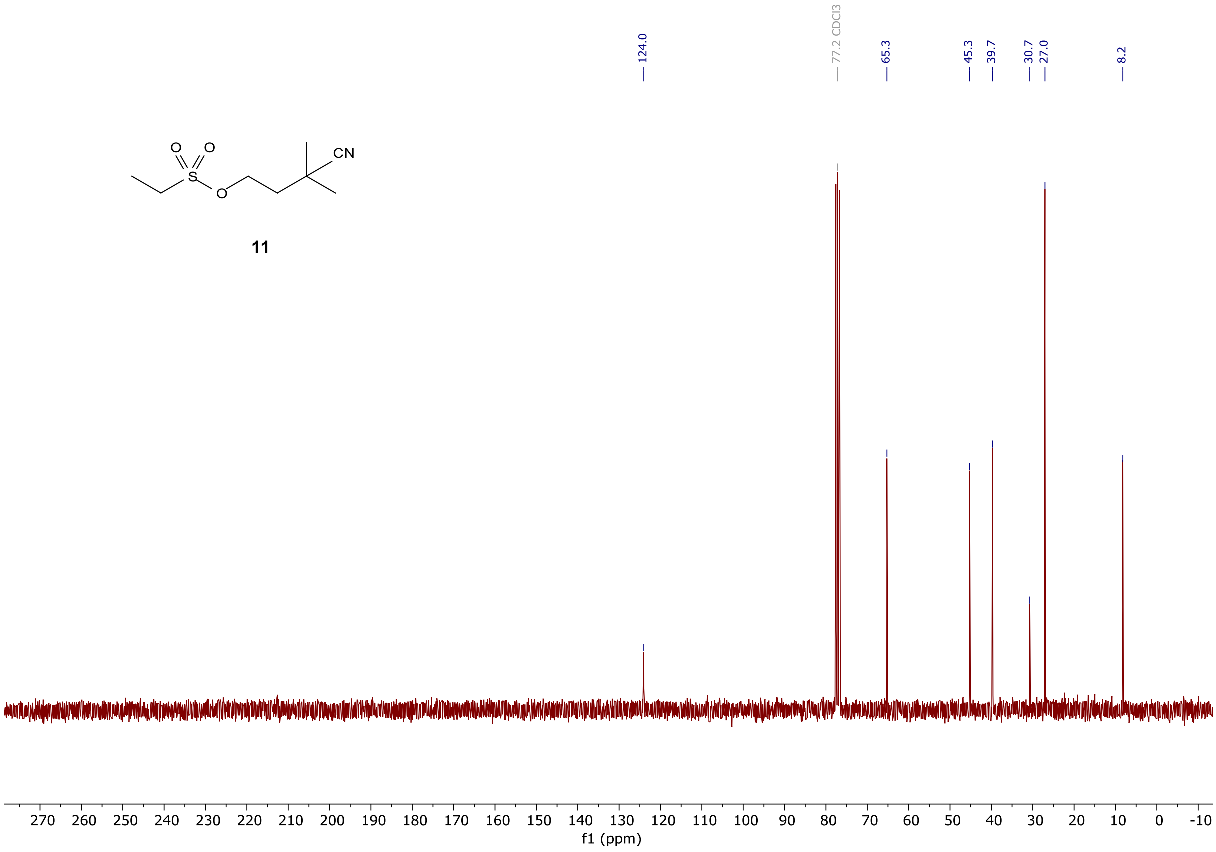
124.0
77.2 CDCl3
65.3
45.3
39.7
30.7
27.0
8.2
O
O
S
O
CN
11
270 260 250 240 230 220 210 200 190 180 170 160 150 140 130 120 110 100 90 80 70 60 50 40 30 20 10 0 -10
f1 (ppm)

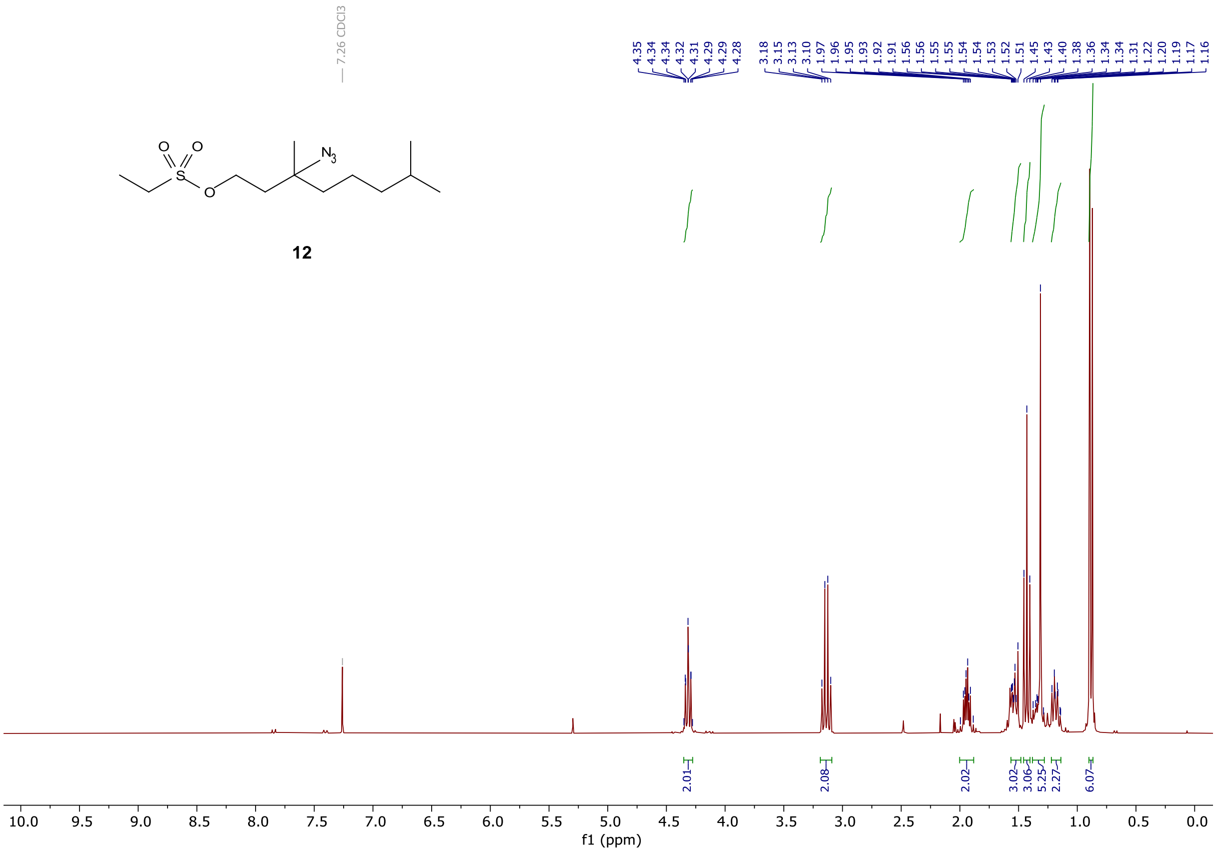
12
7.26 CDCl3
f1 (ppm)

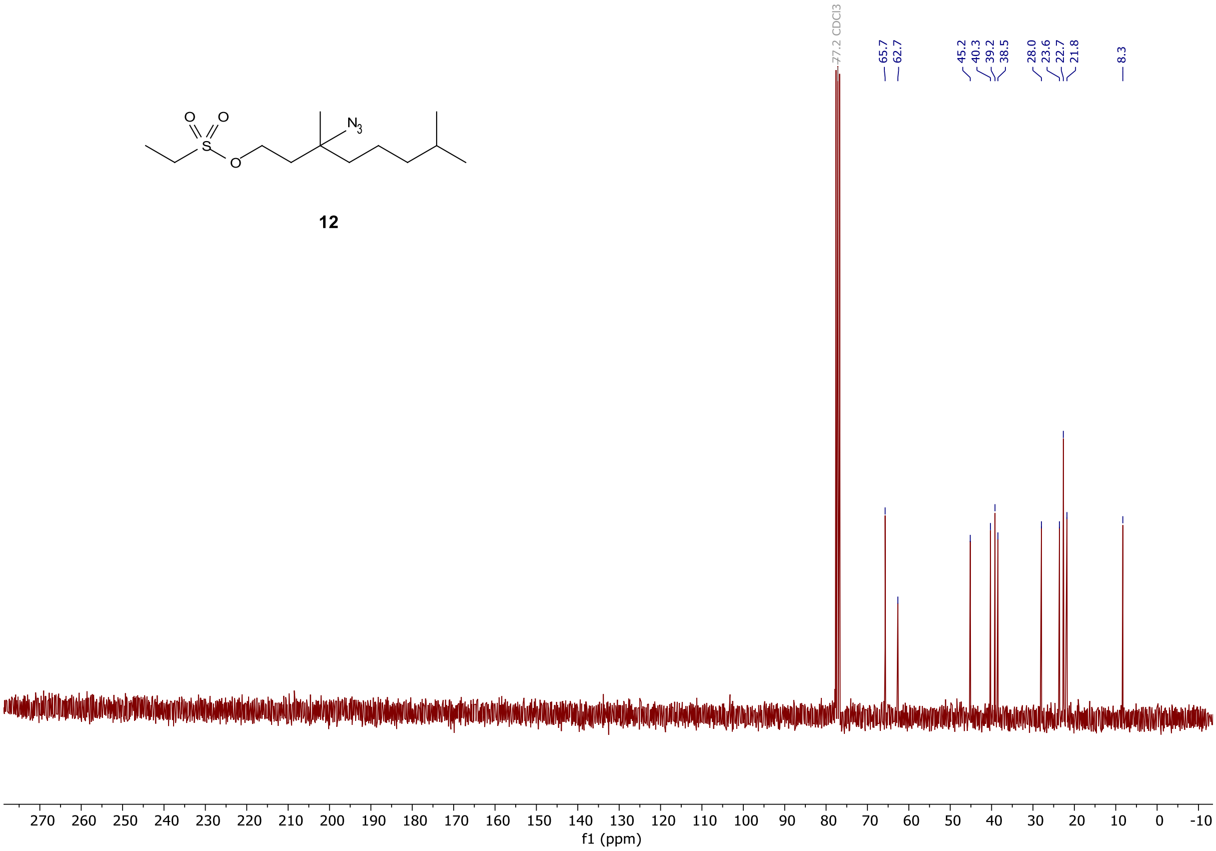
12
77.2 CDCl3
65.7
62.7
45.2
40.3
39.2
38.5
28.0
23.6
22.7
21.8
8.3
f1 (ppm)

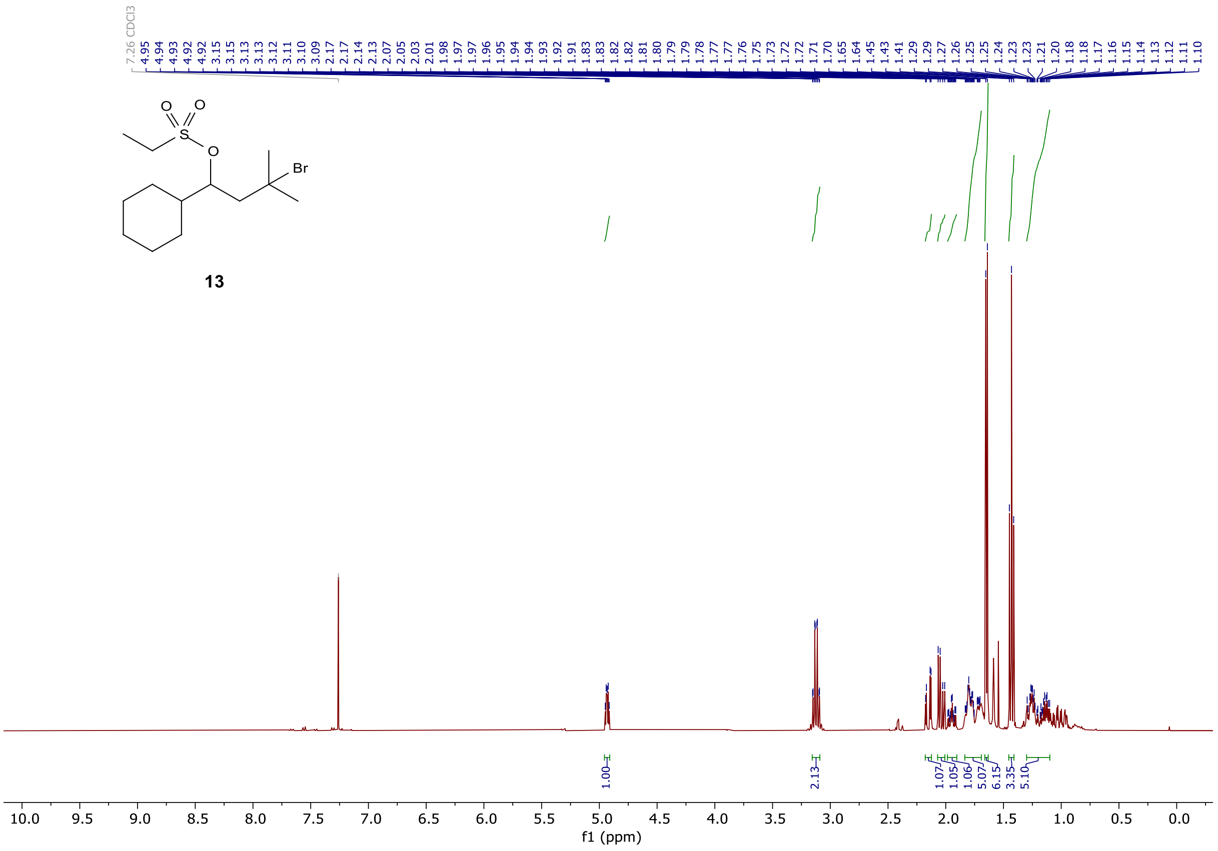

13

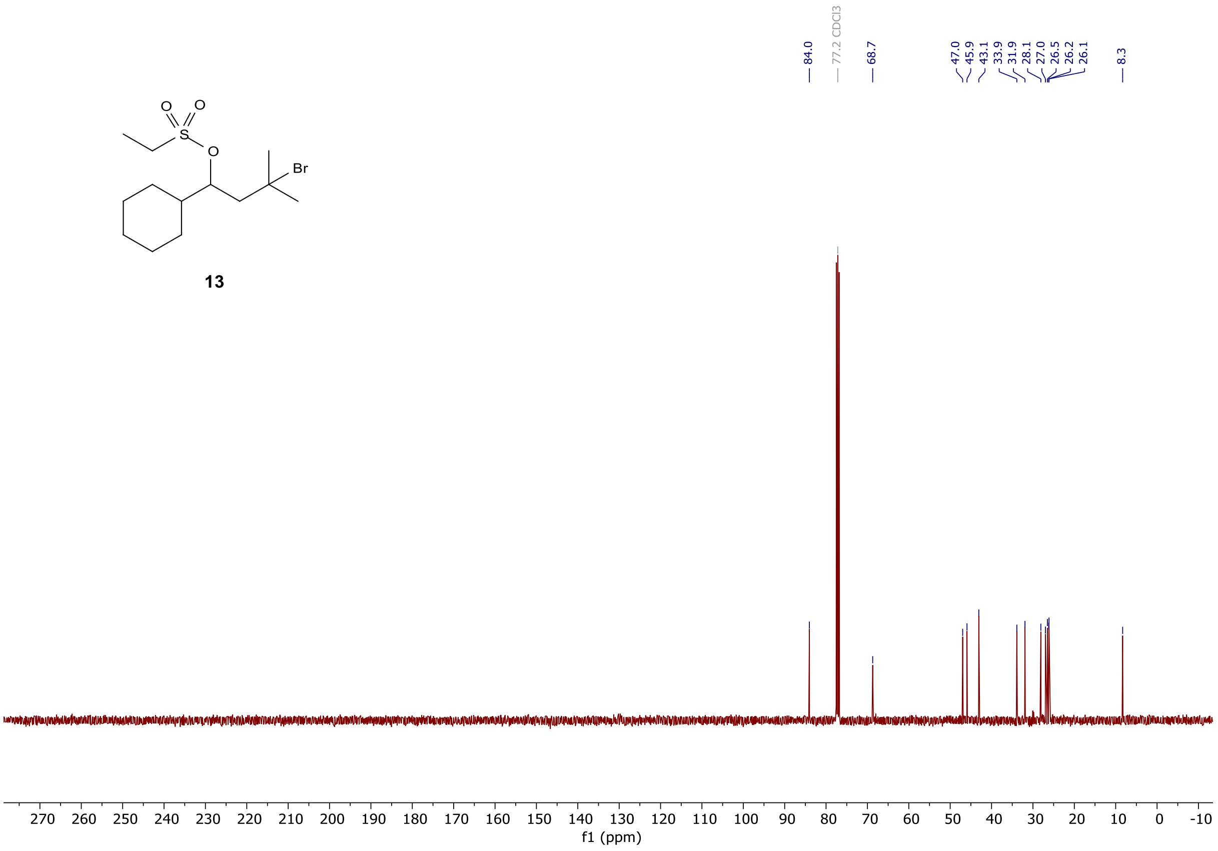

13

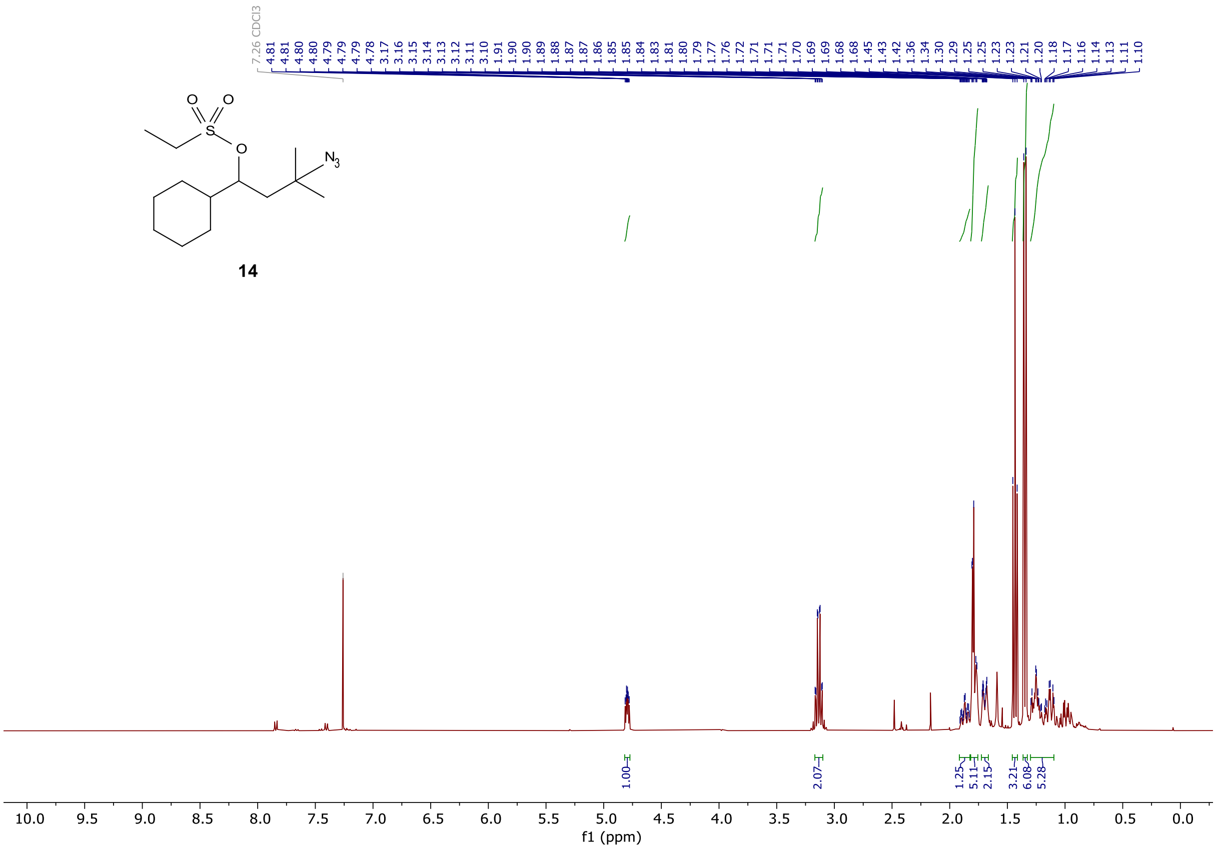
7.26 CDCl3
4.81 4.81 4.80 4.80 4.79 4.79 4.79 4.78 3.17 3.16 3.15 3.14 3.13 3.12 3.11 3.10 1.91 1.90 1.90 1.89 1.88 1.87 1.87 1.86 1.85 1.85 1.84 1.83 1.81 1.80 1.79 1.77 1.76 1.72 1.71 1.71 1.71 1.70 1.69 1.69 1.68 1.68 1.45 1.43 1.42 1.36 1.34 1.30 1.29 1.25 1.25 1.23 1.23 1.21 1.20 1.18 1.17 1.16 1.13 1.11 1.10
N_3
14
1.00
2.07
1.25
5.11
2.15
3.21
6.08
5.28
10.0 9.5 9.0 8.5 8.0 7.5 7.0 6.5 6.0 5.5 5.0 4.5 4.0 3.5 3.0 2.5 2.0 1.5 1.0 0.5 0.0
f1 (ppm)

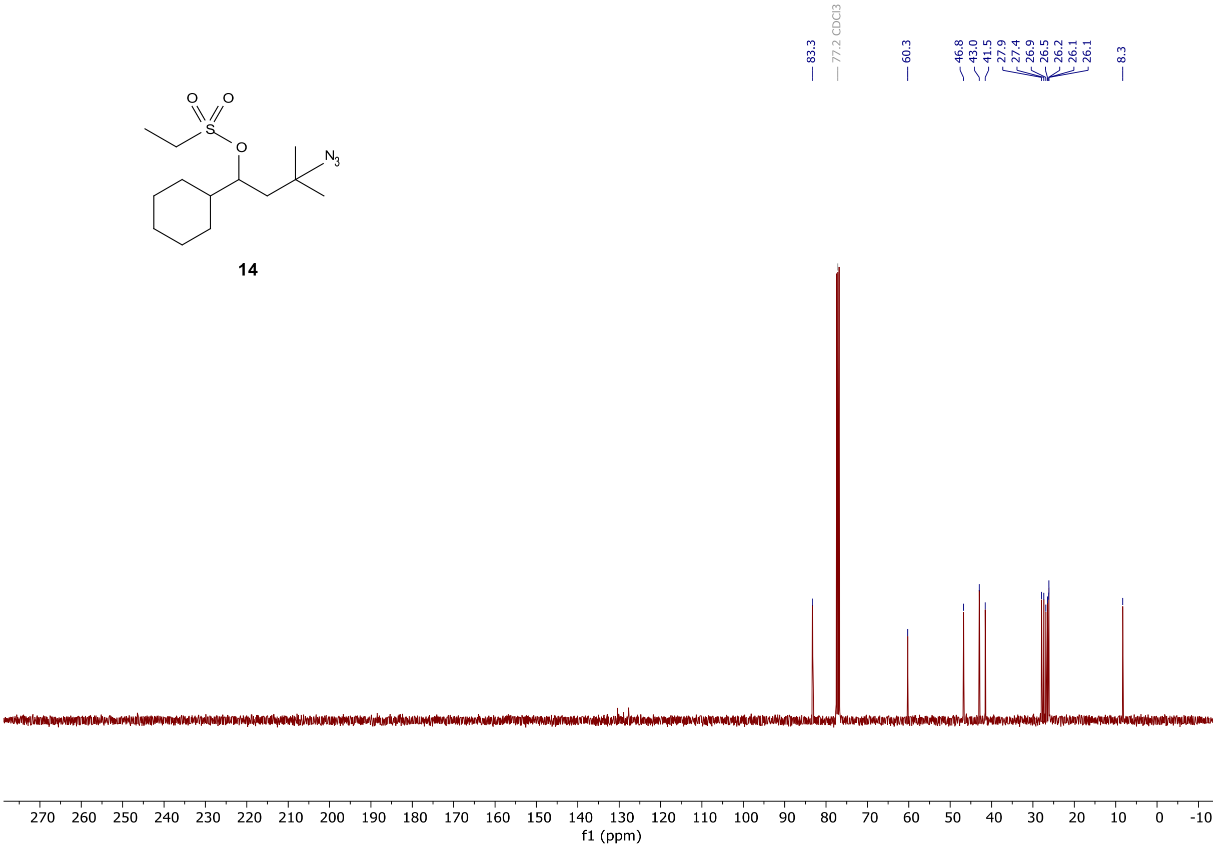
83.3
77.2 CDCl3
60.3
46.8
43.0
41.5
27.9
27.4
26.9
26.5
26.2
26.1
26.1
8.3
N_3
14
270 260 250 240 230 220 210 200 190 180 170 160 150 140 130 120 110 100 90 80 70 60 50 40 30 20 10 0 -10
f1 (ppm)

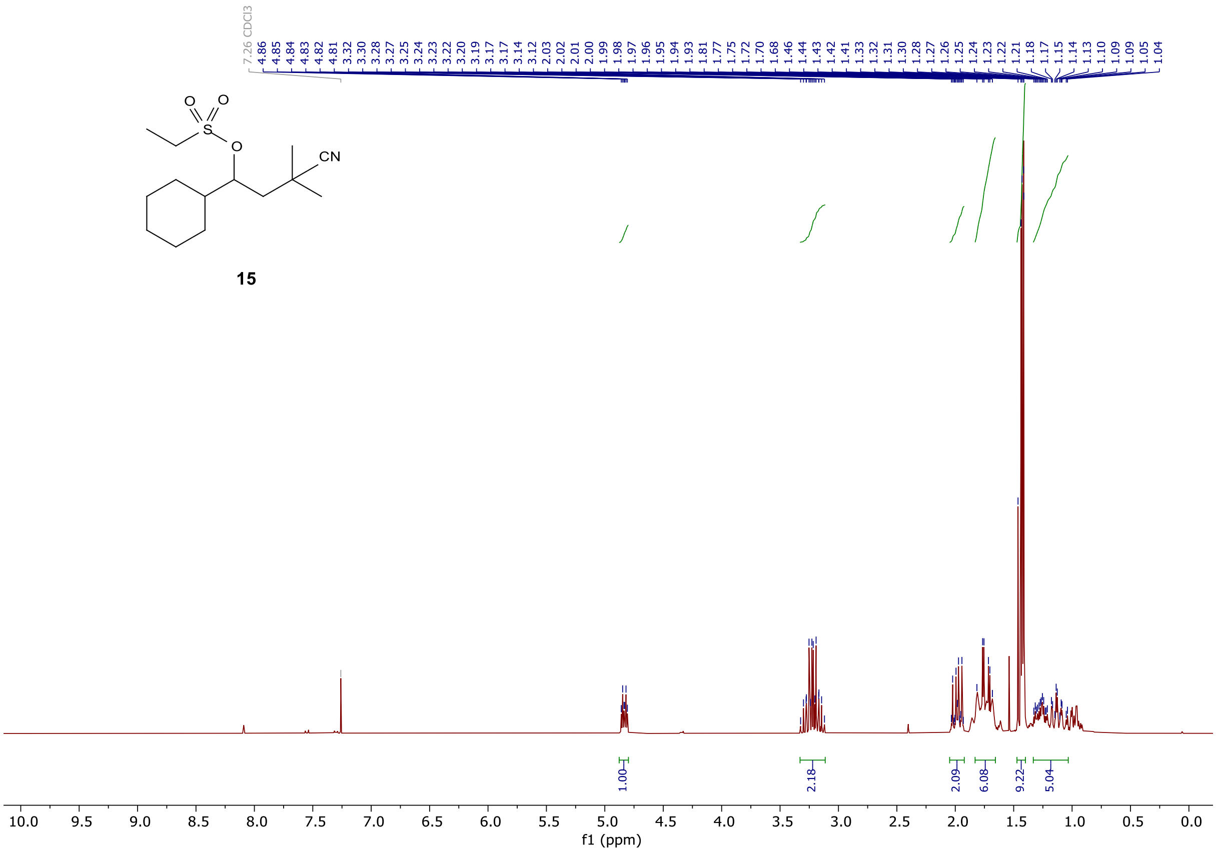
7.26 CDCl3
4.86
4.85
4.84
4.83
4.82
4.81
3.32
3.30
3.28
3.27
3.25
3.24
3.23
3.22
3.20
3.19
3.17
3.17
3.14
3.12
2.03
2.02
2.01
2.00
1.99
1.98
1.97
1.96
1.95
1.94
1.93
1.81
1.77
1.75
1.72
1.70
1.68
1.46
1.44
1.43
1.42
1.41
1.33
1.32
1.31
1.30
1.28
1.27
1.26
1.25
1.24
1.23
1.22
1.21
1.18
1.17
1.15
1.14
1.13
1.10
1.09
1.09
1.05
1.04
CN
15
1.00
2.18
2.09
6.08
9.22
5.04
10.0 9.5 9.0 8.5 8.0 7.5 7.0 6.5 6.0 5.5 5.0 4.5 4.0 3.5 3.0 2.5 2.0 1.5 1.0 0.5 0.0
f1 (ppm)

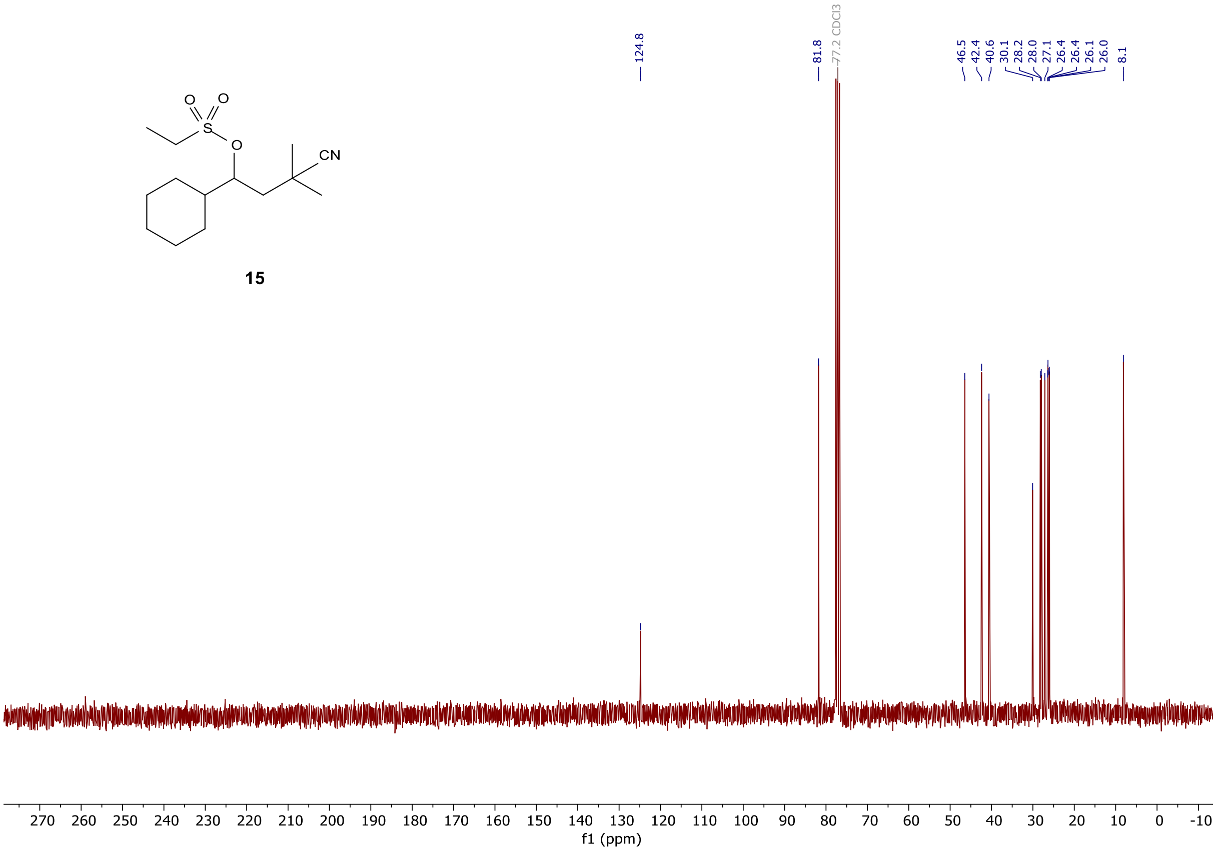
124.8
81.8
77.2 CDCl3
46.5
42.4
40.6
30.1
28.2
28.0
27.1
26.4
26.4
26.1
26.0
8.1
CN
15
270 260 250 240 230 220 210 200 190 180 170 160 150 140 130 120 110 100 90 80 70 60 50 40 30 20 10 0 -10
f1 (ppm)

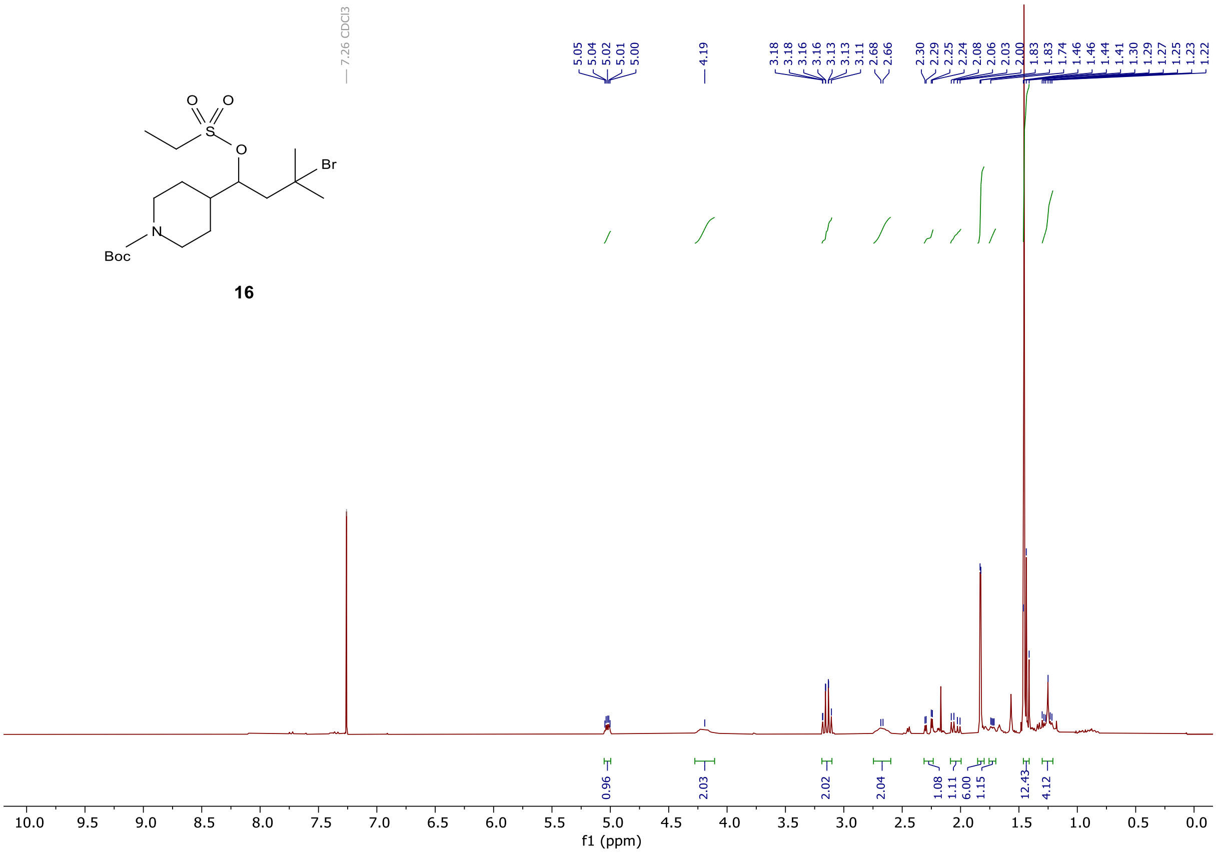
7.26 CDCl3
5.05
5.04
5.02
5.01
5.00
4.19
3.18
3.18
3.16
3.16
3.13
3.13
3.11
2.68
2.66
2.30
2.29
2.25
2.24
2.08
2.06
2.03
2.00
1.83
1.83
1.74
1.46
1.46
1.44
1.41
1.30
1.29
1.27
1.25
1.23
1.22
Br
N
Boc
16
0.96
2.03
2.02
2.04
1.08
1.11
6.00
1.15
12.43
4.12
f1 (ppm)

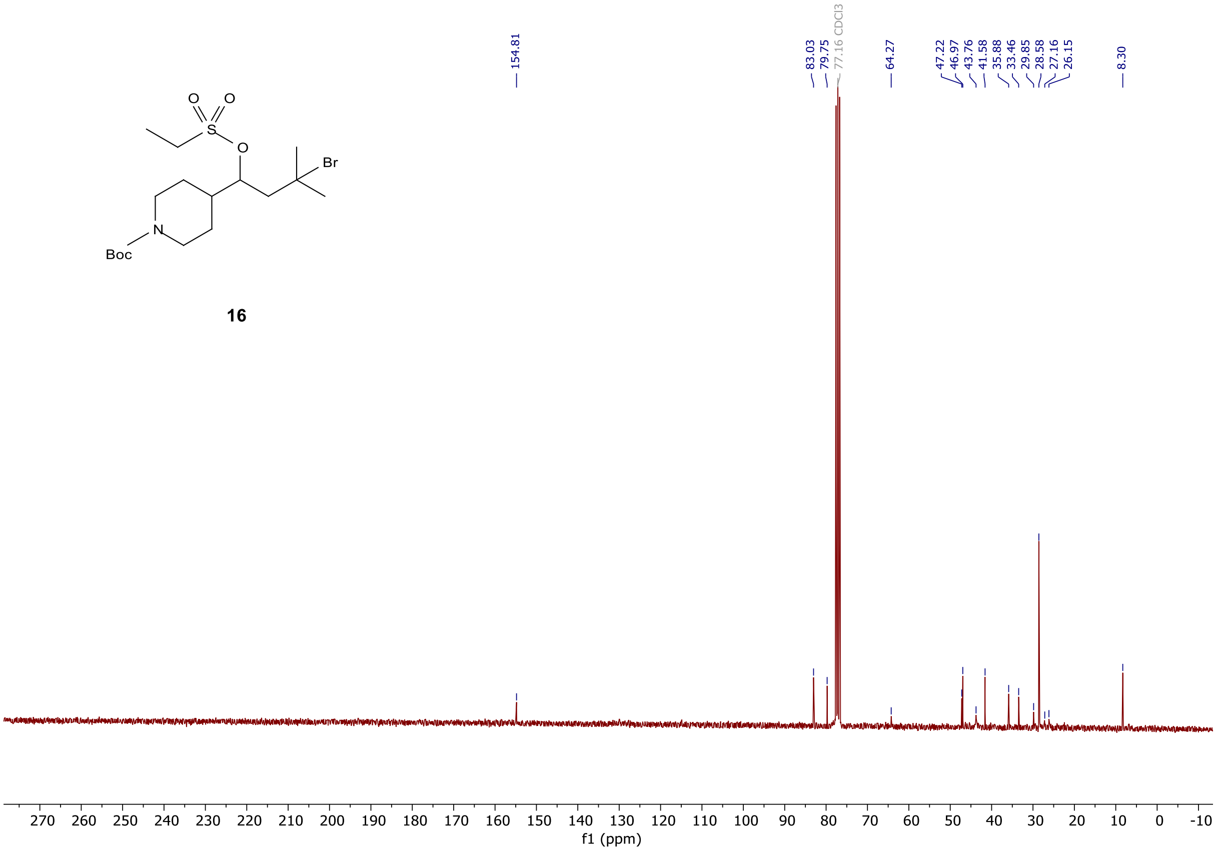
154.81
83.03
79.75
77.16 CDCl3
64.27
47.22
46.97
43.76
41.58
35.88
33.46
29.85
28.58
27.16
26.15
8.30
Br
N
Boc
16
f1 (ppm)

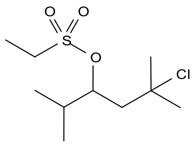
O
O
S
O
Cl

17

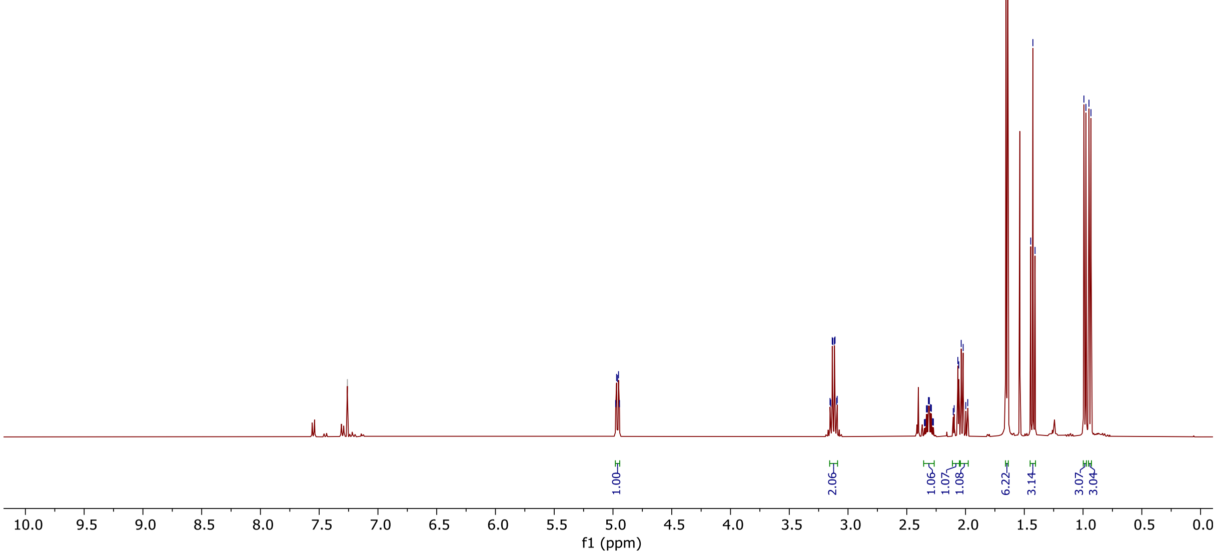
7.26 CDCl3
4.98
4.97
4.96
4.95
4.94
3.15
3.15
3.13
3.13
3.12
3.11
3.10
3.09
2.33
2.32
2.32
2.31
2.30
2.29
2.10
2.07
2.06
2.04
2.02
2.00
1.98
1.66
1.64
1.45
1.43
1.41
0.99
0.98
0.95
0.93
1.00
2.06
1.06
1.07
1.08
6.22
3.14
3.07
3.04
10.0 9.5 9.0 8.5 8.0 7.5 7.0 6.5 6.0 5.5 5.0 4.5 4.0 3.5 3.0 2.5 2.0 1.5 1.0 0.5 0.0
f1 (ppm)

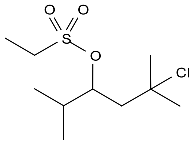
O
O
S
O
Cl

17

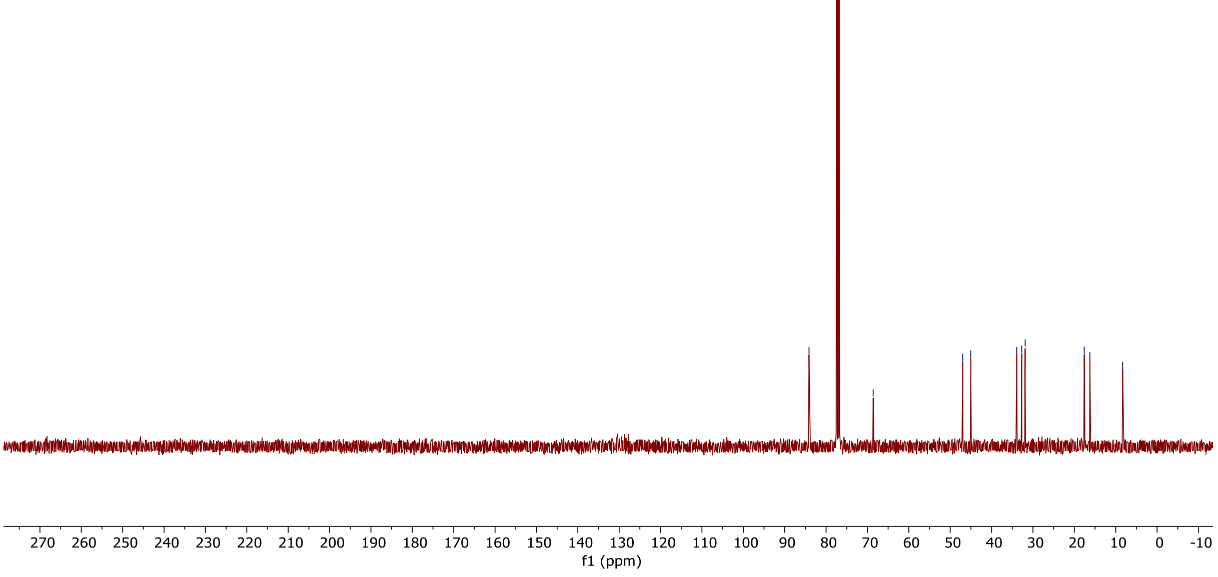
84.1
77.2 CDCl3
68.6
47.0
45.0
33.9
32.7
31.9
17.6
16.2
8.3
270 260 250 240 230 220 210 200 190 180 170 160 150 140 130 120 110 100 90 80 70 60 50 40 30 20 10 0 -10
f1 (ppm)

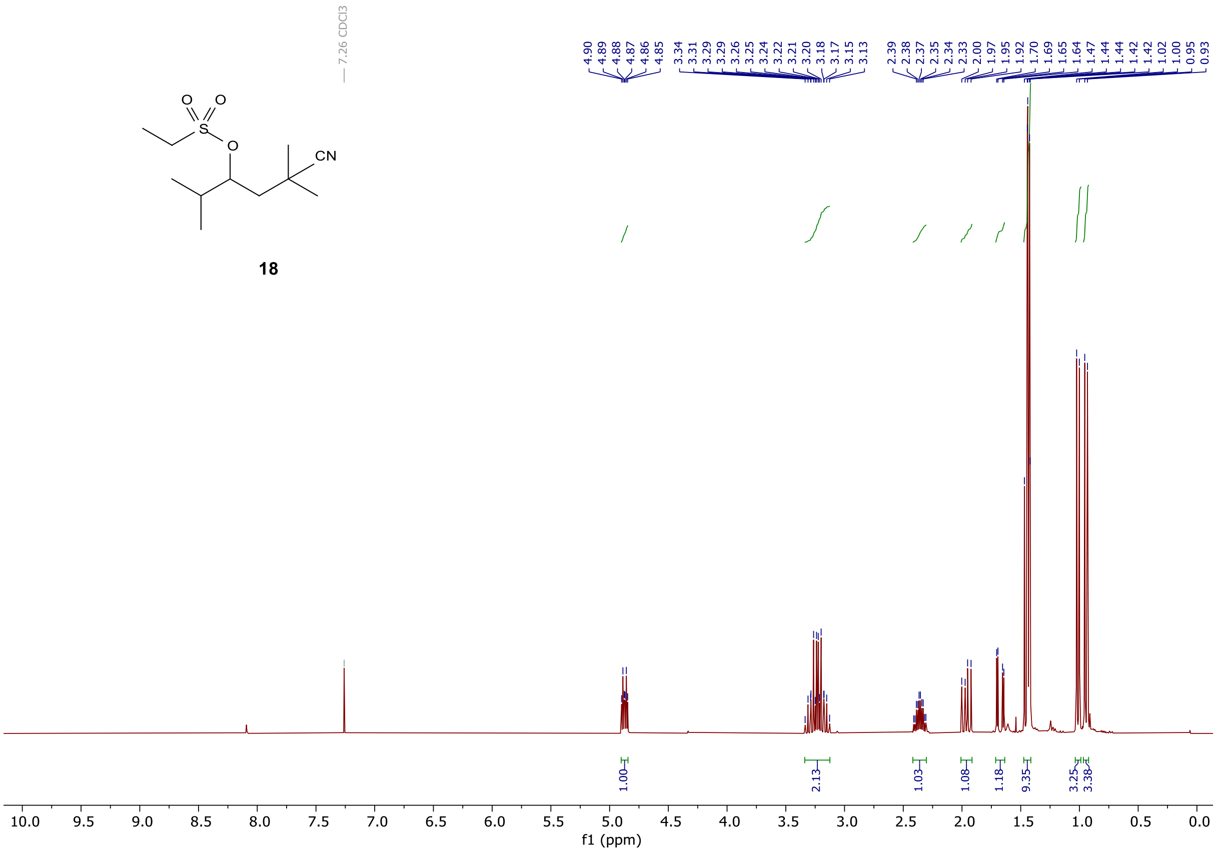

CN
7.26 CDCl3
4.90 4.89 4.88 4.87 4.86 4.85
3.34 3.31 3.29 3.29 3.26 3.25 3.24 3.22 3.21 3.20 3.18 3.17 3.15 3.13
2.39 2.38 2.37 2.35 2.34 2.33 2.00 1.97 1.95 1.92 1.70 1.69 1.65 1.64 1.47 1.44 1.44 1.42 1.42 1.02 1.00 0.95 0.93
1.00 2.13 1.03 1.08 1.18 9.35 3.25 3.38
10.0 9.5 9.0 8.5 8.0 7.5 7.0 6.5 6.0 5.5 5.0 4.5 4.0 3.5 3.0 2.5 2.0 1.5 1.0 0.5 0.0
f1 (ppm)

18

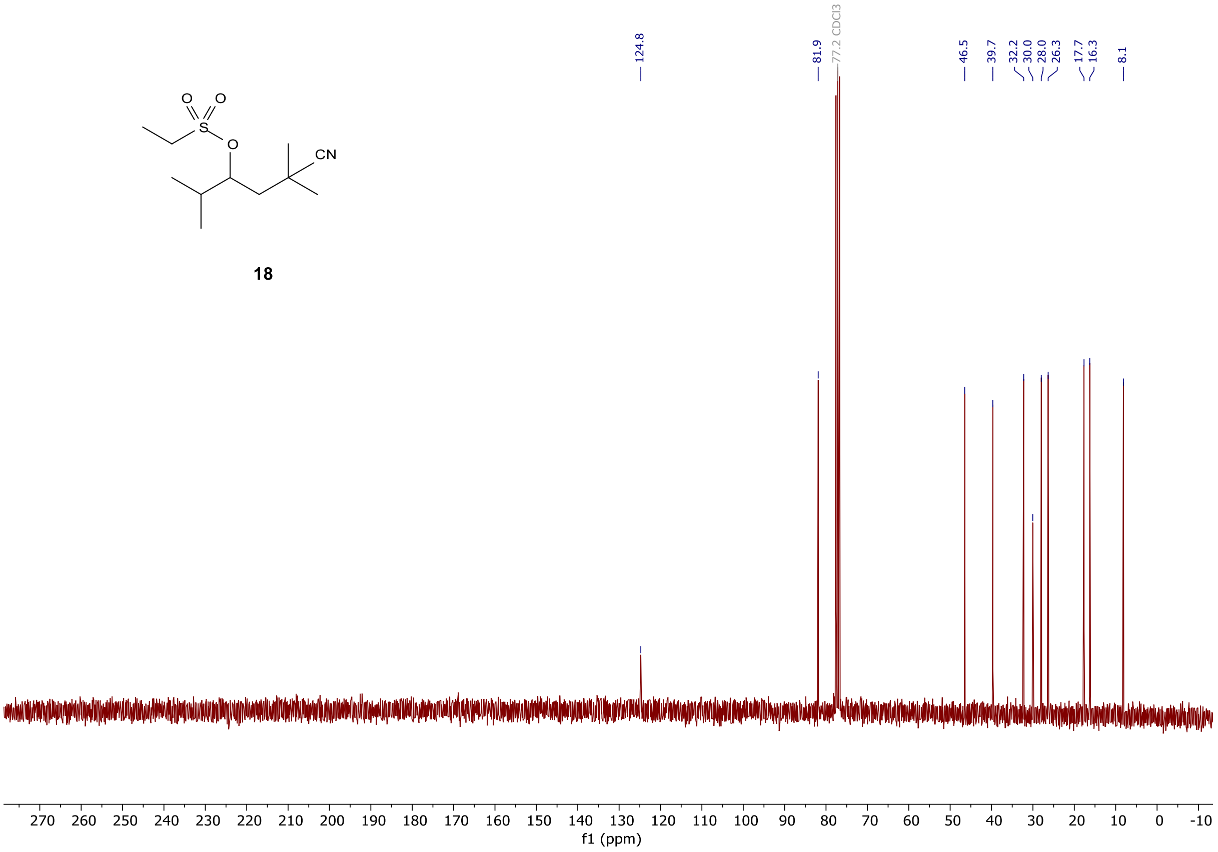

CN
124.8
81.9
77.2 CDCl3
46.5 39.7 32.2 30.0 28.0 26.3 17.7 16.3 8.1
270 260 250 240 230 220 210 200 190 180 170 160 150 140 130 120 110 100 90 80 70 60 50 40 30 20 10 0 -10
f1 (ppm)

18

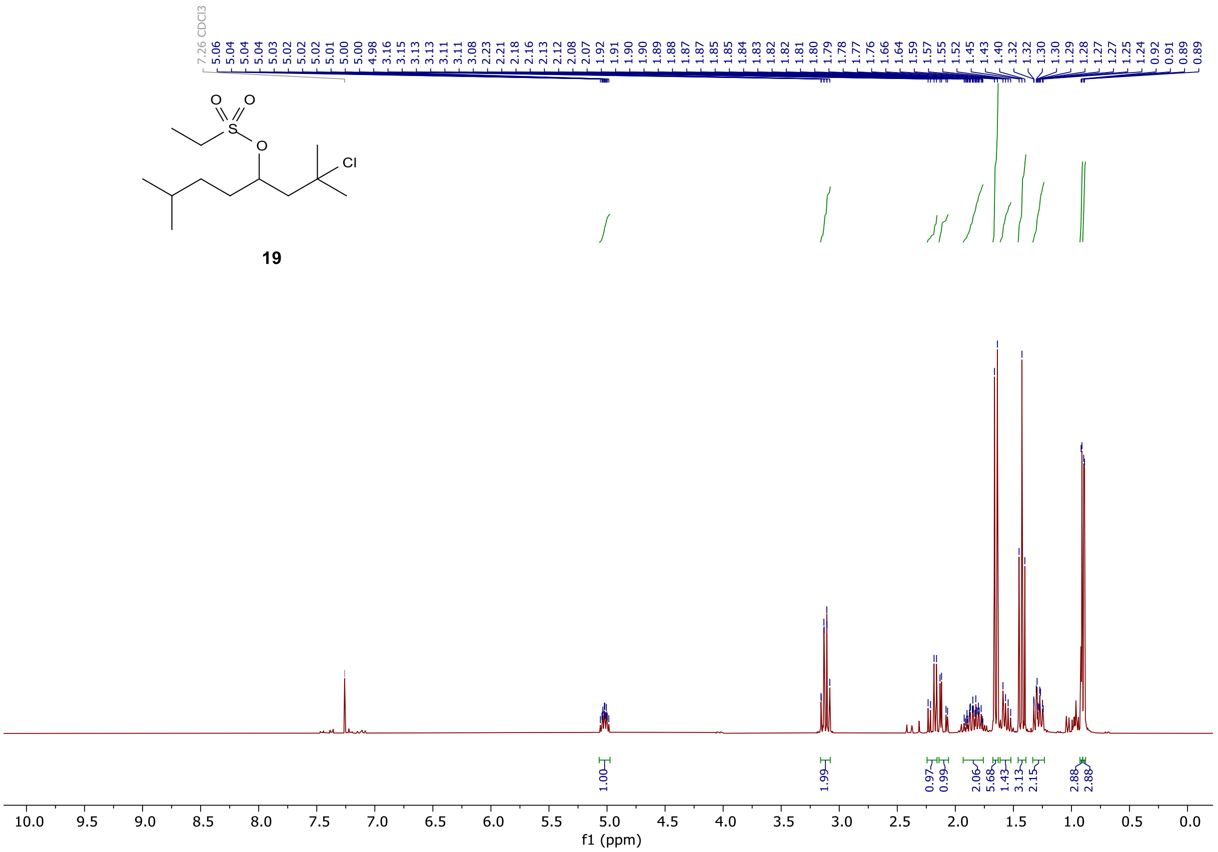
7.26 CDCl3
5.06 5.04 5.04 5.03 5.02 5.02 5.01 5.00 4.98 3.16 3.15 3.13 3.13 3.11 3.11 3.08 2.23 2.21 2.18 2.16 2.13 2.12 2.08 2.07 1.92 1.91 1.90 1.90 1.89 1.88 1.87 1.85 1.85 1.84 1.83 1.82 1.82 1.81 1.80 1.79 1.78 1.77 1.76 1.66 1.64 1.59 1.57 1.55 1.52 1.45 1.43 1.40 1.32 1.32 1.30 1.30 1.29 1.28 1.27 1.25 1.24 0.92 0.91 0.89 0.89
O O S O Cl
19
1.00 1.99 0.97 0.99 2.06 5.68 1.43 3.13 2.15 2.88 2.88
10.0 9.5 9.0 8.5 8.0 7.5 7.0 6.5 6.0 5.5 5.0 4.5 4.0 3.5 3.0 2.5 2.0 1.5 1.0 0.5 0.0
f1 (ppm)

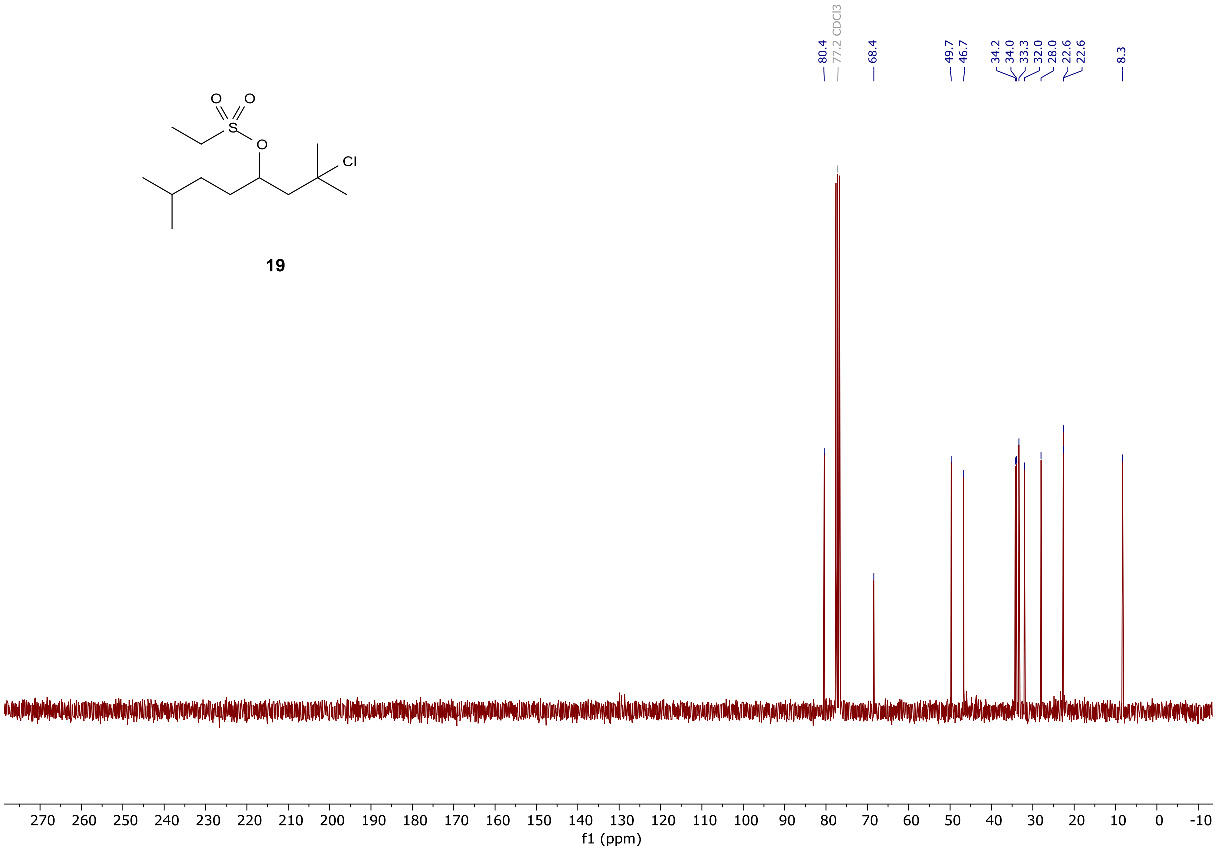
80.4
77.2 CDCl3
68.4
49.7
46.7
34.2 34.0 33.3 32.0 28.0 22.6 22.6
8.3
O O S O Cl
19
270 260 250 240 230 220 210 200 190 180 170 160 150 140 130 120 110 100 90 80 70 60 50 40 30 20 10 0 -10
f1 (ppm)

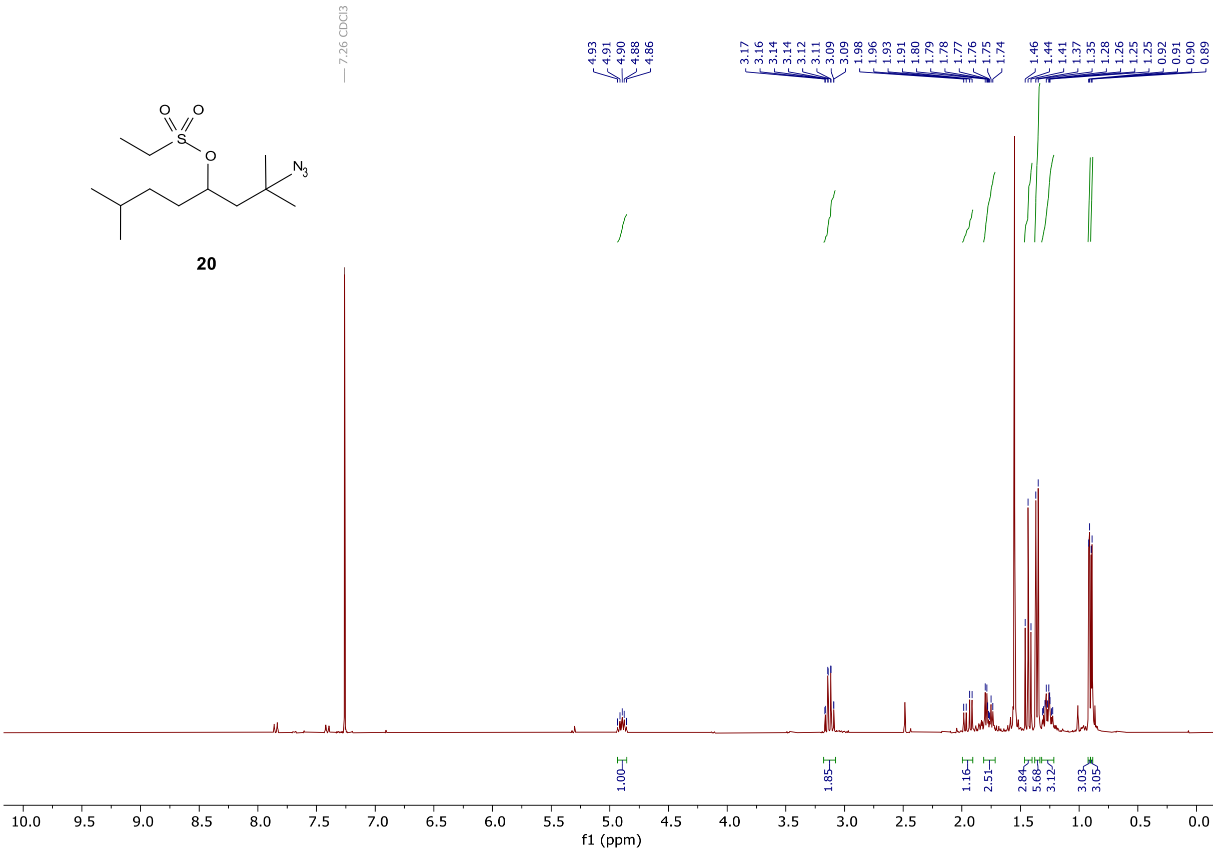

20
7.26 CDCl3
4.93
4.91
4.90
4.88
4.86
3.17
3.16
3.14
3.14
3.12
3.11
3.09
3.09
1.98
1.96
1.93
1.91
1.80
1.79
1.78
1.77
1.76
1.75
1.74
1.46
1.44
1.41
1.37
1.35
1.28
1.26
1.25
1.25
0.92
0.91
0.90
0.89
1.00
1.85
1.16
2.51
2.84
5.68
3.12
3.03
3.05
10.0
9.5
9.0
8.5
8.0
7.5
7.0
6.5
6.0
5.5
5.0
4.5
4.0
3.5
3.0
2.5
2.0
1.5
1.0
0.5
0.0
f1 (ppm)

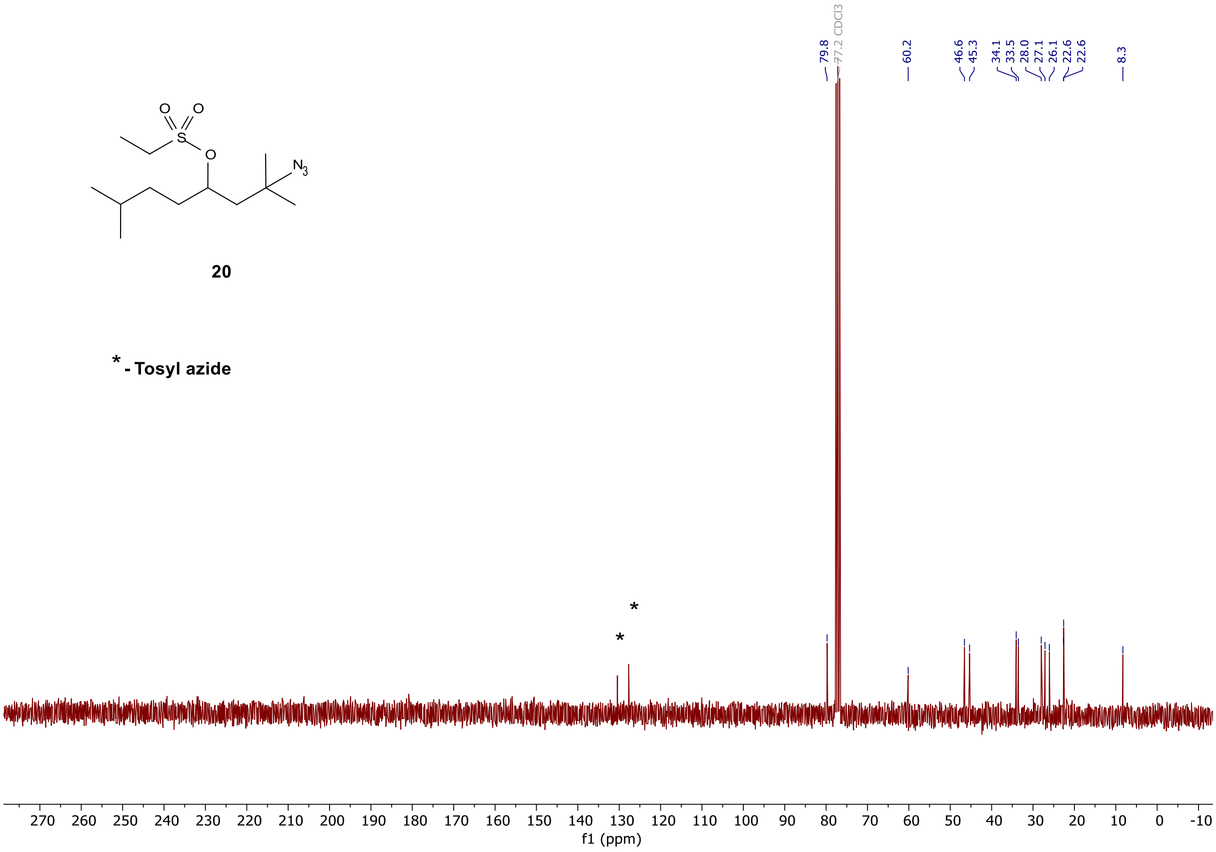

20
* - Tosyl azide
79.8
77.2 CDCl3
60.2
46.6
45.3
34.1
33.5
28.0
27.1
26.1
22.6
22.6
8.3
270
260
250
240
230
220
210
200
190
180
170
160
150
140
130
120
110
100
90
80
70
60
50
40
30
20
10
0
-10
f1 (ppm)

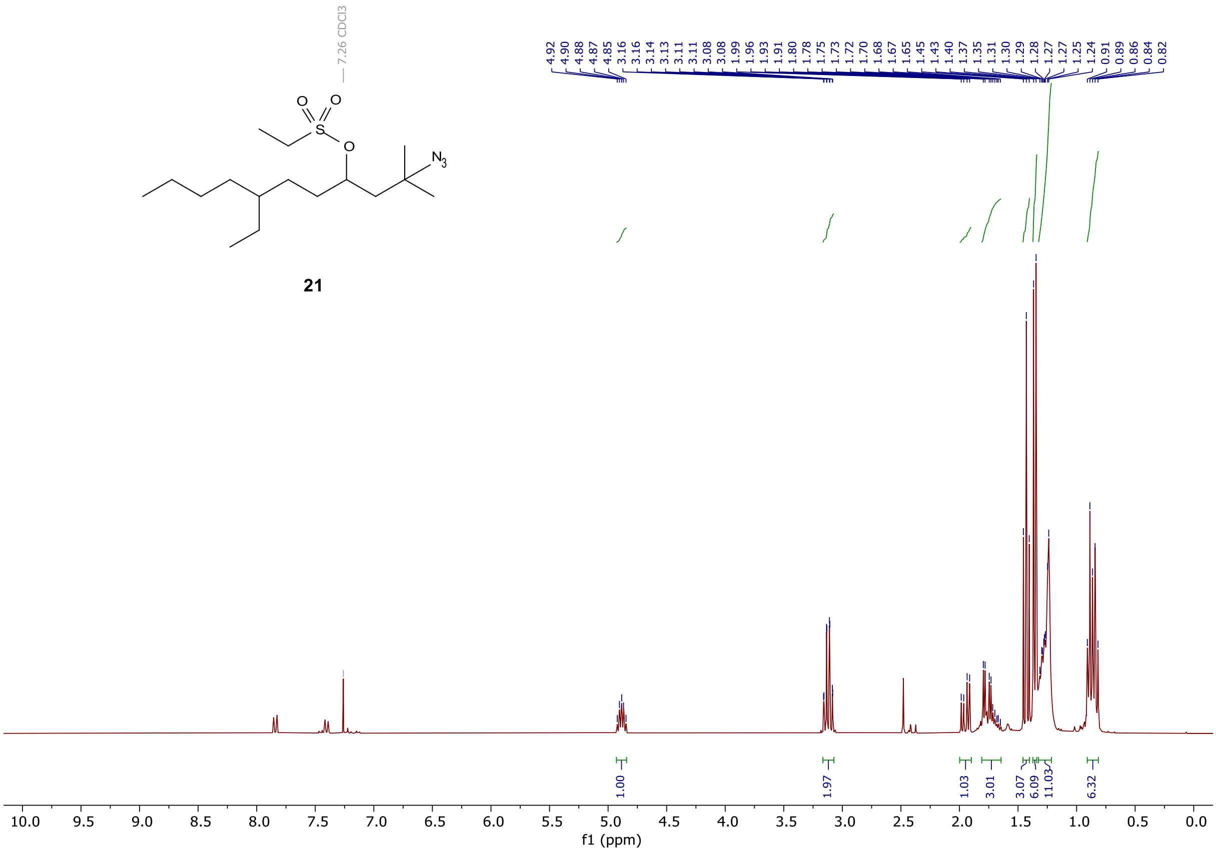
7.26 CDCl3
4.92
4.90
4.88
4.87
4.85
3.16
3.16
3.14
3.13
3.11
3.11
3.08
3.08
1.99
1.96
1.93
1.91
1.80
1.78
1.75
1.73
1.72
1.70
1.68
1.67
1.65
1.45
1.43
1.40
1.37
1.35
1.31
1.30
1.29
1.28
1.27
1.25
1.24
0.91
0.89
0.86
0.84
0.82
N3
O
S
21
1.00
1.97
1.03
3.01
3.07
6.09
11.03
6.32
f1 (ppm)

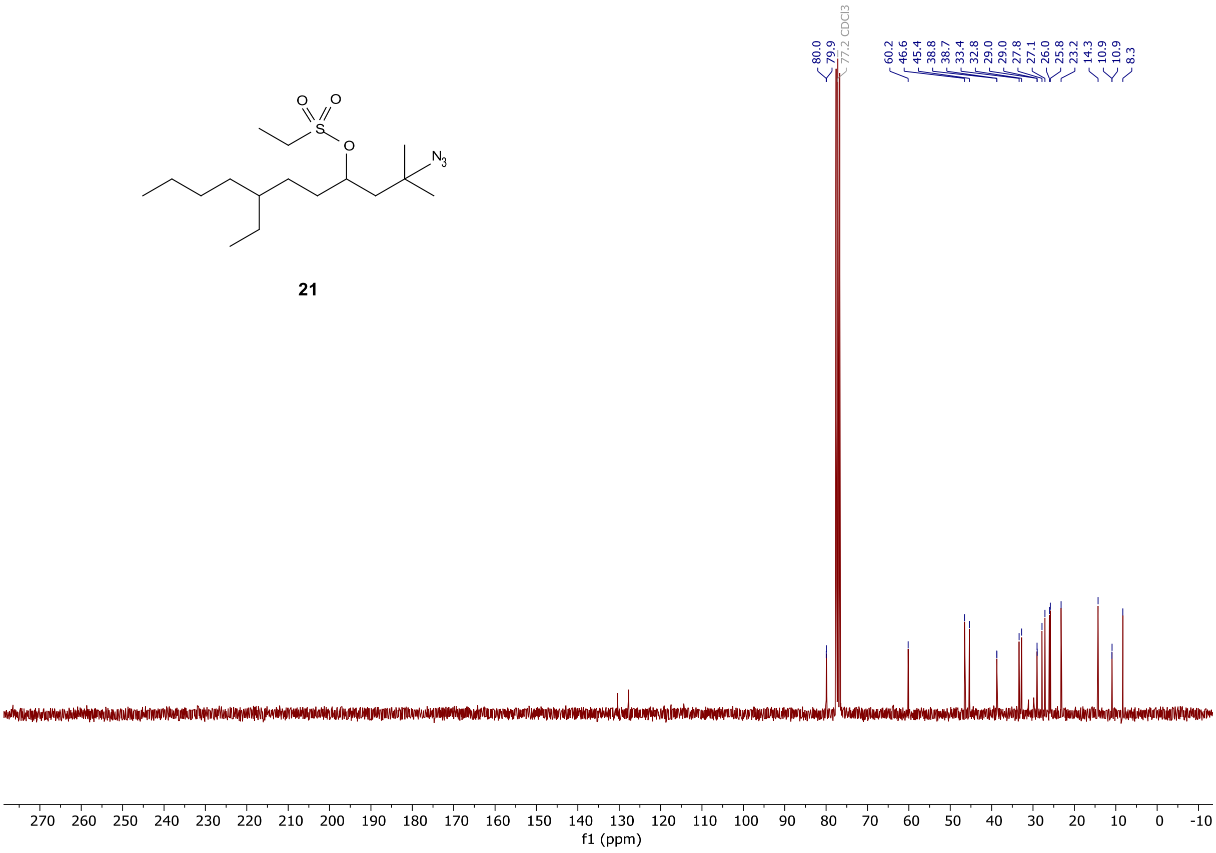
80.0
79.9
77.2 CDCl3
60.2
46.6
45.4
38.8
38.7
33.4
32.8
29.0
29.0
27.8
27.1
26.0
25.8
23.2
14.3
10.9
10.9
8.3
N3
O
S
21
f1 (ppm)

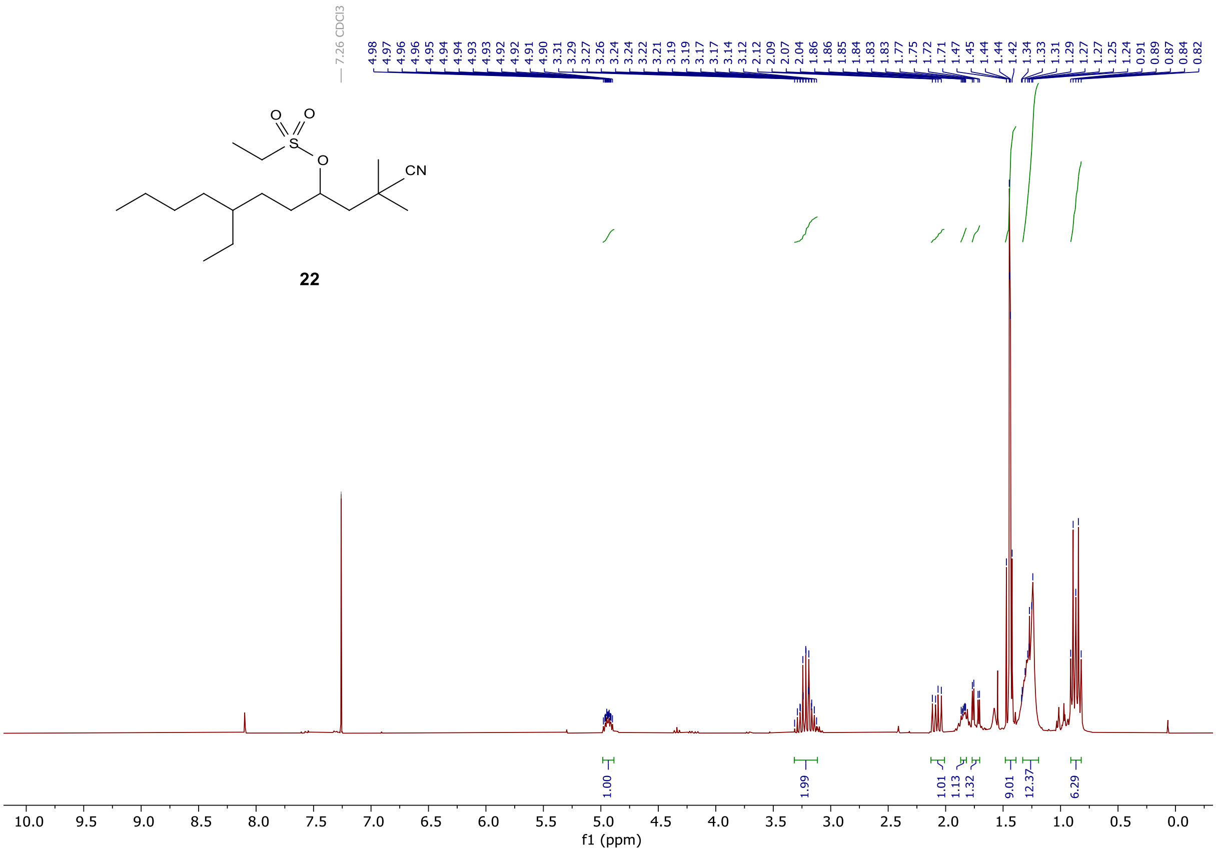
7.26 CDCl3
4.98 4.97 4.96 4.96 4.95 4.94 4.94 4.93 4.93 4.92 4.92 4.91 4.90 3.31 3.29 3.27 3.26 3.24 3.24 3.22 3.21 3.19 3.19 3.17 3.17 3.14 3.12 2.12 2.09 2.07 2.04 1.86 1.85 1.84 1.83 1.83 1.77 1.75 1.72 1.71 1.47 1.45 1.44 1.44 1.42 1.34 1.33 1.31 1.29 1.27 1.25 1.24 0.91 0.89 0.87 0.84 0.82
O
O
S
O
CN
22
1.00
1.99
1.01
1.13
1.32
9.01
12.37
6.29
10.0 9.5 9.0 8.5 8.0 7.5 7.0 6.5 6.0 5.5 5.0 4.5 4.0 3.5 3.0 2.5 2.0 1.5 1.0 0.5 0.0
f1 (ppm)

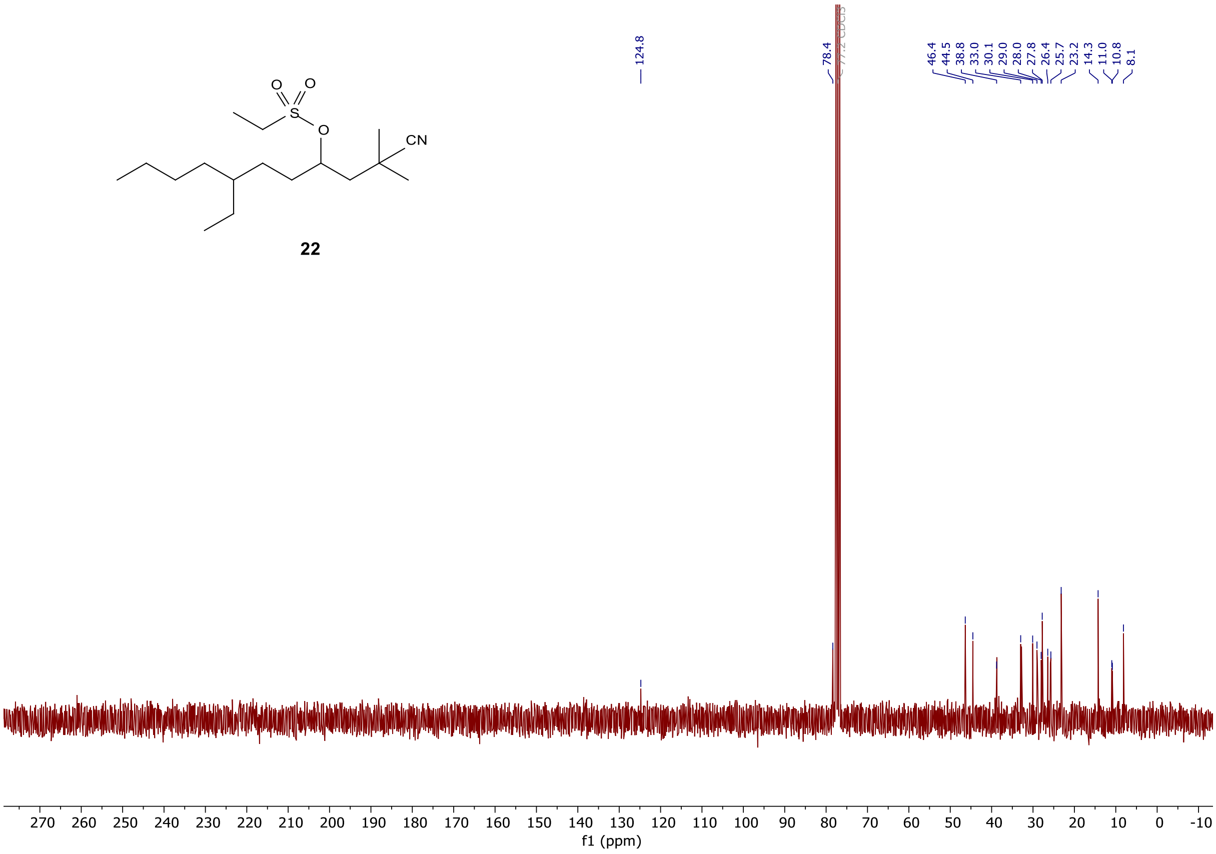
124.8
78.4
77.2 CDCl3
46.4 44.5 38.8 33.0 30.1 29.0 28.0 27.8 26.4 25.7 23.2 14.3 11.0 10.8 8.1
O
O
S
O
CN
22
270 260 250 240 230 220 210 200 190 180 170 160 150 140 130 120 110 100 90 80 70 60 50 40 30 20 10 0 -10
f1 (ppm)

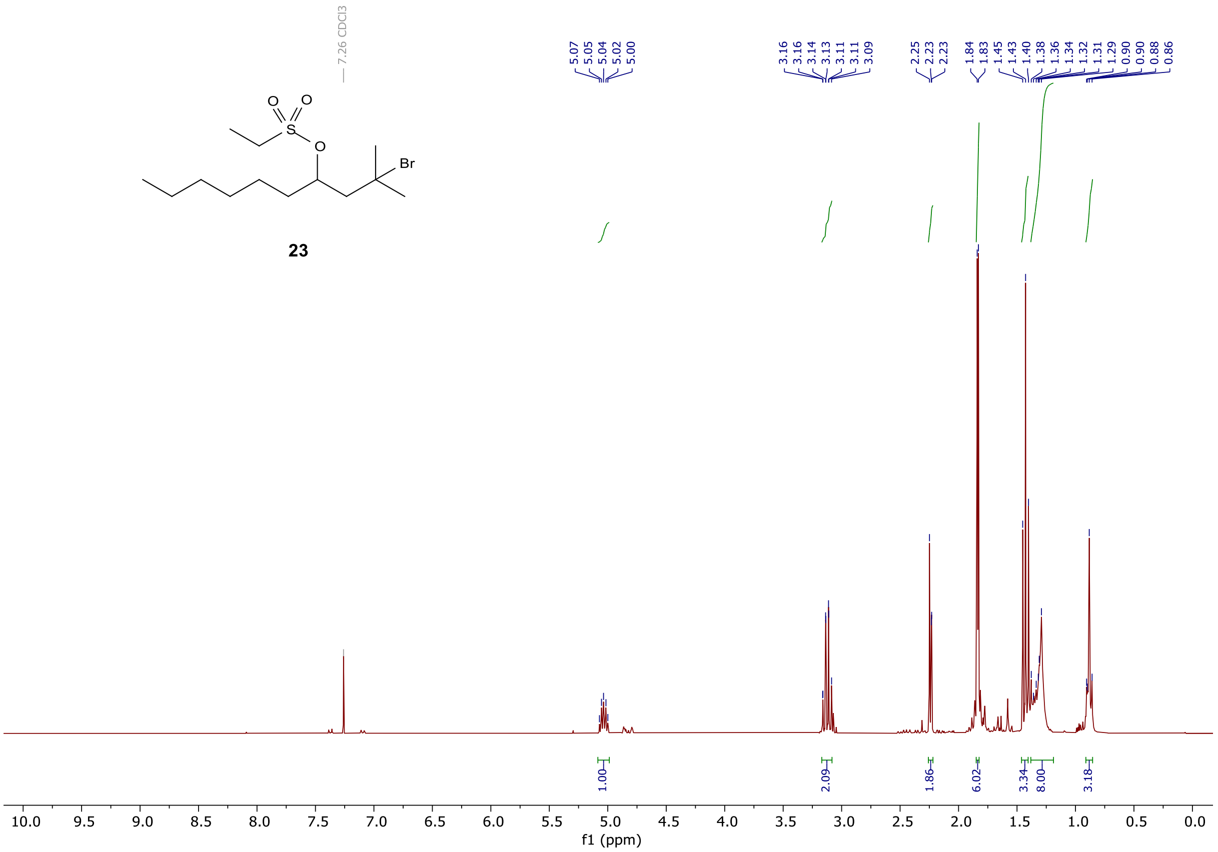
23
7.26 CDCl3
5.07 5.05 5.04 5.02 5.00
3.16 3.16 3.14 3.13 3.11 3.11 3.09
2.25 2.23 2.23
1.84 1.83
1.45 1.43 1.40 1.38 1.36 1.34 1.32 1.31 1.29 0.90 0.90 0.88 0.86
1.00 2.09 1.86 6.02 3.34 8.00 3.18
f1 (ppm)

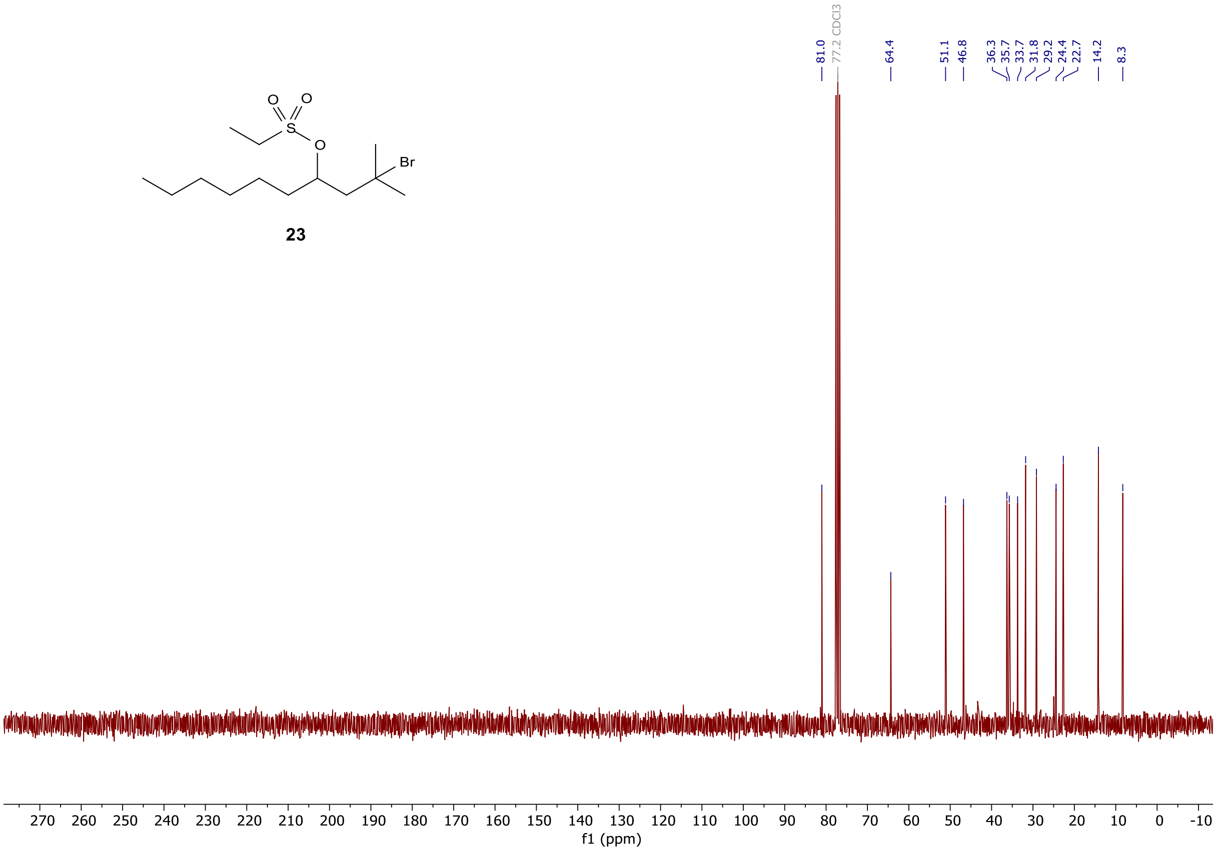
23
81.0
77.2 CDCl3
64.4
51.1
46.8
36.3 35.7 33.7 31.8 29.2 24.4 22.7
14.2
8.3
f1 (ppm)

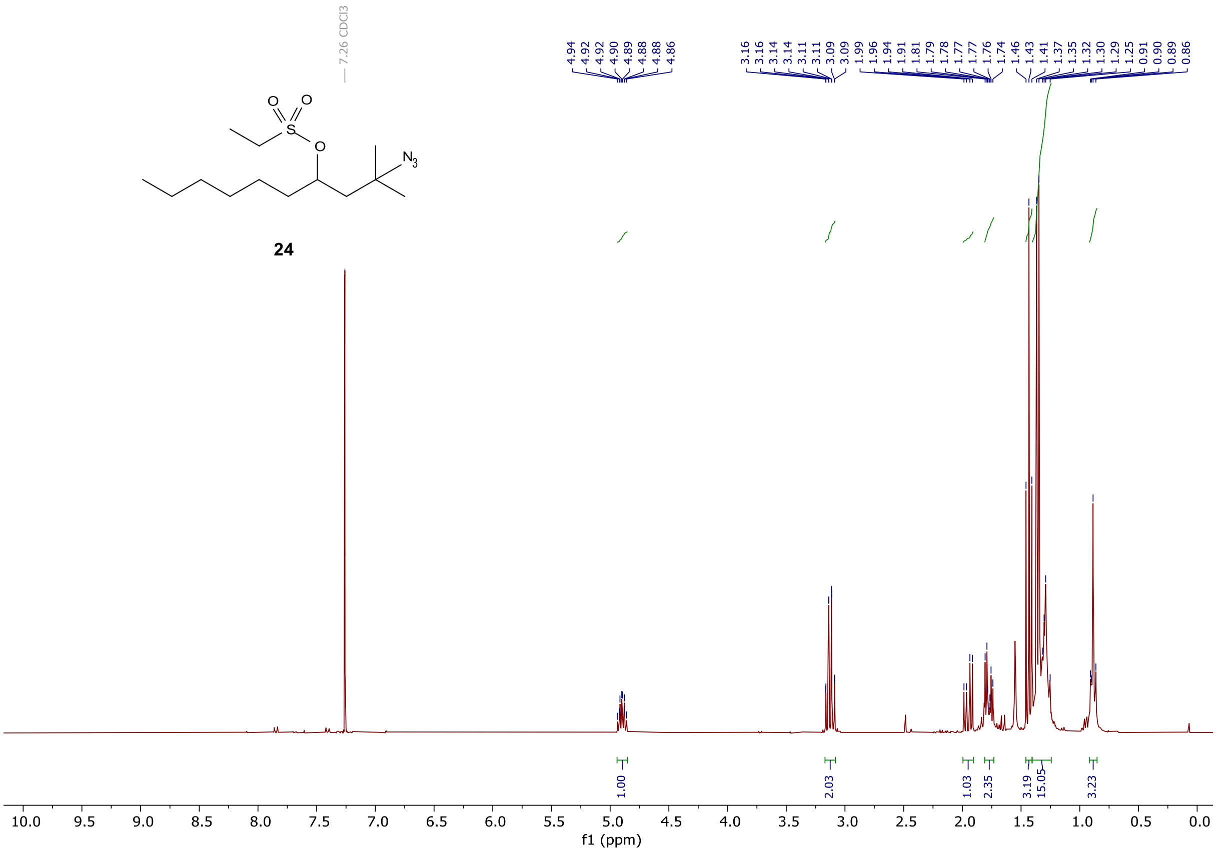
24
N3
7.26 CDCl3
4.94 4.92 4.92 4.90 4.89 4.88 4.88 4.86
3.16 3.16 3.14 3.14 3.11 3.11 3.09 3.09
1.99 1.96 1.94 1.91 1.81 1.79 1.78 1.77 1.77 1.76 1.74
1.46 1.43 1.41 1.37 1.35 1.32 1.30 1.29 1.25
0.91 0.90 0.89 0.86
1.00
2.03
1.03
2.35
3.19
15.05
3.23
10.0 9.5 9.0 8.5 8.0 7.5 7.0 6.5 6.0 5.5 5.0 4.5 4.0 3.5 3.0 2.5 2.0 1.5 1.0 0.5 0.0
f1 (ppm)

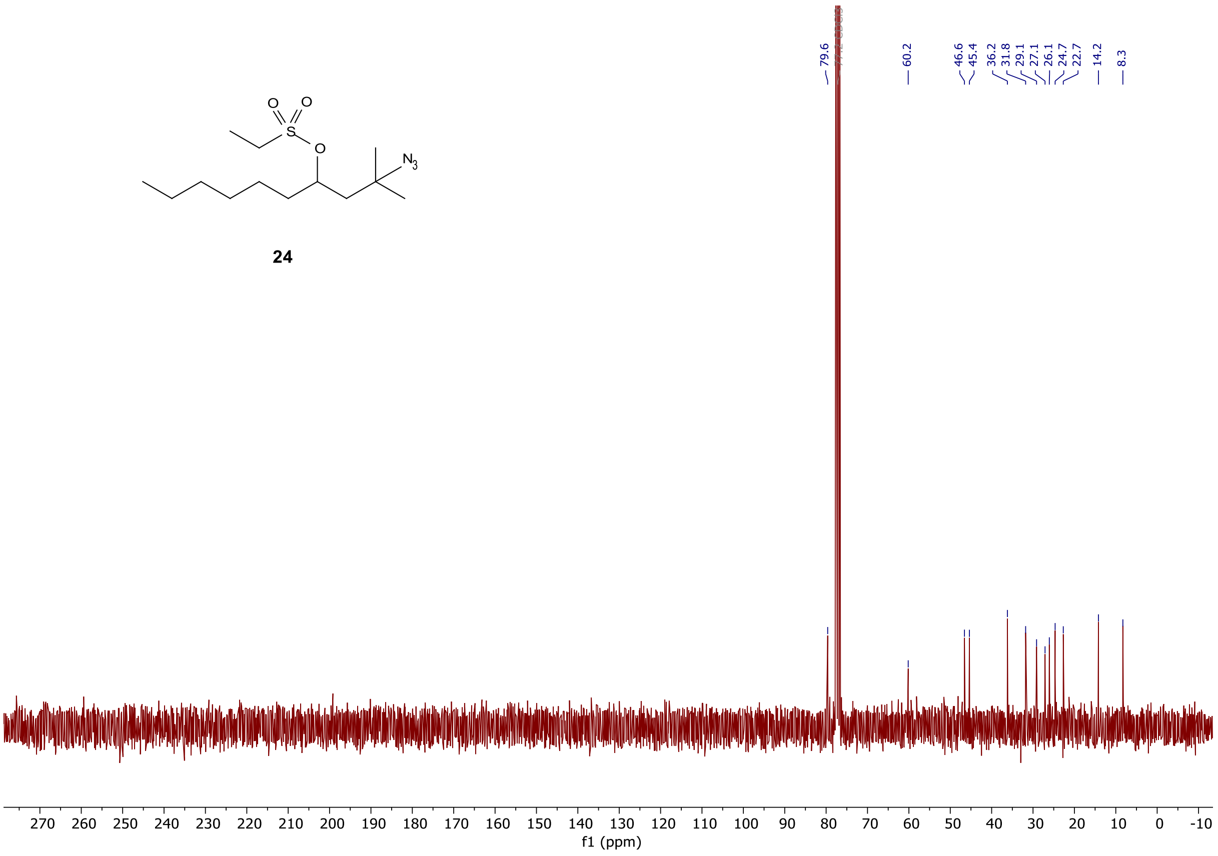
24
N3
79.6
60.2
46.6 45.4
36.2 31.8 29.1 27.1 26.1 24.7 22.7
14.2
8.3
270 260 250 240 230 220 210 200 190 180 170 160 150 140 130 120 110 100 90 80 70 60 50 40 30 20 10 0 -10
f1 (ppm)

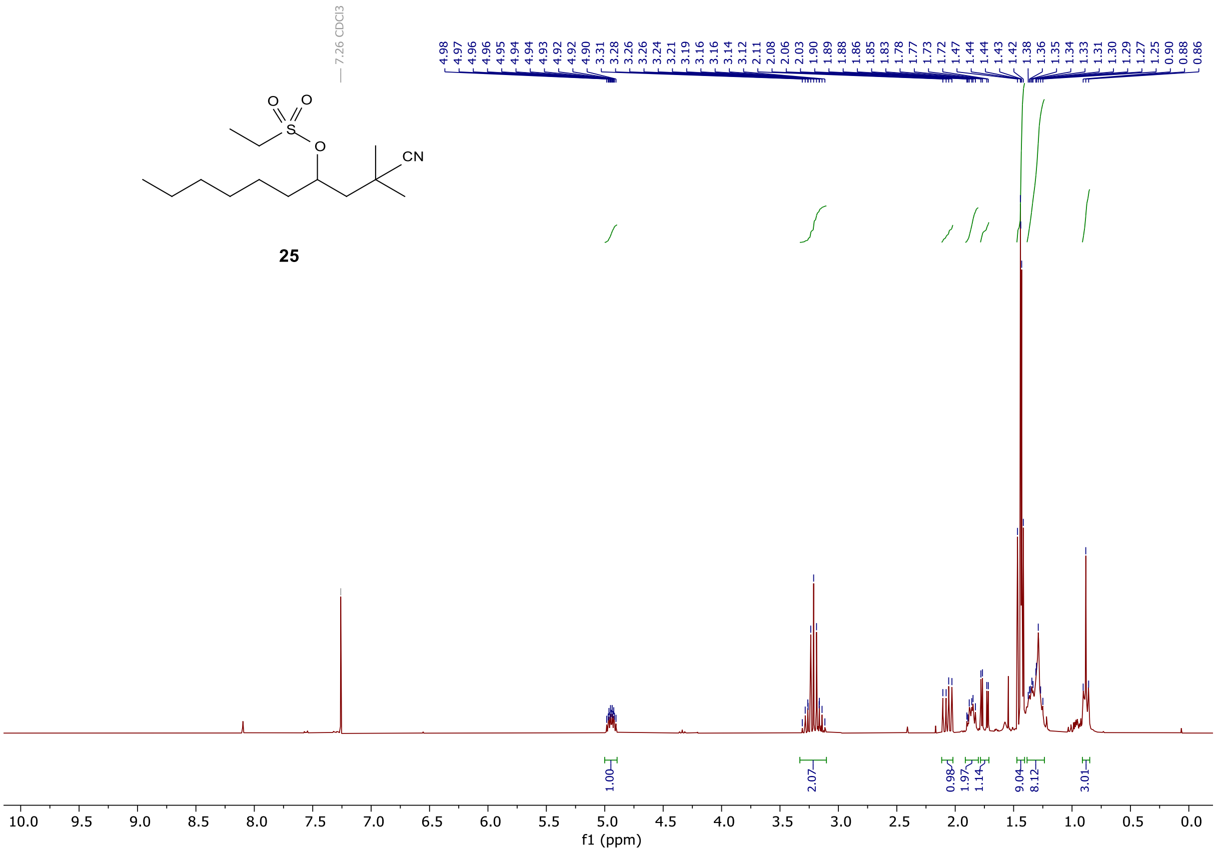

O O S O CN
25
7.26 CDCl3
4.98 4.97 4.96 4.96 4.95 4.94 4.94 4.93 4.92 4.92 4.90 3.31 3.28 3.26 3.26 3.24 3.21 3.19 3.16 3.16 3.14 3.12 2.11 2.08 2.06 2.03 1.90 1.89 1.88 1.86 1.85 1.83 1.78 1.77 1.73 1.72 1.47 1.44 1.44 1.43 1.42 1.38 1.36 1.35 1.34 1.33 1.31 1.30 1.29 1.27 1.25 0.90 0.88 0.86
1.00 2.07 0.98 1.97 1.14 9.04 8.12 3.01
10.0 9.5 9.0 8.5 8.0 7.5 7.0 6.5 6.0 5.5 5.0 4.5 4.0 3.5 3.0 2.5 2.0 1.5 1.0 0.5 0.0
f1 (ppm)

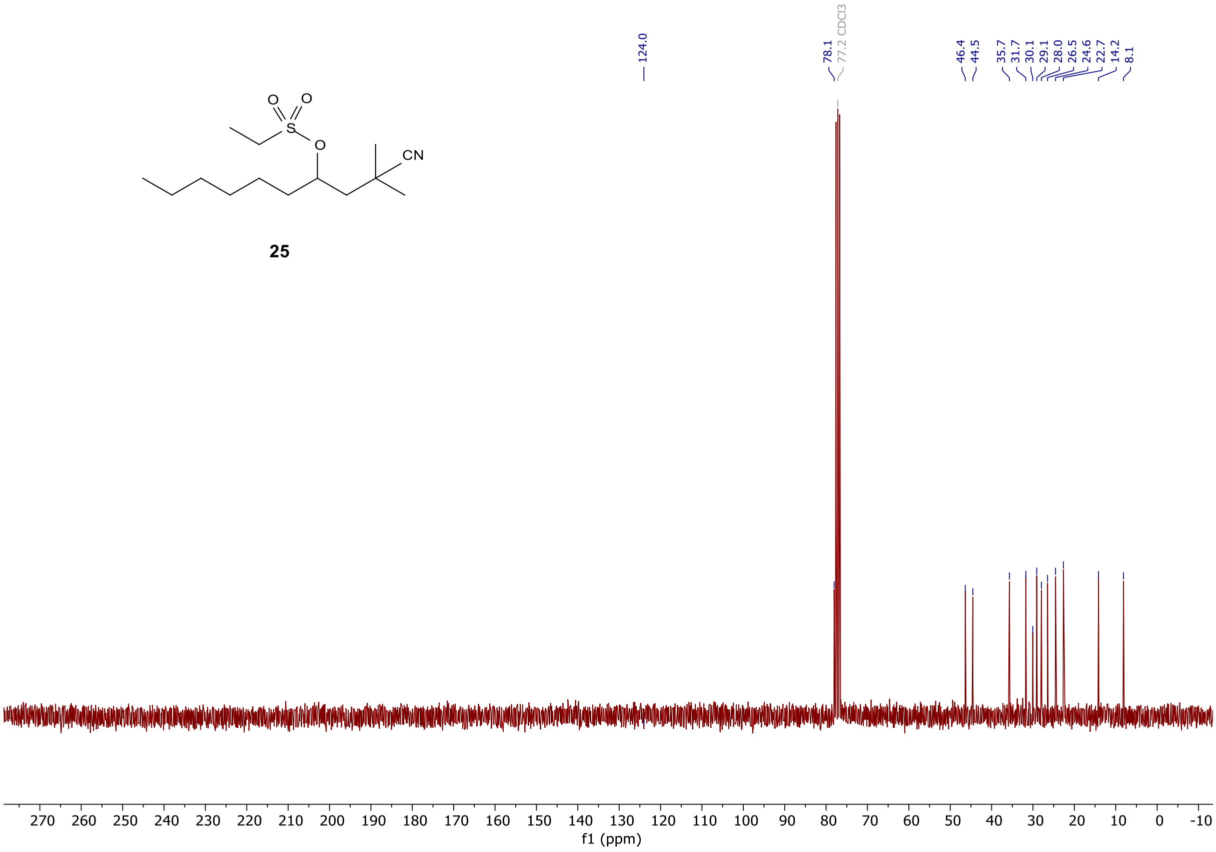

O O S O CN
25
124.0
78.1 77.2 CDCl3
46.4 44.5 35.7 31.7 30.1 29.1 28.0 26.5 24.6 22.7 14.2 8.1
270 260 250 240 230 220 210 200 190 180 170 160 150 140 130 120 110 100 90 80 70 60 50 40 30 20 10 0 -10
f1 (ppm)

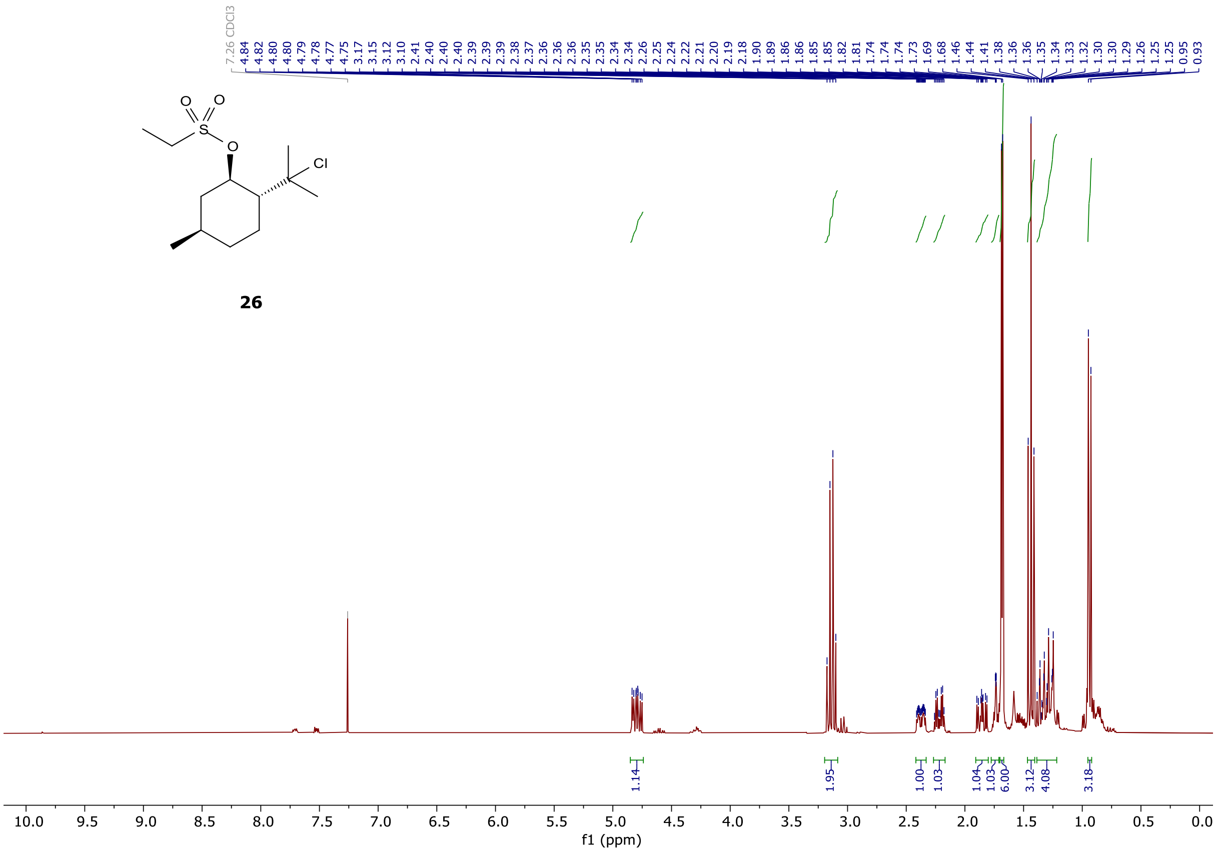

Cl
26
7.26 CDCl3
1.14
1.95
1.00
1.03
1.04
1.03
6.00
3.12
4.08
3.18
f1 (ppm)

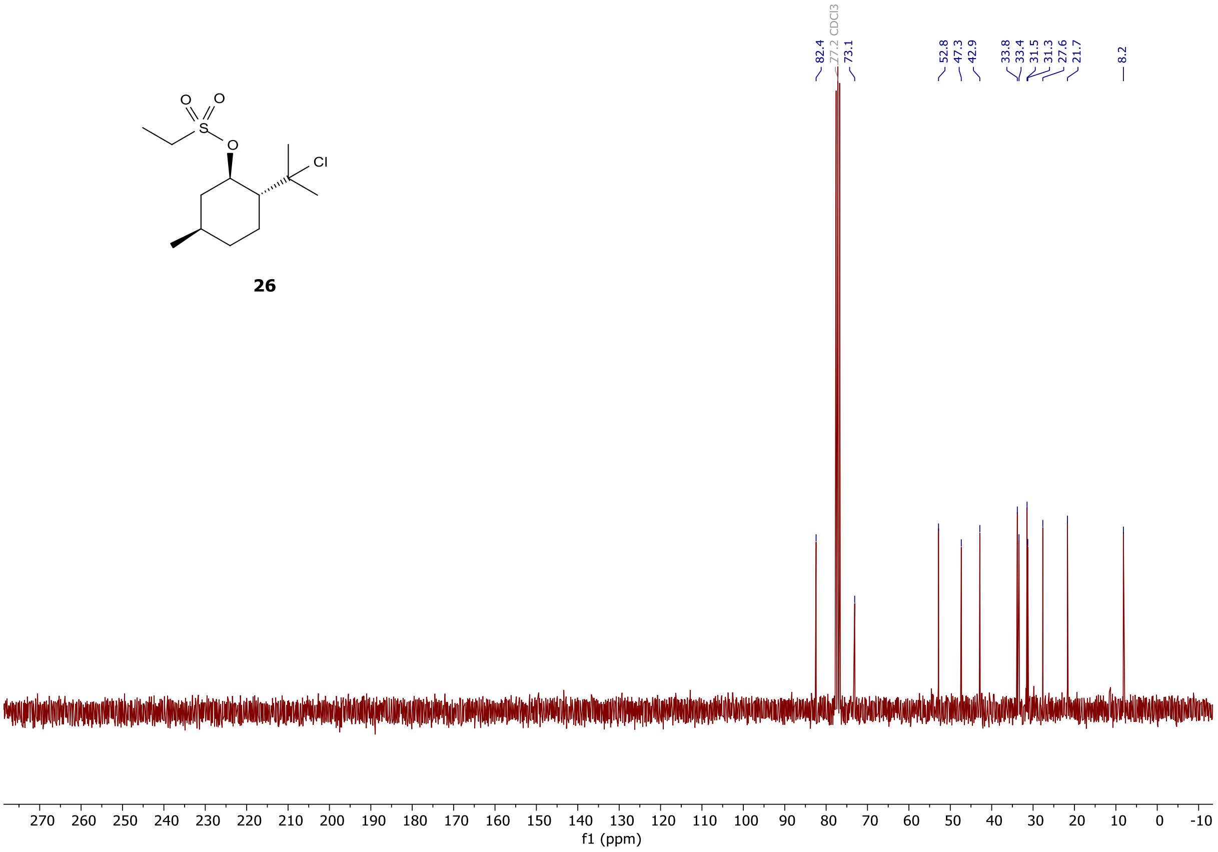

Cl
26
82.4
77.2 CDCl3
73.1
52.8
47.3
42.9
33.8
33.4
31.5
31.3
27.6
21.7
8.2
f1 (ppm)

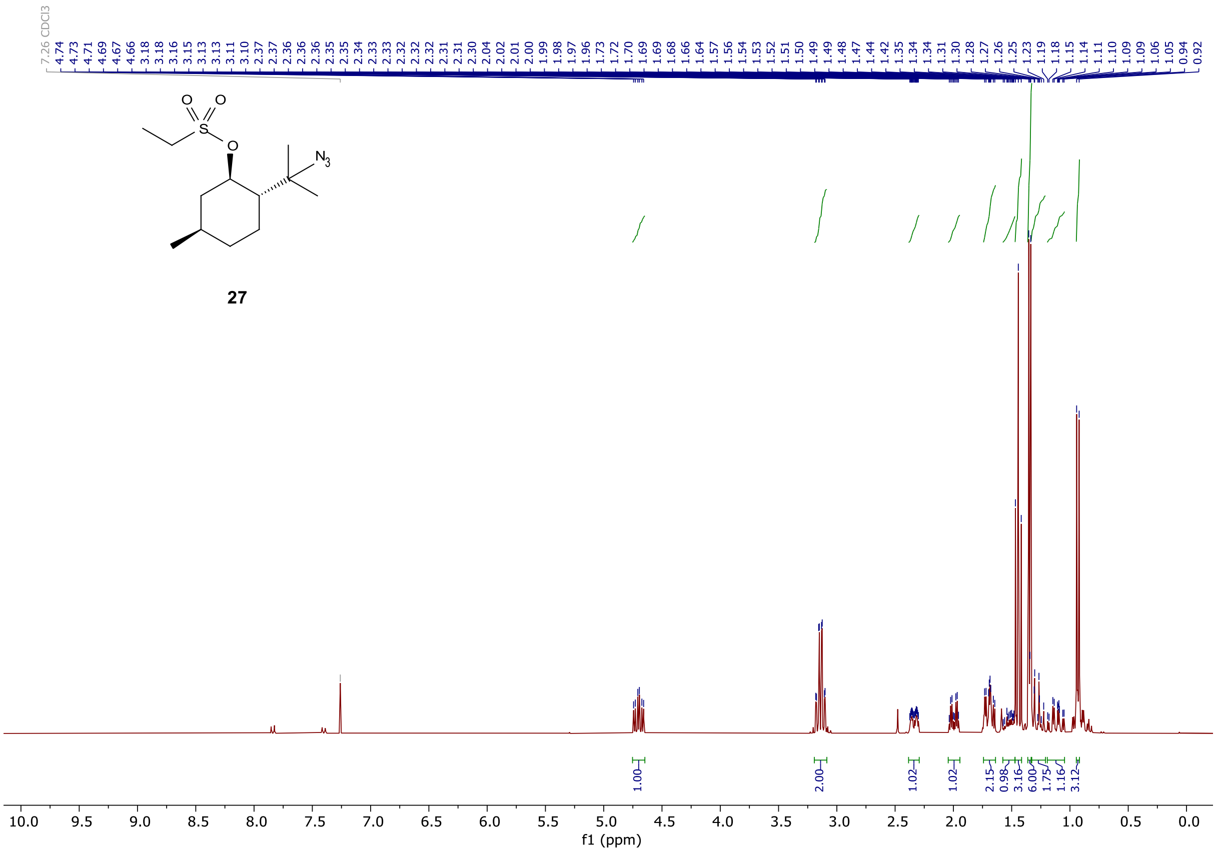

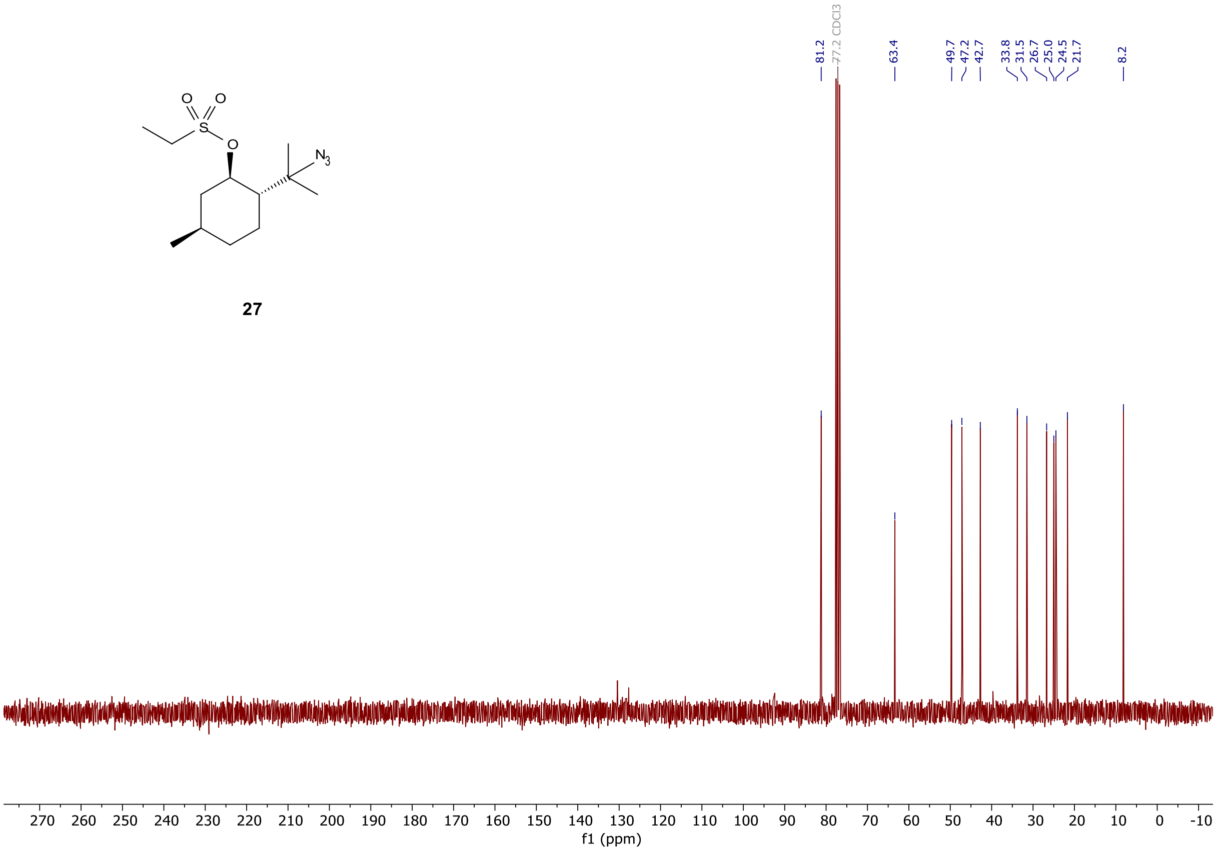

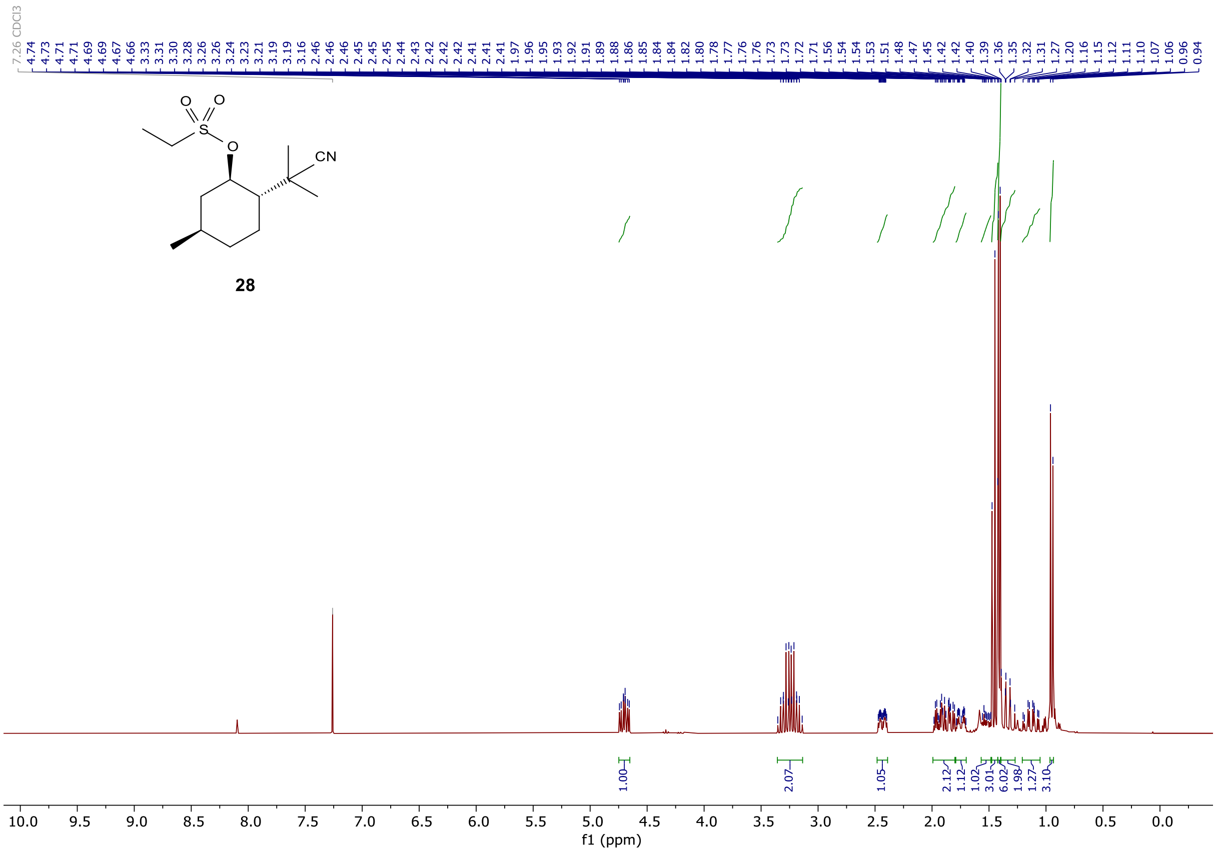
7.26 CDCl3
4.74 4.73 4.71 4.71 4.69 4.69 4.67 4.66 3.33 3.31 3.30 3.28 3.26 3.26 3.24 3.23 3.21 3.19 3.19 3.16 2.46 2.46 2.46 2.45 2.45 2.44 2.43 2.42 2.42 2.42 2.41 2.41 2.41 1.97 1.96 1.95 1.93 1.92 1.91 1.89 1.88 1.86 1.85 1.84 1.84 1.82 1.80 1.78 1.77 1.76 1.76 1.73 1.73 1.72 1.71 1.56 1.54 1.54 1.53 1.51 1.48 1.47 1.45 1.42 1.42 1.40 1.39 1.36 1.35 1.32 1.31 1.27 1.20 1.16 1.15 1.12 1.11 1.10 1.07 1.06 0.96 0.94
O
O
S
O
CN
28
1.00
2.07
1.05
2.12
1.12
1.02
3.01
6.02
1.98
1.27
3.10
f1 (ppm)

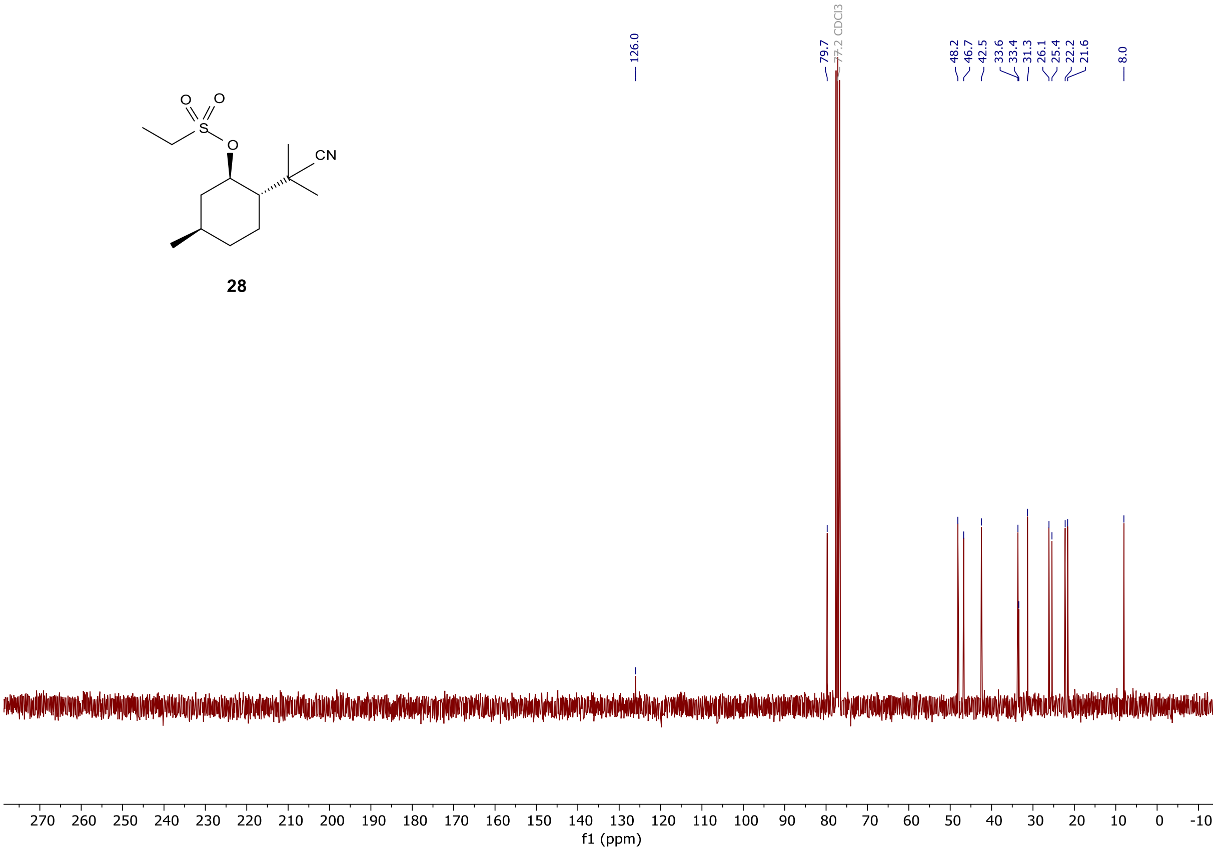
126.0
79.7
77.2 CDCl3
48.2
46.7
42.5
33.6
33.4
31.3
26.1
25.4
22.2
21.6
8.0
O
O
S
O
CN
28
f1 (ppm)

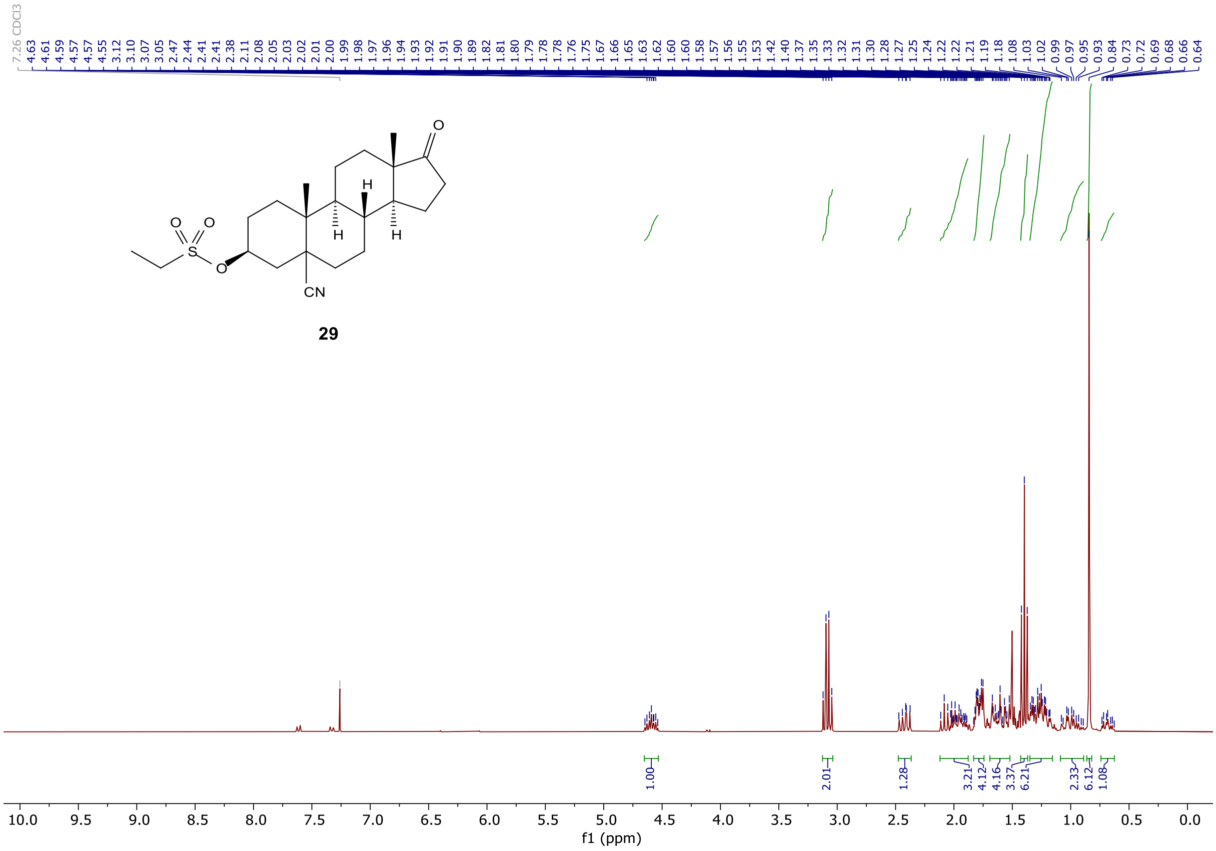
7.26 CDCl3
4.63 4.61 4.59 4.57 4.57 4.55 3.12 3.10 3.07 3.05 2.47 2.44 2.41 2.41 2.38 2.11 2.08 2.05 2.03 2.02 2.01 2.00 1.99 1.98 1.97 1.96 1.94 1.93 1.92 1.91 1.90 1.89 1.82 1.81 1.80 1.79 1.78 1.78 1.76 1.75 1.67 1.66 1.65 1.63 1.62 1.60 1.60 1.58 1.57 1.56 1.55 1.53 1.42 1.40 1.37 1.35 1.33 1.32 1.31 1.30 1.28 1.27 1.25 1.24 1.22 1.22 1.21 1.19 1.18 1.08 1.03 1.02 0.99 0.97 0.95 0.93 0.84 0.73 0.72 0.69 0.68 0.66 0.64
O
H
H
H
O
O
S
O
CN
29
1.00
2.01
1.28
3.21
4.12
4.16
3.37
6.21
2.33
6.12
1.08
10.0 9.5 9.0 8.5 8.0 7.5 7.0 6.5 6.0 5.5 5.0 4.5 4.0 3.5 3.0 2.5 2.0 1.5 1.0 0.5 0.0
f1 (ppm)

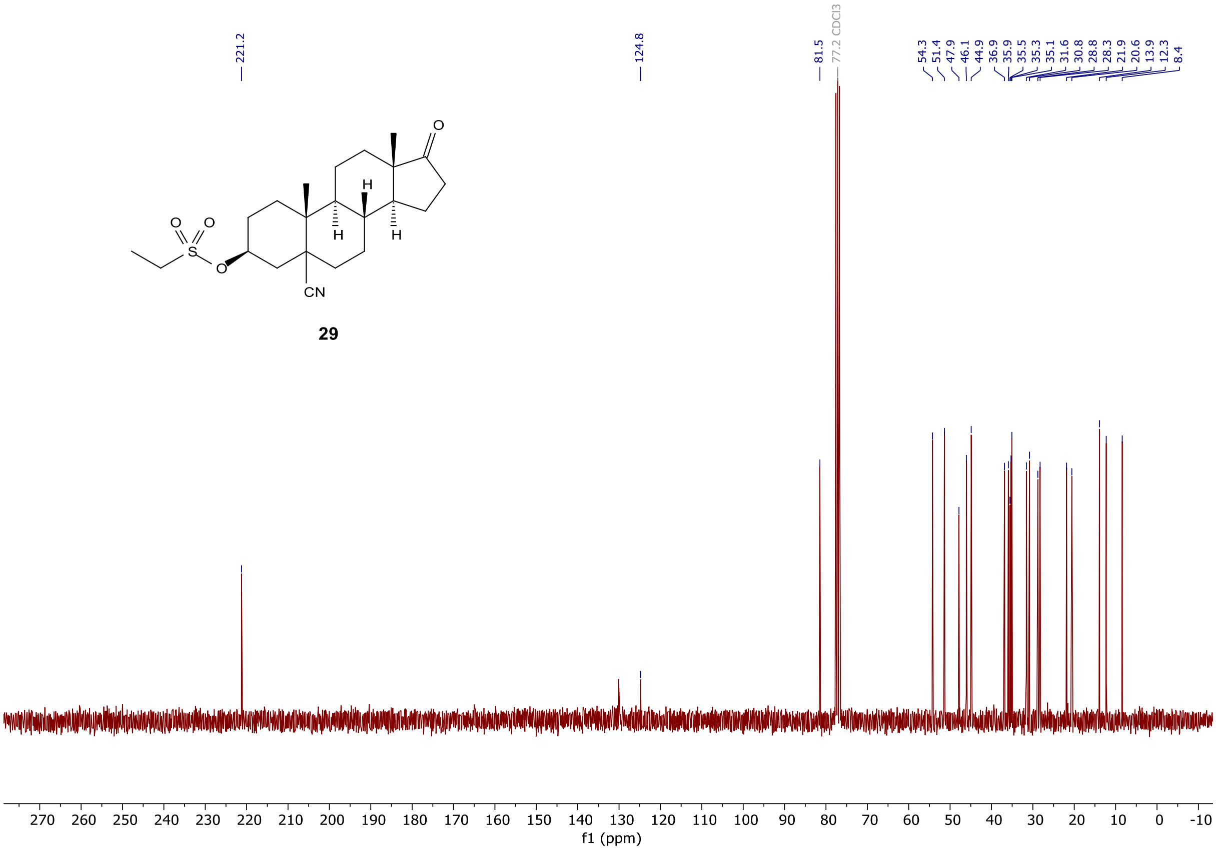
221.2
124.8
81.5
77.2 CDCl3
54.3 51.4 47.9 46.1 44.9 36.9 35.9 35.5 35.3 35.1 31.6 30.8 28.8 28.3 21.9 20.6 13.9 12.3 8.4
O
H
H
H
O
O
S
O
CN
29
270 260 250 240 230 220 210 200 190 180 170 160 150 140 130 120 110 100 90 80 70 60 50 40 30 20 10 0 -10
f1 (ppm)

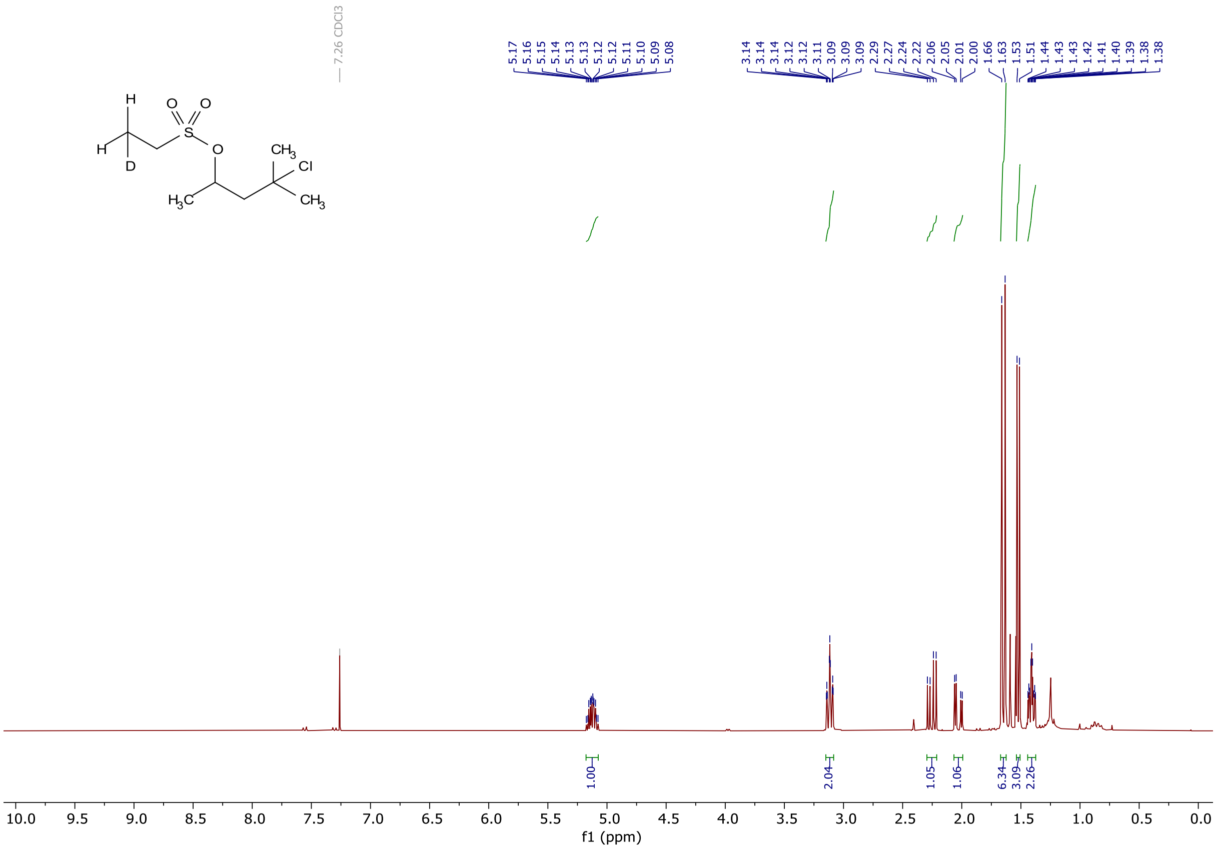
7.26 CDCl3
5.17
5.16
5.15
5.14
5.13
5.13
5.12
5.12
5.11
5.10
5.09
5.08
3.14
3.14
3.14
3.12
3.12
3.11
3.09
3.09
3.09
2.29
2.27
2.24
2.22
2.06
2.05
2.01
2.00
1.66
1.63
1.53
1.51
1.44
1.43
1.43
1.42
1.41
1.40
1.39
1.38
1.38
1.00
2.04
1.05
1.06
6.34
3.09
2.26
f1 (ppm)

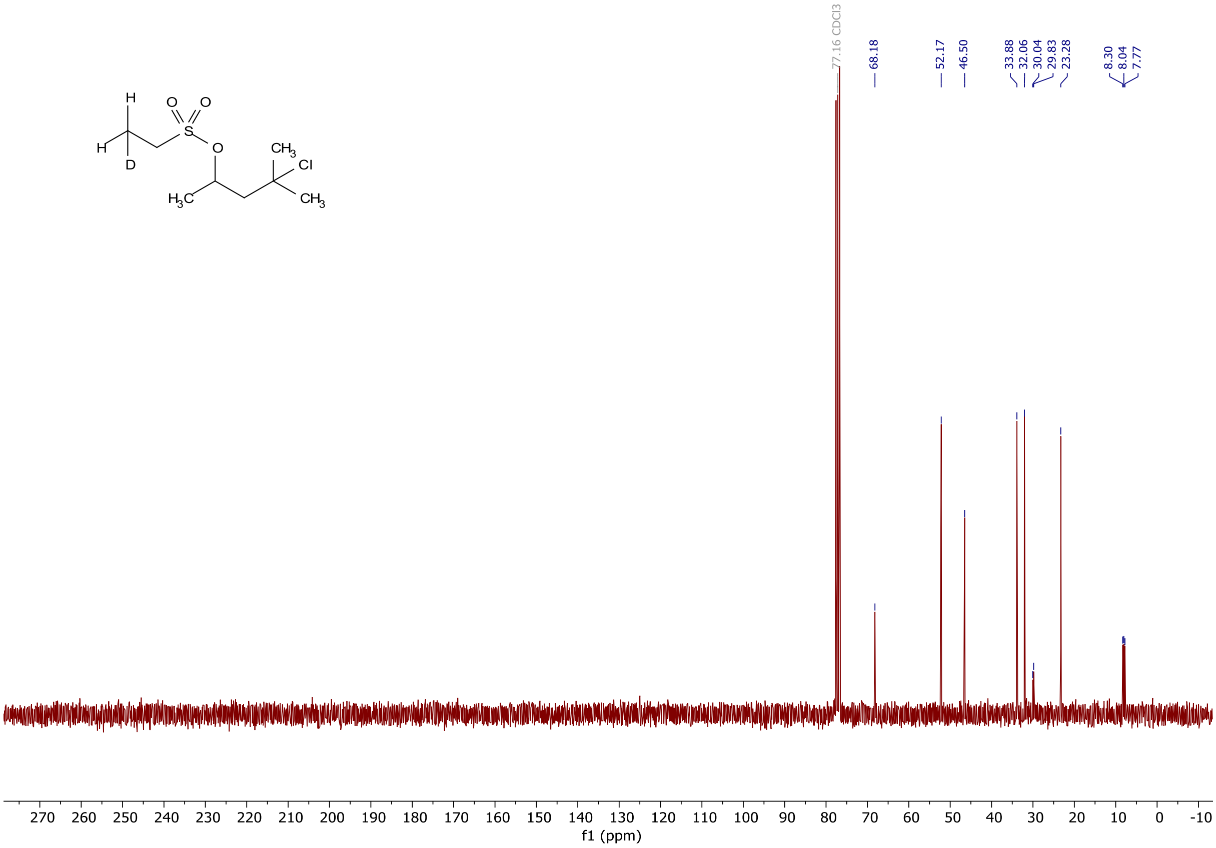
77.16 CDCl3
68.18
52.17
46.50
33.88
32.06
30.04
29.83
23.28
8.30
8.04
7.77
f1 (ppm)

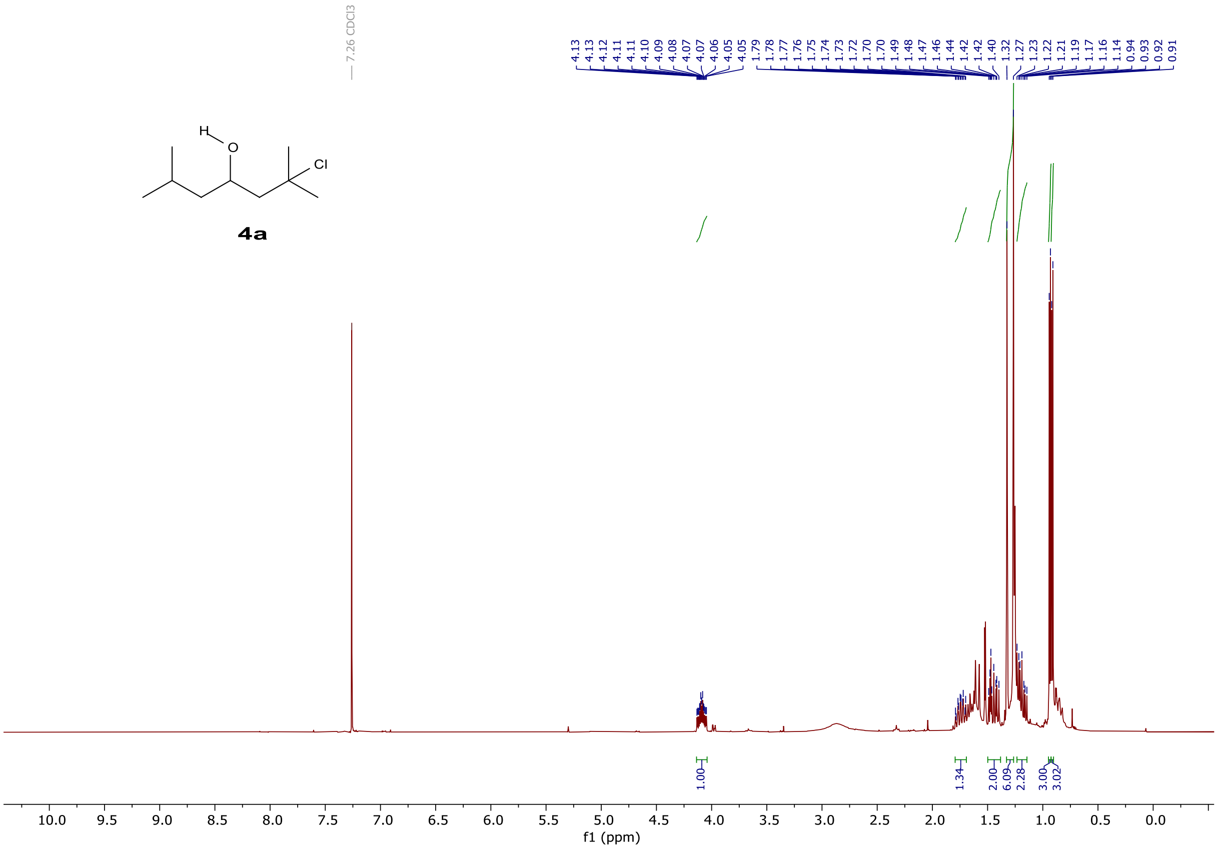
7.26 CDCl3
4.13
4.13
4.12
4.11
4.11
4.10
4.09
4.08
4.07
4.07
4.06
4.05
4.05
1.79
1.78
1.77
1.76
1.75
1.74
1.73
1.72
1.70
1.70
1.49
1.48
1.47
1.46
1.44
1.42
1.42
1.40
1.32
1.27
1.23
1.23
1.22
1.21
1.19
1.17
1.16
1.14
0.94
0.93
0.92
0.91
H
O
Cl
4a
1.00
1.34
2.00
6.09
2.28
3.00
3.02
10.0
9.5
9.0
8.5
8.0
7.5
7.0
6.5
6.0
5.5
5.0
4.5
4.0
3.5
3.0
2.5
2.0
1.5
1.0
0.5
0.0
f1 (ppm)

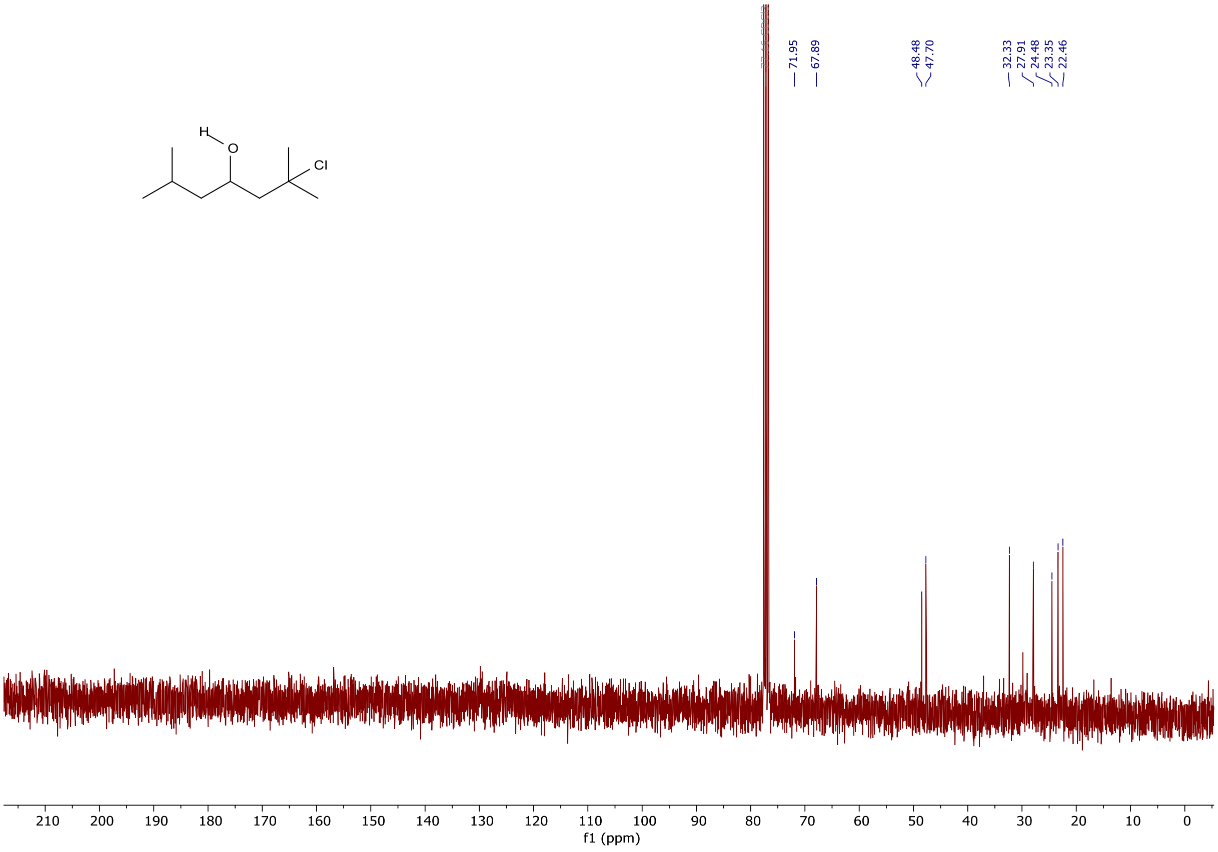
71.95
67.89
48.48
47.70
32.33
27.91
24.48
23.35
22.46
H
O
Cl
210
200
190
180
170
160
150
140
130
120
110
100
90
80
70
60
50
40
30
20
10
0
f1 (ppm)

Selected Crude NMR spectra

The crude NMR spectrum of compound 12

There is a competition between product and the reduction for primary alcohol. No other regioisomers were observed.

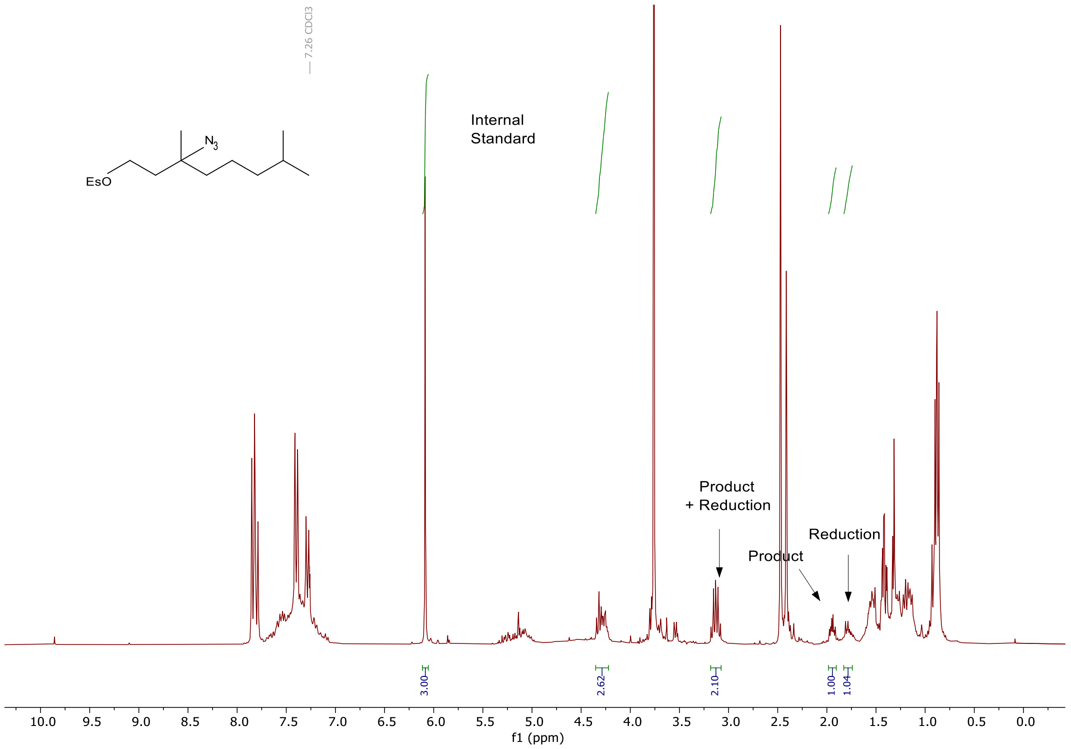

The crude NMR spectrum of compound 20

For secondary alcohol, no side reaction or other regioisomers was observed.

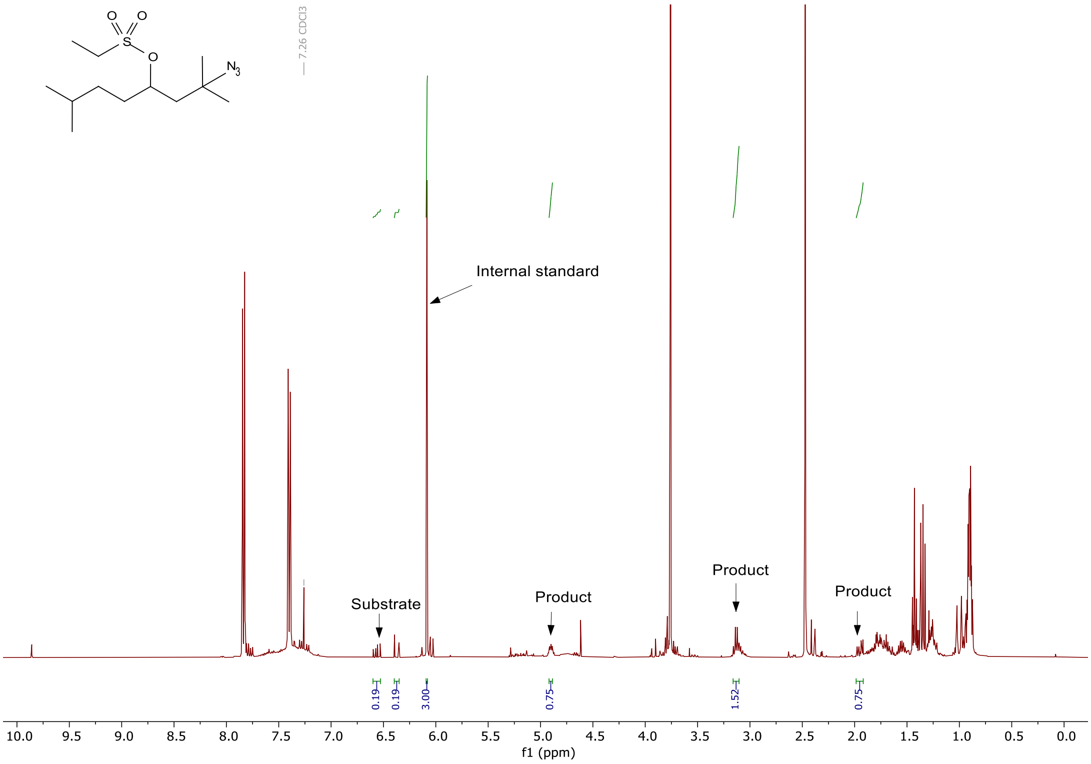

Supplement: Supplementary file 1 [file au5c00909_si_001.pdf]
